# Synthesis of the ABC Core of *Daphniphyllum* Alkaloids with a [5–6–7] Azatricyclic Scaffold via Ring Expansion of Azabicyclic and Azatricyclic Building Blocks

**DOI:** 10.1021/acs.joc.4c01090

**Published:** 2024-07-01

**Authors:** Clàudia Marquès, David González-Lizana, Faïza Diaba, Josep Bonjoch

**Affiliations:** Laboratori de Química Orgànica, Facultat de Farmàcia, Universitat de Barcelona, Av. Joan XXIII 27-31, 08028 Barcelona, Spain

## Abstract

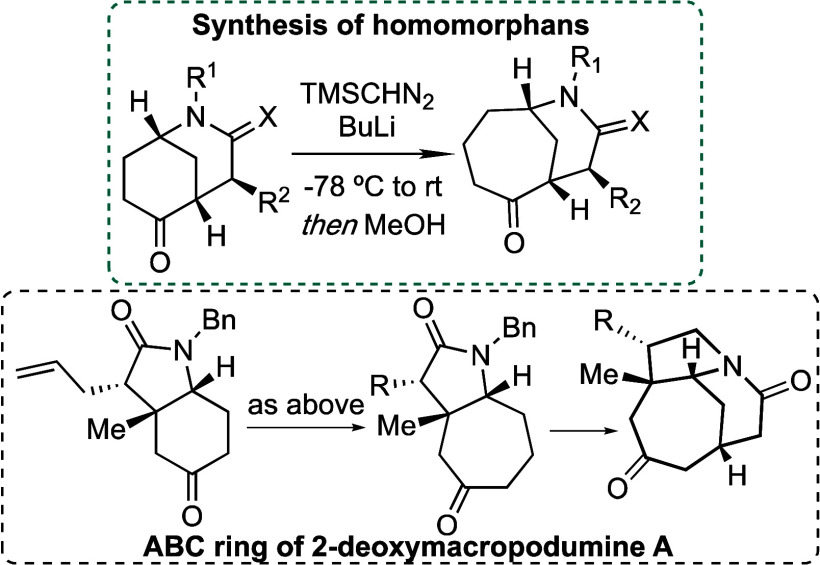

The [5–6–7] azatricyclic ABC core, found in several *Daphniphyllum* alkaloids, has been synthesized through a novel route involving ring expansion of a perhydroindolone to afford the AC ring system and a radical B ring closure as key steps. The level of functionalization of the reported octahydro-1,7-ethanocyclohepta[*b*]pyrroles suggests that they can serve as valuable building blocks in this alkaloid field. Also reported is the first synthesis of homomorphans by the ring enlargement of 2-azabicyclo[3.3.1]nonanes.

## Introduction

**Figure 1:**
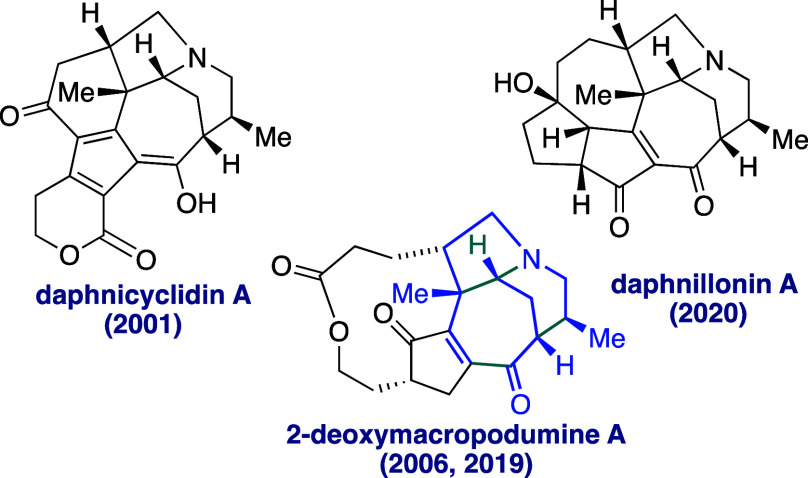
*Daphniphyllum* alkaloids containing the azatricyclic 7/6/5 fragment (ABC core).

Among the plethora of *Daphniphyllum* alkaloids,^1^ some possess a distinctive bridged 7-azabicyclo[4.3.1]decane ring system embedded in their skeleton. Representative alkaloids with this structural feature, which is a homoanalogue of the morphan nucleus, include daphnicyclidins,^2^ daphnillonins,^3^ and macropodumines^4^ (Figure 1). Additionally, a fused pyrrolidine ring completes the characteristic ABC framework of octahydro-1,7-ethanocyclohepta[*b*]pyrrole (colored blue in Figure 1).

Due to their structural complexity, the first total synthesis of one of these alkaloids, daphnillolin B, was not achieved until 2023 by Li.^5^ Also noteworthy are the total syntheses of several *Daphniphyllum* alkaloids recently reported by Li.^9^ Moreover, only a few studies have reported synthetic strategies toward compounds embodying tricyclic scaffolds (ABC rings)^6^ as potential advanced precursors of the target alkaloids.

**Scheme 1:**
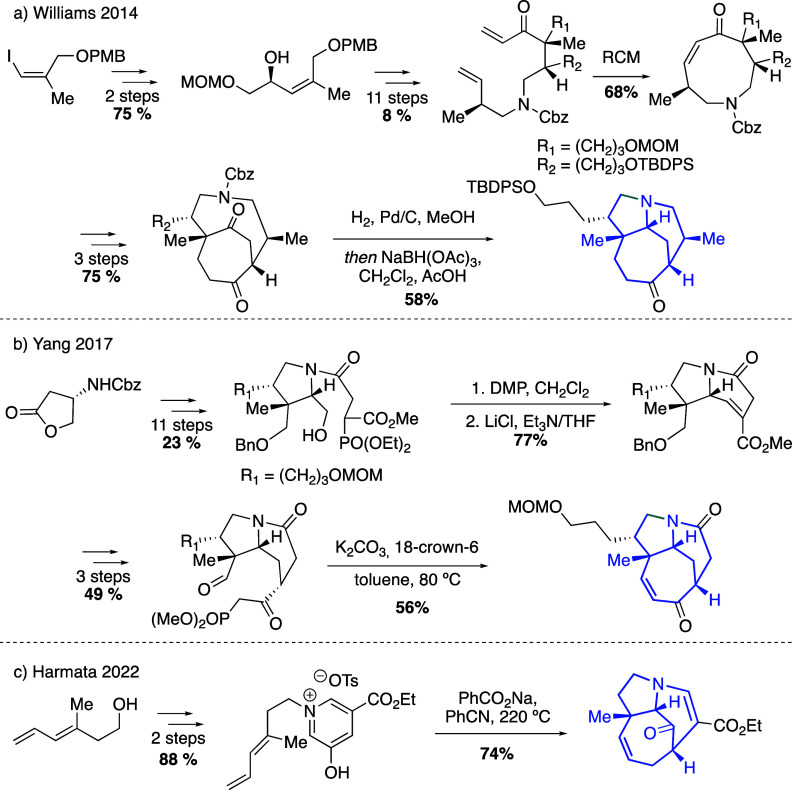
Precedents for the Synthesis of the ABC [7,6,5] Ring System

Previous synthetic approaches to the ABC tricyclic substructure of perhydro-7,1-ethanocyclohepta[*b*]pyrrole have been described in studies of the synthesis of daphnicyclidin A (Scheme 1). (a) Williams^6a^ transformed an intermediate bearing a nine-membered ring into an azabicyclic dione, which after an intramolecular reductive amination furnished the targeted compound. (b) The synthesis of Yang^6b^ involved a 2,3,4-*cis* trisubstituted pyrrolidine as an advanced intermediate, which allowed the construction of rings A and B by means of two intramolecular Horner–Wadsworth–Emmons reactions. (c) Harmata^6c,6d^ described an intramolecular [4+3] cycloaddition of the salt generated from N-alkylation of a 5-hydroxynicotinic acid with a dienyl tosylate.

**Scheme 2:**
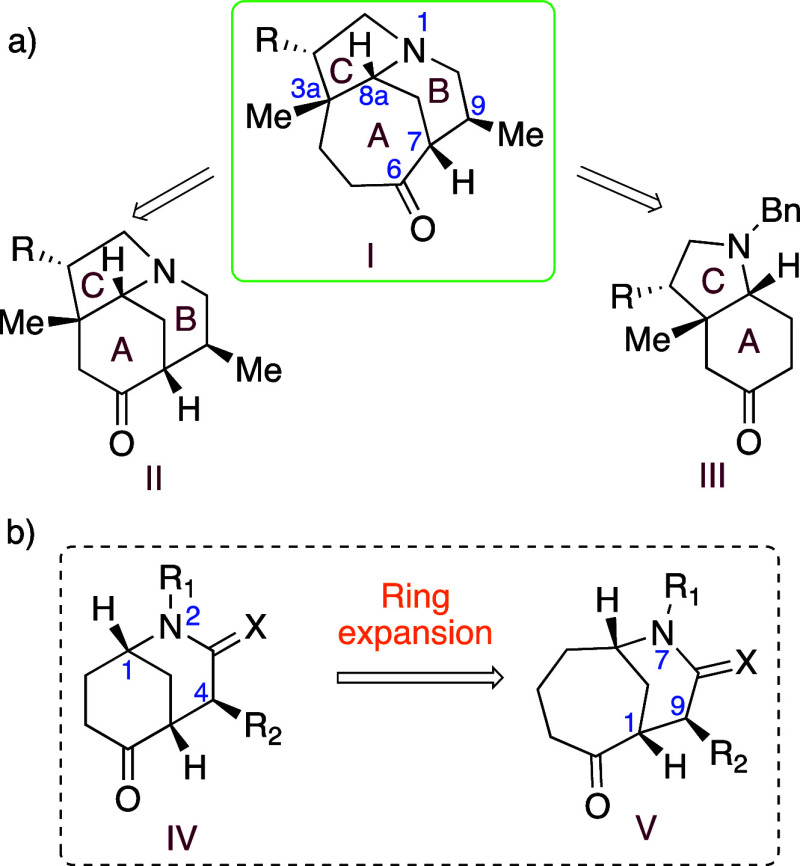
Retrosynthetic Strategy for the Assembly of Azatricyclo **I** and Synthesis of Homomorphans **V**

Gaining access to functionalized building blocks bearing this ABC skeleton would constitute a starting point for exploring new strategies toward the aforementioned alkaloids. Our interest was focused on accessing valuable advanced structures to facilitate studies aimed at the synthesis of 2-deoxymacropodumine A (Figure 1). With this objective, we planned to synthesize a functionalized azatricyclic building block with scaffold **I** through two approaches, starting from either functionalized azatricyclo **II** or azabicyclo **III**, each of which would undergo expansion of the cyclohexanone A ring to the corresponding seven-membered ring^7,8^ (Scheme 2). To evaluate the synthetic protocol for the ring enlargement, easily available morphans (**IV**) bearing a basic nitrogen atom or lactam unit would be used in preliminary studies to obtain homomorphan derivatives (**V**). Subsequently, the tested methodology would be applied to develop a new route to key building block **I**.

**Scheme 3:**
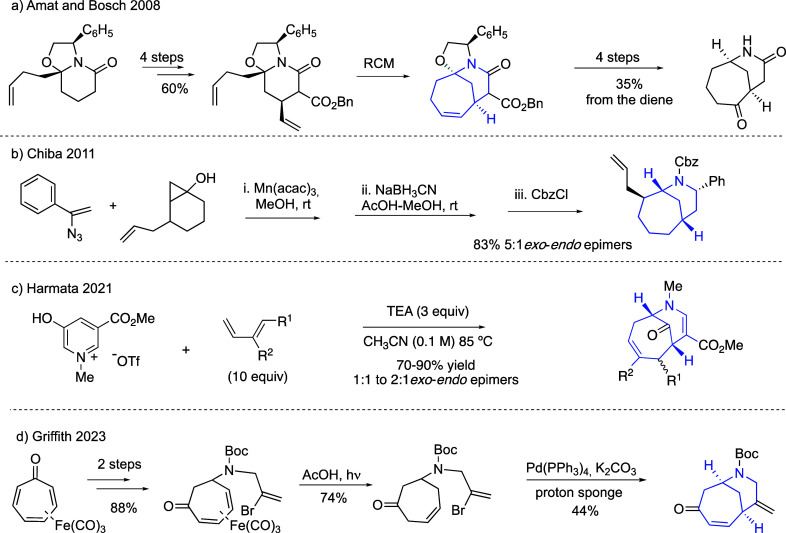
Synthetic Precedents for Homomorphans

In the field of *Daphniphyllum* alkaloid synthesis, despite significant development in recent decades,^9^ few synthetic studies have focused on alkaloids embodying aforementioned structure **I** (Scheme 2), whereas the use of compounds featuring azatricyclic **II** or azabicyclic **III** units as building blocks is unprecedented.^8^ Furthermore, few synthetic pathways have been reported so far for homomorphan-type bicyclic compounds (Scheme 3). The first synthesis, developed in Amat’s group,^10^ was based on a ring-closing metathesis from a functionalized piperidine compound, which gave access to a potentially valuable keto lactam (Scheme 3a). In Chiba’s radical procedure,^11^ a vinyl azide was used as a radical precursor and a Mn(III) as a promoter in a straightforward route to the azabicyclic ring (Scheme 3b). The generation of the homomorphan ring via (4+3) cycloaddition of oxidopyridinium ions was introduced by Harmata (Scheme 3c).^12^ Recently, Griffith^13^ reported a new approach to the synthesis of the 7-azabicyclo[4.3.1]decane ring system using an intramolecular Heck reaction (Scheme 3d).

## Results and Discussion

### Preliminary Studies. Ring Enlargement of Morphans: Synthesis of 7-Azabicyclo[4.3.1]decan-3-ones

**Scheme 4:**
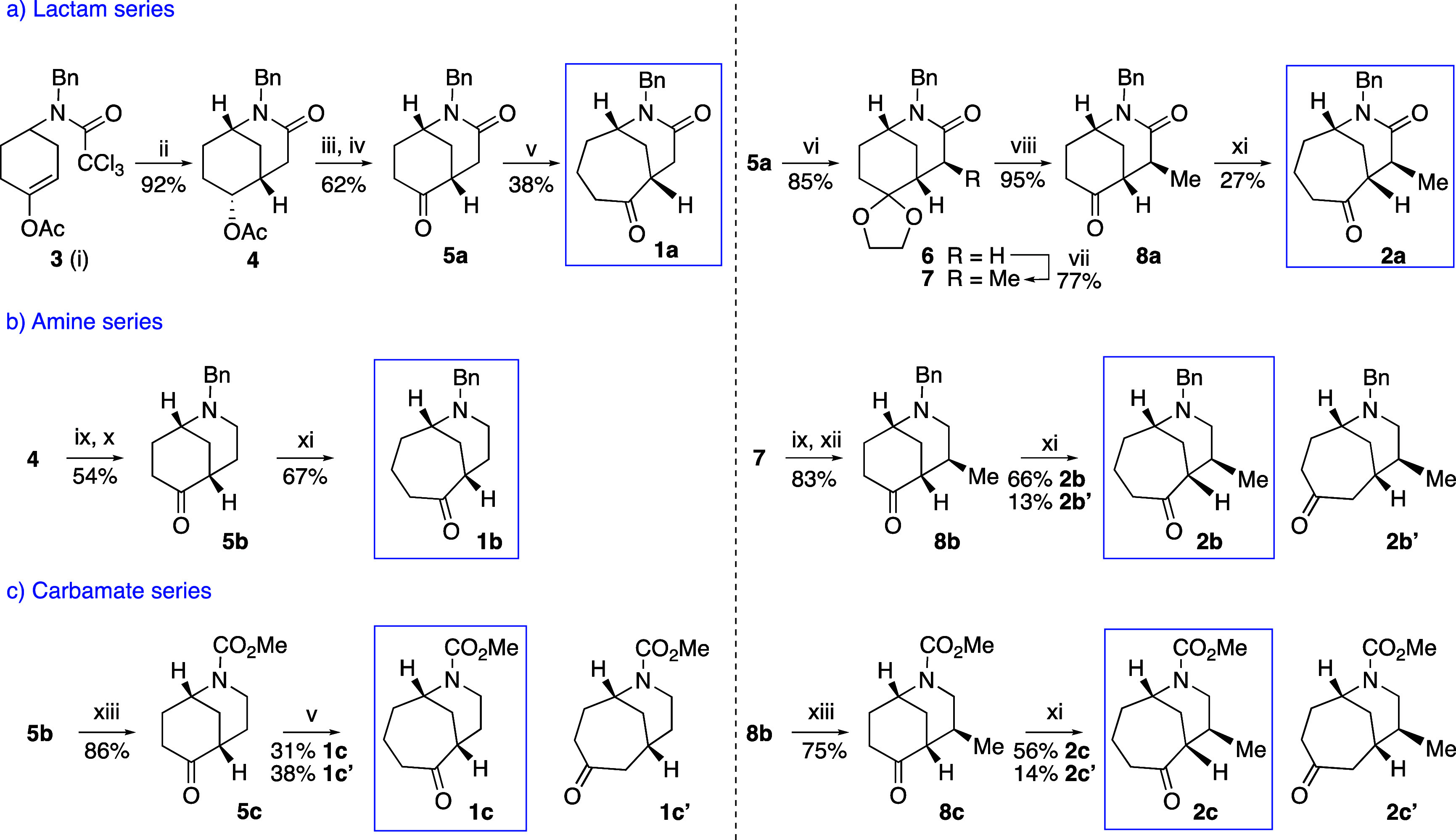
Ring Expansion of Morphans: Synthesis of Homomorphans (**1a**–**c** and **2a**–**c**) (i) Compound **3** was synthesized in five steps (66% overall yield) from 1,4-cyclohexanedione monoethylene acetal;^16^ (ii) Bu_3_SnH, AIBN, benzene, reflux; (iii) NaOH, EtOH, reflux; (iv) Dess-Martin periodinane, CH_2_Cl_2_, rt; (v) TMSCHN_2_, BF_3_·OEt_2_, CH_2_Cl_2_, rt; (vi) (CH_2_OH)_2_, TsOH, benzene, reflux; (vii) LDA, MeI, −78 °C to rt; (viii) 10% HCl, THF, rt; (ix) LiAlH_4_, AlCl_3_, THF, rt; (x) DMP, NaHCO_3_, CH_2_Cl_2_, rt; (xi) TMSCHN_2_, BuLi, Et_2_O/THF, −78 °C; then MeOH and SiO_2_, −78 °C to rt; (xii) 10% HCl, rt; (xiii) ClCO_2_Me, NaHCO_3_, CHCl_3_, reflux.

Initially, we decided to study the unprecedented carbocyclic ring expansion of morphan compounds from ketones **5** and **8** (series a–c) employing diazo derivative reagents,^14^ evaluating the regioselectivity of the Tiffeneau–Demjanov procedure, as well as the influence of the nitrogen atom functionality (amine, lactam, or carbamate) on the overall process (Scheme 4). The required morphans were prepared following the methodology developed by our group as outlined in Scheme 4.^15^

The results reported here constitute a new approach to the synthesis of B-homomorphans. Two different methodologies were tested using trimethylsilyl diazomethane in the presence of either a Lewis acid^17^ or a base.^18^ The latter procedure, in which the ring expansion step was triggered by nucleophilic addition of deprotonated TMSCHN_2_ to the ketone in compounds **5** and **8**, followed by two protonation steps with MeOH and SiO_2_,^19^ gave better yields for the homomorphans (compounds **1** and **2**, series a–c). This process also provided the desired ring-enlargement product with better regioselectivity, with the carbonyl group remaining at the contiguous bridgehead carbon atom, as in the target alkaloids. Notably, the results in the amino series (**1b** and **2b**) were better than the poor yields observed in the lactam series (e.g., **1a** vs **1b**).

**Scheme 5:**
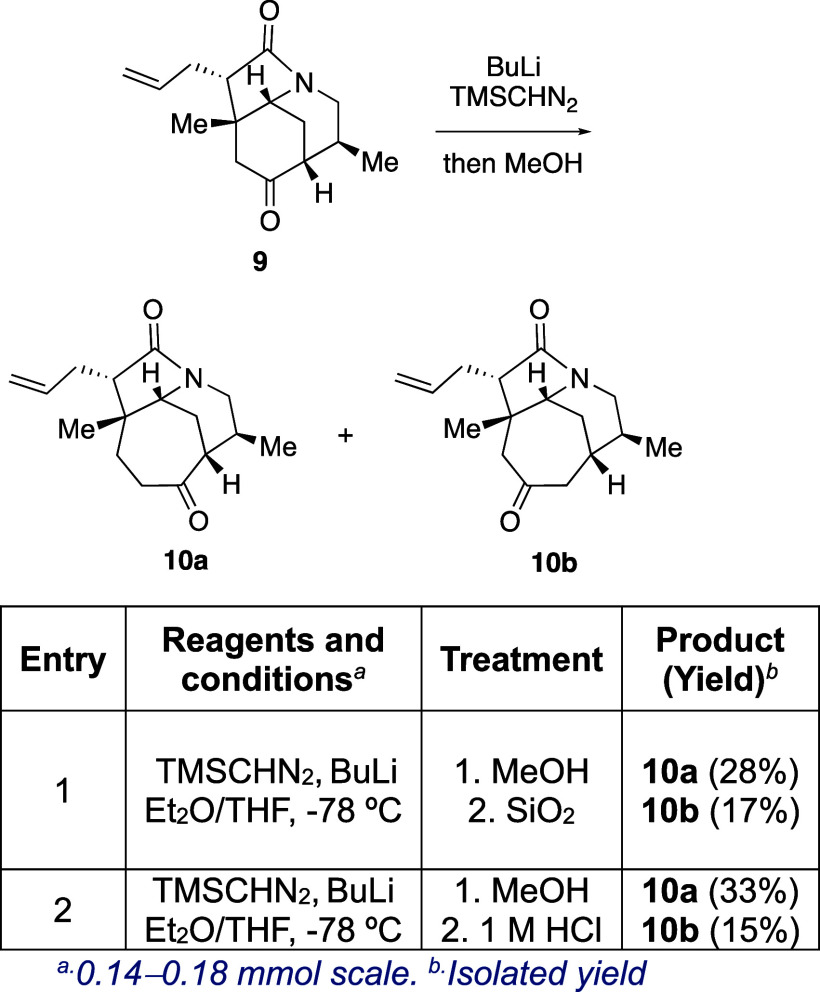
Synthesis of the 7,6,5-Azatricyclic Scaffold by Ring Expansion of the Carbocyclic Ring in **9**

At this point, we decided to test the ring expansion method with a more demanding substrate, namely azatricyclo **9**, which has a 6,6,5 ring system (Scheme 5) and was previously synthesized by our group in a formal synthesis of *rac*-himalensine A.^20^ The enlargement of the cyclohexanone subunit using the same reaction conditions as in the morphan series was not regioselective, and a 2:1 mixture of ketones **10a** and **10b** was isolated in 46% overall yield. This result was not sufficiently satisfactory to justify continuing with this approach toward the target compound, especially considering the laborious preparation of azatricyclo **9** [type II scaffold (Scheme 1)].

After these studies, we turned our attention to developing a method based on the ring expansion of an octahydroindole [type III (Scheme 2)] for an efficient synthesis of a functionalized octahydro*-cis*-cyclohepta[*b*]pyrrole.^21^ The latter would then serve as an advanced intermediate en route to the functionalized 7,6,5-targeted azatricyclic scaffold.

**Scheme 6:**
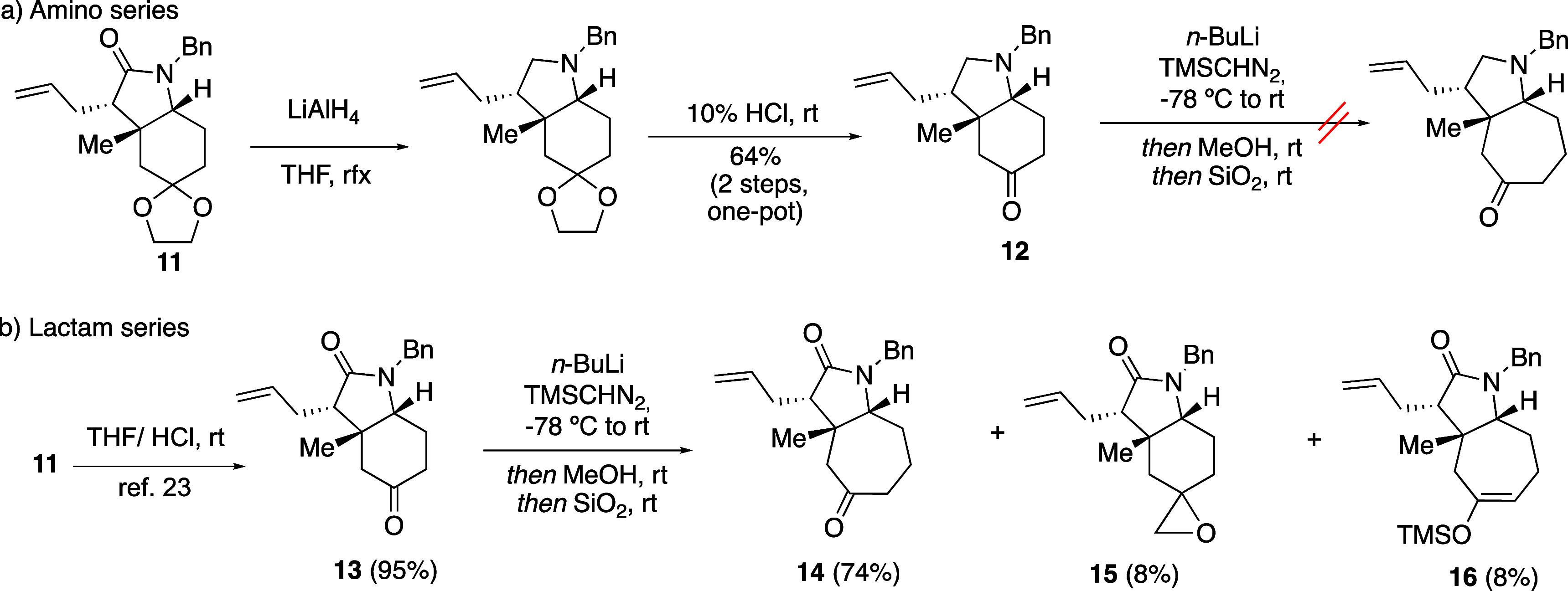
Ring Expansion Process from Octahydroindoles **12** and **13**

On the basis of the results of the B-homomorphan synthesis, initially an amino derivative was prepared (Scheme 6a). Starting from octahydroindole **11**, previously described in our group,^22^ we reduced the lactam moiety, and further ketal hydrolysis provided amino ketone **12** in 64% over two steps. However, upon the reaction of ketone **13a** with TMSCHN_2_ and BuLi and further treatment with MeOH and SiO_2_, only complex mixtures of unidentifiable products were observed.

**Scheme 7:**
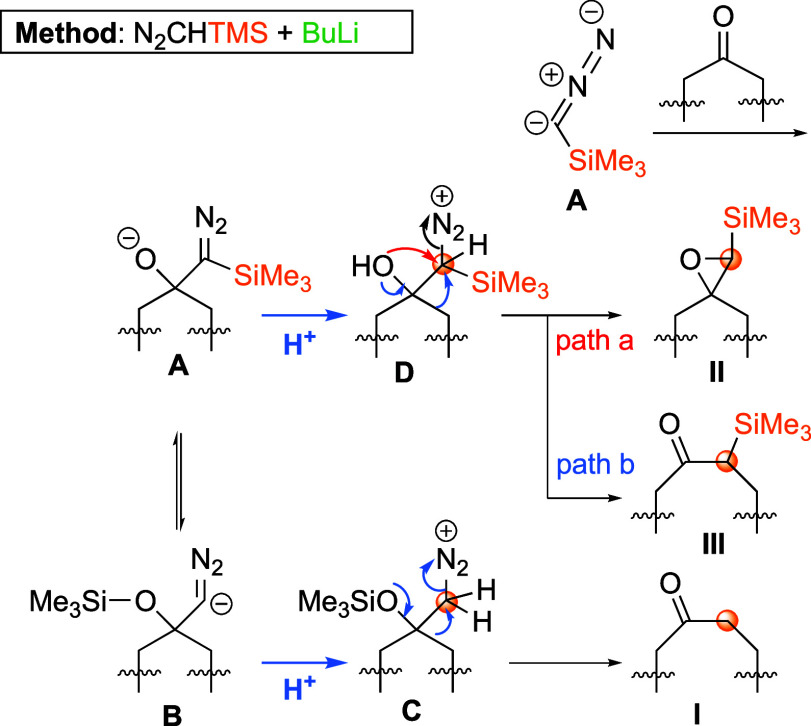


However, when the ketone homologation reaction was tested directly on octahydroindole **13**(23) (Scheme 6b), by treatment with a solution of TMSCHN_2_ and BuLi, only regioisomer **14** was obtained in good yield. Epoxide **15** and silylenol ether **16** derivatives were also isolated but in low yields. Scheme 7 depicts the proposed mechanism^14b^ for the formation of the desired ketone **14** and byproducts **15** and **16**. Thus, treatment of TMSCHN_2_ with BuLi and further addition of the more nucleophilic species **A** to the ketone substrate rendered intermediate **B**. As the ring expansion occurred after the key protonation step, giving **C** or **D**, multiple carbon insertions were avoided. Moreover, the choice of protonation source could determine the proportion of the obtained compounds (**I**–**III**).

**Scheme 8:**
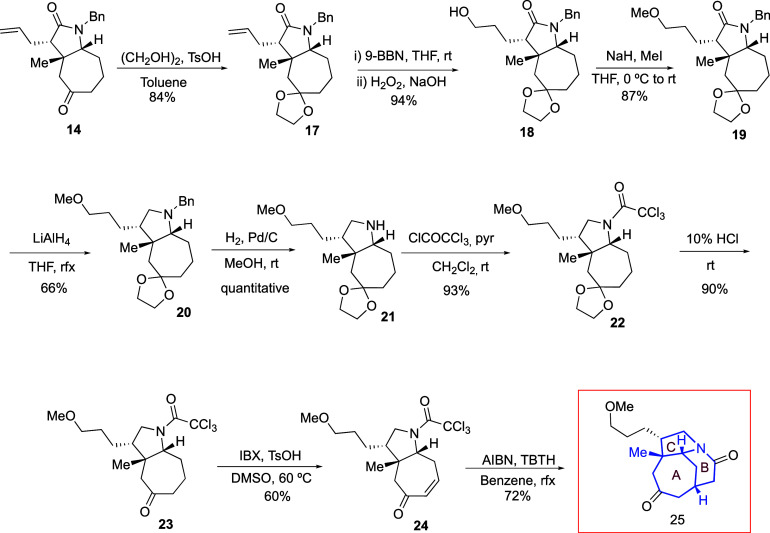
Synthesis of 5/6/7 Azatricyclic Building Block **25** from Azabicyclo **14**

Considering the good yield obtained for **14**, a new synthetic route was explored for the construction of the azatricyclic [5–6–7] ring core (Scheme 8). Thus, after ketone protection, hydroboration and oxidation of the allylic moiety in **17** gave alcohol **18** in 94% yield. Subsequent hydroxyl protection as a methoxy group by treatment with NaH and iodomethane afforded compound **19** in 87% yield.^24^ The reduction of lactam **19** provided tertiary amine **20**, which after N-debenzylation under hydrogenation conditions gave secondary amine **21**. The latter, upon treatment with trichloroacetyl chloride, afforded trichloroacetamide **22**. After hydrolysis of the acetal group, ketone **23** was isolated in 54% overall yield for the four-step sequence.

Taking advantage of the location of the C-5 carbonyl, we decided to prepare an enone that would allow radical closure of the B ring. Thus, trichloroacetamide **23** was oxidized with IBX in the presence of *p*-TsOH^25^ to furnish enone **24**, which was isolated in 60% yield, along with traces of an overoxidized compound **24b** (see the Experimental Section). Finally, we addressed the B ring closure, subjecting trichloroacetamide **24** to reductive radical conditions by the slow addition of AIBN and TBTH over 4 h.^26^ This resulted in the formation of keto-lactam **25** bearing the azatricyclic [5–6–7] fragment in 72% yield.

Notably, the regioselectivity attained in the ring expansion of azabicyclic ketone **13**, resulting in the formation of ketone **14**, afforded valuable azatricyclic ketone **25**. The relative position of the carbonyl group in **25** is consistent with that of alkaloids bearing a 5–6–7 azatricyclic subunit. Interestingly, this regioselectivity differs from that reported by Xie and She,^8^ who applied the ring expansion to a related compound also under the Tiffeneau–Demjanov reaction conditions.

## Conclusions

In summary, the [5,6,7] azatricyclic core of 2-deoxymacropodumine A was achieved via a ring expansion process for the installation of the seven-membered ring. The ketone homologation methodology was first applied to morphan compounds, providing a new pathway for the synthesis of homomorphans. More notably, its subsequent application to octahydroindolones (AC ring of the target alkaloids) allowed us to establish a novel synthetic route to ABC azatricyclic building blocks for use in alkaloid synthesis. The reported results pave the way for a synthetic proposal aimed at achieving the total synthesis for 2-deoxymacropodumine A.

## Experimental Section

### General

All reactions were carried out under an argon atmosphere with dry, freshly distilled solvents under anhydrous conditions. An oil bath was used as the heat source for the reactions that require heating. All product mixtures were analyzed by thin-layer chromatography using TLC silica gel plates with a fluorescent indicator (λ = 254 nm). Analytical thin-layer chromatography was performed on SiO_2_ (Merck silica gel 60 F_254_), and the spots were located by ultraviolet light and/or an aqueous 1% KMnO_4_ solution or hexachloroplatinate reagent. Chromatography refers to flash chromatography and was carried out on SiO_2_ (VWR 60, 40–63 μm) or Al_2_O_3_ (neutral aluminum oxide, 0.063–0.2 mm). Organic extracts were dried during the reaction workup over anhydrous Na_2_SO_4_. Organic solvents were removed under vacuum using a rotatory evaporator. The reaction conditions for the ring expansion (for the safe use and quenching of TMSCHN_2_) are those reported by Gaich in ref (19). The small scale of the reactions minimized the potential risk of the reagents used. Chemical shifts of ^1^H and ^13^C nuclear magnetic resonance (NMR) spectra are reported in parts per million downfield (δ) from Me_4_Si (δ 0.00) and CDCl_3_ (δ 77.00), respectively. All NMR data assignments are supported by gCOSY and gHSQC experiments. HRMS were obtained with an LC/MSD-TOF spectrometer (Agilent technologies, ESI-MS).

### Synthesis of Morphans **5** and **8**

Compound **5a** was prepared following our previously reported procedure.^27^

#### (1*RS*,5*RS*)-2-Benzyl-2-azabicyclo[3.3.1]nonan-6-one (**5b**)

To a solution of AlCl_3_ (1.36 g, 9.9 mmol, 1.5 equiv) in tetrahydrofuran (THF, 30 mL) was added a 1 M solution of LiAlH_4_ in THF (16.6 mL, 16.6 mmol, 2.5 equiv) at 0 °C, and the mixture was stirred for 20 min at room temperature (rt). Then, a solution of morphan **4** (1.9 g, 6.6 mmol) in THF (70 mL) was added dropwise via cannula, and the reaction mixture was stirred overnight at rt. The reaction was quenched with a 30% KOH solution, and the mixture extracted sequentially with CH_2_Cl_2_ (3 × 30 mL), CHCl_3_ (3 × 25 mL), and a CHCl_3_/*i*-PrOH mixture (4:1, 2 × 25 mL). The organics were dried and concentrated to yield the corresponding aminoalcohol that was pure enough to be used in the next step without further purification. The residue (1.35 g, 5.8 mmol) was diluted in CH_2_Cl_2_ (100 mL), and NaHCO_3_ (3.66 g, 43.8 mmol, 7.5 equiv) was added followed by Dess-Martin periodinane (4.94 g, 11.6 mmol, 2.5 equiv). The reaction mixture was stirred at room temperature for 3 h, before the reaction was quenched with a saturated Na_2_S_2_O_3_/Na_2_CO_3_ solution (1:1, 130 mL). After being stirred for 30 min, the mixture was poured into water (100 mL) and extracted with CH_2_Cl_2_ (3 × 100 mL). The combined organics were dried, filtered, and concentrated to obtain a yellow crude residue. After chromatography (Al_2_O_3_, 9:1 hexane/EtOAc), compound **5b** was isolated (0.82 g, 54% over two steps) as a transparent oil: IR (NaCl) 2932, 2808, 1705, 1447, 1123 cm^–1^; ^1^H NMR (400 MHz, CDCl_3_) δ 7.36–7.23 (m, 5H, Ph), 3.68 and 3.60 (2d, *J* = 13.2 Hz, 1H each, CH_2_Ph), 3.09 (br s, 1H, H-1), 2.69 (ddd, *J* = 12.4, 6.0, 2.2 Hz, 1H, H-3), 2.57–2.50 (m, 2H, H-5 and H-7), 2.48 (ddd, *J* = 12.4, 12.4, 4 Hz, 1H, H-3), 2.38 (dd, *J* = 18.0, 7.4 Hz, 1H, H-7), 2.30–2.22 (m, 1H, H-8), 2.08 (ddt, *J* = 13.6, 2.8, 2.8 Hz, 1H, H-9), 2.00–1.91 (m, 2H, H-9 and H-4), 1.81–1.69 (m, 2H, H-4 and H-8); ^13^C{^1^H} NMR (101 MHz, CDCl_3_) δ 216.5 (C-6), 138.9, 128.7, 128.2, and 126.9 (Ph), 59.6 (CH_2_Ph), 49.7 (C-1), 46.0 (C-3), 42.8 (C-5), 38.8 (C-7), 31.7 (C-9), 29.2 (C-4), 21.1 (C-8); HRMS (ESI-TOF) *m*/*z* [M + H]^+^ calcd for C_15_H_20_NO 230.1539, found 230.1543.

#### (1*RS*,5*RS*)-2-Methoxycarbonyl-2-azabicyclo[3.3.1]nonan-6-one (**5c**)^28^

To a solution of compound **5b** (0.12 g, 0.52 mmol, 1 equiv) in dry CHCl_3_ (2 mL) were added NaHCO_3_ (0.66 g, 7.9 mmol,15 equiv) and methyl chloroformate (0.84 g, 0.69 mL, 8.9 mmol, 17 equiv). The reaction mixture was placed in a sealed tube and stirred at reflux overnight. Then, the mixture was cooled to room temperature and diluted with CH_2_Cl_2_ (10 mL), and the organic layer was washed with water, dried, and concentrated. The crude residue was purified by chromatography (1:0 → 3:1 hexane/EtOAc), providing **5c** as a colorless oil (89 mg, 86%): IR (neat) 2942, 1695, 1450, 1215 cm^–1^; ^1^H NMR (400 MHz, CDCl_3_) δ 4.59 and 4.44 (2 bs, 1H, H-1), 4.02–3.91 (m, 1H, H-3), 3.74 (s, 3H, OMe), 3.22 (dd, *J* = 13.6 Hz, 1H, H-3), 2.66 (m, 2H, H-5), 2.54–2.48 (m, 2H, H-7) 2.11 (br s, 2H, H-8), 2.01 (br s, 2H, H-9), 1.90–1.88 (m, 2H, H-4); ^13^C{^1^H} NMR (101 MHz, CDCl_3_) δ 214 and 213.6 (C-6), 156.3 (CO_2_Me), 52.6 (OMe), 44.8 and 44.6 (C-1), 42.2 (C-5), 38.9 (C-3), 37.7(C-7), 31.0 and 30.8 (C-9), 29.4 and 28.7 (C-8), 27.9 and 27.6 (C-4). The NMR spectroscopy data matched the previously reported data.

#### (1*RS*,5*SR*)-2-Benzyl-2-azabicyclo[3.3.1]nonan-3,6-dione Ethylene Acetal (**6**)

A solution of morphan **5a** (0.71 g, 2.9 mmol, 1 equiv), *p-*TsOH (0.22 g, 1.16 mmol, 0.4 equiv), and ethylene glycol (8.1 mL, 145 mmol, 50 equiv) in toluene (70 mL) was placed in a Dean-Stark setup and heated to reflux for 4 h. After the mixture had been cooled, the reaction was quenched with a saturated NaHCO_3_ solution (50 mL). The aqueous layer was extracted with EtOAc (4 × 20 mL), and the combined organics were washed with brine (1 × 40 mL), dried, filtered, and concentrated to obtain a yellow crude product. After purification (1:0 → 3:1 CH_2_Cl_2_/EtOAc), product **6** was obtained (0.71 g, 85%) as a colorless oil: IR (NaCl) 2949, 1635, 1452, 1107 cm^–1^; ^1^H NMR (400 MHz, CDCl_3_) δ 7.33–7.25 (m, 5H, Ph), 5.27 (d, *J* = 15.2 Hz, 1H, CH_2_Ph), 4.01–3.88 (m, 5H, OCH_2_ and CH_2_Ph), 3.44 (br s, 1H, H-1), 2.75 (d, *J* = 18.8 Hz, 1H, H-4), 2.62 (dd, *J* = 18.8, 7.2 Hz, 1H, H-4), 2.09–2.08 (m, 1H, H-5), 2.03 (br d, *J* = 13.0 Hz, 1H, H-9), 1.83–1.78 (m, 2H, H-9 and H-7), 1.67–1.57 (m, 3H, H-7 and H-8); ^13^C{^1^H} NMR (101 MHz, CDCl_3_) δ 170.1 (C-3), 137.6, 128.6, 127.8, and 127.3 (Ph), 109.6 (C-6), 64.6 and 64.5 (OCH_2_), 50.2 (C-1), 48.1 (CH_2_Ph), 36.2 (C-5), 34.0 (C-4), 29.9 (C-9), 27.2 (C-7), 27.0 (C-8); HRMS (ESI-TOF) *m*/*z* [M + H]^+^ calcd for C_17_H_22_NO_3_ 288.1594, found 288.1604.

#### (1*RS*,4*RS*,5*SR*)-2-Benzyl-4-methyl-2-azabicyclo[3.3.1]nonan-3,6-dione Ethylene Acetal (**7**)

To a solution of lactam **6** (0.62 g, 2.19 mmol, 1 equiv) in THF (24 mL) cooled at −78 °C was added dropwise a solution of LDA (1 M in THF, 4.4 mL, 2 equiv). After the mixture was stirred for 1 h, iodomethane (0.4 mL, 5.9 mmol, 2.7 equiv) was added, and the stirring was prolonged for 3 h. A saturated NH_4_Cl solution (20 mL) was added, and the mixture was extracted with EtOAc (4 × 20 mL). The organics were dried, filtered, and concentrated. The crude residue was purified by chromatography (1:0 → 4:1 CH_2_Cl_2_/EtOAc), and compound **7** was obtained (0.50 g, 77%) as a colorless oil: IR (NaCl) 2937, 1634, 1449, 1113 cm^–1^; ^1^H NMR (400 MHz, CDCl_3_) δ 7.34–7.22 (m, 5H, Ph), 5.28 (d, *J* = 14.8 Hz, 1H, CH_2_Ph), 3.99–3.87 (m, 5H, OCH_2_ and CH_2_Ph), 3.40 (br s, 1H, H-1), 2.78 (q, *J* = 7.4 Hz, 1H, H-4), 1.91 (br s, 2H, H-9), 1.81–1.69 (m, 3H, H-5 and H-8), 1.65–1.56 (m, 2H, H-7), 1.39 (d, *J* = 7.4 Hz, 3H, Me); ^13^C{^1^H} NMR (101 MHz, CDCl_3_) δ 173.9 (C-3), 137.8, 128.6, 127.7, and 127.3 (Ph), 109.5 (C-6), 64.5 and 64.5 (OCH_2_), 50.5 (C-1), 47.9 (CH_2_Ph), 43.6 (C-5), 37.9 (C-4), 27.1 (C-7), 26.9 (C-8), 26.6 (C-9), 20.4 (Me); HRMS (ESI-TOF) *m*/*z* [M + H]^+^ calcd for C_18_H_24_NO_3_ 302.1751, found 302.1753.

#### (1*RS*,4*RS*,5*SR*)-2-Benzyl-4-methyl-2-azabicyclo[3.3.1]nonane-3,6-dione (**8a**)

To a solution of lactam **7** (113 mg, 0.38 mmol) in THF (4 mL) was added a 10% HCl solution (8 mL). After the mixture was stirred overnight at room temperature, Na_2_CO_3_ (20 mL) was added, and the mixture was extracted with CH_2_Cl_2_ (4 × 10 mL). The organics were dried, filtered, concentrated, and purified by chromatography (1:0 → 9:1 CH_2_Cl_2_/EtOAc). Compound **8a** was obtained as a colorless oil (92 mg, 95%): ^1^H NMR (400 MHz, CDCl_3_) δ 7.36–7.26 (Ph), 5.34 and 4.05 (2d, *J* = 15.0 Hz, 1H each, CH_2_Ph), 3.63 (br s, 1H, H-1), 2.59 (q, *J* = 7.6 Hz, 1H, H-4), 2.56 (br s, 1H, H-5), 2.46 (dd, *J* = 13.4, 7.2 Hz, 1H, H-7), 2.39–2.33 (m, 1H, H-7), 2.26 (dq, *J* = 13.6, 3.6 Hz, 1H, H-9), 2.23–2.16 (m, 1H, H-8), 1.93 (dm, *J* = 13.6 Hz, 1H, H-9), 1.77 (tdd, *J* = 13.6, 5.4, 2.6 Hz, 1H, H-8), 1.45 (d, *J* = 7.6 Hz, 3H, Me); ^13^C{^1^H} NMR (101 MHz, CDCl_3_) δ 210.8 (C-6), 172.2 (C-3), 137.3, 128.8, 127.8, and 127.3 (Ph), 51.2 (C-5), 50.3 (C-1), 48.3 (CH_2_Ph), 39.2 (C-4), 34.2 (C-7), 29.5 (C-8), 28.5 (C-9), 19.9 (Me); HRMS (ESI-TOF) *m*/*z* [M + H]^+^ calcd for C_16_H_20_NO_2_ 258.1489, found 258.1497.

#### (1*RS*,4*RS*,5*SR*)-2-Benzyl-4-methyl-2-azabicyclo[3.3.1]nonan-6-one (**8b**)

To a solution of AlCl_3_ (0.18 g, 1.35 mmol, 1.5 equiv) in THF (4 mL) was added dropwise a solution of LiAlH_4_ (1 M in THF, 1.8 mL, 2 equiv) at room temperature. After the mixture was stirred for 30 min, a solution of lactam **7** (0.27 g, 0.9 mmol, 1 equiv) was added dropwise via cannula. The reaction mixture was stirred overnight and cooled to 0 °C, and the reaction quenched with a 30% KOH aqueous solution. The mixture was extracted with CH_2_Cl_2_ (4 × 15 mL), and the combined organics were dried, filtered, and concentrated. The residue was diluted in a 10% HCl aqueous solution (5 mL), and the reaction mixture was stirred overnight at room temperature. Then, the reaction was quenched with 15% NaOH, and the mixture was extracted with CH_2_Cl_2_ (5 × 10 mL). The organics were dried, filtered, and concentrated. After chromatography (Al_2_O_3_, 98:2 hexane/EtOAc), aminoketone **8b** (0.18 g, 83% over two steps) was isolated as a colorless oil: IR (NaCl) 2926, 1704, 1425 cm^–1^; ^1^H NMR (400 MHz, CDCl_3_) δ 7.34–7.21 (m, 5H, Ph), 3.63 and 3.57 (2d, *J* = 13.6 Hz, 1H each, CH_2_Ph), 3.07 (br s, 1H, H-1), 2.58 (dd, *J* = 12.2, 5.6 Hz, 1H, H-3), 2.54 (dd, *J* = 8.8, 5.6 Hz, 1H, H-7), 2.49 (dd, *J* = 8.8, 5.6 Hz, 1H, H-7), 2.34–2.29 (m, 1H, H-9), 2.30 (dd, *J* = 12.2, 3.8 Hz, 1H, H-3), 2.24 (br d, *J* = 2.4 Hz, 1H, H-5), 2.17–2.10 (m, 1H, H-8), 2.08–2.05 (m, 1H, H-4), 1.78–1.68 (m, 1H, H-8), 1.16 (d, *J* = 6.8 Hz, 3H, Me); ^13^C{^1^H} NMR (101 MHz, CDCl_3_) δ 216.5 (C-6), 139.5, 128.4, 128.2, and 126.9 (Ph), 59.6 (CH_2_Ph), 51.4 (C-3), 51.0 (C-1), 48.8 (C-5), 37.4 (C-7), 33.2 (C-4), 25.9 (C-9), 22.2 (C-8), 18.6 (Me); HRMS (ESI-TOF) *m*/*z* [M + H]^+^ calcd for C_16_H_22_NO 244.1696, found 244.1703.

#### (1*RS*,4*RS*,5*SR*)-2-Methoxycarbonyl-2-azabicyclo[3.3.1]nonan-6-one (**8c**)

To a solution of **8b** (105 mg, 0.43 mmol, 1 equiv) in dry CHCl_3_ (1.5 mL) were added NaHCO_3_ (0.54 g, 6.47 mmol, 15 equiv) and methyl chloroformate (0.69 g, 0.57 mL, 7.3 mmol, 17 equiv). The reaction mixture was placed in a sealed tube and stirred at reflux overnight. The mixture was cooled to room temperature and diluted with CH_2_Cl_2_ (10 mL), and the organic layer was washed with water, dried, and concentrated. The crude residue was purified by chromatography (1:0 → 3:1 hexane/EtOAc), providing **8c** as a colorless oil (69 mg, 75%): IR (neat) 2956, 2873, 1699, 1447, 1230 cm^–1^; ^1^H NMR (400 MHz, CDCl_3_) δ 4.43–4.28 (2 s, 1H, H-1), 3.71 (s, 3H, OMe), 3.58–3.48 (m, 1H, H-3), 3.31 (dm, *J* = 13.6 Hz, 1H, H-3), 2.61 and 2.40 (2m, 1H each, H-7), 2.31 (s, 1H, H-5), 2.24 (m, 1H, H-9), 2.17 (m, 1H, H-8), 2.03 (s, 1H, H-4), 1.95 (m, 1H, H-8), 1.78 (m, 1H, H-9), 1.09 (s, 3H, Me); ^13^C{^1^H} NMR (101 MHz, CDCl_3_) δ 213.8 and 213.3 (C-6), 156.8 and 156.6 (CO_2_Me), 52.7 and 52.5 (OMe), 48.8 (C-5), 45.4 and 45.3 (C-1), 44.3 and 44.2 (C-3), 35.7 and 35.4 (C-7), 31.6 and 31.4 (C-4), 29.1 and 28.1 (C-8), 26.3 and 25.7 (C-9), 19.0 and 18.7 (Me); HRMS (ESI-TOF) *m*/*z* [M + H]^+^ calcd for C_11_H_18_NO_3_ 212.1281, found 212.1287.

### Synthesis of Homomorphans **1**

#### (1*RS*,6*SR*)-7-Benzyl-7-azabicyclo[4.3.1]decane-2,8-dione (**1a**)

To a solution of BF_3_·OEt_2_ (0.08 mL, 0.6 mmol, 1.3 equiv) in dry CH_2_Cl_2_ (5 mL) at 0 °C was added a solution of **5a** (0.105 g, 0.46 mmol) in CH_2_Cl_2_ (1.5 mL), followed by the addition of a TMSCHN_2_ solution (2 M in hexane, 0.3 mL, 1.3 equiv). The reaction mixture was warmed to rt and stirred overnight. The mixture was diluted in Et_2_O (5 mL), and the reaction quenched with an ice-cooled 2.5% NaHCO_3_ solution. The organic layer was washed with brine, dried, and concentrated to give a yellow crude residue, which was purified by chromatography (1:0 → 9:1 CH_2_Cl_2_/EtOAc). Homomorphan **1a** (45 mg, 38%) was isolated as a colorless oil: IR (NaCl) 2930, 1698, 1638, 1450 cm^–1^; ^1^H NMR (400 MHz, CDCl_3_) δ 7.33–7.23 (m, 5H, Ph), 5.32 and 3.97 (2 d, *J* = 15.0 Hz, 1H each, CH_2_Ph), 3.77–3.73 (m, 1H, H-6), 3.06 (d, *J* = 18.0 Hz, 1H, H-9), 2.76–2.72 (m, 1H, H-1), 2.70–2.63 (m, 2H, H-9 and H-3), 2.59–2.54 (m, 1H, H-3), 2.29–2.25 (m, 2H, H-5 and H-10), 2.21–2.13 (m, 1H, H-10), 1.87–1.74 (m, 1H, H-4), 1.55–1.38 (m, 2H, H-4 and H-5); ^13^C{^1^H} NMR (100 MHz, CDCl_3_) δ 213.3 (C-2), 169.2 (C-8), 137.2, 128.6, 128.0, and 127.4 (Ph), 53.1 (C-6), 47.0 (CH_2_Ph), 43.1 (C-3), 42.6 (C-1), 35.1 (C-9), 32.7 (C-5), 28.7 (C-10), 18.6 (C-4); HRMS (ESI-TOF) *m*/*z* [M + H]^+^ calcd for C_16_H_20_NO_2_ 258.1489, found 258.1494.

#### (1*RS*,6*RS*)-7-Benzyl-7-azabicylo[4.3.1]decan-2-one (**1b**)

BuLi (2.5 M in hexanes, 1.92 mL, 4.8 mmol, 2.25 equiv) was added to Et_2_O at −78 °C followed by the addition of TMSCHN_2_ (0.6 M in hexanes, 8 mL, 4.8 mmol, 2.25 equiv). After the mixture was stirred for 40 min, a solution of **5b** (0.49 g, 2.1 mmol) in THF (53 mL) was added slowly over 20 min with a syringe pump. The reaction mixture was stirred at −78 °C for 2 h; then, a solution of MeOH/THF (1:9, 20 mL) was added, and the mixture was stirred at room temperature for 30 min. A solution of 2 M NaOH was added; the phases were separated, and the aqueous layer was extracted sequentially with CH_2_Cl_2_ (2 × 50 mL), CHCl_3_ (3 × 50 mL), and a CHCl_3_/*i*-PrOH mixture (4:1, 2 × 25 mL). To the combined organics were added SiO_2_ and Na_2_SO_4_, and the mixture was stirred at room temperature for 40 min. The reaction mixture was filtered through a Celite pad and concentrated to yield a crude residue, which was purified by chromatography (Al_2_O_3_, 1:0 → 9.8:0.2 hexane/EtOAc), providing **1b** as a white solid (0.35 g, 67%): mp 78–81 °C; IR (NaCl) 2952, 2360, 2342, 1696, 1446 cm^–1^; ^1^H NMR (400 MHz, CDCl_3_) δ 3.83 and 3.25 (2d, *J* = 14.4 Hz, 1H each, CH_2_Ph), 2.94 (bt, 1H, H-6), 2.82 (td, *J* = 12, 4 Hz, 1H, H-3), 2.56–2.47 (m, 2H, H-3, H-8), 2.45 (ddt, *J* = 14.4, 7.6, 2 Hz, 1H, H-9), 2.32 (dtd, *J* = 7.6, 3.6, 1.6 Hz, H-1), 2.29–2.20 (m, 2H, H-5, H-8), 2.13 (ddd, *J* = 14.4, 3.6, 1.8 Hz, 1H, H-10), 2.06 (ddd, *J* = 14.4, 7.6, 3.6 Hz, 1H, H-10), 1.86–1.74 (m, 2H, H-4), 1.71–1.63 (m, 1H, H-9), 1.48 (dddd, *J* = 14.8, 12.4, 5.4, 2.8 Hz, 1H, H-5); ^13^C{^1^H} NMR (101 MHz, CDCl_3_) δ 215.5 (C-2), 139.5, 128.3, 128.0, and 126.7 (Ph), 57.7 (CH_2_Ph), 55.9 (C-6), 44.9 (C-8), 43.2 (C-3), 41.9 (C-1), 32.4 (C-5), 30.2 (C-10), 27.6 (C-9), 21.9 (C-4); HRMS (ESI-TOF) *m*/*z* [M + H]^+^ calcd for C_16_H_22_NO 244.1696, found 244.1701.

#### (1*RS*,6*RS*)-7-Methoxycarbonyl-7-azabicyclo[4.3.1]decan-2-one (**1c**)

Morphan **5c** (88 mg, 0.45 mmol, 1 equiv) was subjected to the ring expansion procedure being performed as described before for **1a** synthesis. After chromatography (1:0 → 9:1 CH_2_Cl_2_/EtOAc), homomorphan **1c** was obtained as an oil (28.9 mg, 31%) along with **1c′** (36.2 mg, 38%): IR (neat) 2952, 1697, 1447 cm^–1^; ^1^H NMR (400 MHz, CDCl_3_) δ 4.55 and 4.44 (2 s, 1H, H-6), 4.10 and 3.95 (2d, *J* = 11.6 Hz, 1H, H-8), 3.70 (s, 3H, OMe), 2.95–2.78 (m, 2H, H-8 and H-3), 2.62–2.58 (m, 1H, H-1), 2.54 (dd, *J* = 13.0, 6.6 Hz, 1H, H-3), 2.21–2.17 (m, 3H, H-10, H-9 and H-5), 2.03–1.89 (m, 2H, H-10 and H-4), 1.72–1.58 (m, 3H, H-9, H-5 and H-4); ^13^C{^1^H} NMR (101 MHz, CDCl_3_) δ 216.4 and 216.3 (C-2), 156.5 and 156.2 (CO_2_Me), 52.5 (OMe), 48.5 (C-6), 44.1 (C-3), 43.0 (C-1), 40.4 (C-8), 35.9 and 34.9 (C-5), 29.5 (C-10), 28.3 (C-9), 21.7 (C-4); HRMS (ESI-TOF) *m*/*z* [M + H]^+^ calcd for C_11_H_18_NO_3_ 212.1281, found 212.1289.

#### (1*RS*,6*RS*)-7-Methoxycarbonyl-7-azabicyclo[4.3.1]decan-3-one (**1c′**)

IR (neat) 2932, 1697, 1448 cm^–1^; ^1^H NMR (400 MHz, CDCl_3_) δ 4.59 and 4.46 (2d, *J* = 6.2 Hz, 1H, H-6), 3.86 (2 dt, *J* = 14.0, 4.8 Hz, 1H, H-8), 3.70 (s, 3H, OMe), 3.21 (t, *J* = 12.8 Hz, 1H, H-8), 2.65 (2 dd, *J* = 16.8, 5.2 Hz, 1H each, H-2), 2.58–2.42 (m, 2H, H-4), 2.28 (m, 1H, H-1), 2.02–1.96 (m, 3H, H-5 and H-10), 1.82–1.72 (m, 2H, H-9 and H-10), 1.65–1.59 (m, 1H, H-9); ^13^C{^1^H} NMR (101 MHz, CDCl_3_) δ 213.3 and 212.8 (C-3), 156.3 and 156.3 (CO_2_Me), 52.6 and 52.5 (OMe), 48.1 and 47.9 (C-6), 47.8 and 47.4 (C-2), 40.3 and 40.2 (C-4), 36.6 and 36.5 (C-8), 32.2 (C-10), 29.5 and 29.9 (C-9), 27.9 and 27.4 (C-5), 24.2 and 24.1 (C-1); HRMS (ESI-TOF) *m*/*z* [M + H]^+^ calcd for C_11_H_18_NO_3_ 212.1281, found 212.1287.

### Synthesis of Homomorphans **2**

#### (1*RS*,6*SR*,9*SR*)-7-Benzyl-9-methyl-7-azabicyclo[4.3.1]decan-2-one (**2b**)

Morphan **8b** (0.22 g, 0.91 mmol, 1 equiv) was subjected to ring expansion following the procedure for **1b** reported above. After chromatography (Al_2_O_3_, 1:0 → 9.8:0.2 hexane/EtOAc), homomorphan **2b** was obtained as waxy solid (0.154 g, 66% yield) along with **2b′** (31 mg, 13%): ^1^H NMR (400 MHz, CDCl_3_) δ 7.32–7.19 (m, 5H, Ph), 3.87 and 3.04 (2d, *J* = 14.4 Hz, 1H each, CH_2_Ph), 2.93–2.88 (m, 1H, H-9), 2.83–2.76 (m, 2H, H-6 and H-3), 2.57 (dd, *J* = 11.6, 8.4 Hz, 1H, H-8), 2.50 (ddd, *J* = 11.6, 6.4, 1.6 Hz, 1H, H-3), 2.23 (ddd, *J* = 14.4, 7.8, 4.0 Hz, 1H, H-5), 2.07 (dddd, *J* = 15.0, 8.4, 3.6, 0.8 Hz, 1H, H-10), 1.98 (dd, *J* = 15.0, 3.4 Hz, 1H, H-10), 1.91–1.79 (m, 2H, H-1 and H-4), 1.75–1.68 (m, 1H, H-4), 1.64 (dd, *J* = 11.6, 9.6 Hz, 1H, H-8), 1.50 (dddd, *J* = 14.4, 13.2, 4.8, 1.2 Hz, 1H, H-5), 0.88 (d, *J* = 6.8 Hz, 3H, Me); ^13^C{^1^H} NMR (101 MHz, CDCl_3_) δ 214.7 (C-2), 139.6, 128.3, 127.9, 126.6 (Ph), 57.1 (CH_2_Ph), 57.0 (C-6), 53.2 (C-8), 49.0 (C-1), 43.1 (C-3), 33.3 (C-5), 32.1 (C-9), 26.5 (C-10), 21.5 (C-4), 18.0 (Me); HRMS (ESI-TOF) *m*/*z* [M + H]^+^ calcd for C_17_H_24_NO 258.1852, found 258.1860.

#### (1*RS*,6*RS*,9*RS*)-7-Benzyl-9-methyl-7-azabicyclo[4.3.1]decan-3-one (**2b′**)

^1^H NMR (400 MHz, CDCl_3_) δ 7.32–7.21 (m, 5H, Ph), 3.68 and 3.40 (2d, *J* = 13.8 Hz, 1H each, CH_2_Ph), 3.05 (br q, *J* = 5.6 Hz, 1H, H-6), 2.68 (ddd, *J* = 15.6, 8.8, 6.8 Hz, 1H, H-4), 2.64–2.57 (m, 1H, H-8), 2.61 (d, *J* = 6.0 Hz, 2H, H-2), 2.38 (ddd, *J* = 15.6, 6.8, 6.0 Hz, 1H, H-4), 2.27–2.21 (m, 1H, H-10), 2.14–2.03 (m, 2H, H-8 and H-5), 1.82–1.77 (m, 1H, H-9), 1.76–1.71 (m, 1H, H-1), 1.69–1.60 (m, 1H, H-5), 1.47 (br d, *J* = 14.4 Hz, 1H, H-10), 0.95 (d, *J* = 6.8 Hz, 1H, Me); ^13^C{^1^H} NMR (101 MHz, CDCl_3_) δ 213.1 (C-3), 139.4, 128.4, 128.2, and 126.8 (Ph), 59.5 (CH_2_Ph), 54.4 (C-6), 50.6 (C-8), 50.4 (C-2), 40.0 (C-4), 33.6 (C-9), 32.3 (C-1), 29.9 (C- 10), 25.3 (C-5), 19.4 (Me); HRMS (ESI-TOF) *m*/*z* [M + H]^+^ calcd for C_17_H_24_NO 258.1852, found 258.1855.

#### (1*RS*,6*SR*,9*SR*)-7-Methoxycarbonyl-9-methyl-7-azabicyclo[4.3.1]decan-2-one (**2c**)

Morphan **8c** (50 mg, 0.24 mmol, 1 equiv) was subjected to the ring expansion procedure being performed as described above for the preparation of **1b**. After chromatography (1:0 → 9:1 CH_2_Cl_2_/EtOAc), homomorphan **2c** was obtained as a colorless oil (30 mg, 56%) along with **2c′** (7.5 mg, 14%): IR (neat) 2954, 2874, 1697, 1448 cm^–1^; ^1^H NMR (400 MHz, CDCl_3_) δ 4.48 and 4.43 (2 s, 1H, H-6), 3.70 (s, 3H, OMe), 3.59 (br s, 1H, H-8), 3.18 (dd, *J* = 14.0, 3.6 Hz, 1H, H-8), 2.80 (dd, *J* = 13.6, 4.8 Hz, 1H, H-3), 2.56 (dd, *J* = 13.6, 6.6 Hz, 1H, H-3), 2.39 (br s, 1H, H-9), 2.30 (br s, 1H, H-1), 2.24–2.19 (m, 2H, H-10 and H-5), 1.99 (d, *J* = 14.8 Hz, 1H, H-10), 1.90–1.85 (m, 1H, H-4), 1.78–1.61 (m, 2H, H-5 and H-4), 1.09 (d, *J* = 7.2 Hz, 3H, Me); ^13^C{^1^H} NMR (101 MHz, CDCl_3_) δ 216.1 (C-2), 156.9 (CO), 52.5 (OMe), 49.5 (C-1), 48.6 (C-6), 45.4 (C-8), 43.8 (C-3), 31.1 (C-9), 24.1 (C-10), 21.6 (C-4), 17.7 (Me) (the signal for C-5 was not observed); HRMS (ESI-TOF) *m*/*z* [M + H]^+^ calcd for C_12_H_20_NO_3_ 226.1438, found 226.1445.

### Ring Expansion from Azatricyclo **9**

#### (3*RS*,3a*SR*,7*SR*,8a*SR*,9*RS*)-3-Allyl-3a,9-dimethylhexahydro-2*H*-7,1-ethanocyclohepta[*b*]pyrrole-2,6(3*H*)-dione (**10a**)

Tricyclic compound **9** (34 mg, 0.14 mmol, 1 equiv) was subjected to the ring expansion procedure being performed as described before for the preparation of **1b**. After purification by chromatography (1:0 → 3:2 hexane/EtOAc), homologated compound **10a** was obtained as a colorless oil (11.7 mg, 33%) along with compound **10b** (5.4 mg, 15%): IR (NaCl) 2955, 1694, 1456, 1434, 912 cm^–1^; ^1^H NMR (400 MHz, CDCl_3_) δ 6.00–5.90 (m, 1H, =CH), 5.13 (d, *J* = 16.8 Hz, 1H, =CH_2_, H-*trans*), 5.04 (d, *J* = 10.0 Hz, 1H, =CH_2_, H-*cis*), 4.08 (dd, *J* = 13.6, 7.6 Hz, 1H, H-10), 3.63–3.61 (m, 1H, H-8a), 2.69–2.62 (m, 1H, CH_2_-3), 2.50–2.45 (m, 2H, H-5), 2.43–2.30 (m, 3H, H-10, H-3, and H-7), 2.16–2.03 (m, 4H, H-8, CH_2_-3, and H-9), 1.71 (ddd, *J* = 15.5, 8.8, 2.4 Hz, 1H, H-4), 1.48 (ddd, *J* = 15.5, 9.6, 4.0 Hz, 1H, H-4), 1.17 (s, 3H, Me-3a), 1.08 (d, *J* = 6.8 Hz, 3H, Me-9); ^13^C{^1^H} NMR (101 MHz, CDCl_3_) δ 212.7 (C-6), 174.3 (C-2), 137.1 (=CH_2_), 116.1 (=CH), 60.3 (C-8a), 52.5 (C-3), 50.6 (C-7), 45.2 (C-3a), 40.9 (C-10), 37.4 (C-5), 31.6 (C-9), 29.3 (CH_2_-3), 27.2 (C-4), 24.2 (Me-3a), 20.4 (Me-9), 19.2 (C-8); HRMS (ESI-TOF) *m*/*z* [M + H]^+^ calcd for C_16_H_24_NO_2_ 262.1802, found 262.1811.

#### (3*RS*,3a*SR*,7*RS*,8a*SR*,9*RS*)-3-Allyl-3a,9-dimethylhexahydro-2*H*-7,1-ethanocyclohepta[*b*]pyrrole-2,5(3*H*)-dione (**10b**)

IR (NaCl) 2957, 1699, 1459, 903 cm^–1^; ^1^H NMR (400 MHz, CDCl_3_) δ 5.98–5.88 (m, 1H, =CH), 5.17 (br d, *J* = 17 Hz, 1H, =CH_2_, H-*trans*), 5.02 (br d, *J* = 10 Hz, 1H, =CH_2_, H-*cis*), 4.00 (dd, *J* = 13.6, 8.0 Hz, 1H, H-10), 3.58 (d, *J* = 8.4 Hz, 1H, H-8a), 2.88–2.80 (m, 1H, CH_2_-3), 2.64 (dd, *J* = 12.0, 7.2 Hz, 1H, H-6), 2.55–2.50 (m, 1H, CH_2_-3), 2.48 and 2.40 (2d, *J* = 11.8 Hz, 1H each, H-4), 2.35–2.19 (m, 4H, H-6, H-10, H-3, and H-8), 2.02 (dd, *J* = 15.4, 2.2 Hz, H-8), 1.95–1.88 (m, 2H, H-7 and H-9), 1.18 (s, 3H, Me-3a), 0.96 (d, *J* = 6.8 Hz, Me-9); ^13^C{^1^H} NMR (101 MHz, CDCl_3_) δ 208.1 (C-5), 172.7 (C-2), 137.4 (=CH), 116.0 (=CH_2_), 61.1 (C-8a), 54.5 (C-6), 52.0 (C-3), 45.9 (C-4), 44.1 (C-3a), 41.1 (C-10), 33.5 (C-9), 32.9 (C-7), 30.3 (CH_2_-3), 25.7 (Me-3a), 22.0 (C-8), 20.0 (Me-9).

#### (3*RS*,3a*SR*,7a*SR*)-3-Allyl-1-benzyl-3a-methyloctahydro-5*H*-indol-5-one (**12**)

To a cooled (0 °C) suspension of AlCl_3_ (0.29 g, 2.18 mmol, 1.5 equiv) in THF (7 mL) was added a solution of LiAlH_4_ (1 M in THF, 2.9 mL, 2 equiv). After being stirred at this temperature for 20 min, a solution of compound **11**(22) (0.5 g, 1.45 mmol, 1 equiv) in THF (14 mL) was added via cannula, and the reaction mixture was stirred at room temperature overnight. Next, the reaction was quenched with a 30% KOH solution (20 mL), and the mixture extracted with a CHCl_3_/*i*-PrOH mixture (4:1, 4 × 20 mL). The combined organics were dried, filtered, and concentrated to afford a crude residue, which was diluted in 10% HCl (30 mL) and stirred overnight. After basification with a 15% NaOH solution (40 mL), the mixture was extracted with CH_2_Cl_2_ (4 × 30 mL) and the combined organics were dried, filtered, concentrated, and purified by chromatography (9.5:0.5 → 4:1 hexane/EtOAc) to provide compound **12** (0.26 g, 64%) as a waxy solid: IR (NaCl) 3027, 2954, 1712, 1452, 912, 739, 699 cm^–1^; ^1^H NMR (400 MHz, CDCl_3_) δ 5.66 (ddt, *J* = 17.0, 10.2, 6.8 Hz, 1H, =CH), 4.97 (dq, *J* = 17.0 Hz, 1H, =CH_2_, H-*trans*), 4.91 (ddt, *J* = 10.2, 2.1, 1.1 Hz, 1H, =CH_2_, H-*cis*), 4.08 and 3.34 (2d, *J* = 13.6 Hz, 1H each, CH_2_Ph), 2.80 (dd, *J* = 19.8, 13.8 Hz, 1H, H-6), 2.80 (dd, *J* = 10.4, 8.2 Hz, 1H, H-2), 2.63 (dd, *J* = 10.4, 9.6 Hz, 1H, H-2), 2.62 (d, *J* = 13.0 Hz, 1H, H-4), 2.53 (t, *J* = 2.6 Hz, 1H, H-7a), 2.20–2.05 (m, 3H, CH_2_-3, H-7, and H-6), 1.88 (d, *J* = 13.0 Hz, 1H, H-4), 1.92–1.75 (m, 3H, H-3, H-7, and CH_2_-3), 1.02 (s, 3H, Me); ^13^C{^1^H} NMR (101 MHz, CDCl_3_) δ 212.9 (C-5), 139.9 (Ph), 137.1 (=CH), 128.3, 128.1, and 126.8 (Ph), 115.6 (=CH_2_), 68.3 (C-7a), 58.0 (CH_2_Ph), 56.8 (C-2), 47.8 (C-3a), 47.1 (C-3), 45.3 (C-4), 35.8 (C-6), 32.8 (C-7), 24.2 (CH_2_-3), 22.9 (Me); HRMS (ESI-TOF) *m*/*z* [M + H]^+^ calcd for C_19_H_26_NO 284.2009, found 284.2013.

### Ring Expansion from Azatricyclo **13**

#### (3*RS*,3a*SR*,8a*SR*)-1-Benzyl-3a-methyl-3-(prop-2-en-1-yl)hexahydrocyclohepta[*b*]pyrrole-2,5(1*H*,3*H*)-dione (**14**)

Lactam **13**(23) (4.91 g, 16.5 mmol) was subjected to the ring expansion procedure being performed as described above for the preparation of **1b**. After chromatography (9:1 → 4:1 hexane/EtOAc), ring-expanded product **14** (3.81 g, 74%) was obtained as a waxy solid along with subproduct **15** (0.40 g, 8%) and silyl enol ether **16** (0.51 g, 8%). The NMR data for **14** matched those previously reported by our research group.^23^ Epoxide **15**: IR (NaCl) 3065, 2938, 1690, 1432, 1411, 914, 732 cm^–1^; ^1^H NMR (400 MHz, CDCl_3_) δ 7.26–7.13 (m, 5H, Ph), 5.95–5.85 (m, 1H, =CH), 5.05–4.91 (m, 3H, =CH_2_ and CH_2_Ph), 3.93 (d, *J* = 15.0 Hz, 1H, CH_2_Ph), 3.11 (t, *J* = 3.1 Hz, 1H, H-7a), 2.60–2.54 (m, 1H, CH_2_-3), 2.47 (s, 2H, OCH_2_), 2.10 (dd, *J* = 8.6, 5.2 Hz, 1H, H-3), 2.08–1.99 (m, 1H, CH_2_-3), 1.94–1.80 (m, 3H, H-7 and H-4), 1.57 (td, *J* = 13.5, 5.2 Hz, 1H, H-6), 1.19 (s, 3H, Me), 0.93–0.87 (m, 2H, H-6 and H-4); ^13^C{^1^H} NMR (101 MHz, CDCl_3_) δ 177.0 (C-2), 137.3 (=CH), 136.8, 128.6, 127.9, and 127.4 (Ph), 116.0 (=CH_2_), 59.7 (C-7a), 56.1 (C-5), 54.6 (C-3), 52.0 (OCH_2_), 44.1 (CH_2_Ph), 41.2 (C-3a), 34.8 (C-4), 28.9 (CH_2_-3), 26.6 (C-6), 22.8 (Me), 19.9 (C-7); HRMS (ESI-TOF) *m*/*z* [M + H]^+^ calcd for C_20_H_26_NO_2_ 312.1958, found 312.1967

#### Silyl Enol Ether **16**

IR (NaCl) 3066, 2957, 1690, 1433, 1251, 845, 733 cm^–1^; ^1^H NMR (400 MHz, CDCl_3_) δ 7.33–7.18 (m, 5H, Ph), 5.99 (dddd, *J* = 17.0, 9.8, 8.0, 5.6 Hz, 1H, =CH), 5.15 (dd, *J* = 17.0 Hz, 1H, =CH_2_, H-*trans*), 5.06 (d, *J* = 9.8 Hz, 1H, =CH_2_, H-*cis*), 5.05 (d, *J* = 15.2 Hz, 1H, CH_2_Ph), 4.88 (br t, *J* = 4.5 Hz, 1H, H-6), 3.94 (d, *J* = 15.2 Hz, 1H, CH_2_Ph), 3.07 (dd, *J* = 9.2, 4.2 Hz, 1H, H-8a), 2.66 (d, *J* = 15.8 Hz, 1H, H-4), 2.65–2.58 (m, 1H, CH_2_-3), 2.29 (dd, *J* = 8.2, 5.8, 1H, H-3), 2.20 (dt, *J* = 14.0, 8.0 Hz, 1H, CH_2_-3), 2.06 (dt, *J* = 15.8, 7.0 Hz, 1H, H-7), 1.95–1.78 (m, 2H, H-7 and H-8), 1.78 (d, *J* = 15.8 Hz, 1H, H-4), 1.69–1.61 (m, 1H, H-8), 1.19 (s, 3H, Me), 0.16 (s, 9H, TMS); ^13^C{^1^H} NMR (101 MHz, CDCl_3_) δ 175.6 (C-2), 150.1 (C-5), 137.3 (=CH), 136.8, 128.6, 127.8, and 127.4 (Ph), 116.1 (=CH_2_), 107.1 (C-6), 64.7 (C-8a), 53.5 (C-3), 43.8 (NCH_2_), 42.1 (C-3a), 35.9 (C-4), 30.6 (CH_2_-3), 27.5 (C-8), 25.1 (Me), 20.9 (C-7), 0.3 (TMS).

### Synthesis of the ABC Ring System

#### (3*RS*,3a*SR*,8a*SR*)-1-Benzyl-3a-methyl-3-(prop-2-en-1-yl)hexahydrocyclohepta[*b*]pyrrole-2,5(1*H*,3*H*)-dione Ethylene Acetal (**17**)

To a solution of bicyclic lactam **14** (3.81 g, 12.2 mmol, 1 equiv) in toluene (250 mL) were added ethylene glycol (34.2 mL) and *p*-TsOH (0.93 g, 4.89 mmol, 0.4 equiv). The reaction mixture was heated to reflux with a Dean-Stark apparatus over 5 h. Then, after the mixture had cooled, a saturated NaHCO_3_ solution (300 mL) was added and the mixture was extracted with EtOAc (3 × 150 mL). The combined organics were dried, filtered, concentrated, and purified by chromatography (9:1 → 4:1 hexane/EtOAc) to afford protected compound **17** (3.64 g, 84%) as a waxy solid: IR 2933, 1682, 1434, 1036, 700 cm^–1^; ^1^H NMR (400 MHz, CDCl_3_) δ 7.34–7.20 (m, 5H, Ph), 6.00–5.90 (m, 1H, =CH), 5.13–5.01 (m, 3H, =CH_2_ and CH_2_Ph), 3.92–3.83 (m, 5H, OCH_2_ and CH_2_Ph), 2.98 (dd, *J* = 11.0, 2.0 Hz, 1H, H-8a), 2.49–2.40 (m, 2H, 3-CH_2_), 2.15–2.10 (m, 1H, H-3), 2.12 (d, *J* = 13.8 Hz, 1H, H-4), 1.92–1.88 (m, 1H, H-8), 1.76–1.67 (m, 2H, H-7 and H-6), 1.62 (d, *J* = 13.8 Hz, 1H, H-4), 1.64–1.56 (m, 1H, H-6), 1.55–1.46 (m, 1H, H-8), 1.40–1.32 (m, 1H, H-7), 1.12 (s, 3H, Me); ^13^C{^1^H} NMR (101 MHz, CDCl_3_) δ 175.3 (CO), 137.1 (=CH), 136.7, 128.6, 128.3, and 127.5 (Ph), 116.0 (=CH_2_), 111.2 (C-5), 67.1 (C-8a), 64.8 and 63.5 (OCH_2_), 55.1 (C-3), 44.3 (CH_2_Ph), 41.0 (C-4), 39.5 (C-3a), 39.0 (C-6), 33.9 (3-CH_2_), 30.3 (Me), 28.1 (C-8), 20.6 (C-7); HRMS (ESI-TOF) *m*/*z* [M + H]^+^ calcd for C_22_H_30_NO_3_ 356.2220, found 356.2228.

#### (3*RS*,3a*SR*,8a*SR*)-1-Benzyl-3a-methyl-3-(3′-hydroxypropyl)hexahydrocyclohepta[*b*]pyrrole-2,5(1*H*,3*H*)-dione Ethylene Acetal (**18**)

A solution of **17** (3.61 g, 10.2 mmol) and a 9-BBN solution (0.5 M in THF, 40.6 mL, 20.3 mmol) was stirred for 5 h at room temperature. Then, at 0 °C, 2 M NaOH (40.5 mL) and H_2_O_2_ (46 mL) were added, and the reaction mixture was stirred at room temperature overnight. Water was added (150 mL), and the mixture was extracted with EtOAc (4 × 100 mL). The combined organics were dried, filtered, concentrated, and purified by chromatography (9.9:0.1 → 9.5:0.5 CH_2_Cl_2_/MeOH) to give alcohol **18** (3.56 g, 94%) as a colorless waxy solid: IR (NaCl) 3406, 2869, 1673, 1434, 700 cm^–1^; ^1^H NMR (400 MHz, CDCl_3_) δ 7.34–7.20 (m, 5H, Ph), 5.01 (d, *J* = 14.8 Hz, 1H, CH_2_Ph), 3.92–3.83 (m, 5H, CH_2_Ph and OCH_2_), 3.77–3.72 and 3.69–3.63 (2m, 1H each, CH_2_OH), 2.98 (dd, *J* = 10.8, 2.4 Hz, 1H, H-8a), 2.12–2.08 (m, 2H, H-4 and H-3), 1.94–1.57 (m, 9H, H-4, H-6, 1′-CH_2_-3, H-7, 2′-CH_2_-3, H-8), 1.55–1.45 (m, 1H, H-8), 1.40–1.33 (m, 1H, H-7), 1.10 (s, 3H, Me); ^13^C{^1^H} NMR (101 MHz, CDCl_3_) δ 176.4 (C-2), 136.6, 128.6, 128.3, 127.5 (Ph), 111.2 (C-5), 67.1 (C-8a), 64.9 and 63.5 (OCH_2_), 62.6 (CH_2_OH), 54.4 (C-3), 44.3 (CH_2_Ph), 40.5 (C-4), 39.7 (C-3a), 39.0 (C-6), 31.2 (2′-CH_2_-3), 29.8 (Me), 28.2 (C-8), 26.5 (1′-CH_2_-3), 20.8 (C-7); HRMS (ESI-TOF) *m*/*z* [M + H]^+^ calcd for C_22_H_32_NO_4_ 374.2326, found 374.2329.

#### (3*RS*,3a*SR*,8a*SR*)-1-Benzyl-3a-methyl-3-(3′-methoxypropyl)hexahydrocyclohepta[*b*]pyrrole-2,5(1*H*,3*H*)-dione Ethylene Acetal (**19**)

To a solution of **18** (3.63 g, 9.72 mmol) in THF (150 mL) at 0 °C was added portionwise NaH (60% in mineral oil, 1.17 g, 29.2 mmol, 3 equiv). After the mixture had been stirred at this temperature for 30 min, iodomethane (1.8 mL, 29.2 mmol, 3 equiv) was added. The reaction mixture was stirred at room temperature overnight, the reaction quenched with NH_4_Cl, and the mixture extracted with CH_2_Cl_2_ (4 × 100 mL). The combined organics were dried, filtered, concentrated, and purified by chromatography (9.9:0.1 → 9.5:0.5 CH_2_Cl_2_/MeOH) to provide protected alcohol **19** (3.3 g, 87%) as a yellowish waxy solid: IR (NaCl) 2933, 1680, 1448, 1118, 1074, 731, 702 cm^–1^; ^1^H NMR (400 MHz, CDCl_3_) δ 7.33–7.20 (m, 5H, Ph), 5.00 (d, *J* = 14.8 Hz, 1H, CH_2_Ph), 3.90–3.83 (m, 4H, OCH_2_ and CH_2_Ph), 3.47–3.35 (m, 2H, CH_2_OMe), 3.33 (s, 3H, OMe), 2.95 (dd, *J* = 11.0, 2.2 Hz, 1H, H-8a), 2.11 (d, *J* = 14.6, 1H, H-4), 1.94 (dd, *J* = 9.7, 5.4 Hz, 1H, H-3), 2.05–1.88 (m, 2H, 2′-CH_2_-3 and H-8), 1.80–1.66 (m, 4H, 2′-CH_2_-3, 1′-CH_2_-3, H-6, and H-7), 1.64–1.59 (m, 1H, H-6), 1.59 (d, *J* = 14.6 Hz, 1H, H-4), 1.55–1.43 (m, 2H, 1′-CH_2_-3 and H-8), 1.40–1.32 (m, 1H, H-7), 1.09 (s, 3H, Me); ^13^C{^1^H} NMR (101 MHz, CDCl_3_) δ 175.9 (C-2), 136.8, 128.6, 128.3, and 127.4 (Ph), 111.3 (C-5), 72.6 (CH_2_OMe), 67.0 (C-8a), 64.9 and 63.4 (OCH_2_), 58.5 (OMe), 54.9 (C-3), 44.2 (CH_2_Ph), 40.7 (C-4), 39.4 (C-3a), 38.9 (C.6), 30.0 (Me), 28.5 (2′-CH_2_-3), 28.1 (C-8), 26.7 (1′-CH_2_-3), 20.9 (C-7); HRMS (ESI-TOF) *m*/*z* [M + H]^+^ calcd for C_23_H_34_NO_4_ 388.2482, found 388.2475.

#### (3*RS*,3a*SR*,8a*SR*)-3-(3-Methoxypropyl)-3a-methyloctahydrocyclohepta[*b*]pyrrol-5(1*H*)-one Ethylene Acetal (**21**)

A suspension of LiAlH_4_ (0.84 g, 22.1 mmol, 4 equiv) in THF (22 mL) was cooled to 0 °C, and then a solution of lactam **19** (2.15 g, 5.54 mmol) in THF (27 mL) was added dropwise. Then, the reaction mixture was heated to reflux and stirred for 4 h before the reaction was cautiously quenched with 15% NaOH. Water was added (20 mL), and the aqueous layer was extracted with CH_2_Cl_2_ (5 × 20 mL). The organic phase was dried, filtered, and concentrated under vacuum to afford a crude residue, which was purified by a chromatographic column (4:1 → 1:1 hexane/EtOAc), providing amine **20** (1.35 g, 66%) as a waxy solid: IR (NaCl) 2927, 1451, 1008, 701 cm^–1^. To a solution of amine **20** (1.35 g, 3.61 mmol) in MeOH (42 mL) was added Pd/C (0.68 g, 50% wt.), and the reaction mixture was stirred under a H_2_ atmosphere at rt overnight. Then, it was filtered through a Celite pad and concentrated to afford compound **21** (1.02 g, quantitative yield), which was used directly in the next step: IR (NaCl) 3425, 3421, 2929, 1694, 1455, 1112 cm^–1^; ^1^H NMR (400 MHz, CDCl_3_) δ 3.39–3.85 (m, 3H, OCH_2_), 3.80–3.75 (m, 1H, OCH_2_), 3.36 (t, *J* = 6 Hz, 2H, CH_2_OMe), 3.33 (s, 3H, OMe), 3.20 (dd, *J* = 10.8, 8.4 Hz, 1H, H-2), 3.00 (t, *J* = 4.4 Hz, 1H, H-8a), 2.65 (dd, *J* = 10.8, 9.8 Hz, 1H, H-2), 1.93 (d, *J* = 15.0 Hz, 1H, H-4), 1.95–1.85 (m, 2H, H-6 and H-8), 1.78–1.64 (m, 4H, H-3, H-6, H-8, and H-7), 1.58–1.48 (m, 4H, 3-CH_2_-2′, H-7, and 3-CH_2_-1′), 1.45 (d, *J* = 15.0 Hz, 1H, H-4), 1.24–1.16 (m, 1H, 3-CH_2_-1′), 1.14 (s, 3H, Me); ^13^C{^1^H} NMR (101 MHz, CDCl_3_) δ 112.6 (C-5), 72.8 (CH_2_OMe), 67.7 (C-8a), 64.6 and 63.3 (OCH_2_), 58.5 (OMe), 53.1 (C-3), 48.7 (C-2), 43.4 (C-3a), 39.5 (C-6), 37.3 (C-4), 31.2 (C-8), 28.9 (3-CH_2_-2′), 25.6 (3-CH_2_-1′), 23.9 (Me), 18.4 (C-7).

#### (3*RS*,3a*SR*,8a*SR*)-3-(3-Methoxypropyl)-3a-methyl-1-(2,2,2-trichloroacetyl)octahydrocyclohepta[*b*]pyrrol-5(1*H*)-one Ethylene Acetal (**22**)

A solution of amine **21** (1.02 g, 3.53 mmol) in CH_2_Cl_2_ (11 mL) was cooled to 0 °C, and pyridine (0.6 mL, 7.41 mmol, 2.1 equiv) and trichloroacetyl chloride (0.6 mL, 5.29 mmol, 1.5 equiv) were added. The reaction mixture was stirred overnight; then water basified with 15% NaOH was added, and the aqueous layer was extracted with CH_2_Cl_2_ (4 × 20 mL). The combined organics were dried, filtered, and concentrated to afford a crude residue, which was purified by chromatography (9:1 hexane/EtOAc), providing trichloroacetamide **22** (1.44 g, 93%): IR (NaCl) 2933, 1698, 1376, 1113, 700 cm^–1^; ^1^H NMR (400 MHz, CDCl_3_) δ 4.28 (dd, *J* = 11.6, 7.2 Hz, 1H, H-2), 3.99 (dd, *J* = 10, 2.4 Hz, 1H, H-8a), 3.96–3.87 (m, 4H, OCH_2_), 3.52 (dd, *J* = 11.6, 8 Hz, 1H, H-2), 3.39 (t, *J* = 6 Hz, 2H, CH_2_OMe), 3.33 (s, 3H, OMe), 2.10–2.04 (m, 2H, H-4 and H-8), 1.86–1.75 (m, 2H, H-3 and H-6), 1.71–1.51 (m, 7H, H-4, H-6, 3-CH_2_-2′, H-8, 3-CH_2_-1′, and H-7), 1.53–1.44 (m, 1H, 3-CH_2_-2′), 1.35–1.29 (m, 1H, 3-CH_2_-1′), 1.26 (s, 3H, Me); ^13^C{^1^H} NMR (101 MHz, CDCl_3_) δ 156.3 (CO), 111.5 (C-5), 93.9 (CCl_3_), 72.5 (CH_2_OMe), 71.0 (C-8a), 64.8 and 63.6 (OCH_2_), 58.7 (OMe), 52.5 (C-2), 51.6 (C-3), 42.5 (C-3a), 40.9 (C-4), 38.8 (C-6), 30.2 (Me), 29.3 (3-CH_2_-2′), 28.1 (C-8), 26.1 (3-CH_2_-1′), 20.0 (C-7); HRMS (ESI-TOF) *m*/*z* [M + H]^+^ calcd for C_18_H_29_Cl_3_NO_4_ 428.1157, found 428.1164.

#### (3*RS*,3a*SR*,8a*SR*)-3-(3-Methoxypropyl)-3a-methyl-1-(2,2,2-trichloroacetyl)octahydrocyclohepta[*b*]pyrrol-5(1*H*)-one (**23**)

Chloroacetamide **22** (1.16 g, 2.71 mmol) was diluted in THF (20 mL), and a 10% HCl solution (40 mL) was added. After the mixture had been stirred at room temperature overnight, water (20 mL) was added, and the mixture was extracted with CH_2_Cl_2_ (4 × 25 mL). The combined organics were dried, filtered, and concentrated, and the obtained crude residue was purified by chromatography (9:1 → 4:1 hexane/EtOAc) to yield chloroacetamide **23** (0.94 g, 90%) as a colorless oil: IR (NaCl) 2934, 2870, 1703, 1679, 1453, 1389, 1116, 811, 699 cm^–1^; ^1^H NMR (400 MHz, CDCl_3_) δ 4.37 (dd, *J* = 11.2, 6.8 Hz, 1H, H-2), 3.90 (dd, *J* = 7.0, 6.2 Hz, 1H, H-8a), 3.41–3.33 (m, 3H, H-2 and CH_2_OMe), 3.34 (s, 3H, OMe), 2.73 (d, *J* = 11.4 Hz, 1H, H-4), 2.66–2.58 (m, 1H, H-8), 2.40 (bt, *J* = 6.0 Hz, 2H, H-6), 2.14 (d, *J* = 11.4 Hz, 1H, H-4), 1.87–1.52 (m, 7H, H-3, H-8, 2′-CH_2_-3, H-7, 1′-CH_2_-3), 1.36–1.27 (m, 1H, 1′-CH_2_-3), 1.13 (s, 3H, Me); ^13^C{^1^H} NMR (101 MHz, CDCl_3_) δ 211.9 (C-5), 159.7 (CO), 93.7 (C), 72.4 (CH_2_OMe), 71.1 (C-8a), 58.7 (OMe), 53.5 (C-2), 50.1 (C-3), 45.3 (C-6), 44.4 (C-4), 43.6 (C-3a), 28.9 (2′-CH_2_-3), 25.7 (C-8), 25.1 (Me), 23.7 (1′-CH_2_-3), 16.9 (C-7); HRMS (ESI-TOF) *m*/*z* [M + H]^+^ calcd for C_16_H_25_Cl_3_NO_3_ 384.0895, found 384.0904.

#### (3*RS,*3a*SR*,8a*SR*)-3-(3-Methoxypropyl)-3a-methyl-1-(2,2,2-trichloroacetyl)-2,3,3a,4,8,8a-hexahydrocyclohepta[*b*]pyrrol-5(1*H*)-one (**24**)

A solution of trichloroacetamide **23** (0.36 g, 0.93 mmol), IBX (0.65 g, 2.33 mmol, 2.5 equiv), and *p*-TsOH (53 mg, 0.28 mmol, 0.4 equiv) in DMSO (9 mL) was heated at 70 °C overnight. Upon cooling, the mixture was portioned between EtOAc (20 mL) and water (20 mL), and the aqueous layer was extracted with EtOAc (5 × 10 mL). The combined organic layers were dried, filtered, and concentrated to afford the crude product, which was purified by flash column chromatography (1:0 → 9.5:0.5 CH_2_Cl_2_/MeOH), and enone **24** (0.21 g, 60%) was obtained along with traces of overoxidized product **24b**: IR (NaCl) 2931, 1700, 1075, 1115 cm^–1^; ^1^H NMR (400 MHz, CDCl_3_) δ 6.64 (ddd, *J* = 11.2, 8.2, 4.6 Hz, 1H, H-7), 6.14 (dd, *J* = 11.2, 2.0 Hz, 1H, H-6), 4.31 (dd, *J* = 11.6, 7.2 Hz, 1H, H-2), 4.12 (d, *J* = 7.2 Hz, 1H, H-8a), 3.43–3.32 (m, 3H, CH_2_OMe and H-8), 3.34 (s, 3H, OMe), 3.09 (t, *J* = 11.6 Hz, 1H, H-2), 2.74 (dddd, *J* = 16.4, 4.6, 2.2, 2.0 Hz, 1H, H-8), 2.68 and 2.53 (2d, *J* = 13.2 Hz, 1H each, H-4), 1.97–1.89 (m, 1H, H-3), 1.71–1.48 (m, 4H, 1′-CH_2_-3 and 2′-CH_2_-3), 1.22 (s, 3H, Me); ^13^C{^1^H} NMR (101 MHz, CDCl_3_) δ 200.7 (C-5), 158.6 (CO), 143.9 (C-7), 134.9 (C-6), 93.3 (C), 72.4 (CH_2_OMe), 70.5 (C-8a), 58.7 (OMe), 54.2 (C-2), 49.5 (C-4), 49.1 (C-3), 44.8 (C-3a), 29.0 (2′-CH_2_-3), 27.7 (C-8), 27.1 (Me), 23.9 (1′-CH_2_-3); HRMS (ESI-TOF) *m*/*z* [M + H]^+^ calcd for C_16_H_23_Cl_3_NO_3_ 382.0738, found 382.0739.

#### Overoxidized Compound **24b**

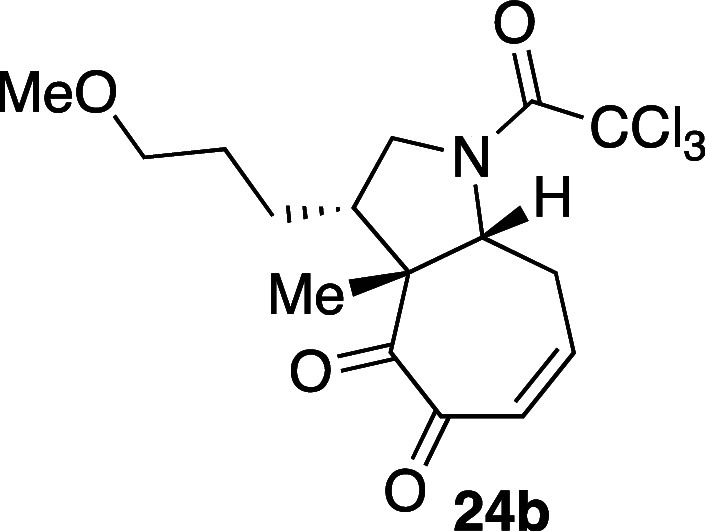



IR (NaCl) 2927, 2868, 1702, 1677, 1459, 1390, 1116, 843 cm^–1^; ^1^H NMR (400 MHz, CDCl_3_) δ 6.87 (ddd, *J* = 11.2, 9.0, 3.0 Hz, 1H, H-7), 6.30 (dd, *J* = 11.2, 3.2 Hz, 1H, H-6), 4.47 (dd, *J* = 11.8, 8.0 Hz, 1H, H-2), 4.18 (d, *J* = 6.8 Hz, 1H, H-8a), 3.62 (ddd, *J* = 17.2, 9.2, 7.2 Hz, 1H, H-8), 3.45 (t, *J* = 11.8 Hz, 1H, H-2), 3.39–3.33 (m, 2H, CH_2_OMe), 3.32 (s, 3H, OMe), 2.46 (dt, *J* = 17.2, 2.8 Hz, 1H, H-8), 2.24–2.15 (m, 1H, H-3), 1.69–1.51 (m, 3H, 2′-CH_2_-3 and 1′-CH_2_-3), 1.46–1.42 (m, 1H, 3-CH_2_-1′), 1.39 (s, 3H, Me); ^13^C{^1^H} NMR (101 MHz, CDCl_3_) δ 206.6 (C-4), 196.6 (C-5), 159.1 (CO), 146.9 (C-7), 130.3 (C-6), 92.9 (C), 72.1 (CH_2_OMe), 67.7 (C-8a), 58.7 (OMe), 58.6 (C-3a), 54.5 (C-2), 47.7 (C-3), 28.8 (2′-CH_2_-3), 28.2 (C-8), 24.8 (1′-CH_2_-3), 18.8 (Me).

#### (3*RS*,3a*SR*,7*RS*,8a*SR*)-3-(3-Methoxypropyl)-3a-methyloctahydro-5*H*-1,7-ethanocyclohepta[*b*]pyrrole-5,10-dione (**25**)

A solution of trichloroacetamide **24** (0.21 g, 0.56 mmol) in benzene (19 mL) was heated to reflux. Then, a solution of AIBN (46 mg, 0.28 mmol, 0.5 equiv) and Bu_3_SnH (0.6 mL, 2.24 mmol, 4 equiv) in benzene (4 mL) was added over 4 h via a syringe pump. The reaction mixture was stirred for an additional 1 h, cooled, and concentrated. The residue was purified by chromatography (1:0 → 9.9:0.1 CH_2_Cl_2_/MeOH) to obtain tricyclic compound **25** (110 mg, 72%): IR (NaCl) 2926, 2870, 1695, 1645, 1455, 1117 cm^–1^; ^1^H NMR (400 MHz, CDCl_3_) δ 3.45–3.37 (m, 4H, 3-CH_2_-3′, H-2, and H-8a), 3.33 (s, 3H, OMe), 3.12 (dd, *J* = 12.0, 10.0 Hz, 1H, H-2), 2.64 (dd, *J* = 13.0, 8.2 Hz, 1H, H-6), 2.50 (m, 1H, H-7), 2.44–2.31 (m, 4H, H-6, H-4, and H-9), 2.25–2.21 (m, 3H, H-4 and H-8), 1.84 (ddd, *J* = 20.0, 10.0, 1.8 Hz, 1H, H-3), 1.67–1.62 (m, 2H, 2′-CH_2_-3 and 1′-CH_2_-3), 1.56–1.49 (m, 2H, 2′-CH_2_-3 and 1′-CH_2_-3), 1.06 (s, 3H, Me); ^13^C{^1^H} NMR (101 MHz, CDCl_3_) δ 207.9 (C-5), 168.2 (C-10), 72.6 (3-CH_2_-3), 64.8 (C-8a), 58.6 (OMe), 50.2 (C-6), 48.2 (C-2), 46.8 (C-3a), 46.7 (C-3), 45.1 (C-4), 39.7 (C-9), 28.6 (2′-CH_2_-3), 26.1 (1′-CH_2_-3), 25.9 (C-7), 25.3 (C-8), 24.3 (Me); HRMS (ESI-TOF) *m*/*z* [M + H]^+^ calcd for C_16_H_26_NO_3_ 280.1907, found 280.1914.

## Data Availability

The data underlying this study are available in the published article and its Supporting Information.

## Supporting Information Available

Copies of ^1^H and ^13^C NMR spectra of all reported compounds (PDF)
FAIR data, including the primary NMR FID files, for compounds **1–25** (ZIP)
