# Supplementary material for: Synthesis of the ABC Core of Daphniphyllum Alkaloids with a [5–6–7] Azatricyclic Scaffold via Ring Expansion of Azabicyclic and Azatricyclic Building Blocks
Source: J Org Chem. 2024 Jul 1;89(14):10212–22. doi: 10.1021/acs.joc.4c01090 (PMC11267610; doi:10.1021/acs.joc.4c01090)
Supplement: Supplementary file 1 — jo4c01090_si_001.pdf [file jo4c01090_si_001.pdf]

Supporting Information

Synthesis of the ABC Core of *Daphniphyllum* Alkaloids with a [5-6-7] Azatricyclic Scaffold via the Ring Expansion of Azabicyclic and Azatricyclic Building Blocks

Clàudia Marquès, David González-Lizana, Faïza Diaba,* and Josep Bonjoch*

Laboratori de Química Orgànica, Facultat de Farmàcia, Universitat de Barcelona, Av. Joan XXIII 27-31, 08028-Barcelona, Spain

e-mail address: josep.bonjoch@ub.edu; faiza.diaba@ub.edu

Contents

- NMR spectra of homomorphans **1a-2c'** **S2-S16**
- NMR spectra of morphans **5b-8c** **S17-S29**
- NMR spectra of homomorphans **10a** and **10b** **S30-S33**
- NMR spectra of compounds **12-25** **S34-S59**

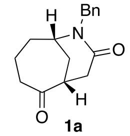

1a

1a/1H
Equip: B400F / N.Inv: 1037597
N.Reg: 22060575
Usuari: san / Mostra: XDG046-36
Nom: CLAUDIA MARQUES GARCIA
Data: 14/06/2022 18:01:21 h./ Ope.: AUTOSERVEI
Experiment: A-H1-zg30 Solvent: CDCl3

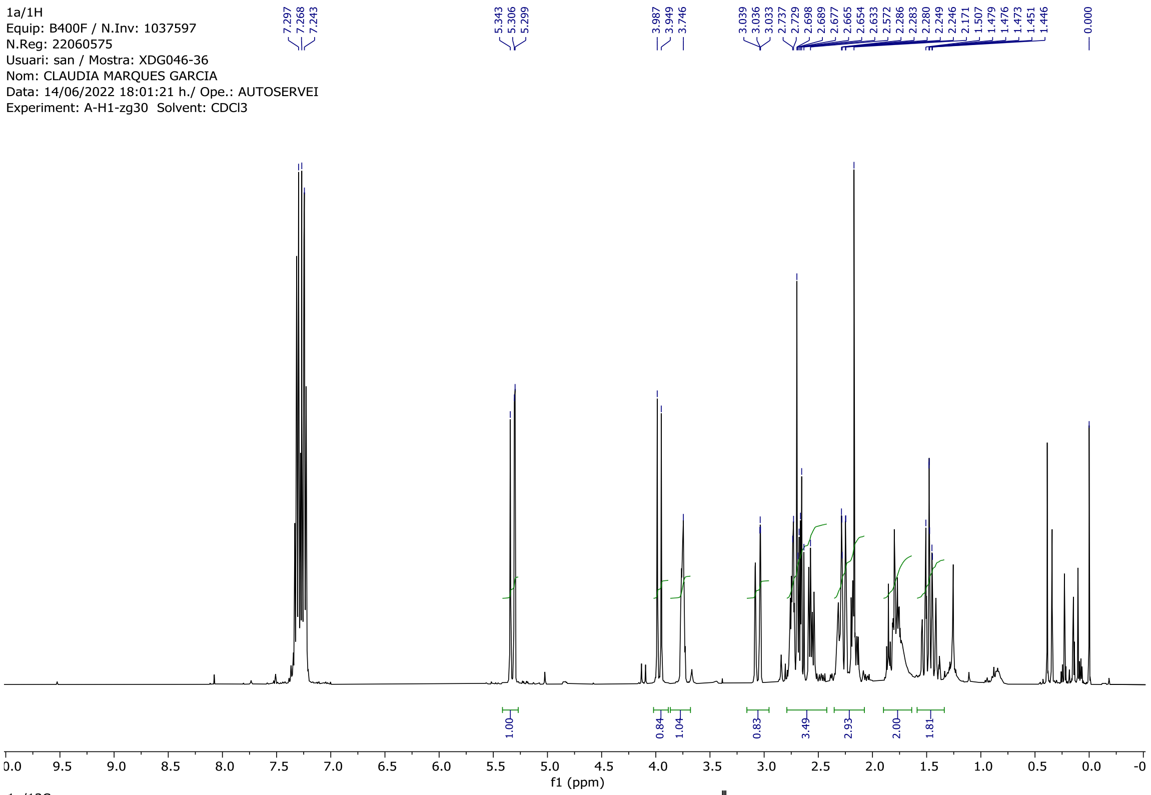

1a/13C
Equip: B400F / N.Inv: 1037597
N.Reg: 22060575
Usuari: san / Mostra: XDG046-36
Nom: CLAUDIA MARQUES GARCIA
Data: 14/06/2022 18:01:21 h./ Ope.: AUTOSERVEI
Experiment: A-C13-zgpg30 Solvent: CDCl3

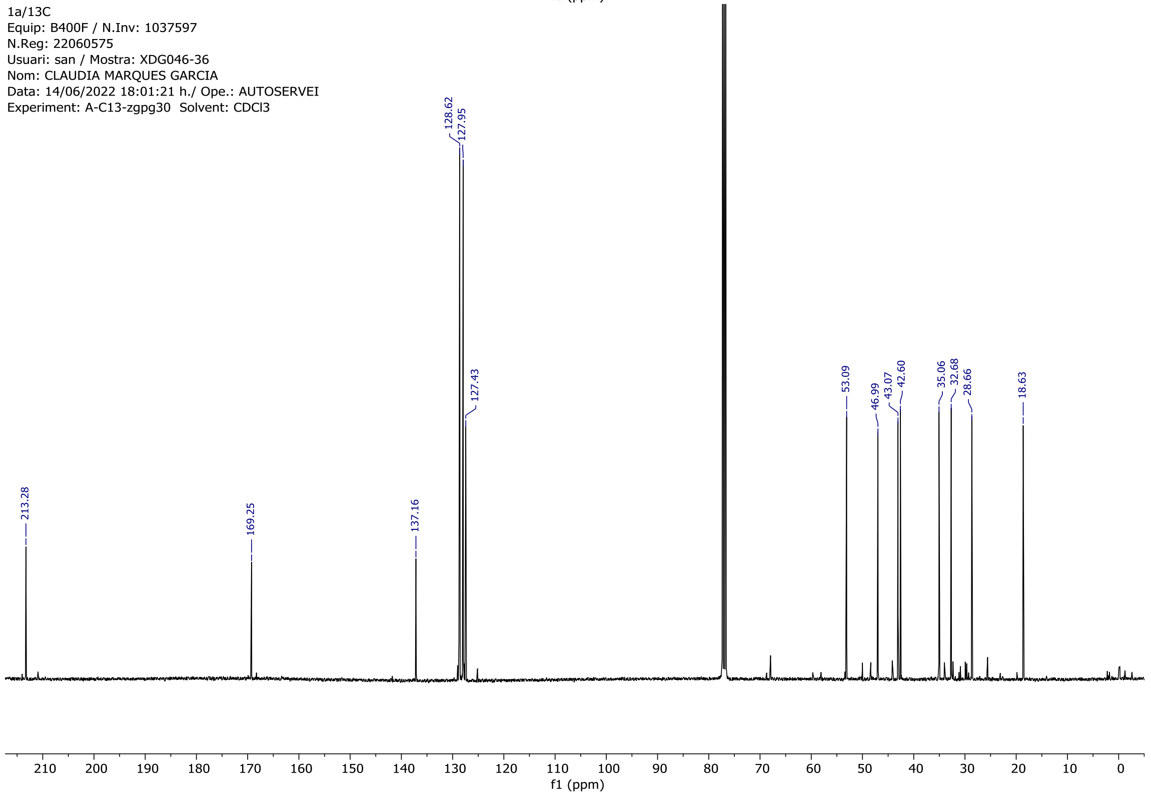

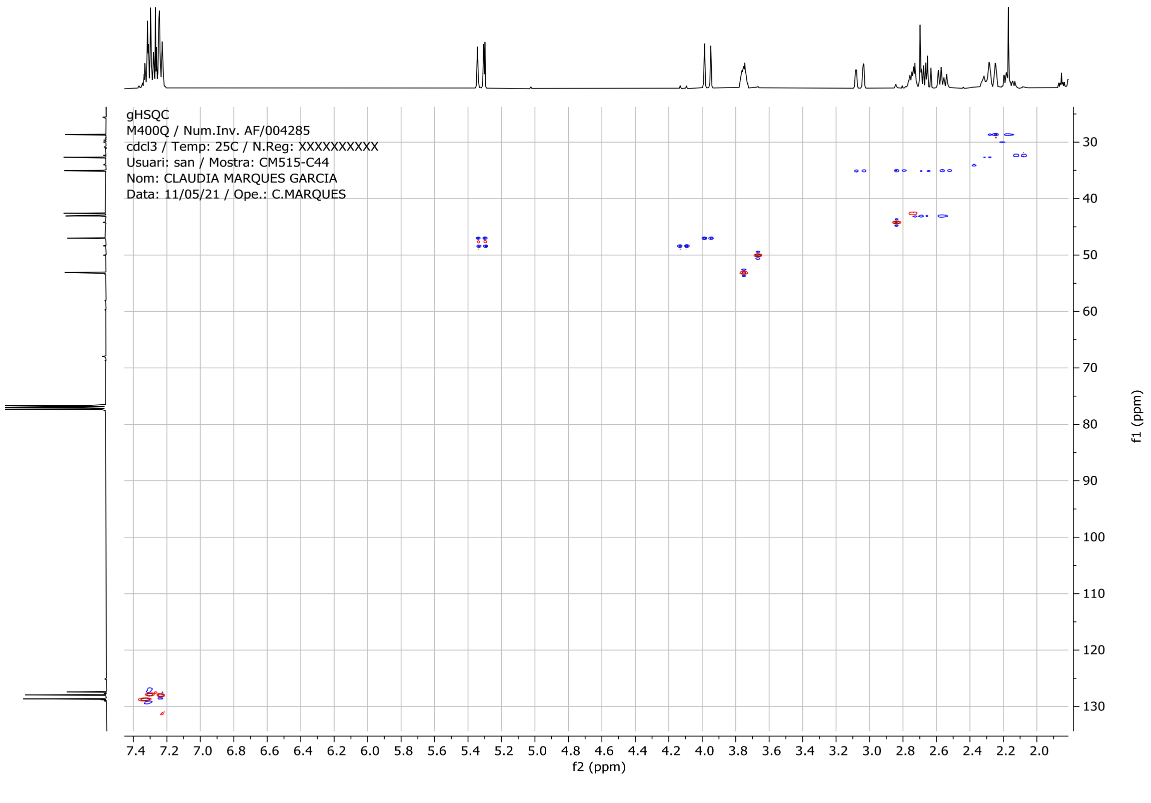
gHSQC
M400Q / Num.Inv. AF/004285
cdcl3 / Temp: 25C / N.Reg: XXXXXXXXXXX
Usuari: san / Mostra: CM515-C44
Nom: CLAUDIA MARQUES GARCIA
Data: 11/05/21 / Ope.: C.MARQUES
f2 (ppm)
f1 (ppm)

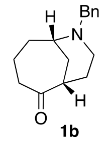

1b

1H
M400Q / Num.Inv. AF/004285
cdcl3 / Temp: 25C / N.Reg: XXXXXXXXXX
Usuari: san / Mostra: XCM607-14
Nom: CLAUDIA MARQUES GARCIA
Data: 01/10/21 / Ope.: C.MARQUES

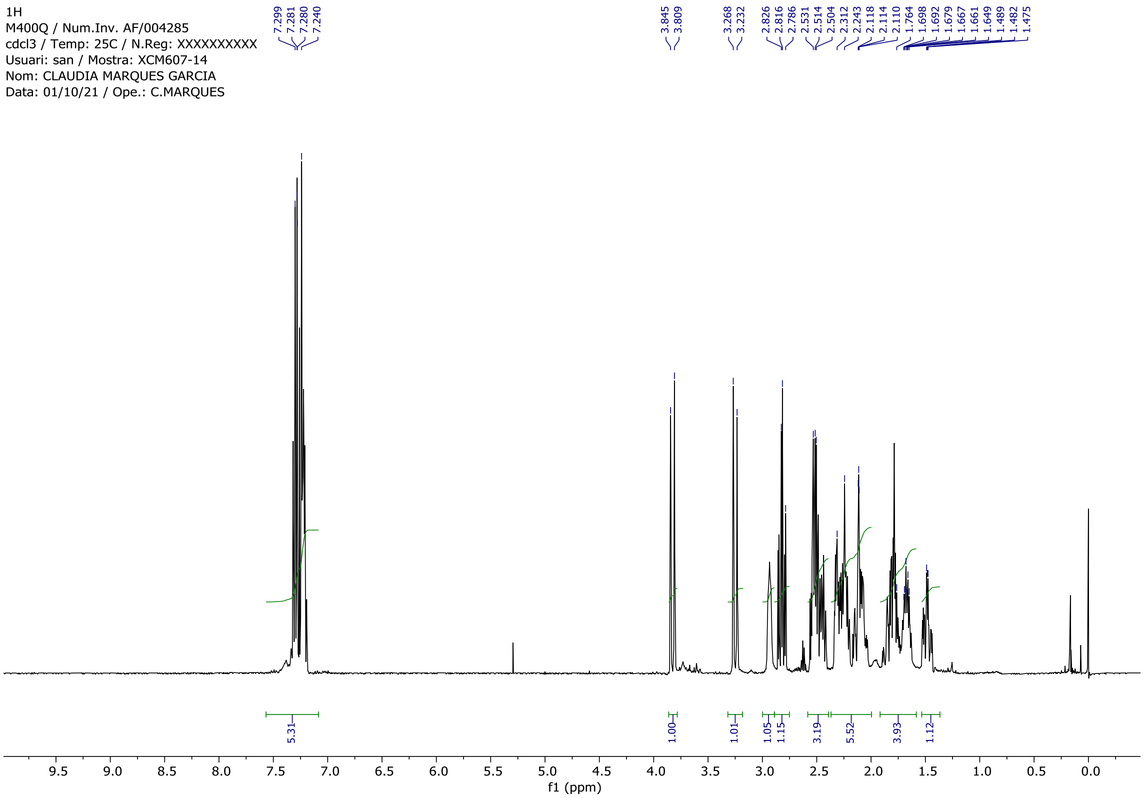

13C
M400Q / Num.Inv. AF/004285
cdcl3 / Temp: 25C / N.Reg: XXXXXXXXXX
Usuari: san / Mostra: XCM607-14
Nom: CLAUDIA MARQUES GARCIA
Data: 01/10/21 / Ope.: C.MARQUES

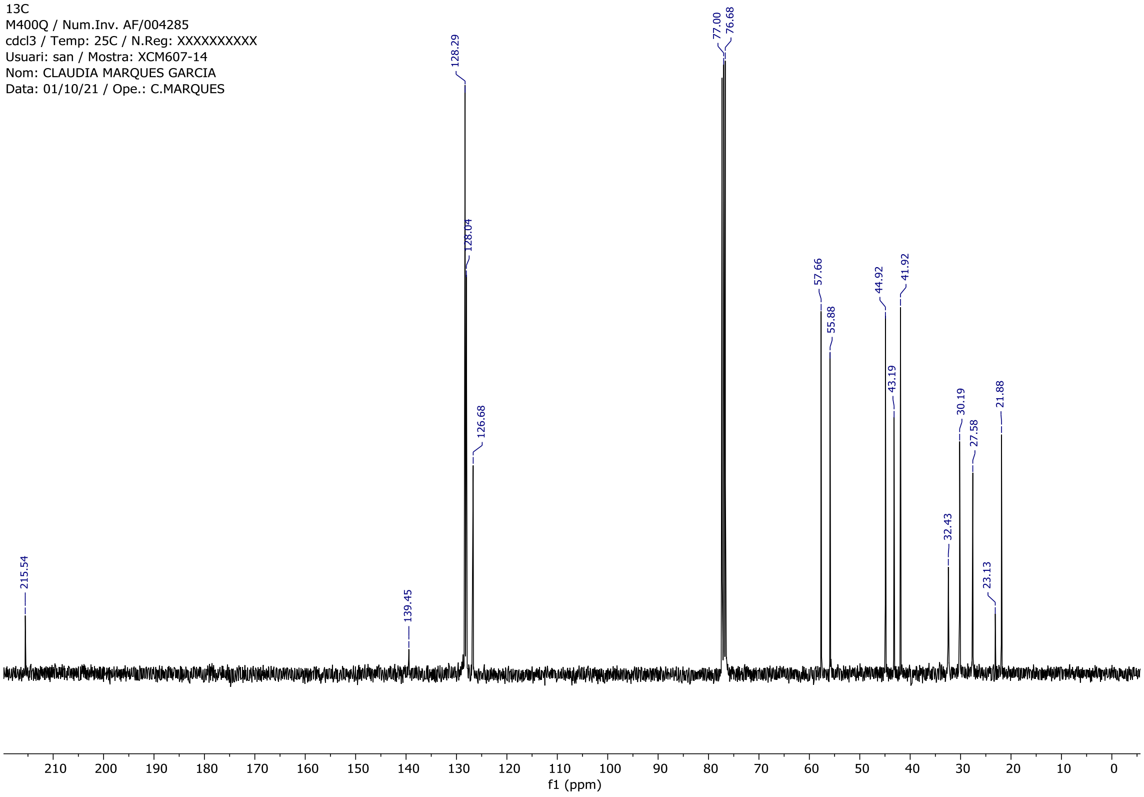

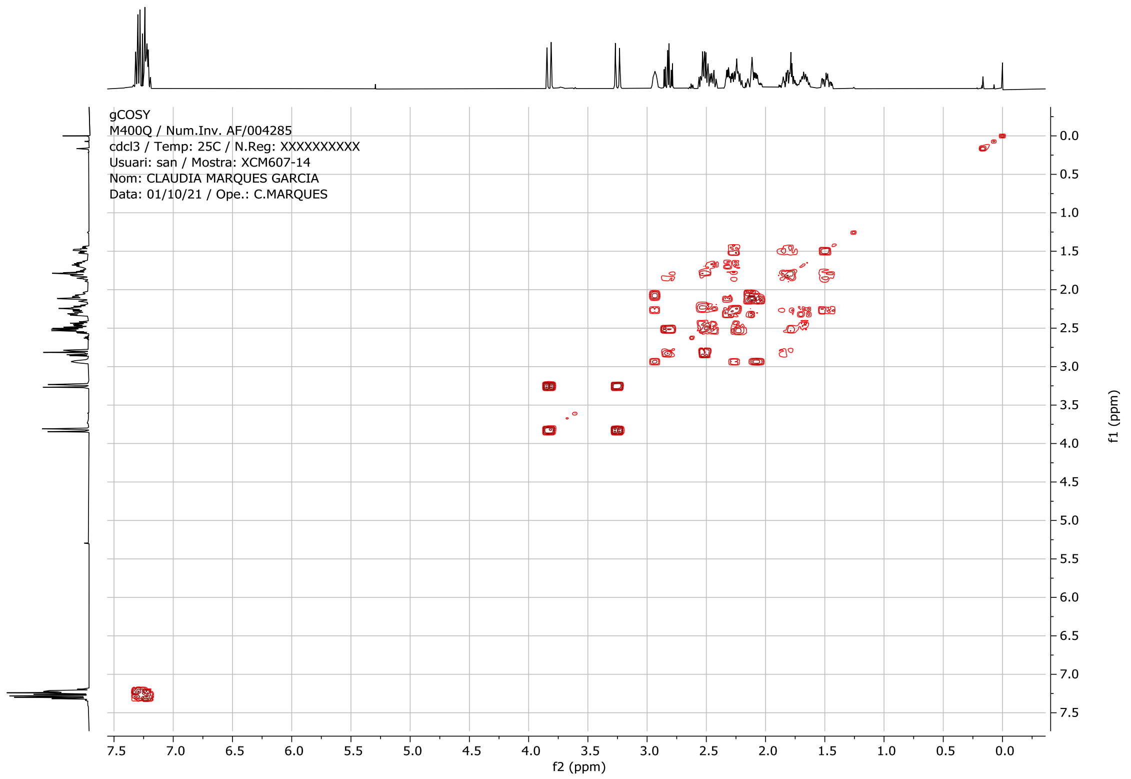
gCOSY
M400Q / Num.Inv. AF/004285
cdcl3 / Temp: 25C / N.Reg: XXXXXXXXXX
Usuari: san / Mostra: XCM607-14
Nom: CLAUDIA MARQUES GARCIA
Data: 01/10/21 / Ope.: C.MARQUES
f2 (ppm)
f1 (ppm)

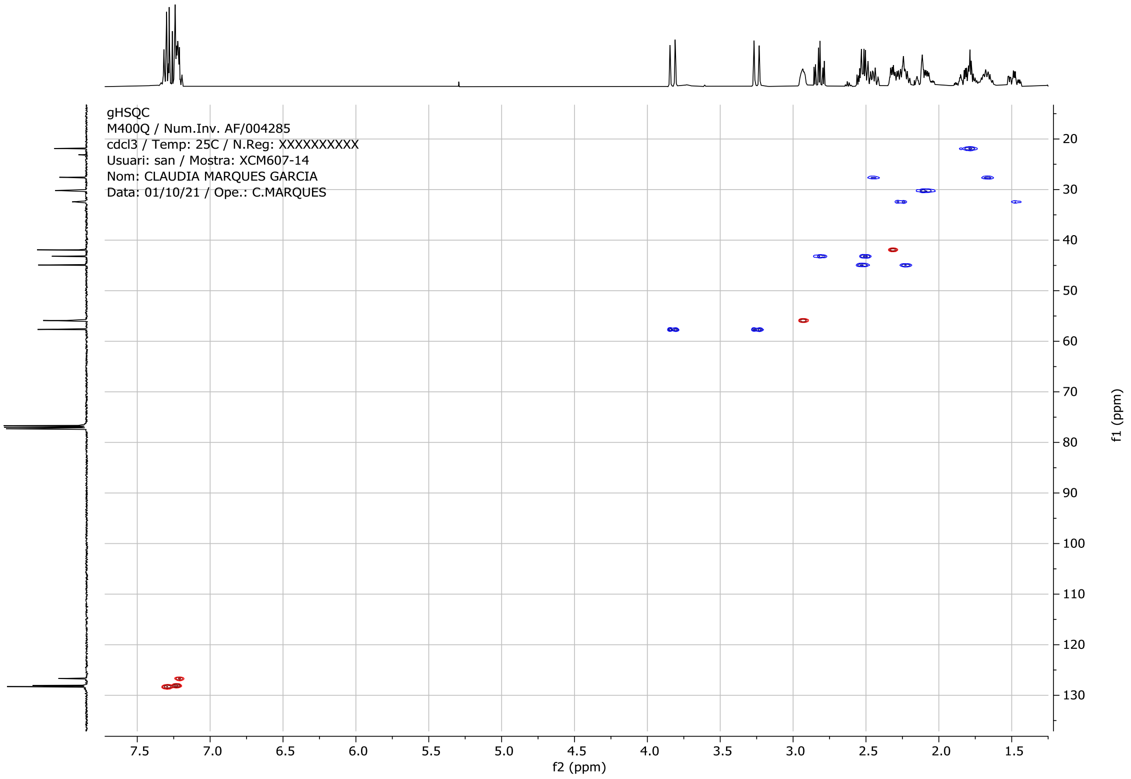
gHSQC
M400Q / Num.Inv. AF/004285
cdcl3 / Temp: 25C / N.Reg: XXXXXXXXXX
Usuari: san / Mostra: XCM607-14
Nom: CLAUDIA MARQUES GARCIA
Data: 01/10/21 / Ope.: C.MARQUES
f2 (ppm)
f1 (ppm)

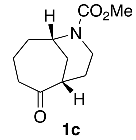

1c

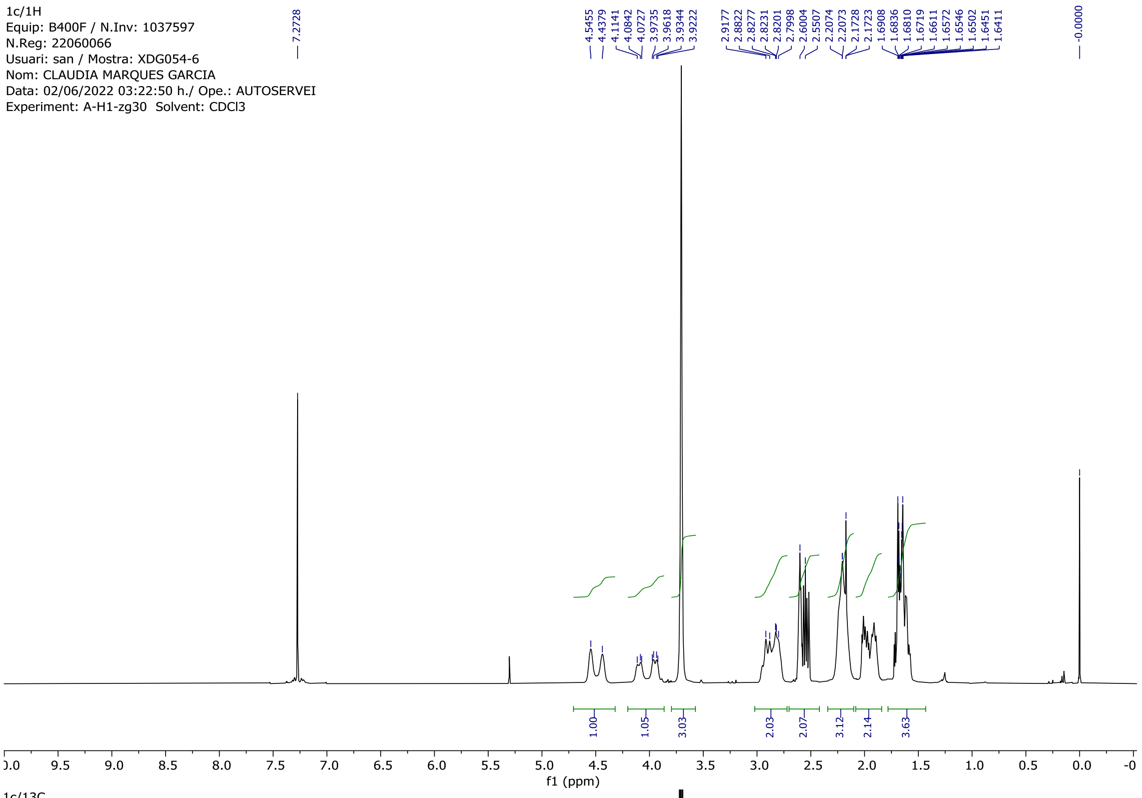

1c/1H
Equip: B400F / N.Inv: 1037597
N.Reg: 22060066
Usuari: san / Mostra: XDG054-6
Nom: CLAUDIA MARQUES GARCIA
Data: 02/06/2022 03:22:50 h./ Ope.: AUTOSERVEI
Experiment: A-H1-zg30 Solvent: CDCl3
f1 (ppm)

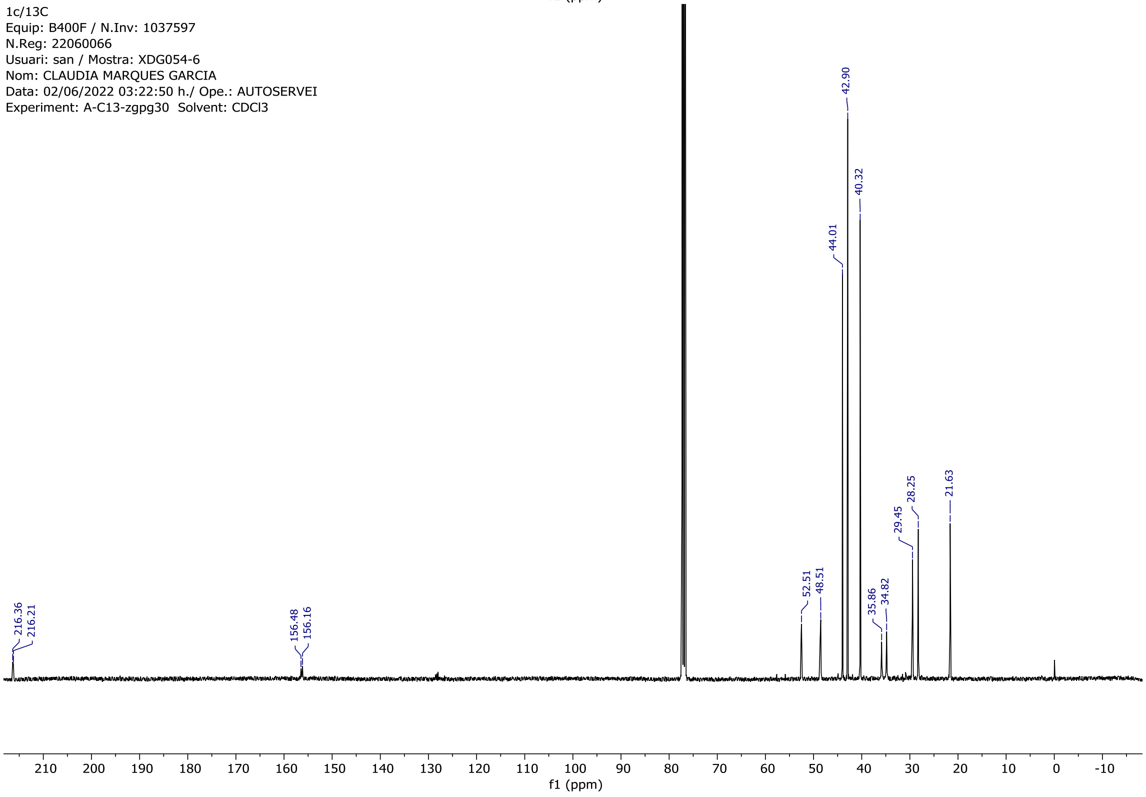

1c/13C
Equip: B400F / N.Inv: 1037597
N.Reg: 22060066
Usuari: san / Mostra: XDG054-6
Nom: CLAUDIA MARQUES GARCIA
Data: 02/06/2022 03:22:50 h./ Ope.: AUTOSERVEI
Experiment: A-C13-zgpg30 Solvent: CDCl3
f1 (ppm)

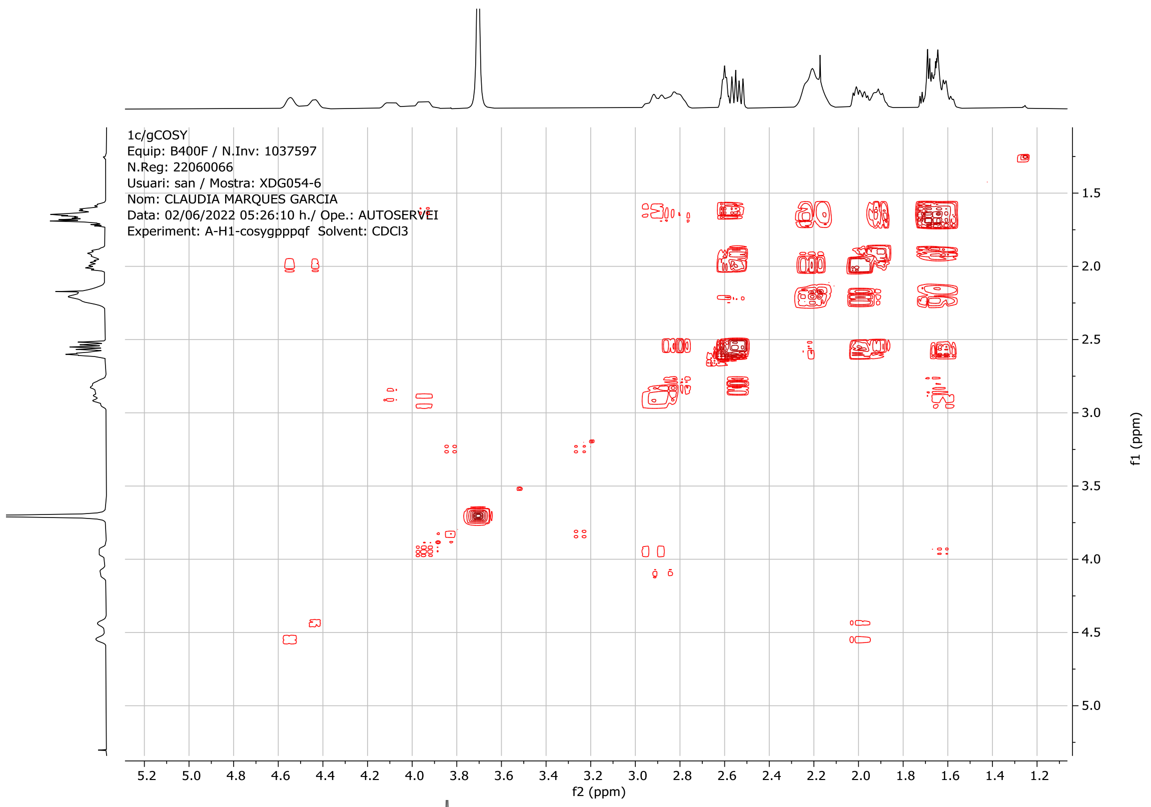
1c/gCOSY
Equip: B400F / N.Inv: 1037597
N.Reg: 22060066
Usuari: san / Mostra: XDG054-6
Nom: CLAUDIA MARQUES GARCIA
Data: 02/06/2022 05:26:10 h./ Ope.: AUTOSERVEI
Experiment: A-H1-cosygpppqf Solvent: CDCl3
f2 (ppm)
f1 (ppm)

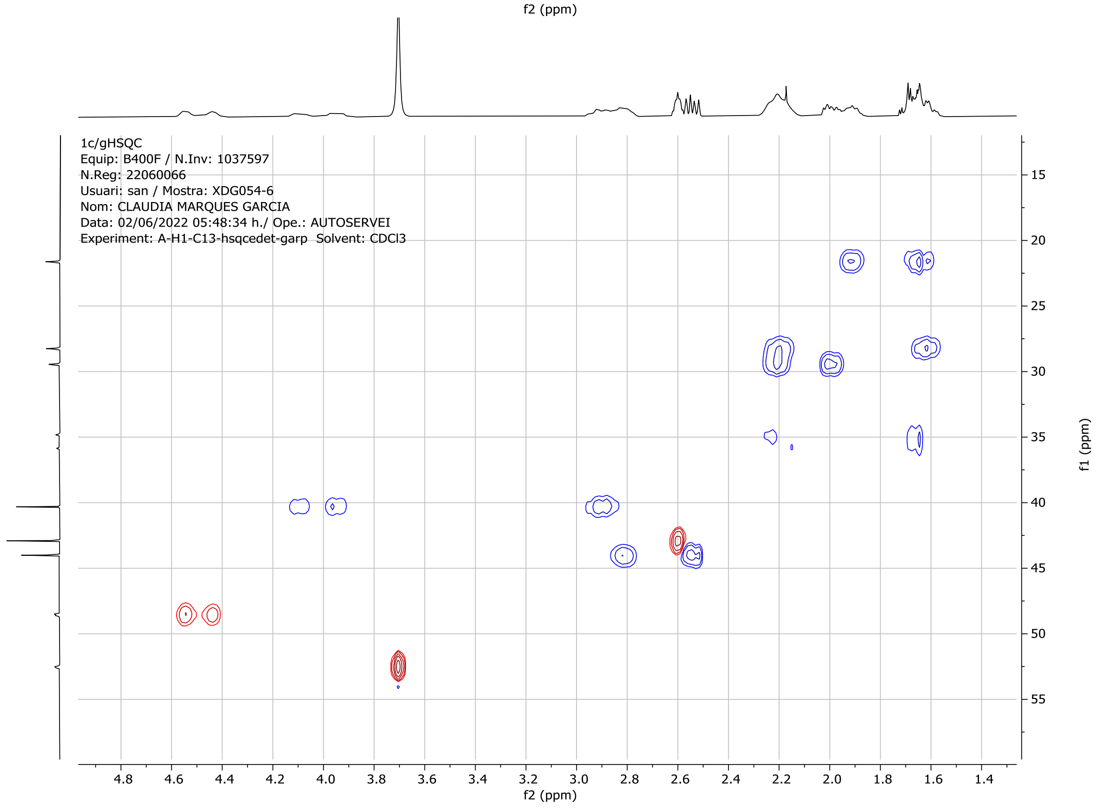
1c/gHSQC
Equip: B400F / N.Inv: 1037597
N.Reg: 22060066
Usuari: san / Mostra: XDG054-6
Nom: CLAUDIA MARQUES GARCIA
Data: 02/06/2022 05:48:34 h./ Ope.: AUTOSERVEI
Experiment: A-H1-C13-hsqcedet-garp Solvent: CDCl3
f2 (ppm)
f1 (ppm)

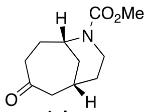

1c'

1c'/1H
Equip: B400F / N.Inv: 1037597
N.Reg: 22060421
Usuari: san / Mostra: XDG055-30
Nom: CLAUDIA MARQUES GARCIA
Data: 12/06/2022 10:51:16 h./ Ope.: AUTOSERVEI
Experiment: A-H1-zg30 Solvent: CDCl3

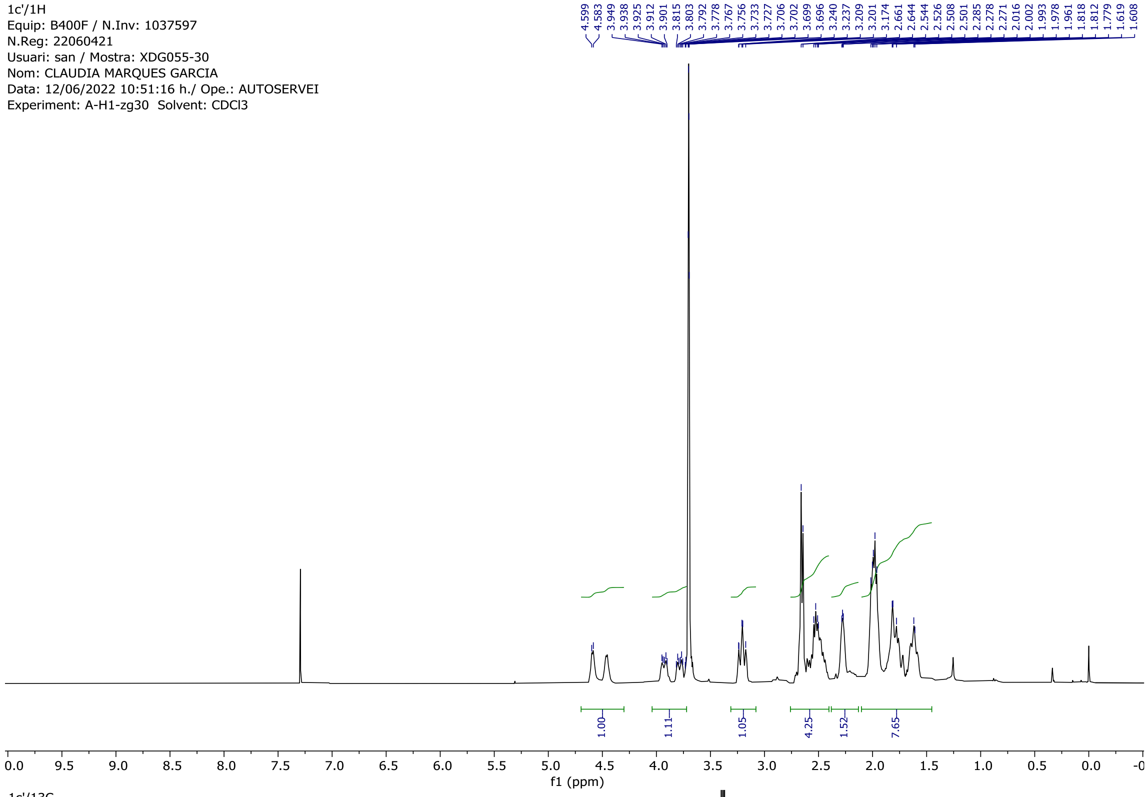

1c'/13C
Equip: B400F / N.Inv: 1037597
N.Reg: 22060421
Usuari: san / Mostra: XDG055-30
Nom: CLAUDIA MARQUES GARCIA
Data: 12/06/2022 10:51:16 h./ Ope.: AUTOSERVEI
Experiment: A-C13-zgpg30 Solvent: CDCl3

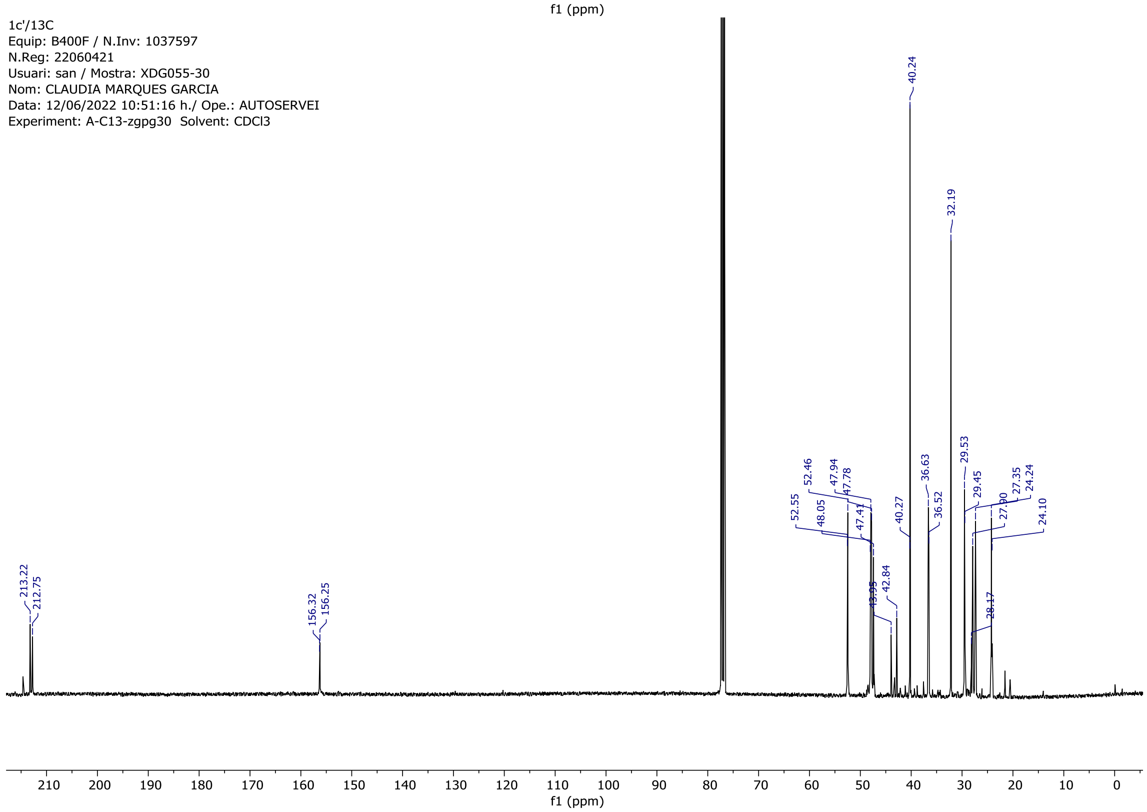

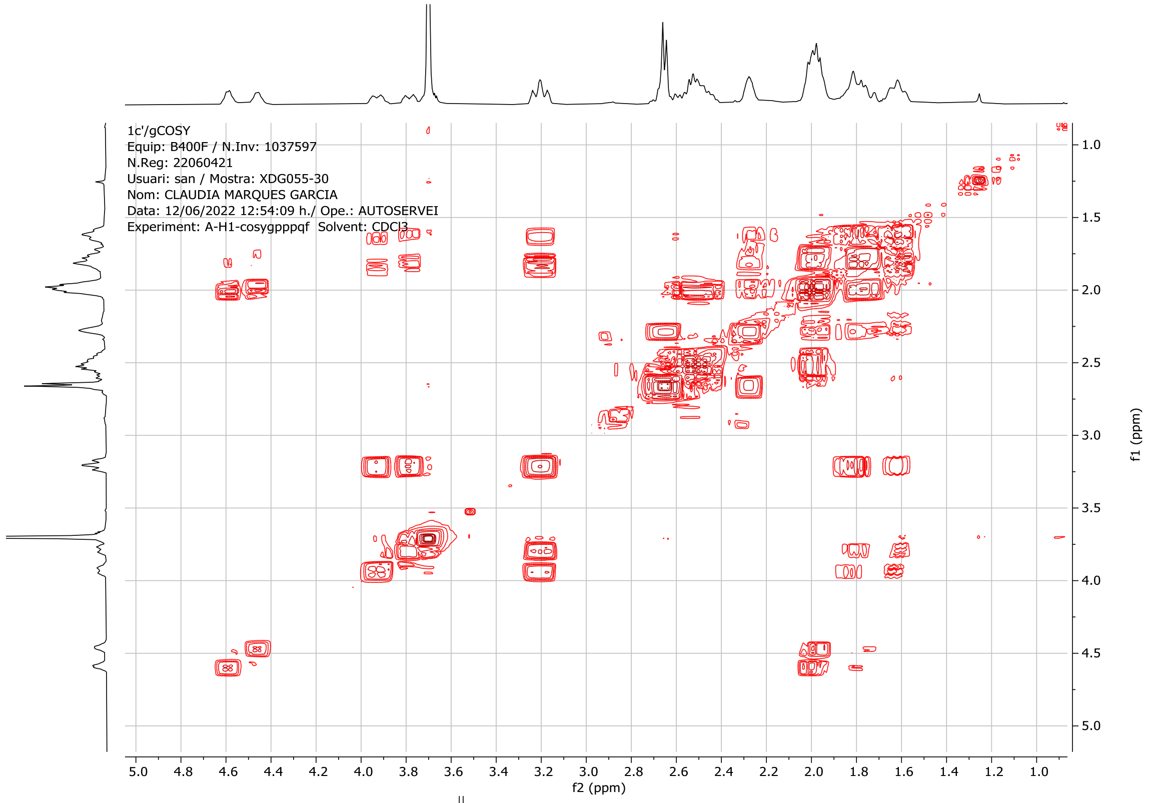

1c'/gCOSY
Equip: B400F / N.Inv: 1037597
N.Reg: 22060421
Usuari: san / Mostra: XDG055-30
Nom: CLAUDIA MARQUES GARCIA
Data: 12/06/2022 12:54:09 h./ Ope.: AUTOSERVEI
Experiment: A-H1-cosygpppqf Solvent: CDCl3
f2 (ppm)
f1 (ppm)

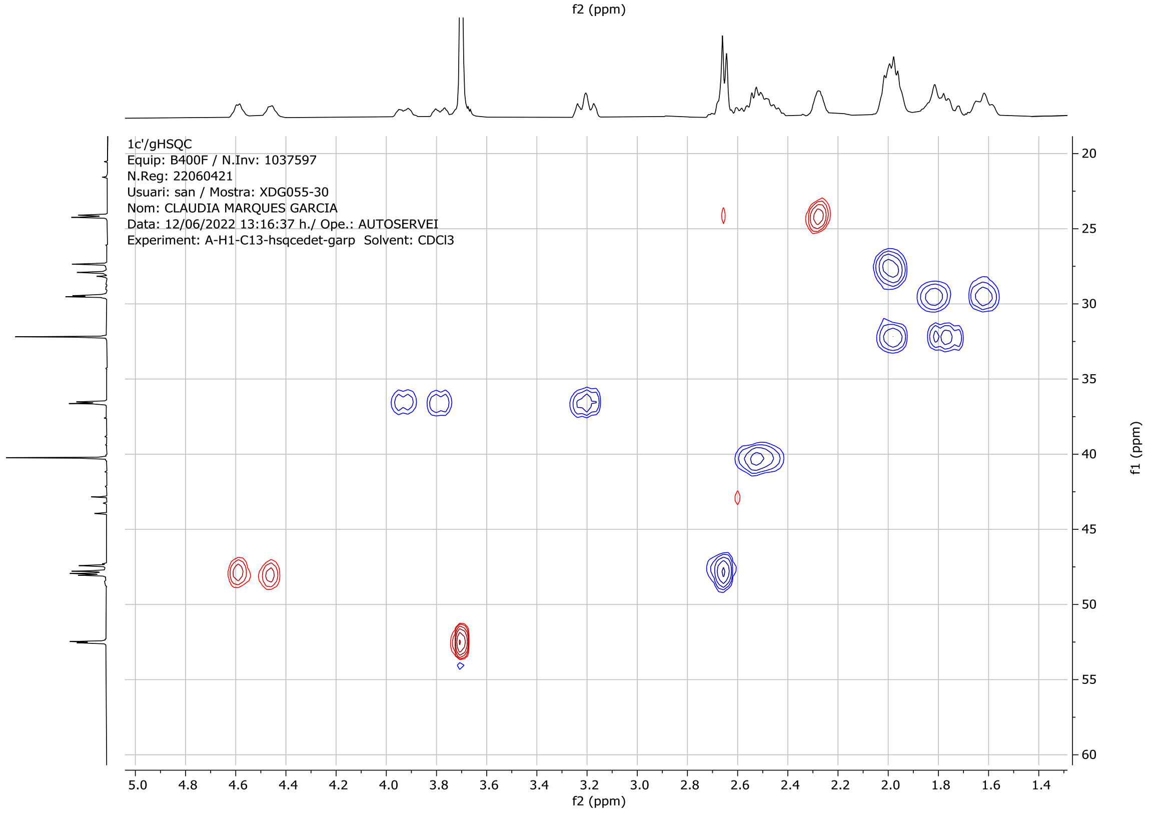

1c'/gHSQC
Equip: B400F / N.Inv: 1037597
N.Reg: 22060421
Usuari: san / Mostra: XDG055-30
Nom: CLAUDIA MARQUES GARCIA
Data: 12/06/2022 13:16:37 h./ Ope.: AUTOSERVEI
Experiment: A-H1-C13-hsqcedet-garp Solvent: CDCl3
f2 (ppm)
f1 (ppm)

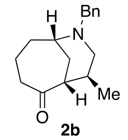

2b

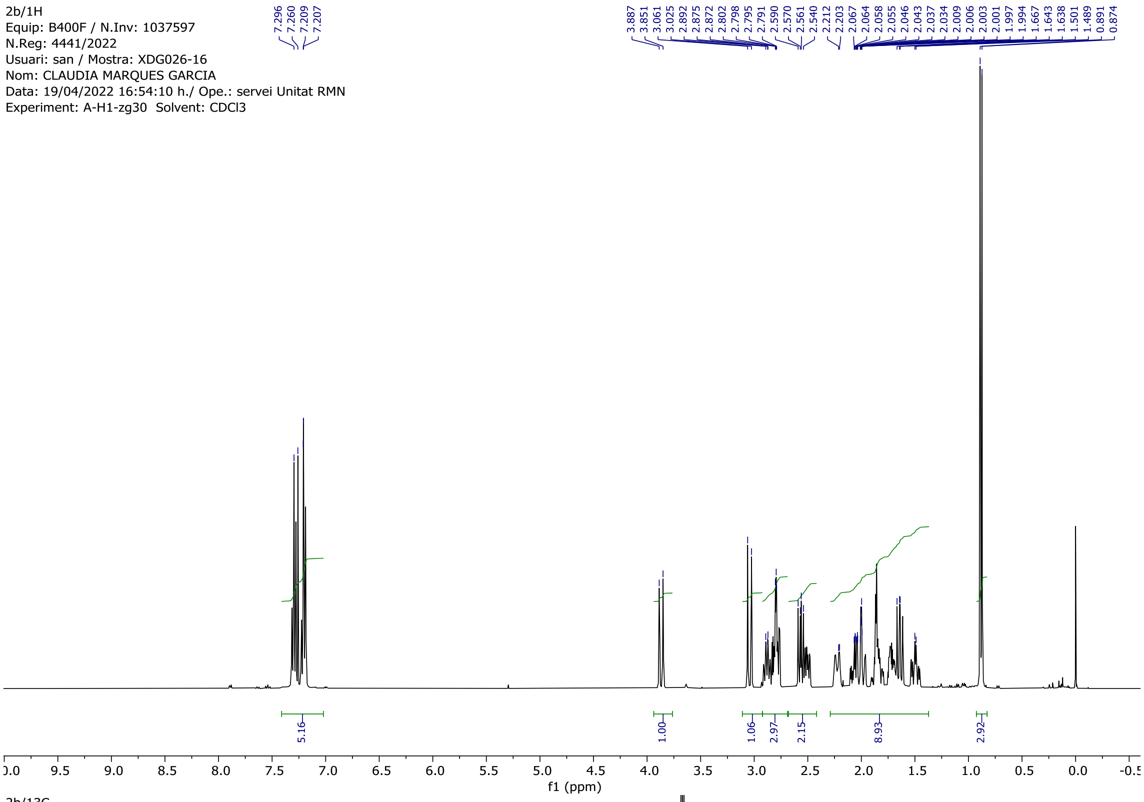
2b/1H
Equip: B400F / N.Inv: 1037597
N.Reg: 4441/2022
Usuari: san / Mostra: XDG026-16
Nom: CLAUDIA MARQUES GARCIA
Data: 19/04/2022 16:54:10 h./ Ope.: servei Unitat RMN
Experiment: A-H1-zg30 Solvent: CDCl3
f1 (ppm)

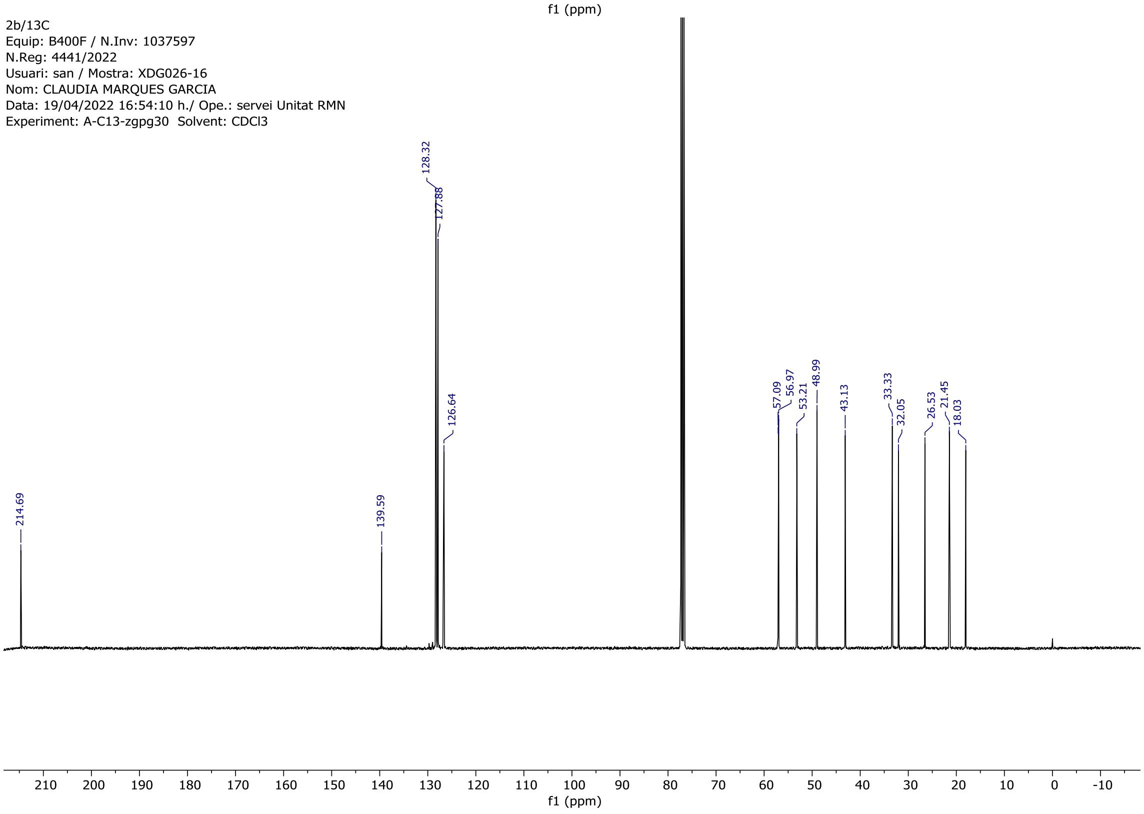
2b/13C
Equip: B400F / N.Inv: 1037597
N.Reg: 4441/2022
Usuari: san / Mostra: XDG026-16
Nom: CLAUDIA MARQUES GARCIA
Data: 19/04/2022 16:54:10 h./ Ope.: servei Unitat RMN
Experiment: A-C13-zgpg30 Solvent: CDCl3
f1 (ppm)

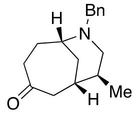
H
Bn
N
O
H
Me

2b'

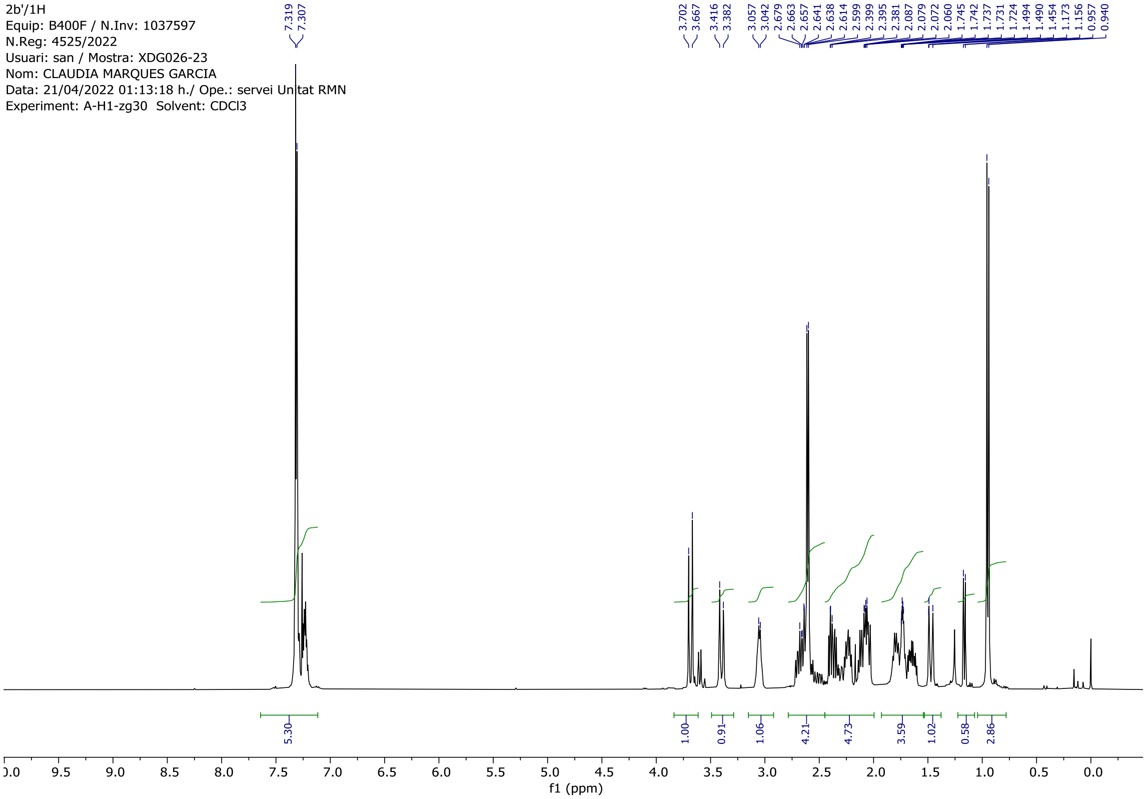
2b'/1H
Equip: B400F / N.Inv: 1037597
N.Reg: 4525/2022
Usuari: san / Mostra: XDG026-23
Nom: CLAUDIA MARQUES GARCIA
Data: 21/04/2022 01:13:18 h./ Ope.: servei Unitat RMN
Experiment: A-H1-zg30 Solvent: CDCl3
7.319
7.307
3.702
3.667
3.416
3.382
3.057
3.042
2.679
2.663
2.657
2.641
2.638
2.614
2.599
2.399
2.395
2.381
2.087
2.079
2.072
2.060
1.745
1.742
1.737
1.731
1.724
1.494
1.490
1.454
1.173
1.156
0.957
0.940
5.30
1.00
0.91
1.06
4.21
4.73
3.59
1.02
0.58
2.86
f1 (ppm)

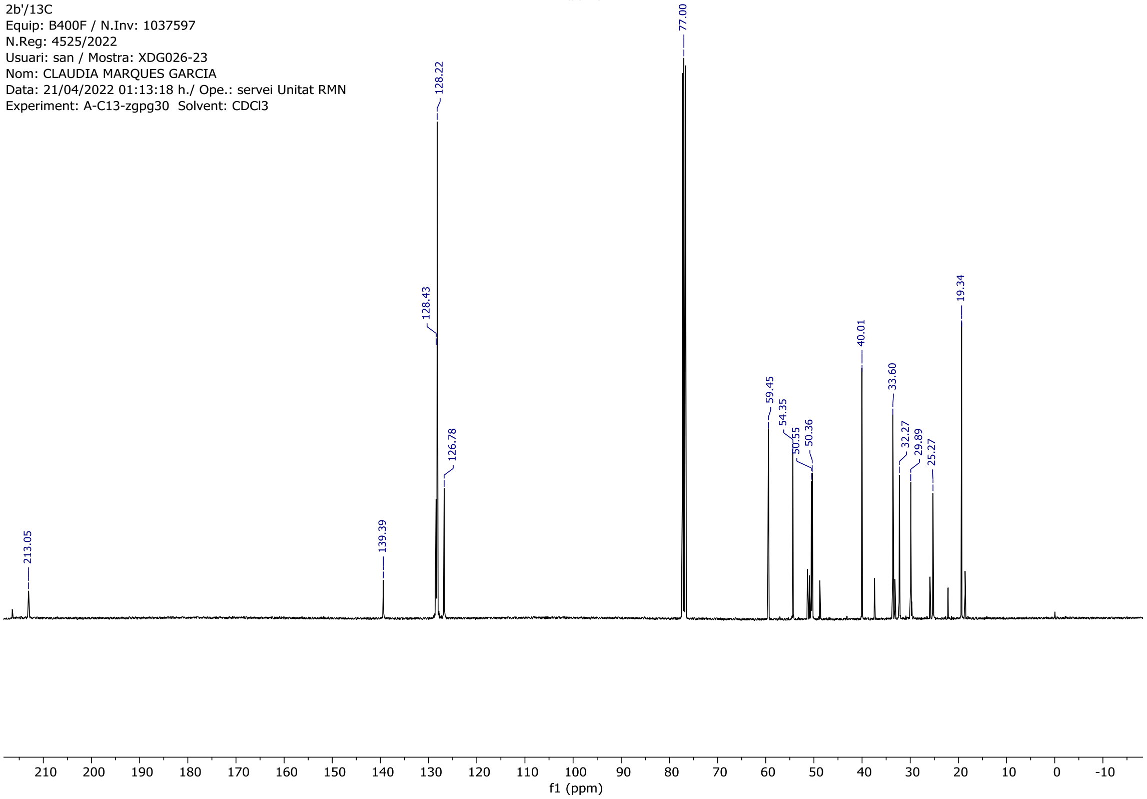
2b'/13C
Equip: B400F / N.Inv: 1037597
N.Reg: 4525/2022
Usuari: san / Mostra: XDG026-23
Nom: CLAUDIA MARQUES GARCIA
Data: 21/04/2022 01:13:18 h./ Ope.: servei Unitat RMN
Experiment: A-C13-zgpg30 Solvent: CDCl3
213.05
139.39
128.43
128.22
126.78
77.00
59.45
54.35
50.55
50.36
40.01
33.60
32.27
29.89
25.27
19.34
f1 (ppm)

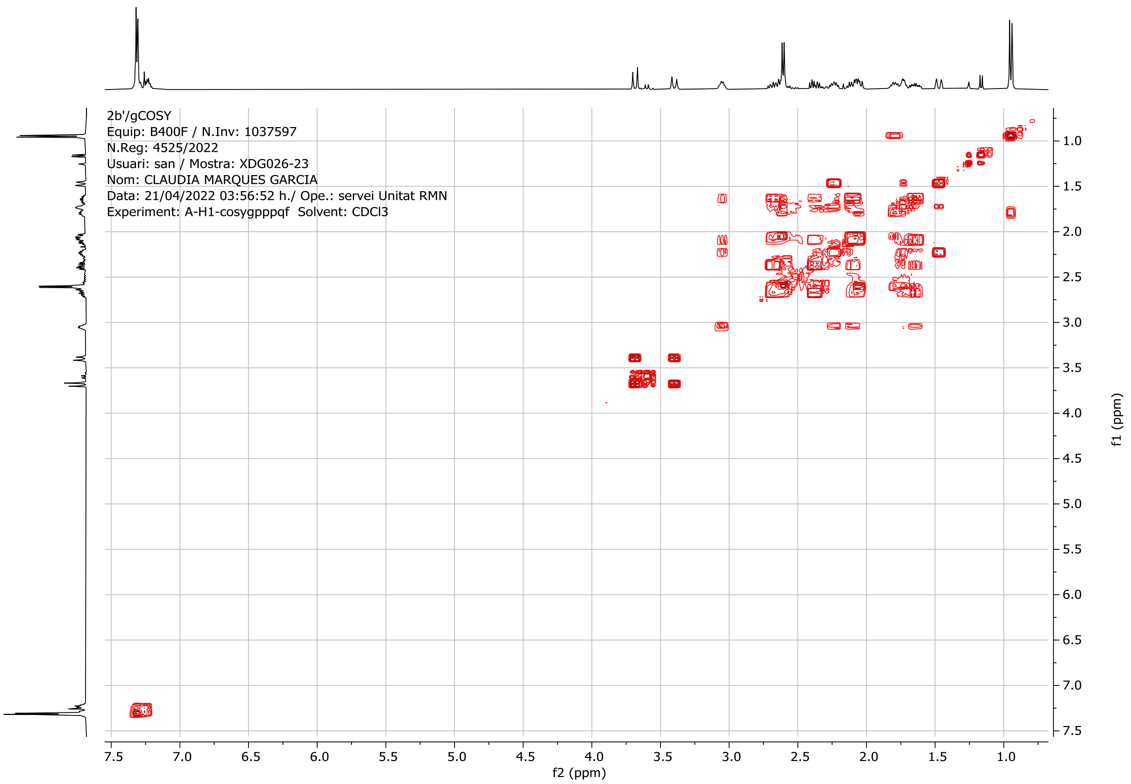
2b'/gCOSY
Equip: B400F / N.Inv: 1037597
N.Reg: 4525/2022
Usuari: san / Mostra: XDG026-23
Nom: CLAUDIA MARQUES GARCIA
Data: 21/04/2022 03:56:52 h./ Ope.: servei Unitat RMN
Experiment: A-H1-cosygpppqf Solvent: CDCl3
f2 (ppm)
f1 (ppm)

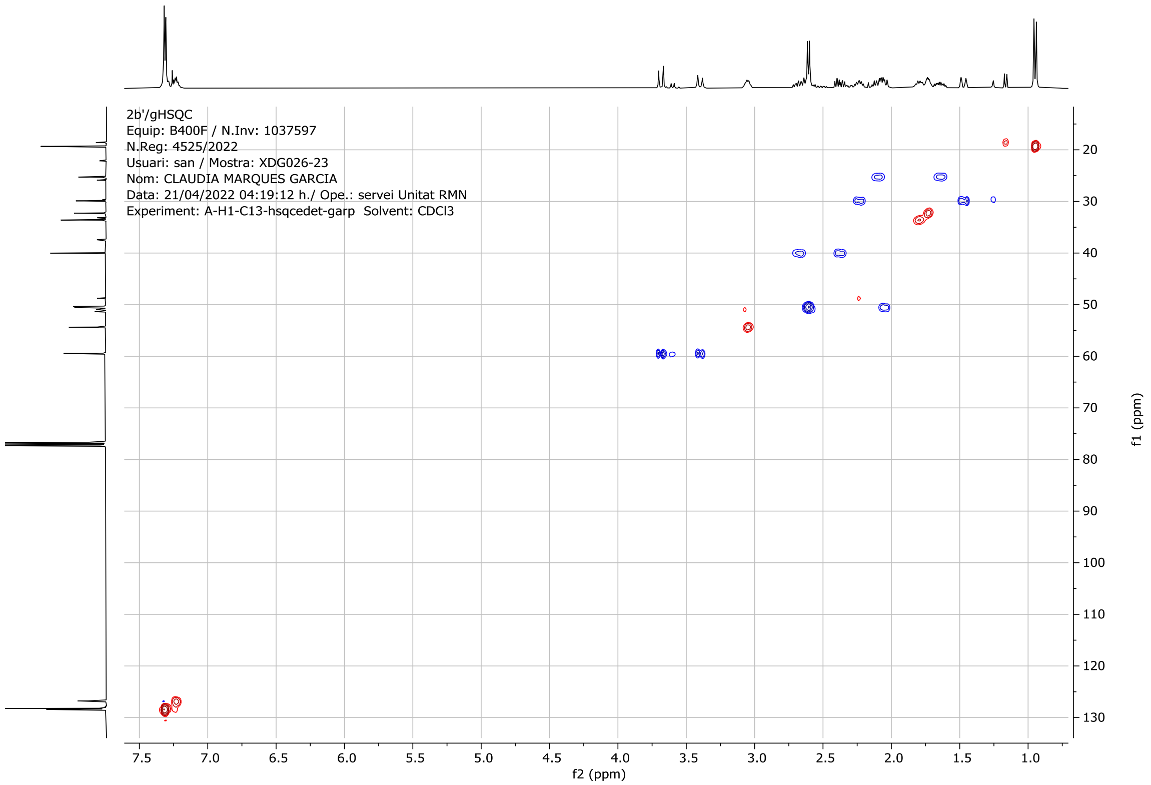
2b'/gHSQC
Equip: B400F / N.Inv: 1037597
N.Reg: 4525/2022
Usuari: san / Mostra: XDG026-23
Nom: CLAUDIA MARQUES GARCIA
Data: 21/04/2022 04:19:12 h./ Ope.: servei Unitat RMN
Experiment: A-H1-C13-hsqcedet-garp Solvent: CDCl3
f2 (ppm)
f1 (ppm)

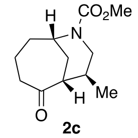

2c

2c/1H
Equip: B400F / N.Inv: 1037597
N.Reg: 22060731
Usuari: san / Mostra: XCM726-14
Nom: CLAUDIA MARQUES GARCIA
Data: 17/06/2022 11:16:02 h./ Ope.: AUTOSERVEI
Experiment: A-H1-zg30 Solvent: CDCl3

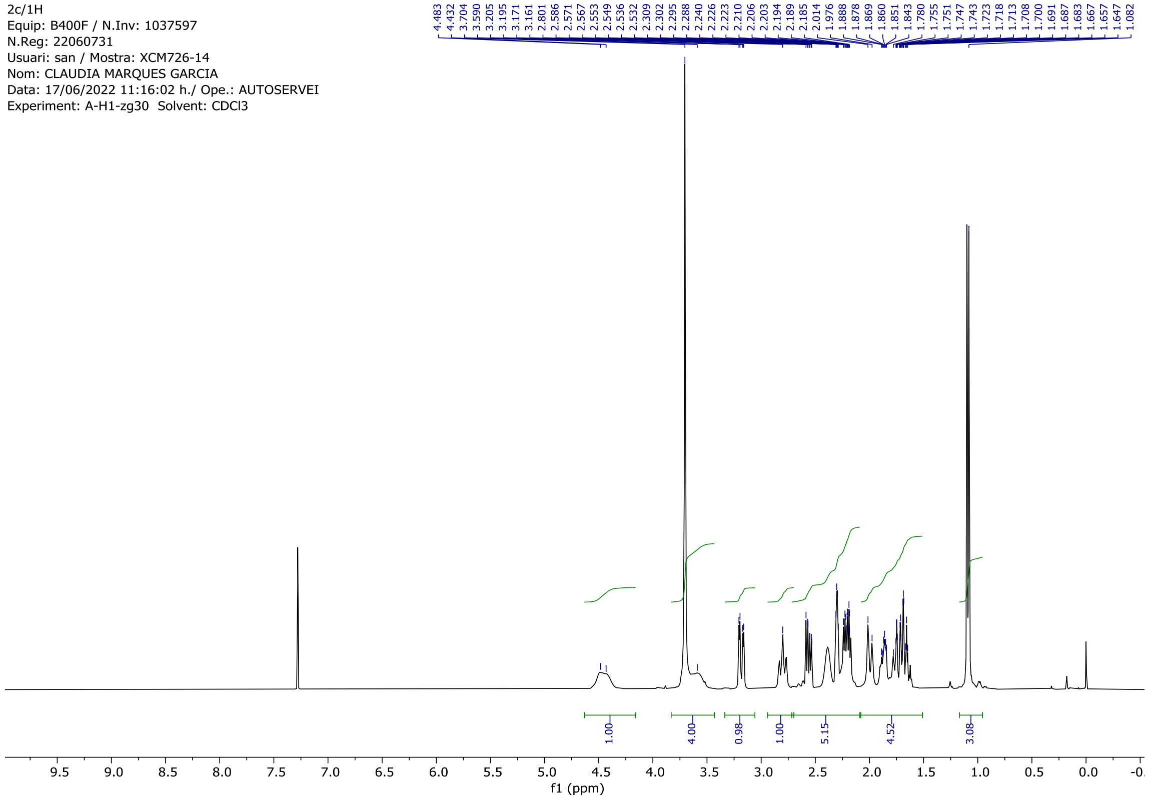

2c/13C
Equip: B400F / N.Inv: 1037597
N.Reg: 22060731
Usuari: san / Mostra: XCM726-14
Nom: CLAUDIA MARQUES GARCIA
Data: 18/06/2022 09:08:57 h./ Ope.: AUTOSERVEI
Experiment: A-C13-zgpg30 Solvent: CDCl3

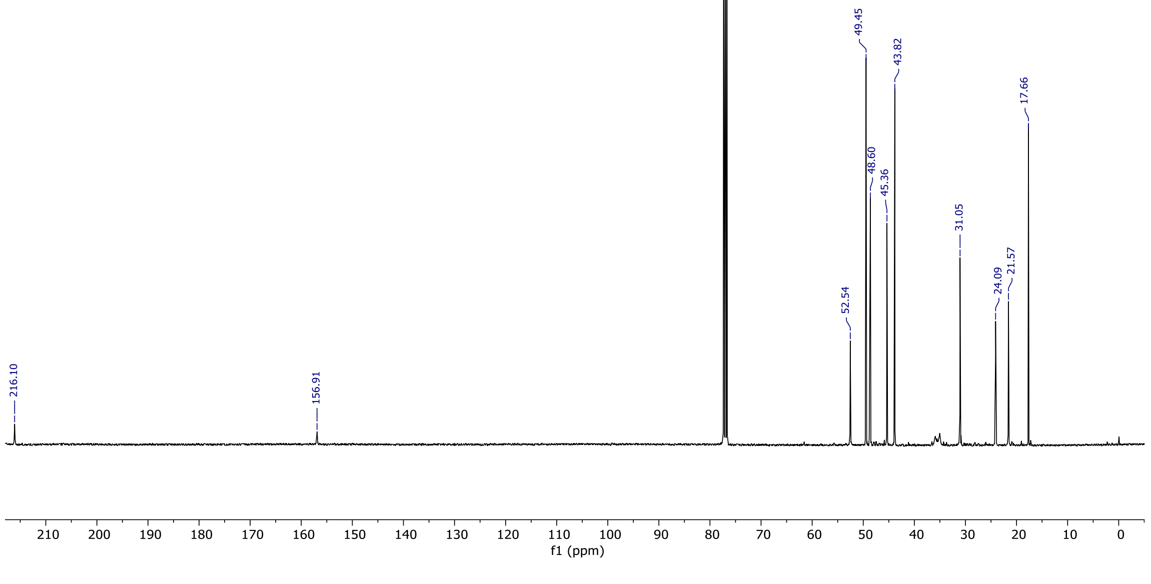

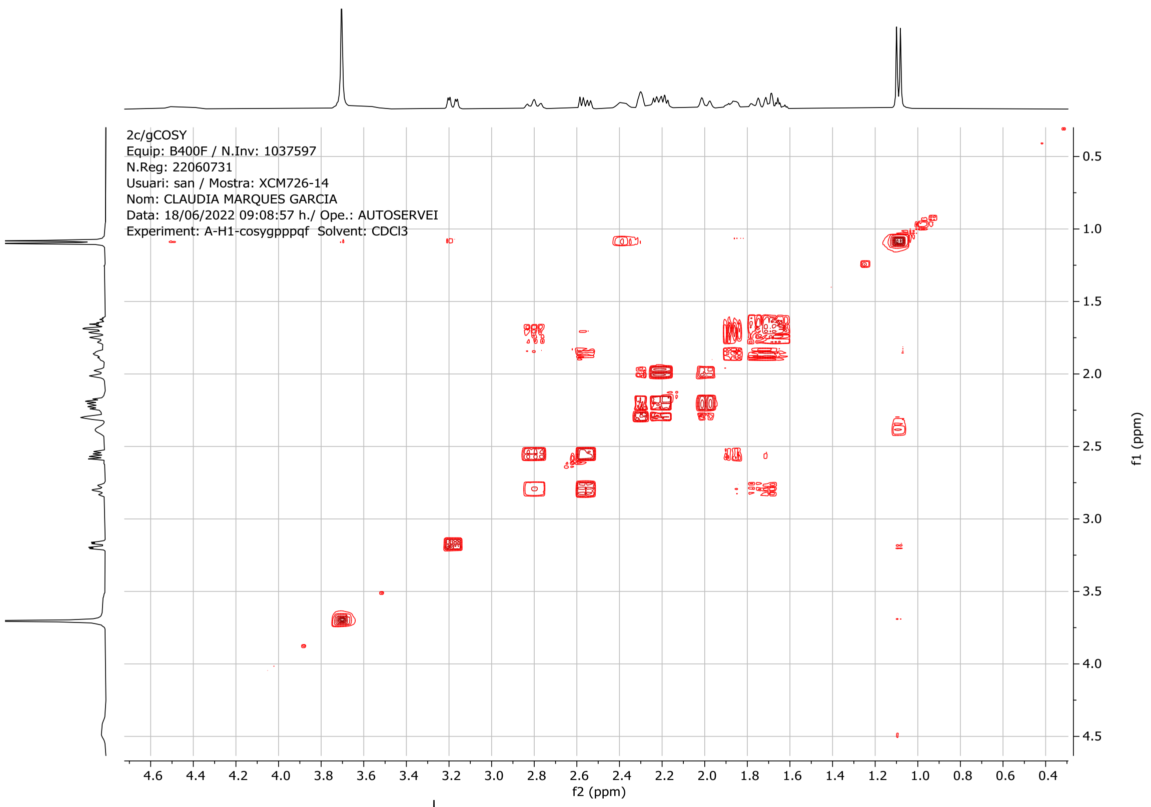
2c/gCOSY
Equip: B400F / N.Inv: 1037597
N.Reg: 22060731
Usuari: san / Mostra: XCM726-14
Nom: CLAUDIA MARQUES GARCIA
Data: 18/06/2022 09:08:57 h./ Ope.: AUTOSERVEI
Experiment: A-H1-cosygpppqf Solvent: CDCl3
f2 (ppm)
f1 (ppm)

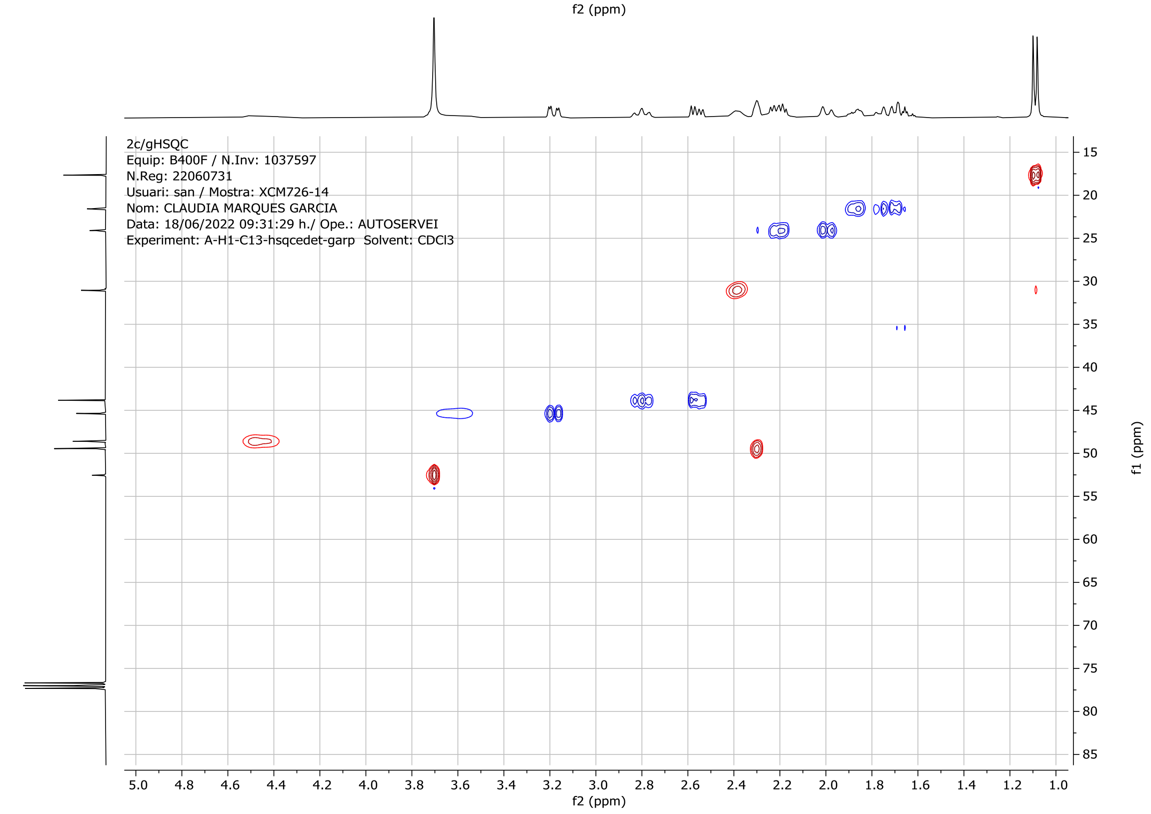
2c/gHSQC
Equip: B400F / N.Inv: 1037597
N.Reg: 22060731
Usuari: san / Mostra: XCM726-14
Nom: CLAUDIA MARQUES GARCIA
Data: 18/06/2022 09:31:29 h./ Ope.: AUTOSERVEI
Experiment: A-H1-C13-hsqcedet-garp Solvent: CDCl3
f2 (ppm)
f1 (ppm)

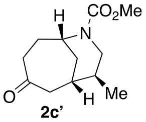
H
CO2Me
N
O
H
Me

2c'

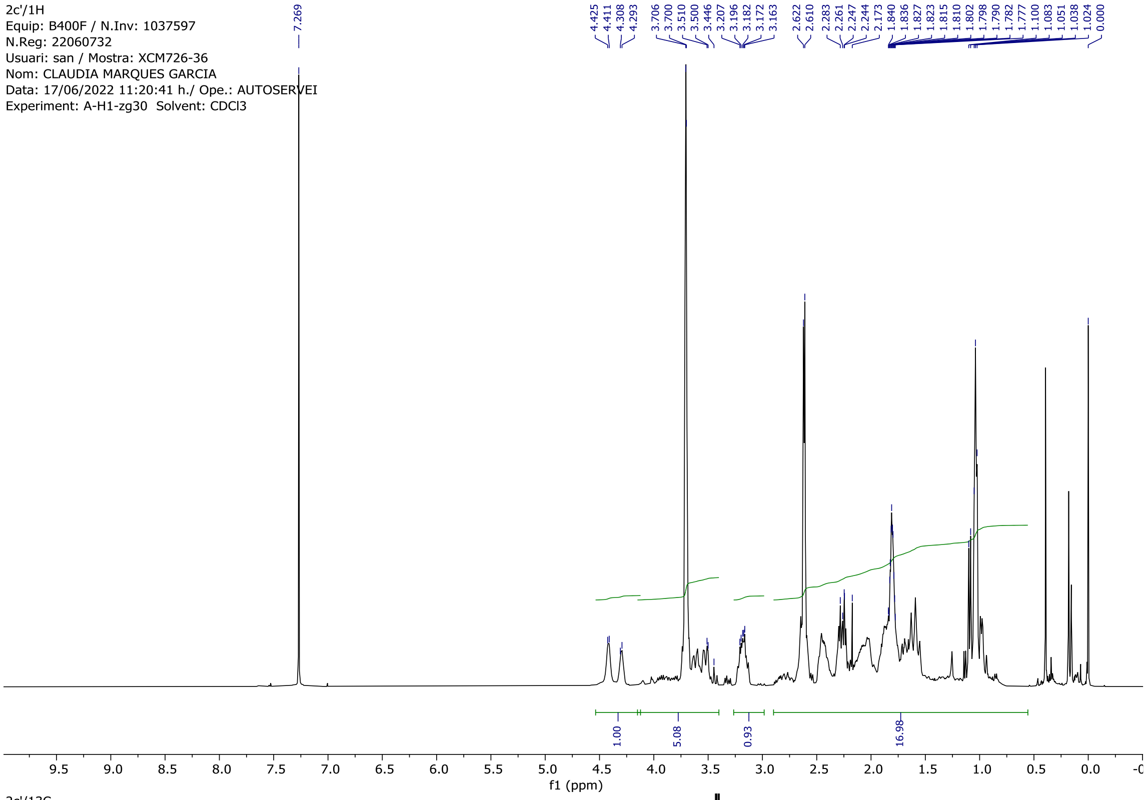
2c'/1H
Equip: B400F / N.Inv: 1037597
N.Reg: 22060732
Usuari: san / Mostra: XCM726-36
Nom: CLAUDIA MARQUES GARCIA
Data: 17/06/2022 11:20:41 h./ Ope.: AUTOSERVEI
Experiment: A-H1-zg30 Solvent: CDCl3
7.269
4.425
4.411
4.308
4.293
3.706
3.700
3.510
3.500
3.446
3.207
3.196
3.182
3.172
3.163
2.622
2.610
2.283
2.261
2.247
2.244
2.173
1.840
1.836
1.827
1.823
1.815
1.810
1.802
1.798
1.790
1.782
1.777
1.100
1.083
1.051
1.038
1.024
0.000
1.00
5.08
0.93
16.98
9.5 9.0 8.5 8.0 7.5 7.0 6.5 6.0 5.5 5.0 4.5 4.0 3.5 3.0 2.5 2.0 1.5 1.0 0.5 0.0
f1 (ppm)

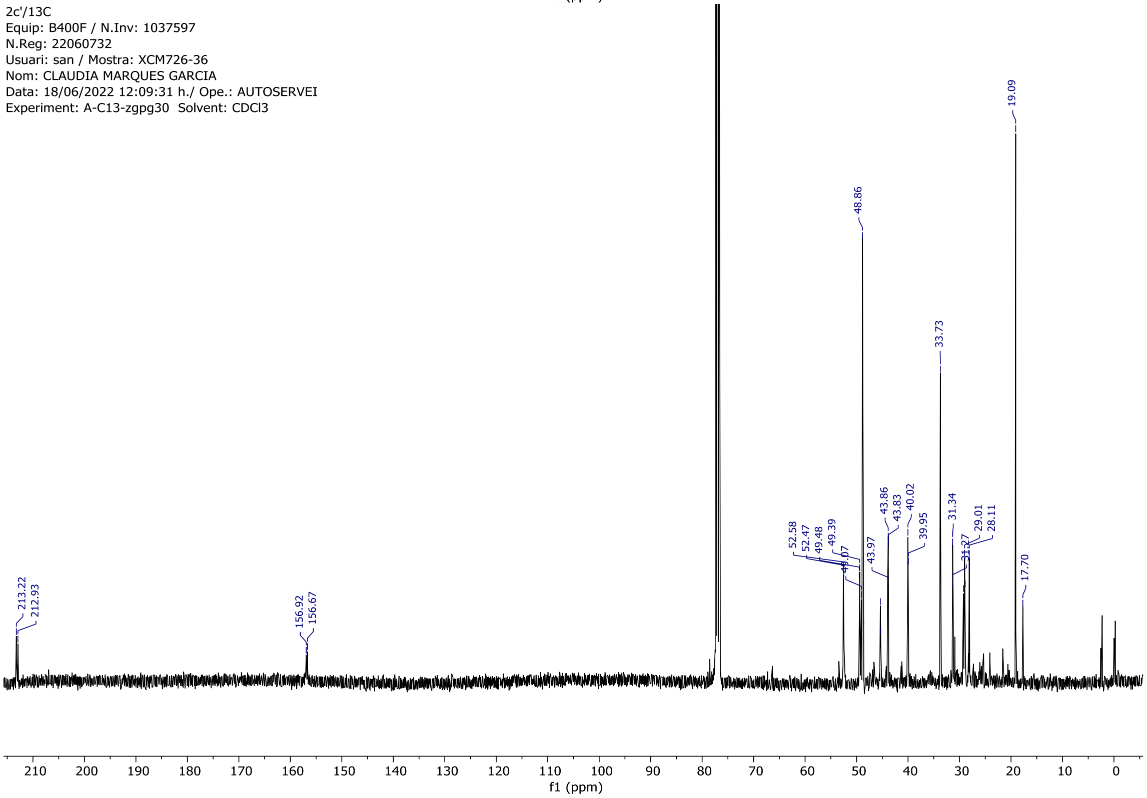
2c'/13C
Equip: B400F / N.Inv: 1037597
N.Reg: 22060732
Usuari: san / Mostra: XCM726-36
Nom: CLAUDIA MARQUES GARCIA
Data: 18/06/2022 12:09:31 h./ Ope.: AUTOSERVEI
Experiment: A-C13-zgpg30 Solvent: CDCl3
213.22
212.93
156.92
156.67
52.58
52.47
49.48
49.39
49.07
48.86
43.97
43.86
43.83
40.02
39.95
33.73
31.34
31.27
29.01
28.11
19.09
17.70
210 200 190 180 170 160 150 140 130 120 110 100 90 80 70 60 50 40 30 20 10 0
f1 (ppm)

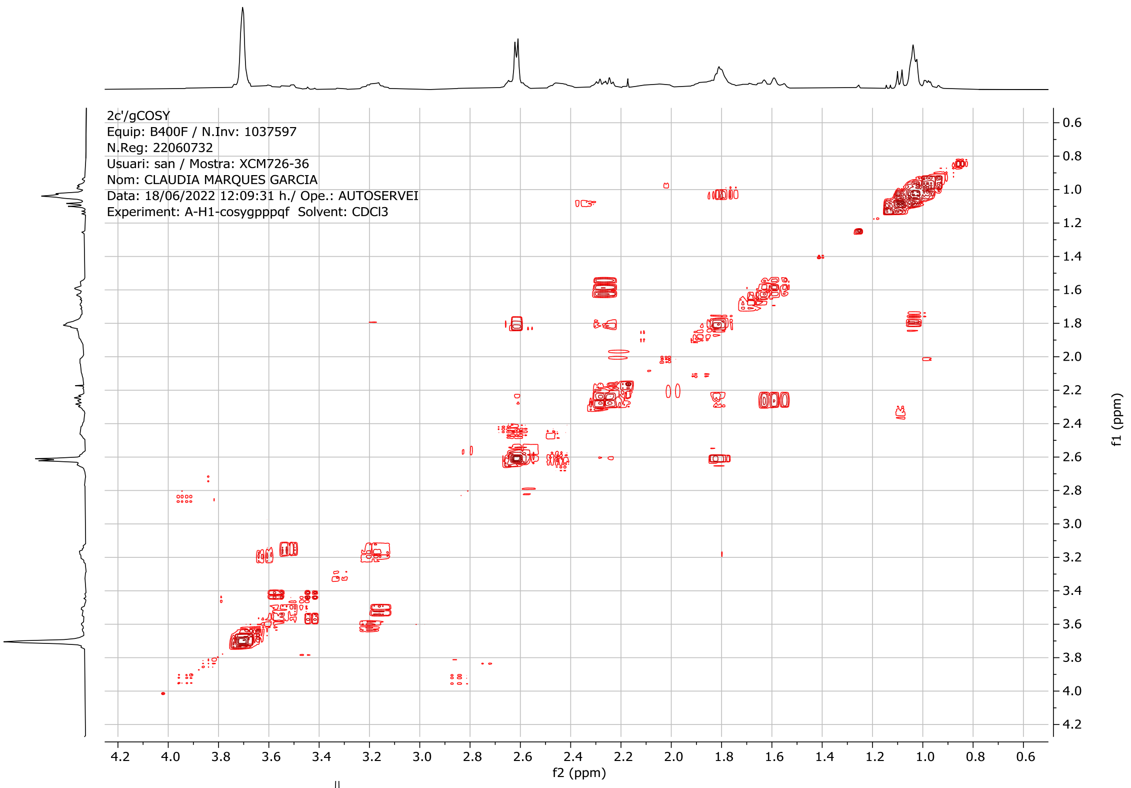
2c'/gCOSY
Equip: B400F / N.Inv: 1037597
N.Reg: 22060732
Usuari: san / Mostra: XCM726-36
Nom: CLAUDIA MARQUES GARCIA
Data: 18/06/2022 12:09:31 h./ Ope.: AUTOSERVEI
Experiment: A-H1-cosygpppqf Solvent: CDCl3
f1 (ppm)
f2 (ppm)

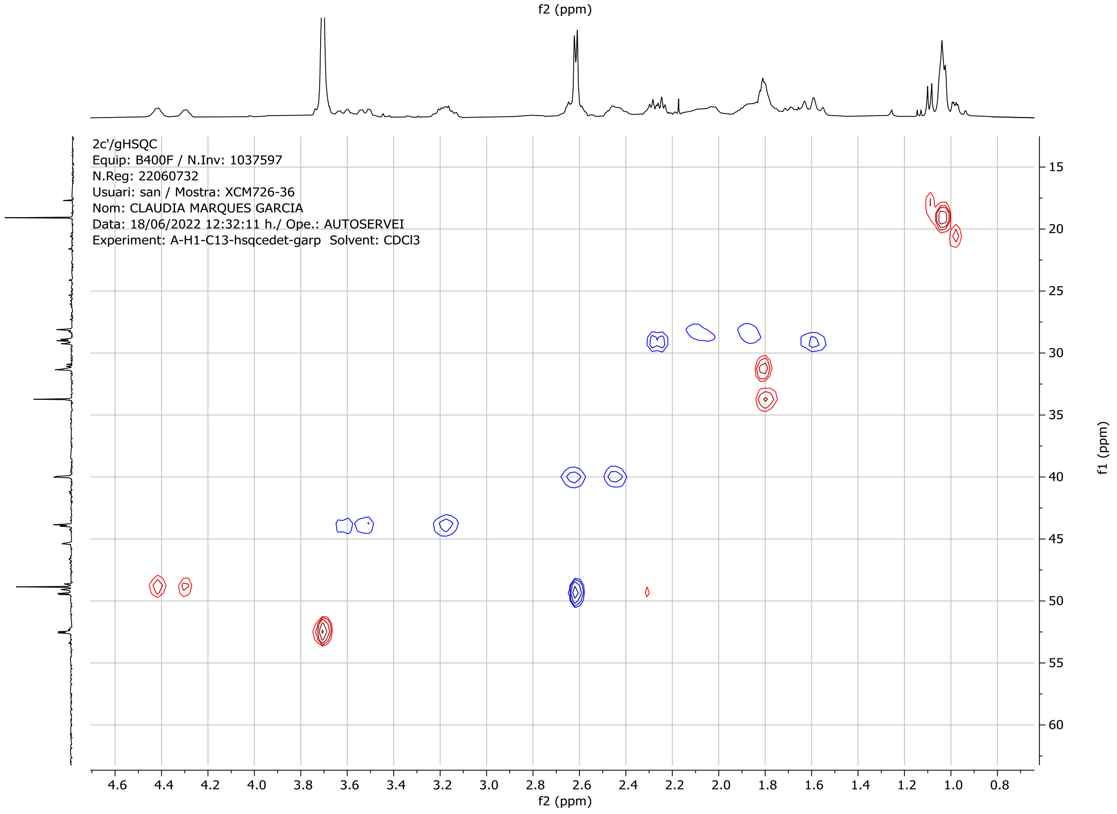
2c'/gHSQC
Equip: B400F / N.Inv: 1037597
N.Reg: 22060732
Usuari: san / Mostra: XCM726-36
Nom: CLAUDIA MARQUES GARCIA
Data: 18/06/2022 12:32:11 h./ Ope.: AUTOSERVEI
Experiment: A-H1-C13-hsqcedet-garp Solvent: CDCl3
f1 (ppm)
f2 (ppm)

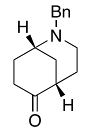

5b

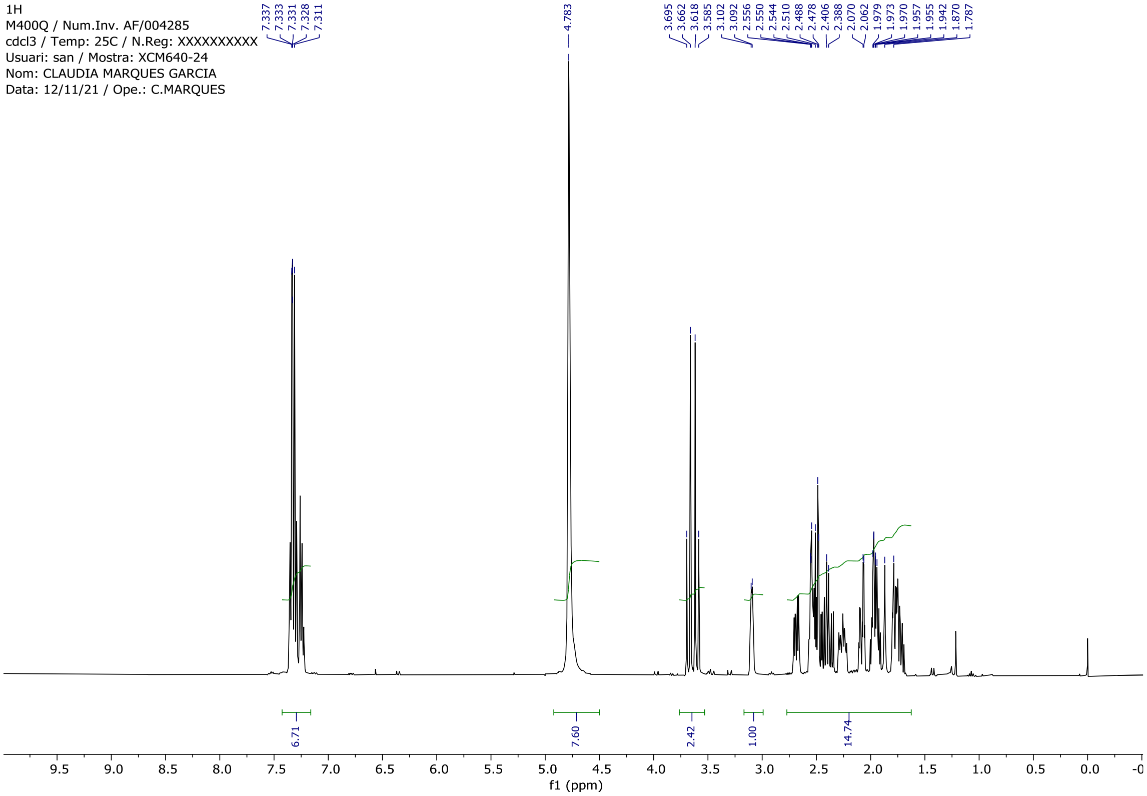
1H
M400Q / Num.Inv. AF/004285
cdcl3 / Temp: 25C / N.Reg: XXXXXXXXXX
Usuari: san / Mostra: XCM640-24
Nom: CLAUDIA MARQUES GARCIA
Data: 12/11/21 / Ope.: C.MARQUES
f1 (ppm)

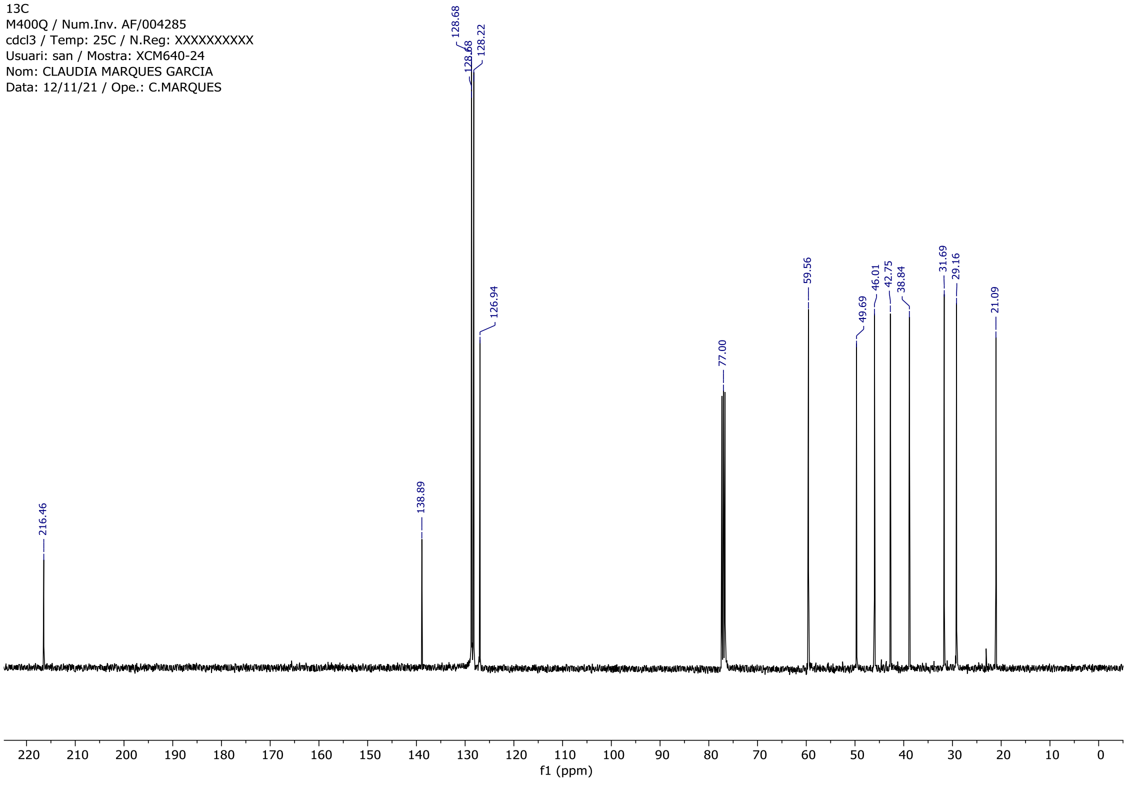
13C
M400Q / Num.Inv. AF/004285
cdcl3 / Temp: 25C / N.Reg: XXXXXXXXXX
Usuari: san / Mostra: XCM640-24
Nom: CLAUDIA MARQUES GARCIA
Data: 12/11/21 / Ope.: C.MARQUES
f1 (ppm)

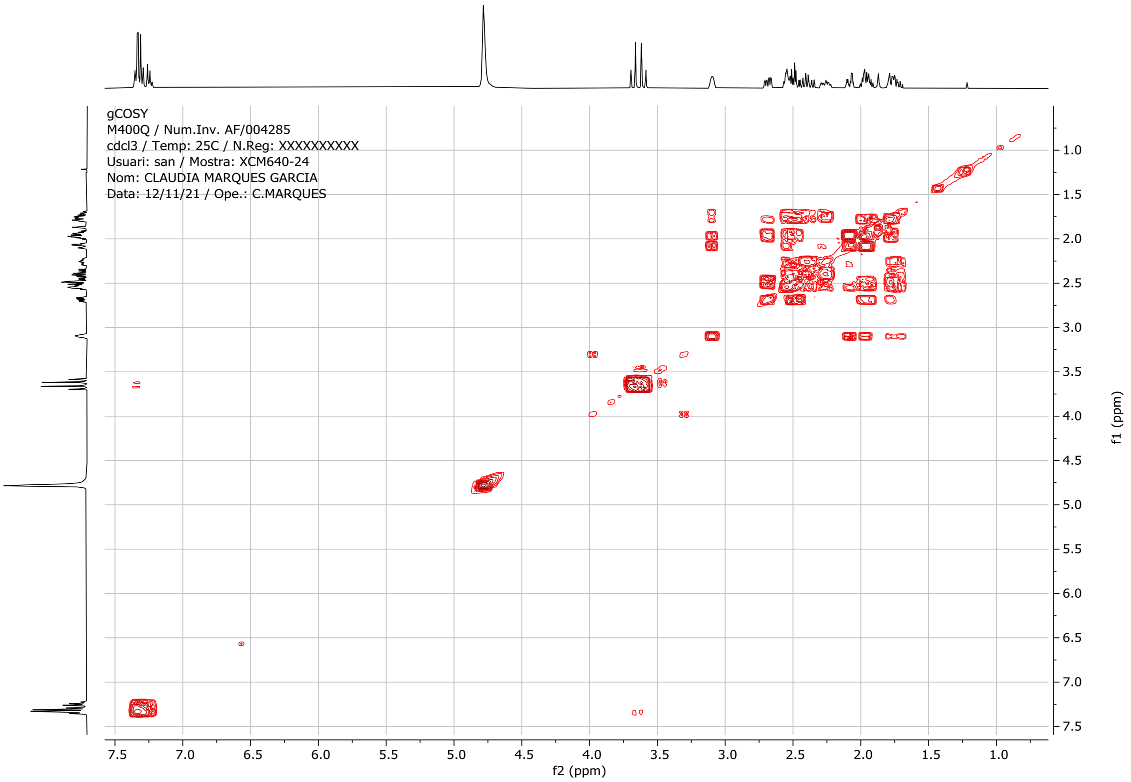
gCOSY
M400Q / Num.Inv. AF/004285
cdcl3 / Temp: 25C / N.Reg: XXXXXXXXXX
Usuari: san / Mostra: XCM640-24
Nom: CLAUDIA MARQUES GARCIA
Data: 12/11/21 / Ope.: C.MARQUES
f2 (ppm)
f1 (ppm)

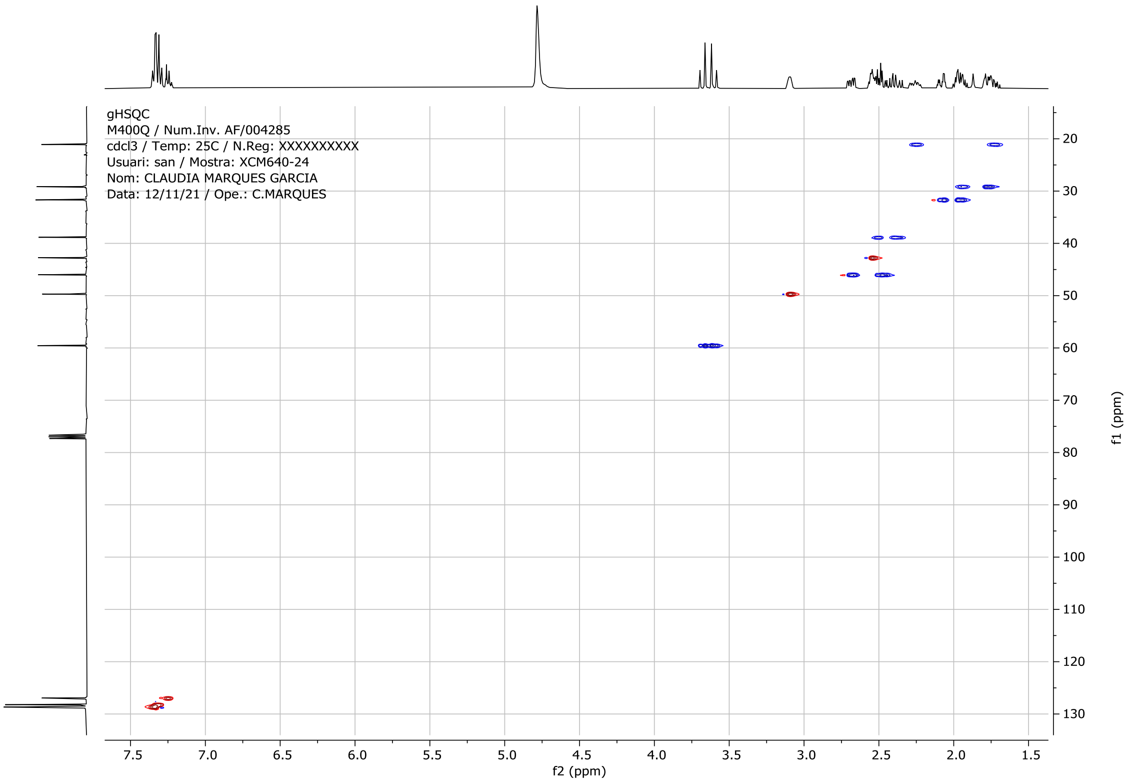
gHSQC
M400Q / Num.Inv. AF/004285
cdcl3 / Temp: 25C / N.Reg: XXXXXXXXXX
Usuari: san / Mostra: XCM640-24
Nom: CLAUDIA MARQUES GARCIA
Data: 12/11/21 / Ope.: C.MARQUES
f2 (ppm)
f1 (ppm)

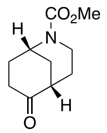
CO2Me
H
N
H
O

5c

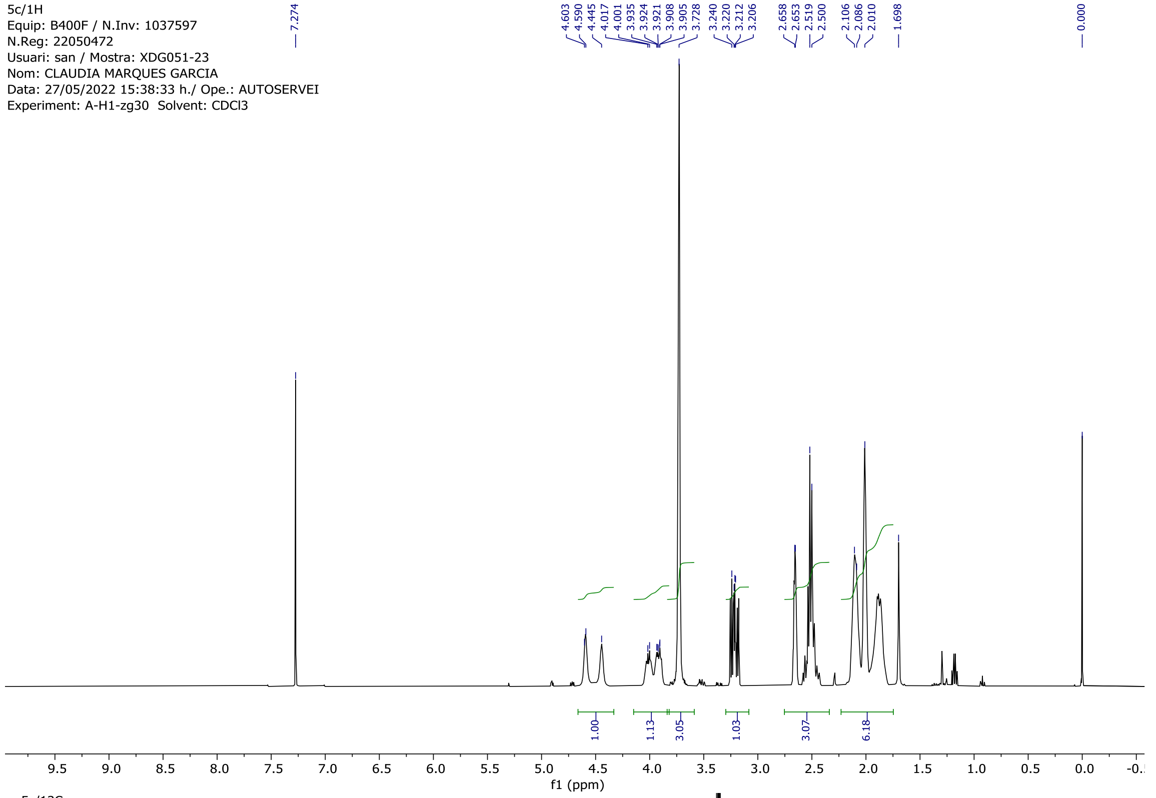
5c/1H
Equip: B400F / N.Inv: 1037597
N.Reg: 22050472
Usuari: san / Mostra: XDG051-23
Nom: CLAUDIA MARQUES GARCIA
Data: 27/05/2022 15:38:33 h./ Ope.: AUTOSERVEI
Experiment: A-H1-zg30 Solvent: CDCl3
7.274
4.603 4.590 4.445 4.017 4.001 3.935 3.924 3.921 3.908 3.905 3.728 3.240 3.220 3.212 3.206 2.658 2.653 2.519 2.500 2.106 2.086 2.010 1.698
0.000
1.00 1.13 3.05 1.03 3.07 6.18
f1 (ppm)

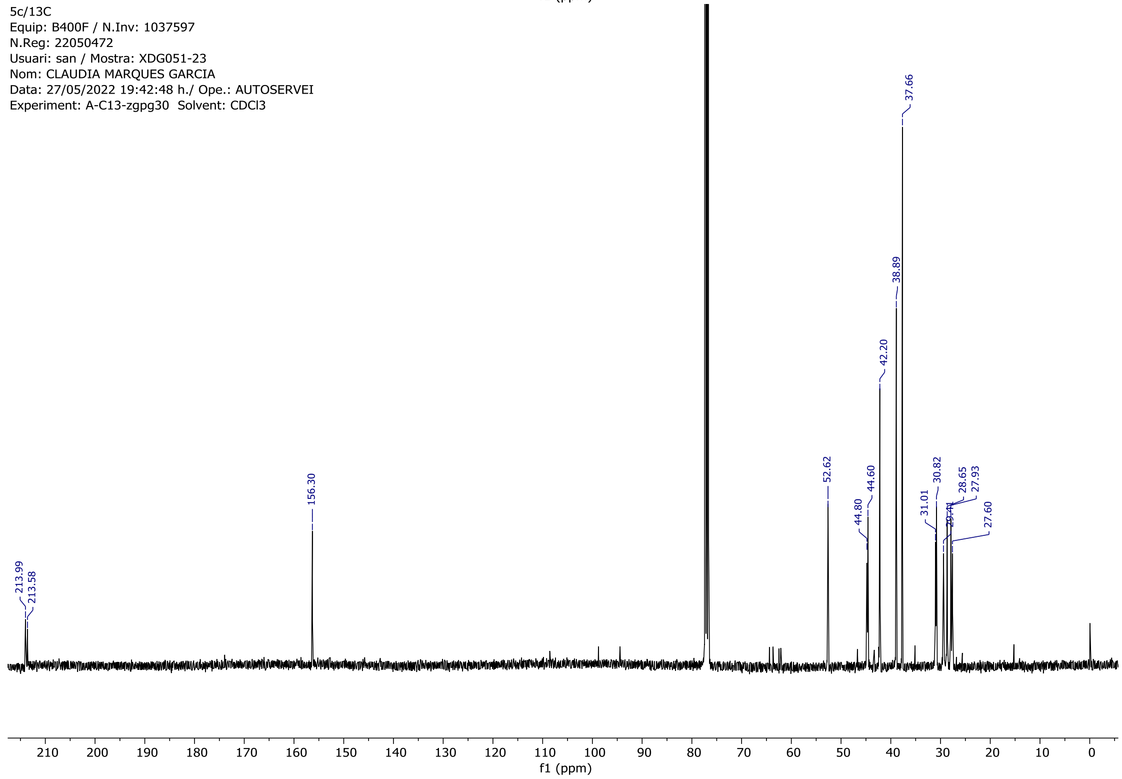
5c/13C
Equip: B400F / N.Inv: 1037597
N.Reg: 22050472
Usuari: san / Mostra: XDG051-23
Nom: CLAUDIA MARQUES GARCIA
Data: 27/05/2022 19:42:48 h./ Ope.: AUTOSERVEI
Experiment: A-C13-zgpg30 Solvent: CDCl3
213.99 213.58
156.30
52.62
44.80 44.60
42.20
38.89
37.66
31.01 30.82
28.65 27.93
27.60
f1 (ppm)

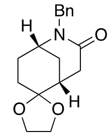

6

1H
M400Q / Num.Inv. AF/004285
cdcl3 / Temp: 25C / N.Reg: XXXXXXXXXX
Usuari: san / Mostra: XCM641-27
Nom: CLAUDIA MARQUES GARCIA
Data: 12/11/21 / Ope.: C.MARQUES

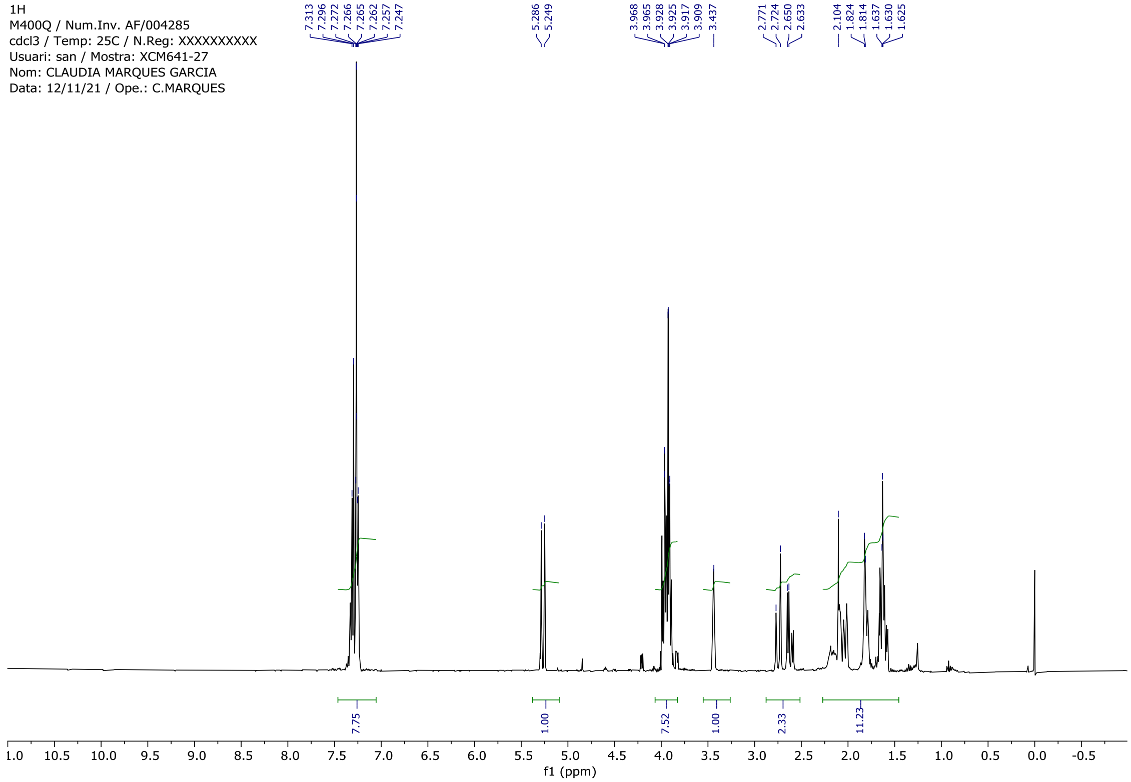

13C
M400Q / Num.Inv. AF/004285
cdcl3 / Temp: 25C / N.Reg: XXXXXXXXXX
Usuari: san / Mostra: XCM641-27
Nom: CLAUDIA MARQUES GARCIA
Data: 12/11/21 / Ope.: C.MARQUES

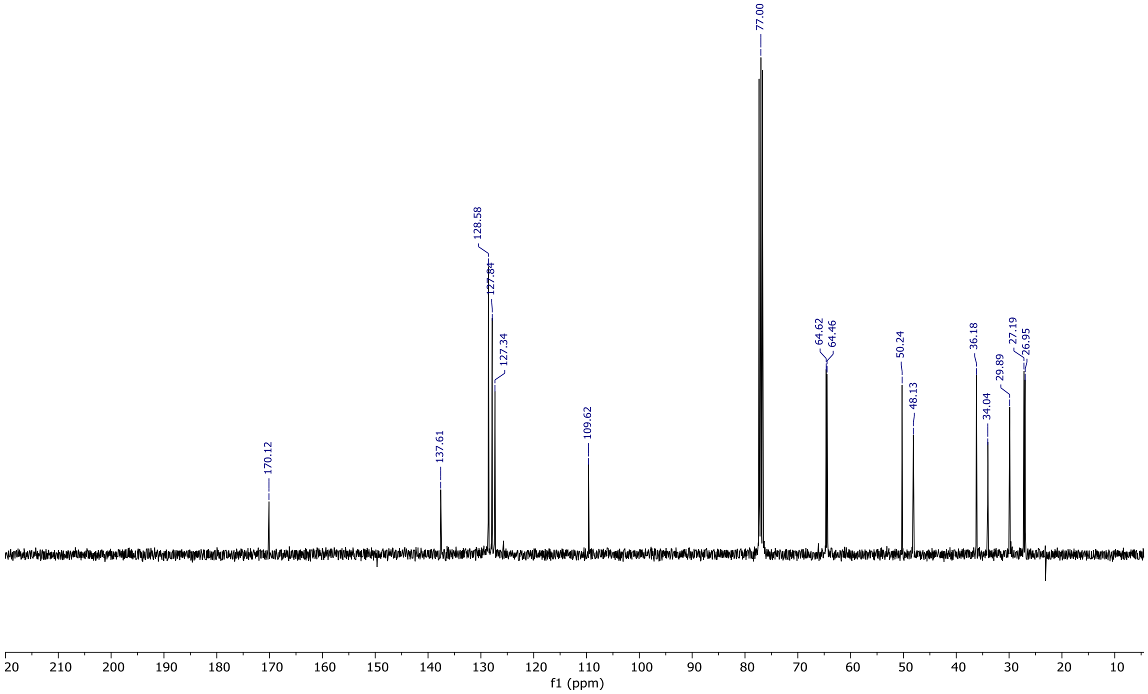

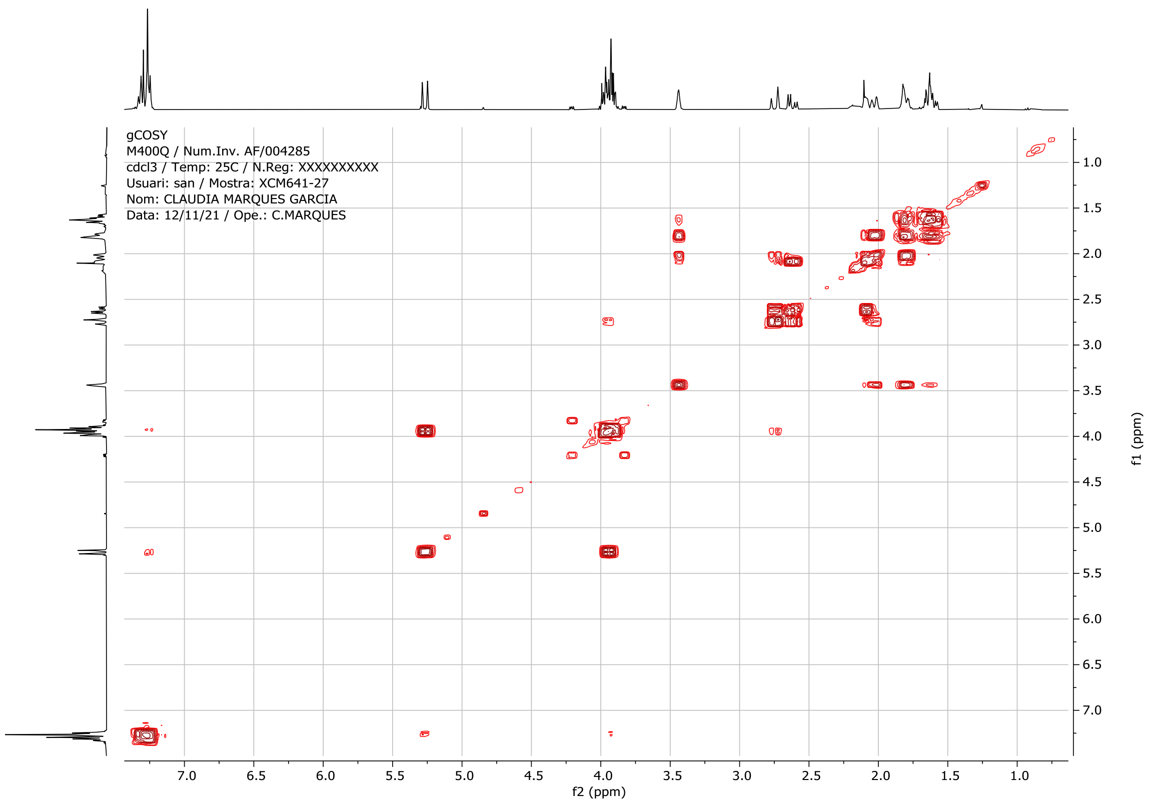
gCOSY
M400Q / Num.Inv. AF/004285
cdcl3 / Temp: 25C / N.Reg: XXXXXXXXXX
Usuari: san / Mostra: XCM641-27
Nom: CLAUDIA MARQUES GARCIA
Data: 12/11/21 / Ope.: C.MARQUES
f2 (ppm)
f1 (ppm)

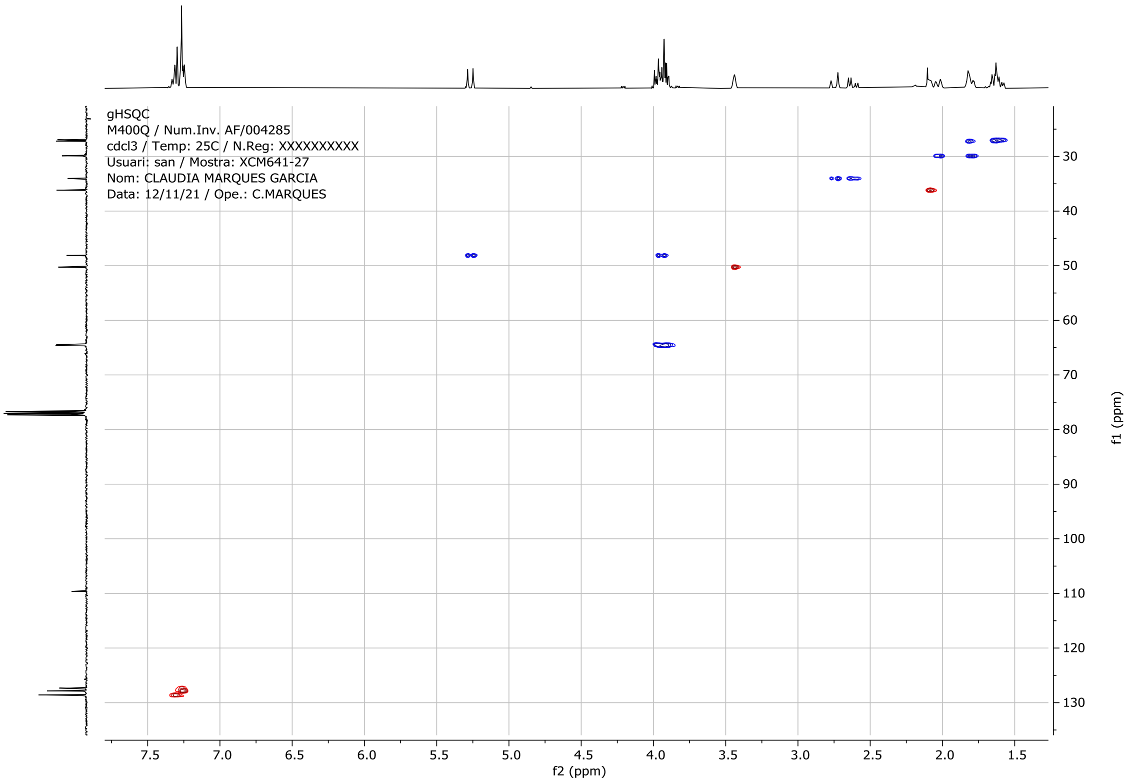
gHSQC
M400Q / Num.Inv. AF/004285
cdcl3 / Temp: 25C / N.Reg: XXXXXXXXXX
Usuari: san / Mostra: XCM641-27
Nom: CLAUDIA MARQUES GARCIA
Data: 12/11/21 / Ope.: C.MARQUES
f2 (ppm)
f1 (ppm)

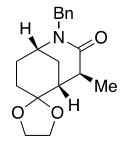

7

1H
M400Q / Num.Inv. AF/004285
cdcl3 / Temp: 25C / N.Reg: XXXXXXXXXX
Usuari: san / Mostra: CM617-22
Nom: CLAUDIA MARQUES GARCIA
Data: 20/10/21 / Ope.: C.MARQUES

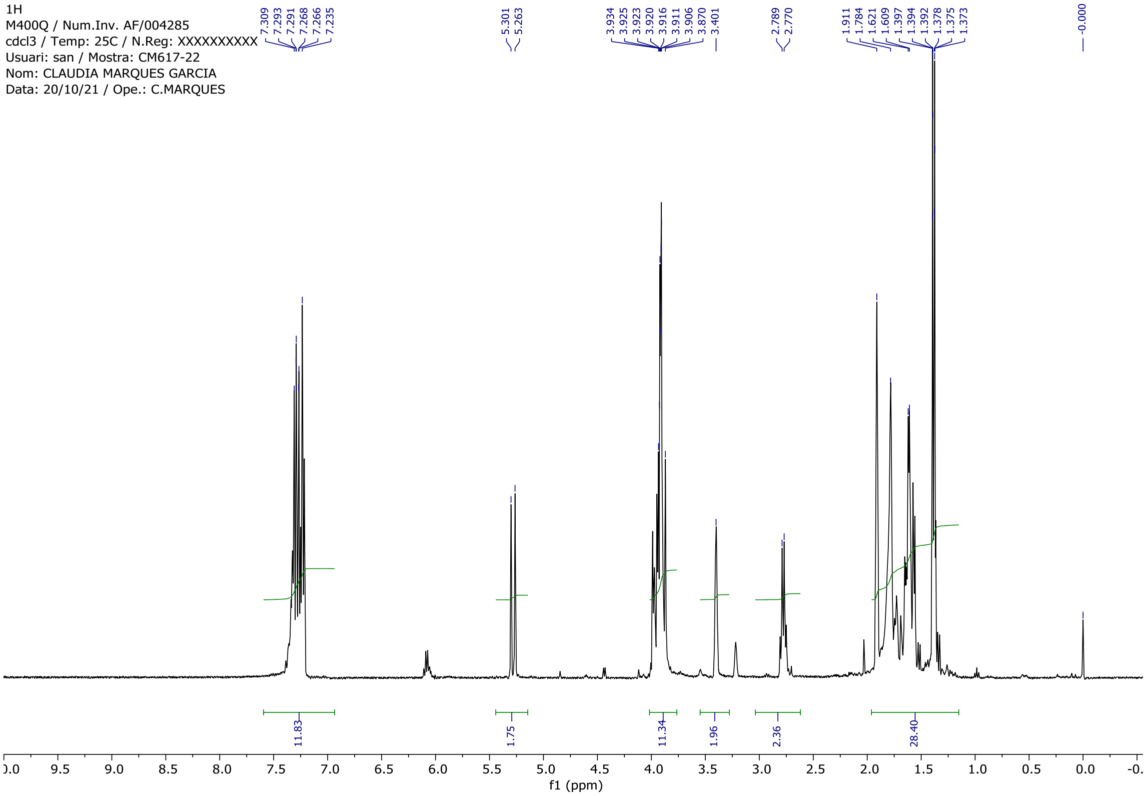

13C
M400Q / Num.Inv. AF/004285
cdcl3 / Temp: 25C / N.Reg: XXXXXXXXXX
Usuari: san / Mostra: CM617-22
Nom: CLAUDIA MARQUES GARCIA
Data: 20/10/21 / Ope.: C.MARQUES

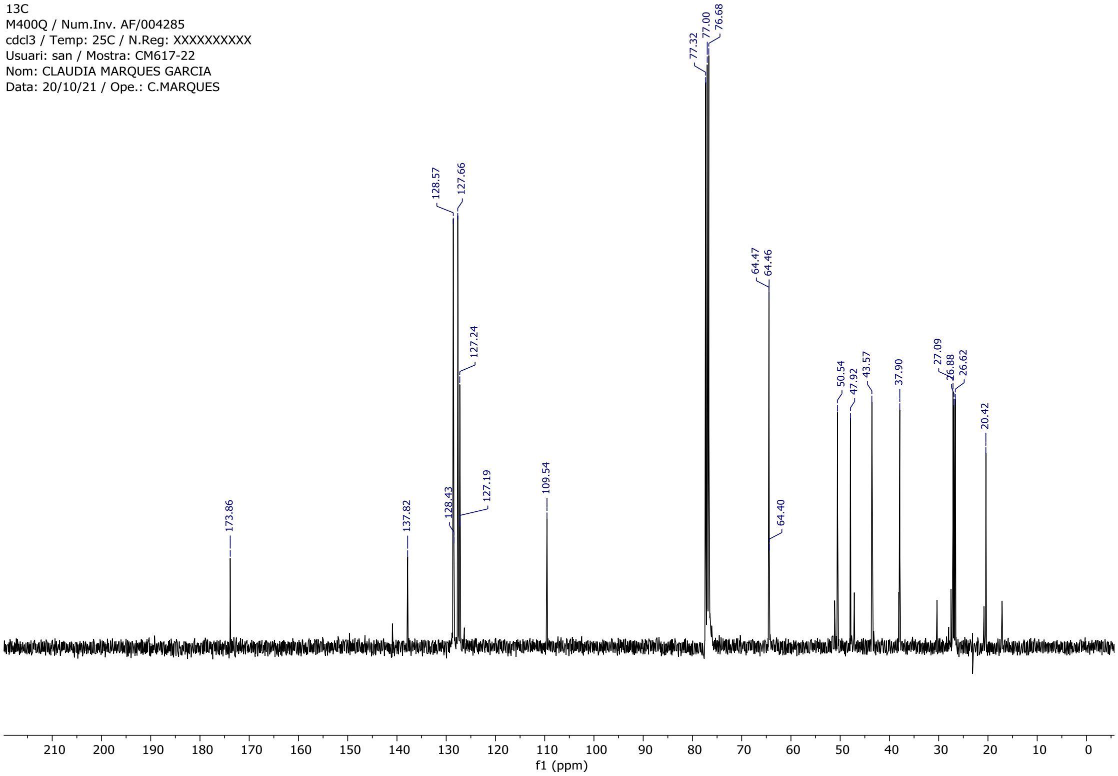

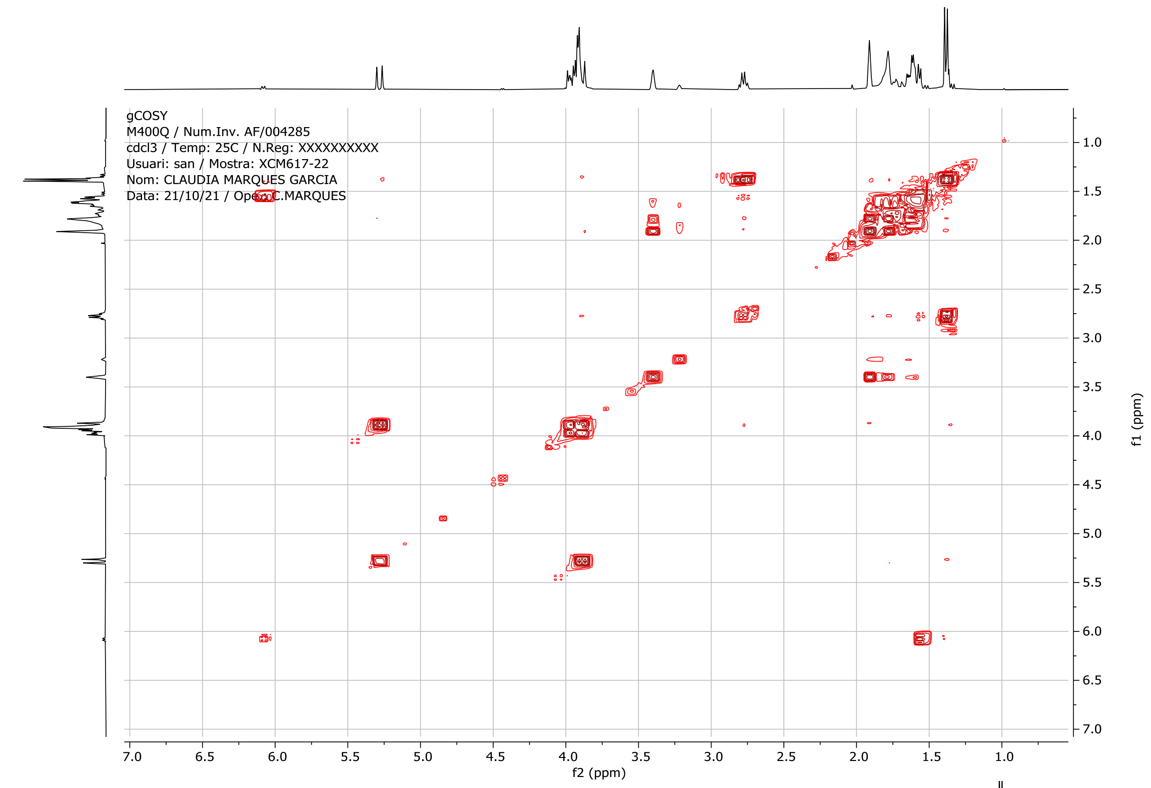
gCOSY
M400Q / Num.Inv. AF/004285
cdcl3 / Temp: 25C / N.Reg: XXXXXXXXXX
Usuari: san / Mostra: XCM617-22
Nom: CLAUDIA MARQUES GARCIA
Data: 21/10/21 / Ope.: C.MARQUES
f2 (ppm)
f1 (ppm)

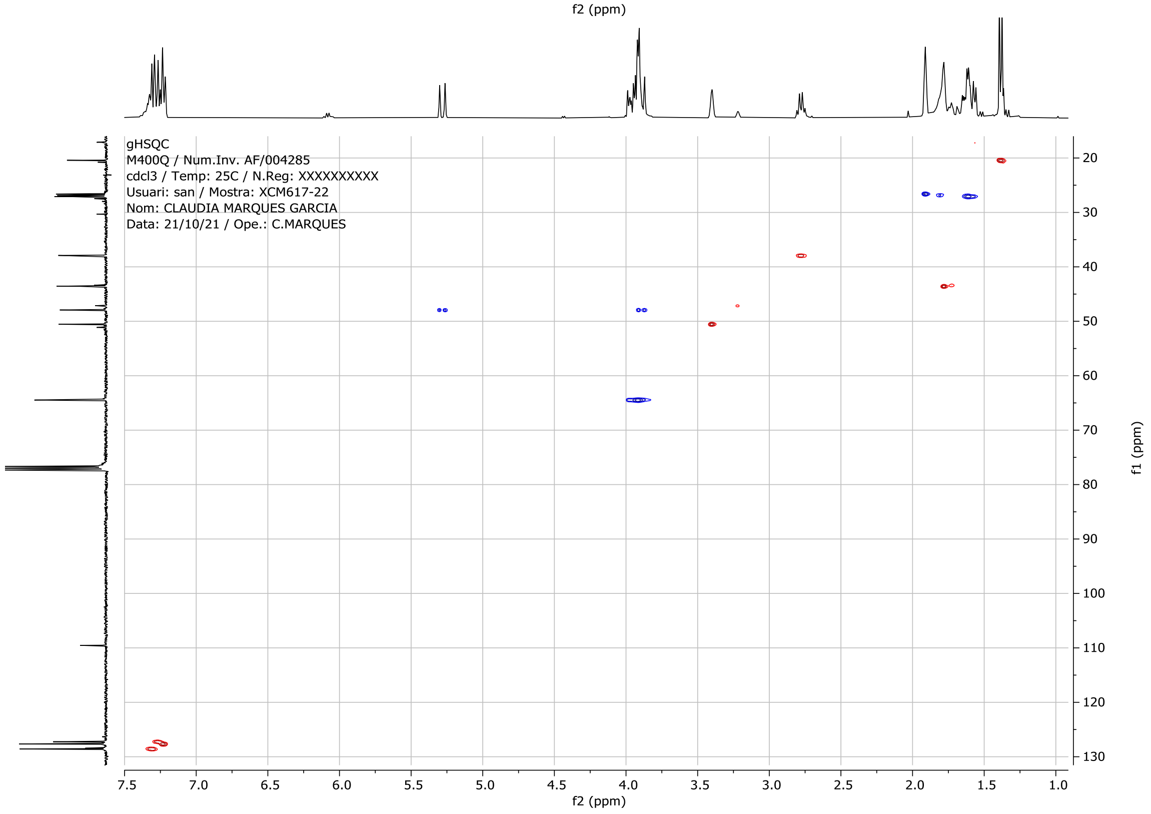
gHSQC
M400Q / Num.Inv. AF/004285
cdcl3 / Temp: 25C / N.Reg: XXXXXXXXXX
Usuari: san / Mostra: XCM617-22
Nom: CLAUDIA MARQUES GARCIA
Data: 21/10/21 / Ope.: C.MARQUES
f2 (ppm)
f1 (ppm)

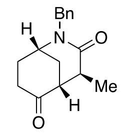

8a

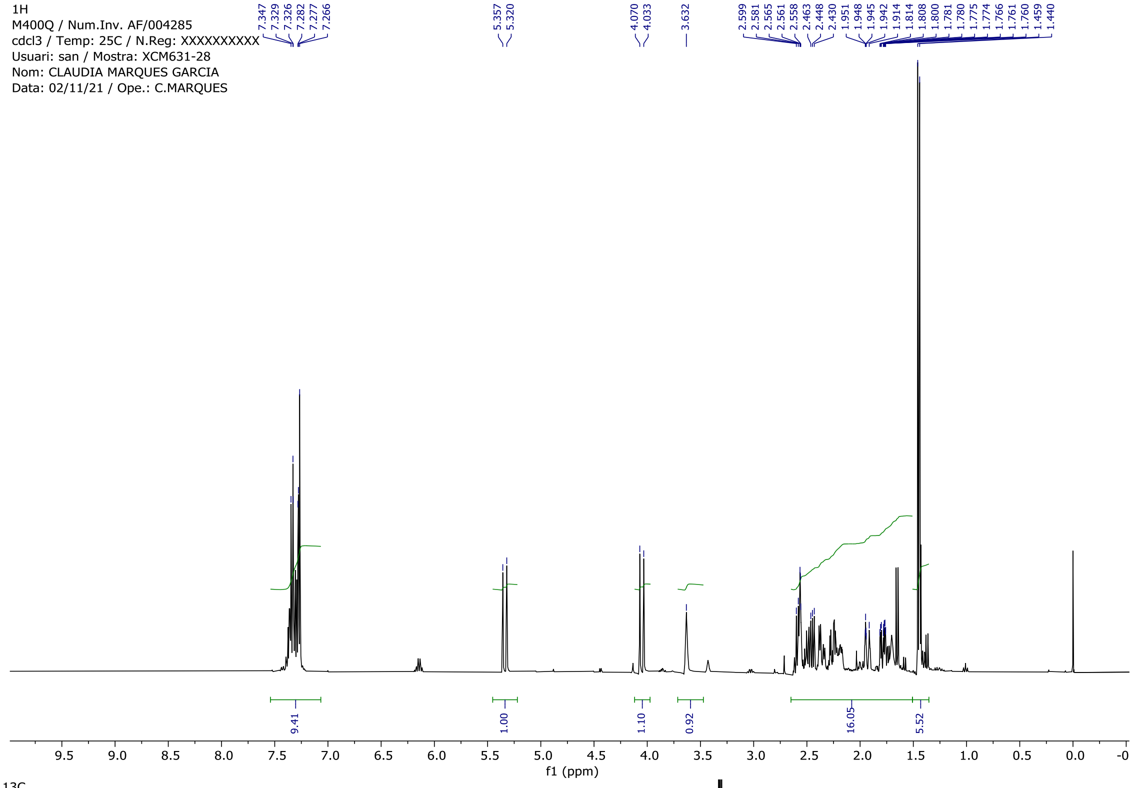

1H
M400Q / Num.Inv. AF/004285
cdcl3 / Temp: 25C / N.Reg: XXXXXXXXXX
Usuari: san / Mostra: XCM631-28
Nom: CLAUDIA MARQUES GARCIA
Data: 02/11/21 / Ope.: C.MARQUES
7.347 7.329 7.326 7.282 7.277 7.266
5.357 5.320
4.070 4.033
3.632
2.599 2.581 2.565 2.561 2.558 2.463 2.448 2.430 1.951 1.948 1.945 1.942 1.914 1.814 1.808 1.800 1.781 1.780 1.775 1.774 1.766 1.761 1.760 1.459 1.440
9.41
1.00
1.10
0.92
16.05
5.52
f1 (ppm)

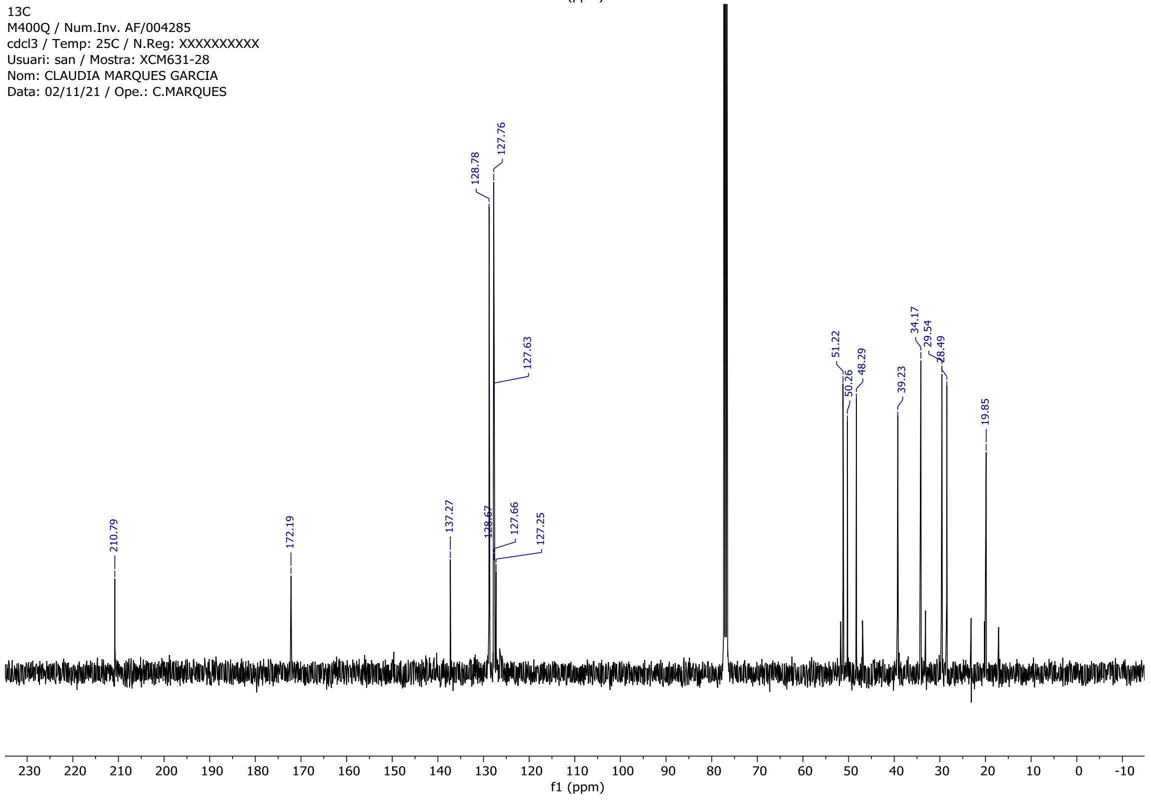

13C
M400Q / Num.Inv. AF/004285
cdcl3 / Temp: 25C / N.Reg: XXXXXXXXXX
Usuari: san / Mostra: XCM631-28
Nom: CLAUDIA MARQUES GARCIA
Data: 02/11/21 / Ope.: C.MARQUES
210.79
172.19
137.27
128.78
127.76
127.63
128.67
127.66
127.25
51.22
50.26
48.29
39.23
34.17
29.54
28.49
19.85
f1 (ppm)

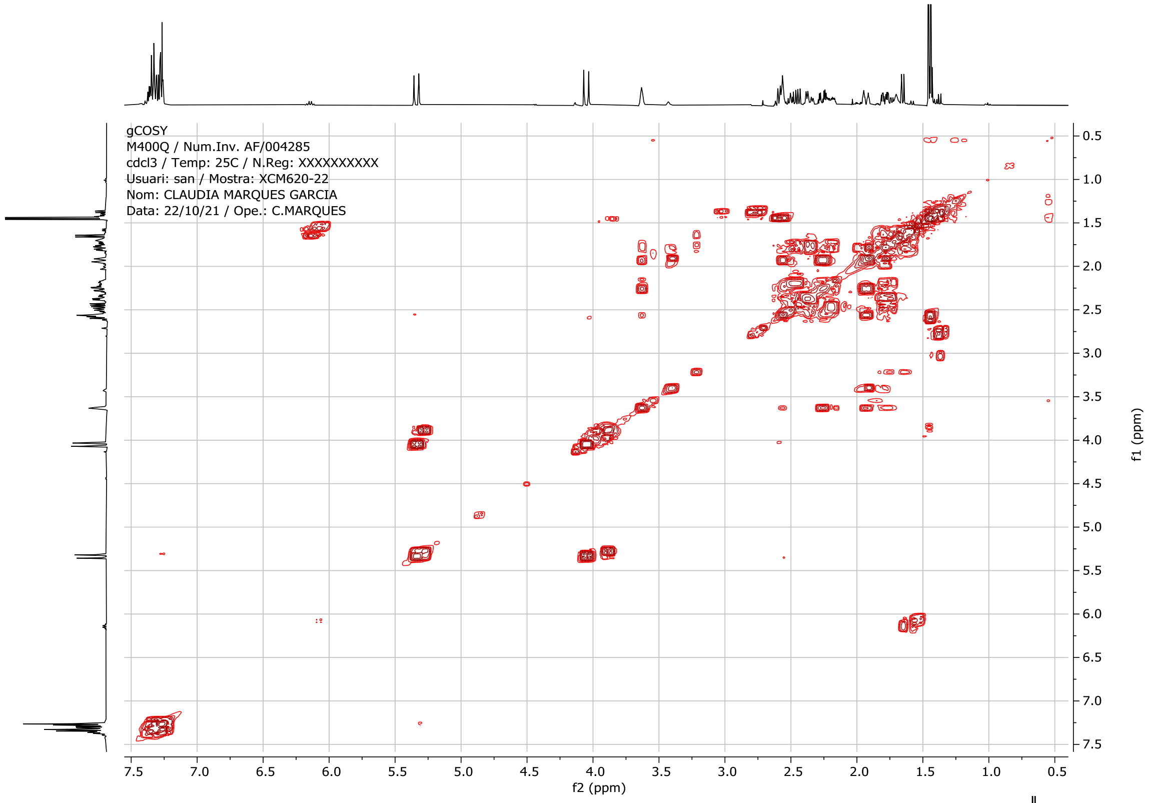
gCOSY
M400Q / Num.Inv. AF/004285
cdcl3 / Temp: 25C / N.Reg: XXXXXXXXXX
Usuari: san / Mostra: XCM620-22
Nom: CLAUDIA MARQUES GARCIA
Data: 22/10/21 / Ope.: C.MARQUES
f2 (ppm)
f1 (ppm)

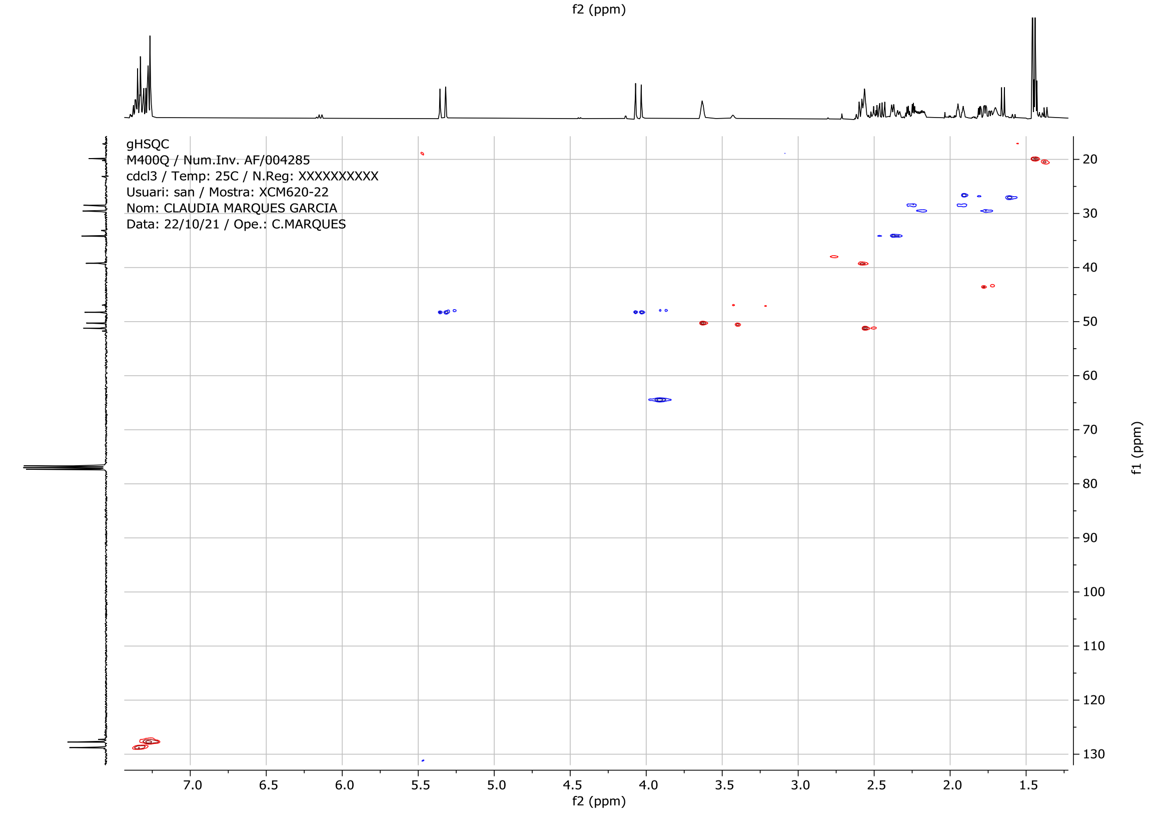
gHSQC
M400Q / Num.Inv. AF/004285
cdcl3 / Temp: 25C / N.Reg: XXXXXXXXXX
Usuari: san / Mostra: XCM620-22
Nom: CLAUDIA MARQUES GARCIA
Data: 22/10/21 / Ope.: C.MARQUES
f2 (ppm)
f1 (ppm)

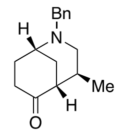

8b

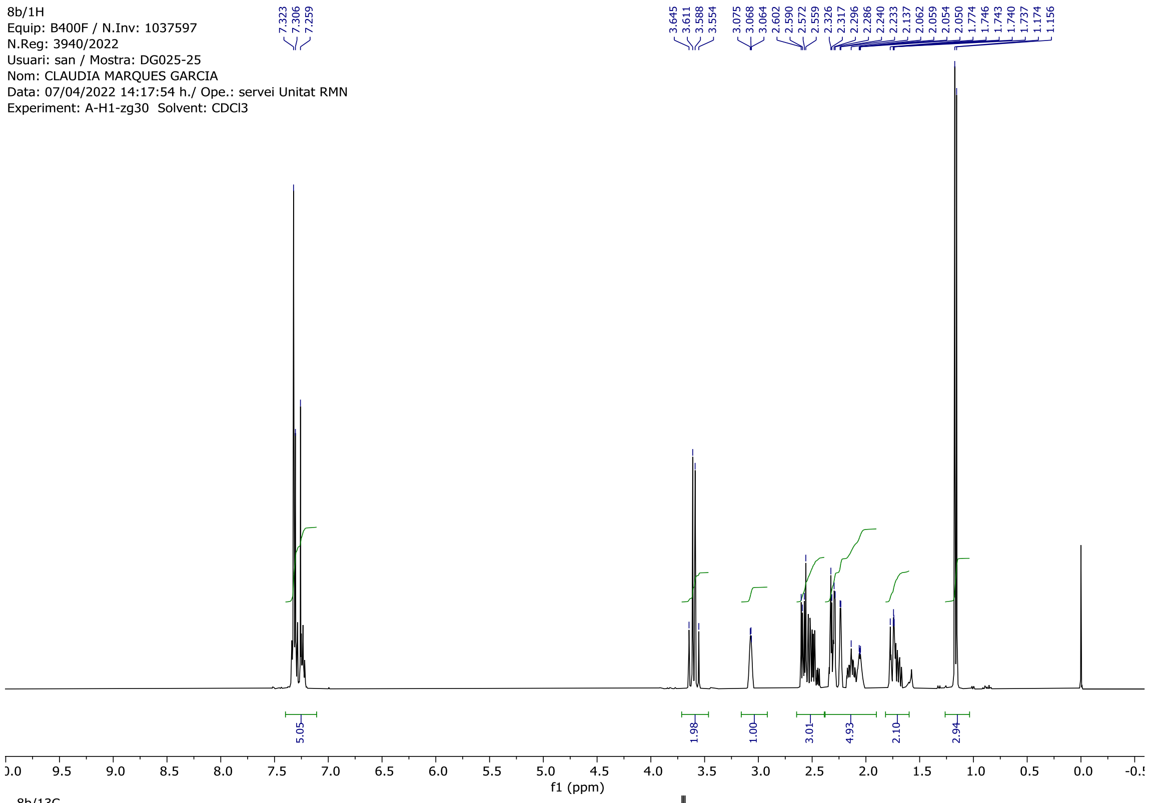
8b/1H
Equip: B400F / N.Inv: 1037597
N.Reg: 3940/2022
Usuari: san / Mostra: DG025-25
Nom: CLAUDIA MARQUES GARCIA
Data: 07/04/2022 14:17:54 h./ Ope.: servei Unitat RMN
Experiment: A-H1-zg30 Solvent: CDCl3
7.323
7.306
7.259
3.645
3.611
3.588
3.554
3.075
3.068
3.064
2.602
2.590
2.572
2.559
2.326
2.317
2.296
2.286
2.240
2.233
2.137
2.062
2.059
2.054
2.050
1.774
1.746
1.743
1.740
1.737
1.174
1.156
5.05
1.98
1.00
3.01
4.93
2.10
2.94
f1 (ppm)

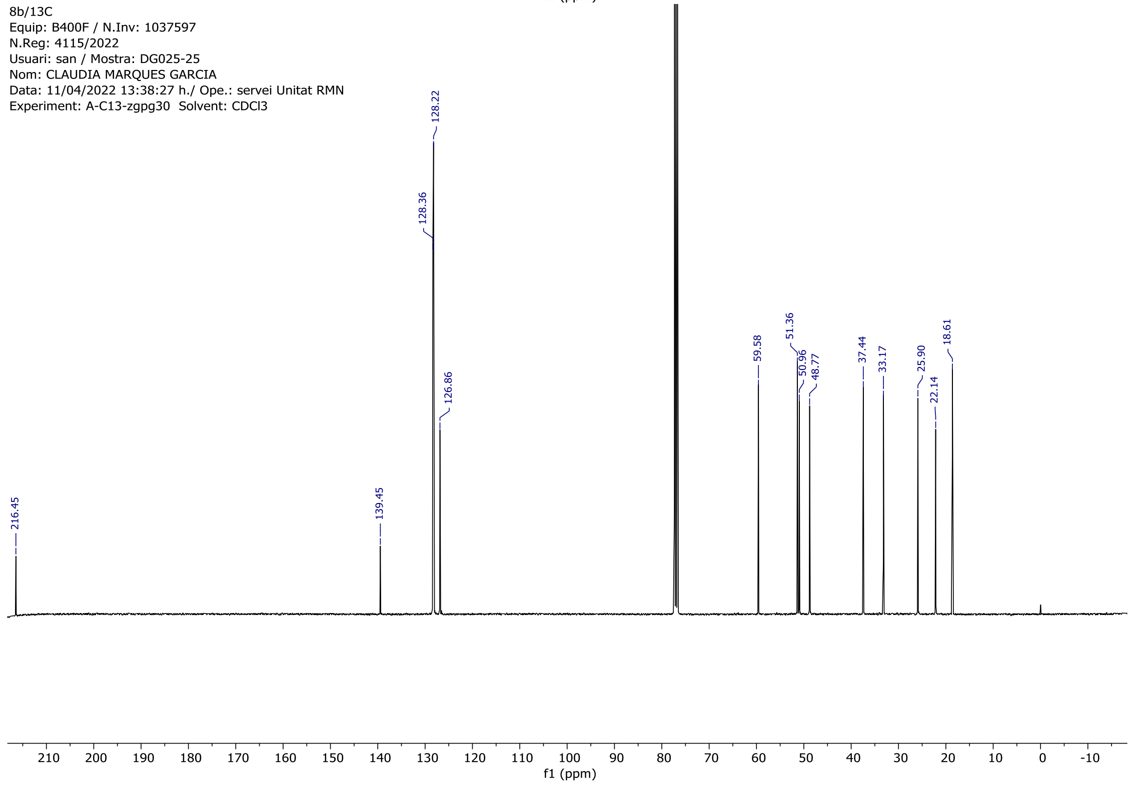
8b/13C
Equip: B400F / N.Inv: 1037597
N.Reg: 4115/2022
Usuari: san / Mostra: DG025-25
Nom: CLAUDIA MARQUES GARCIA
Data: 11/04/2022 13:38:27 h./ Ope.: servei Unitat RMN
Experiment: A-C13-zgpg30 Solvent: CDCl3
216.45
139.45
128.36
128.22
126.86
59.58
51.36
50.96
48.77
37.44
33.17
25.90
22.14
18.61
f1 (ppm)

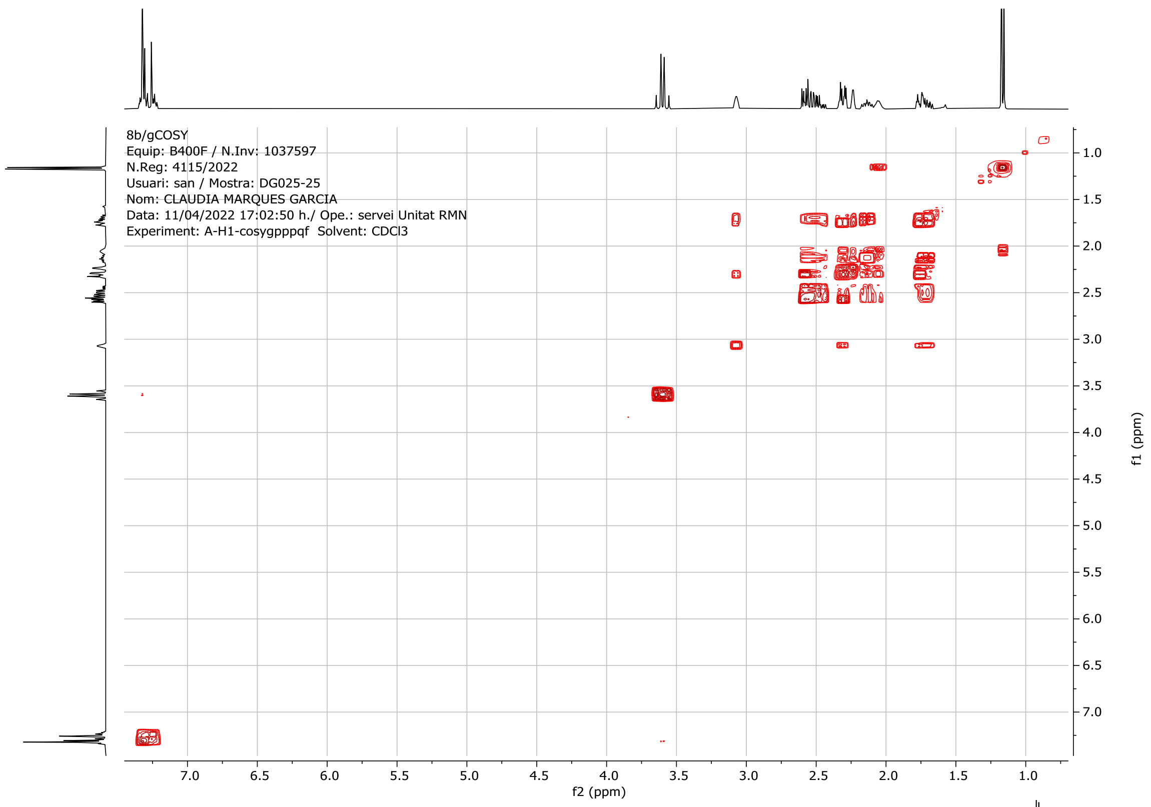
8b/gCOSY
Equip: B400F / N.Inv: 1037597
N.Reg: 4115/2022
Usuari: san / Mostra: DG025-25
Nom: CLAUDIA MARQUES GARCIA
Data: 11/04/2022 17:02:50 h./ Ope.: servei Unitat RMN
Experiment: A-H1-cosygpppqf Solvent: CDCl3
f2 (ppm)
f1 (ppm)

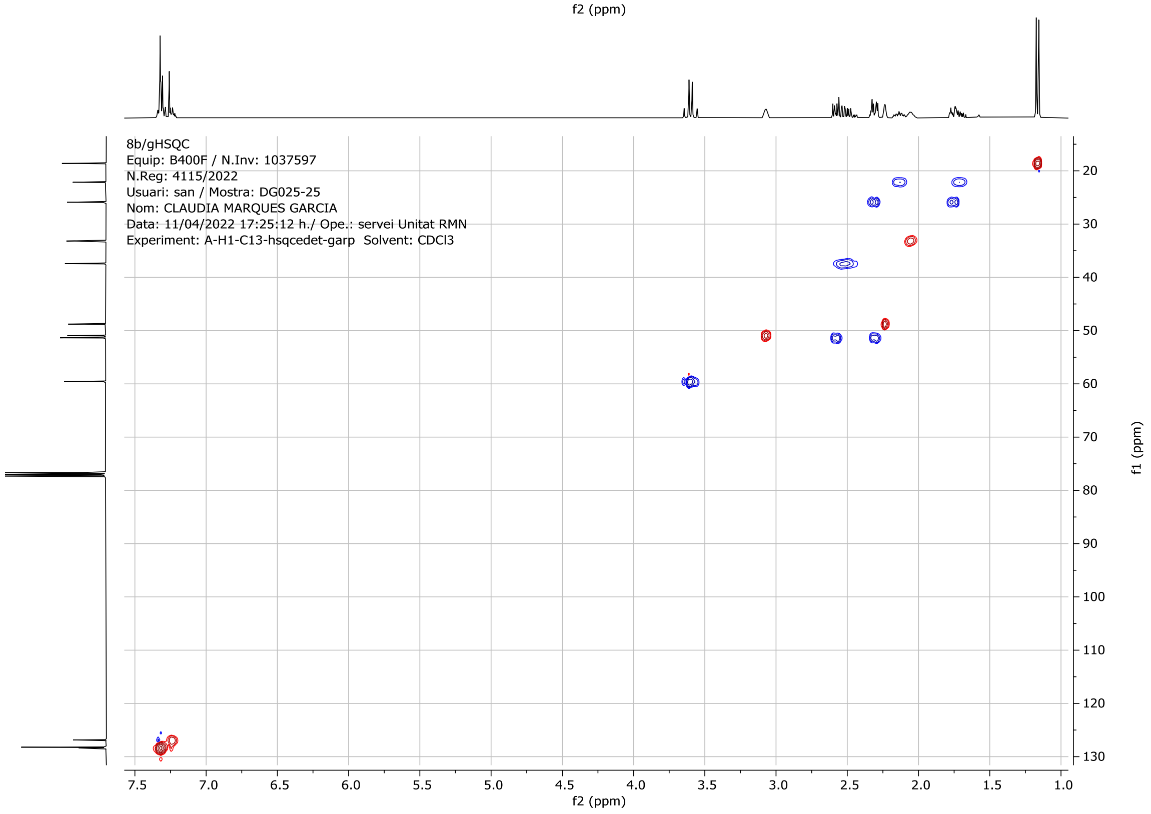
8b/gHSQC
Equip: B400F / N.Inv: 1037597
N.Reg: 4115/2022
Usuari: san / Mostra: DG025-25
Nom: CLAUDIA MARQUES GARCIA
Data: 11/04/2022 17:25:12 h./ Ope.: servei Unitat RMN
Experiment: A-H1-C13-hsqcedet-garp Solvent: CDCl3
f2 (ppm)
f1 (ppm)

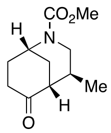
H
CO2Me
N
Me
H
O

8c

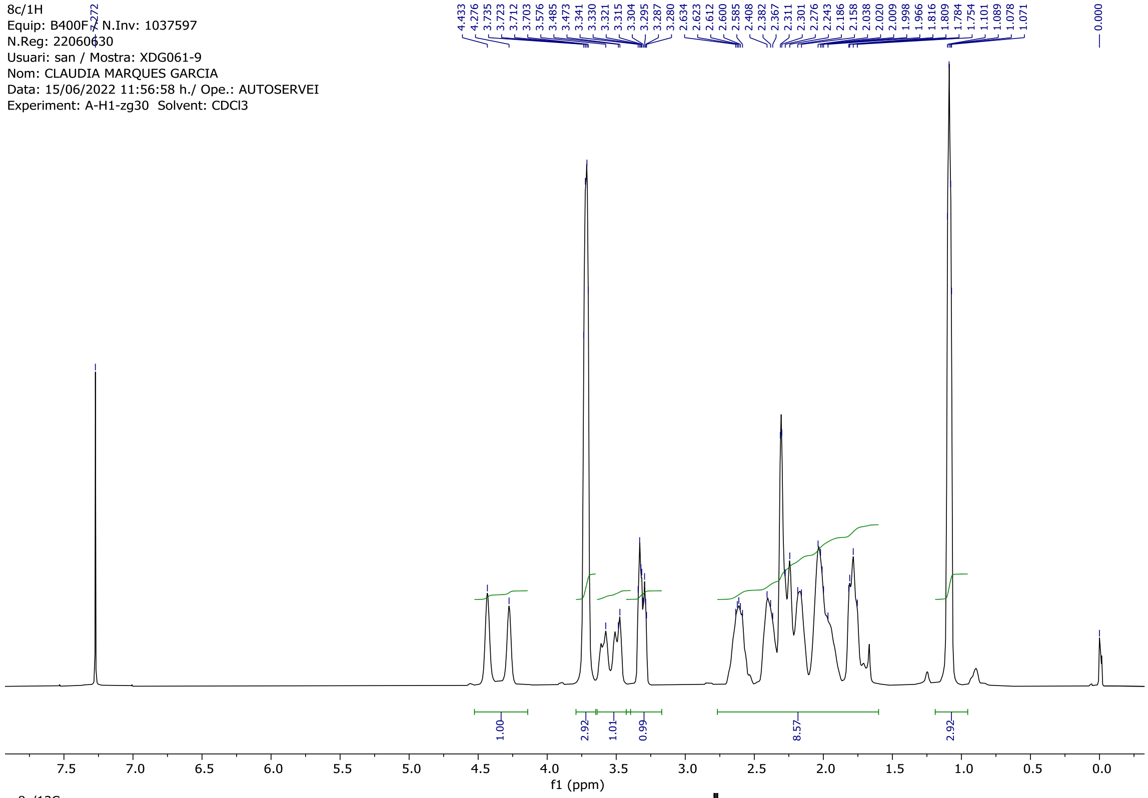
8c/1H
Equip: B400F / N.Inv: 1037597
N.Reg: 22060630
Usuari: san / Mostra: XDG061-9
Nom: CLAUDIA MARQUES GARCIA
Data: 15/06/2022 11:56:58 h./ Ope.: AUTOSERVEI
Experiment: A-H1-zg30 Solvent: CDCl3
7.272
4.433 4.276 3.735 3.723 3.712 3.703 3.576 3.485 3.473 3.341 3.330 3.321 3.315 3.304 3.295 3.287 3.280 2.634 2.623 2.612 2.600 2.585 2.408 2.382 2.367 2.311 2.301 2.276 2.243 2.186 2.158 2.038 2.020 2.009 1.998 1.966 1.816 1.809 1.784 1.754 1.101 1.089 1.078 1.071
0.000
1.00
2.92
1.01
0.99
8.57
2.92
f1 (ppm)

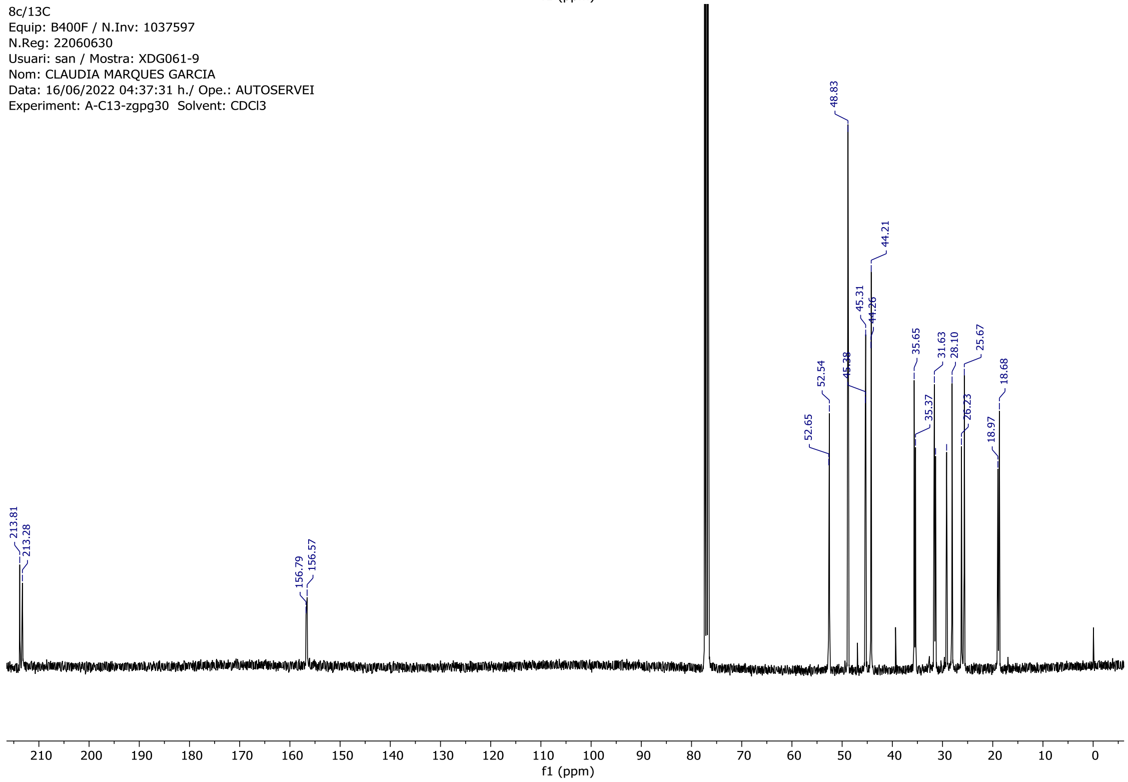
8c/13C
Equip: B400F / N.Inv: 1037597
N.Reg: 22060630
Usuari: san / Mostra: XDG061-9
Nom: CLAUDIA MARQUES GARCIA
Data: 16/06/2022 04:37:31 h./ Ope.: AUTOSERVEI
Experiment: A-C13-zgpg30 Solvent: CDCl3
213.81
213.28
156.79
156.57
52.65
52.54
48.83
45.38
45.31
44.26
44.21
35.65
35.37
31.63
28.10
26.23
25.67
18.97
18.68
f1 (ppm)

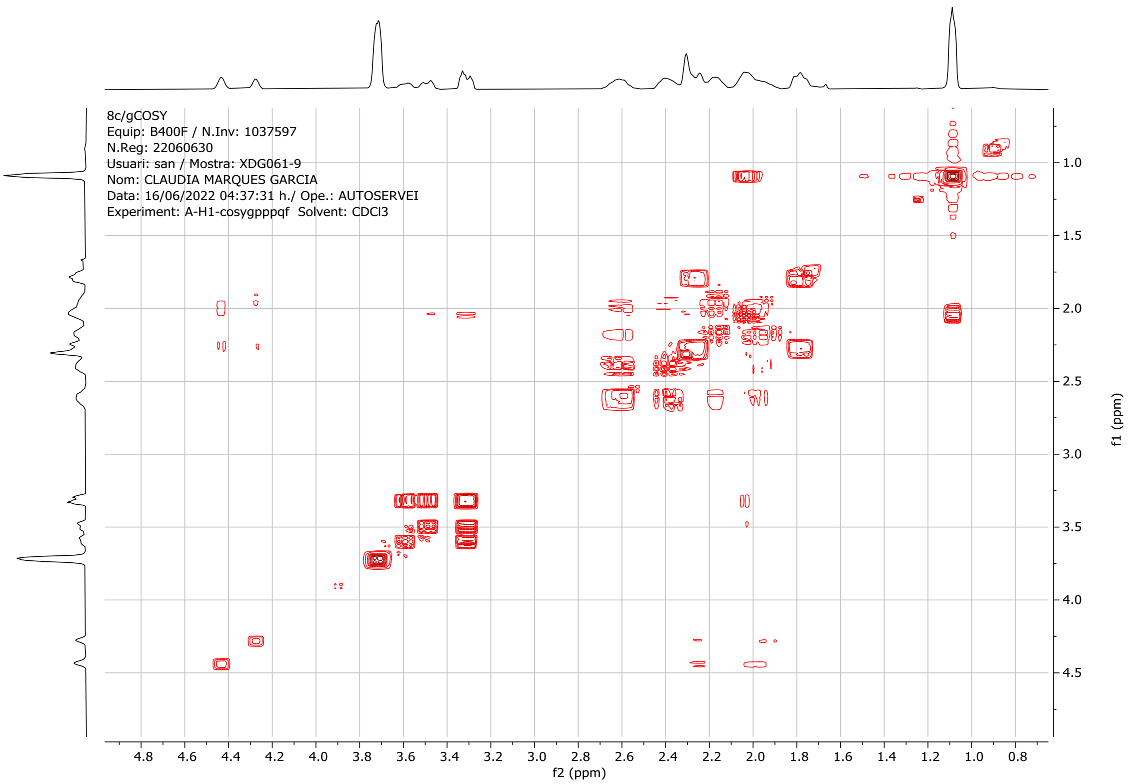
8c/gCOSY
Equip: B400F / N.Inv: 1037597
N.Reg: 22060630
Usuari: san / Mostra: XDG061-9
Nom: CLAUDIA MARQUES GARCIA
Data: 16/06/2022 04:37:31 h./ Ope.: AUTOSERVEI
Experiment: A-H1-cosygpppqf Solvent: CDCl3
f2 (ppm)
f1 (ppm)

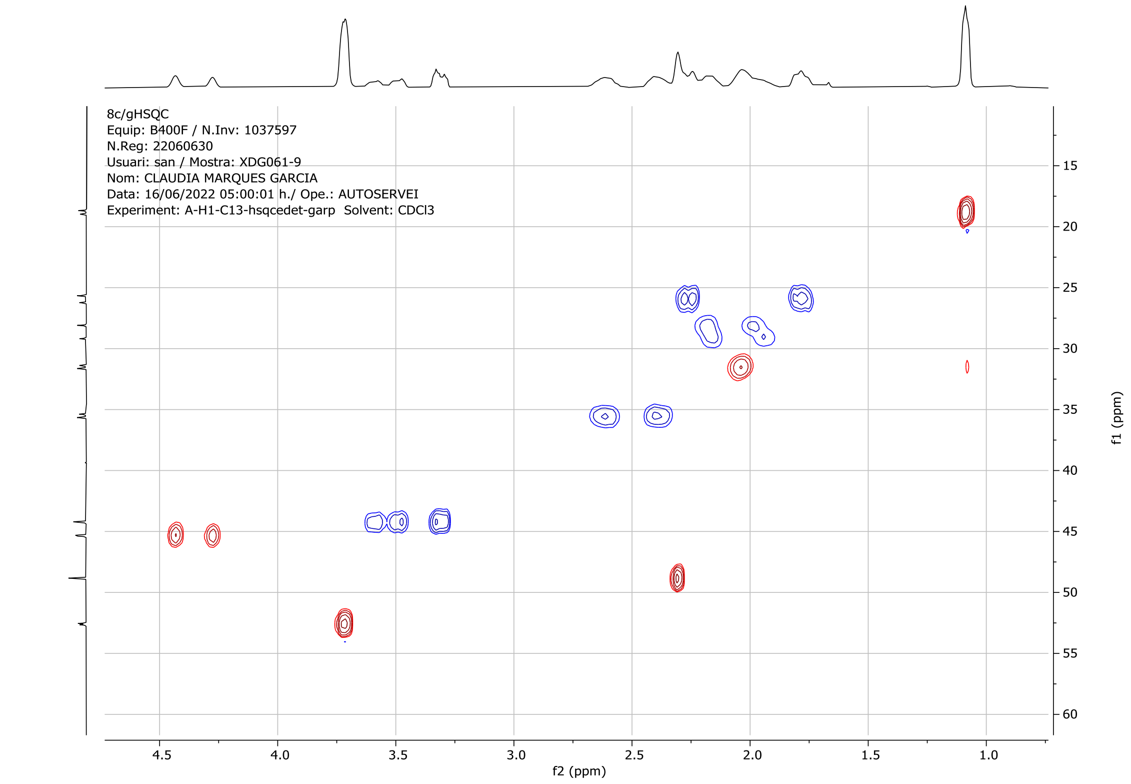
8c/gHSQC
Equip: B400F / N.Inv: 1037597
N.Reg: 22060630
Usuari: san / Mostra: XDG061-9
Nom: CLAUDIA MARQUES GARCIA
Data: 16/06/2022 05:00:01 h./ Ope.: AUTOSERVEI
Experiment: A-H1-C13-hsqcedet-garp Solvent: CDCl3
f2 (ppm)
f1 (ppm)

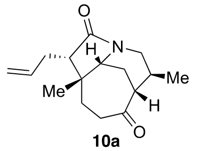

10a

10a/1H
Equip: B400F / N.Inv: 1037597
N.Reg: 22070312
Usuari: san / Mostra: XCM728-48
Nom: CLAUDIA MARQUES GARCIA
Data: 13/07/2022 12:29:05 h./ Ope.: AUTOSERVEI
Experiment: A-H1-zg30 Solvent: CDCl3

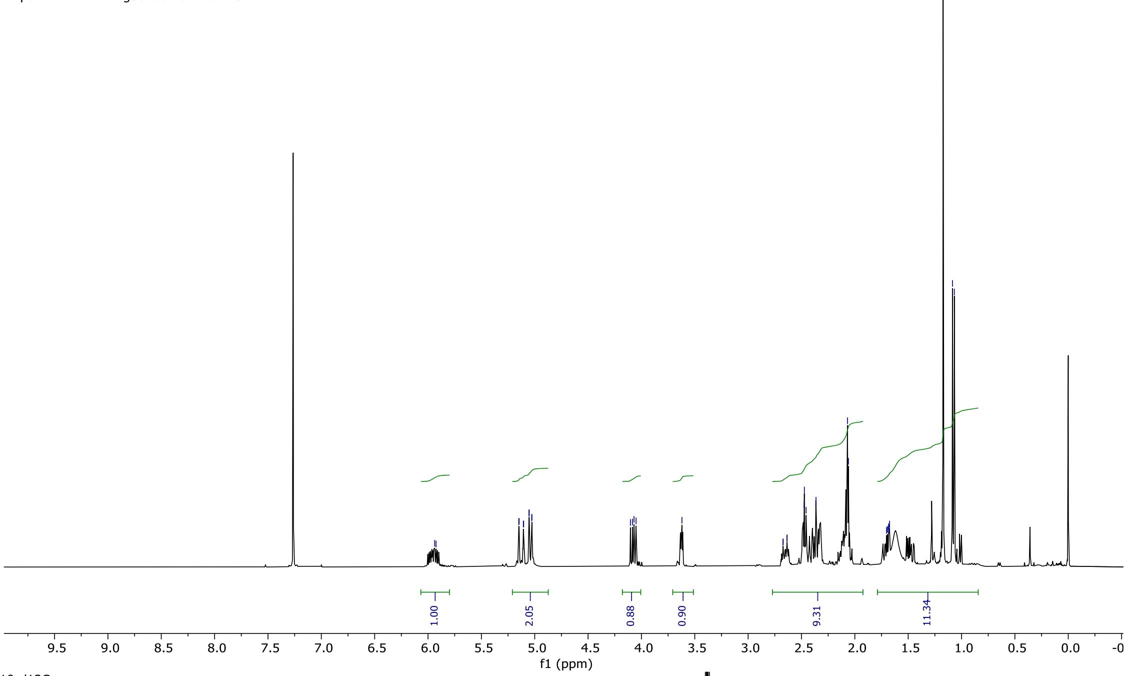

10a/13C
Equip: B400F / N.Inv: 1037597
N.Reg: 22070312
Usuari: san / Mostra: XCM728-48
Nom: CLAUDIA MARQUES GARCIA
Data: 14/07/2022 00:39:07 h./ Ope.: AUTOSERVEI
Experiment: A-C13-zgpg30 Solvent: CDCl3

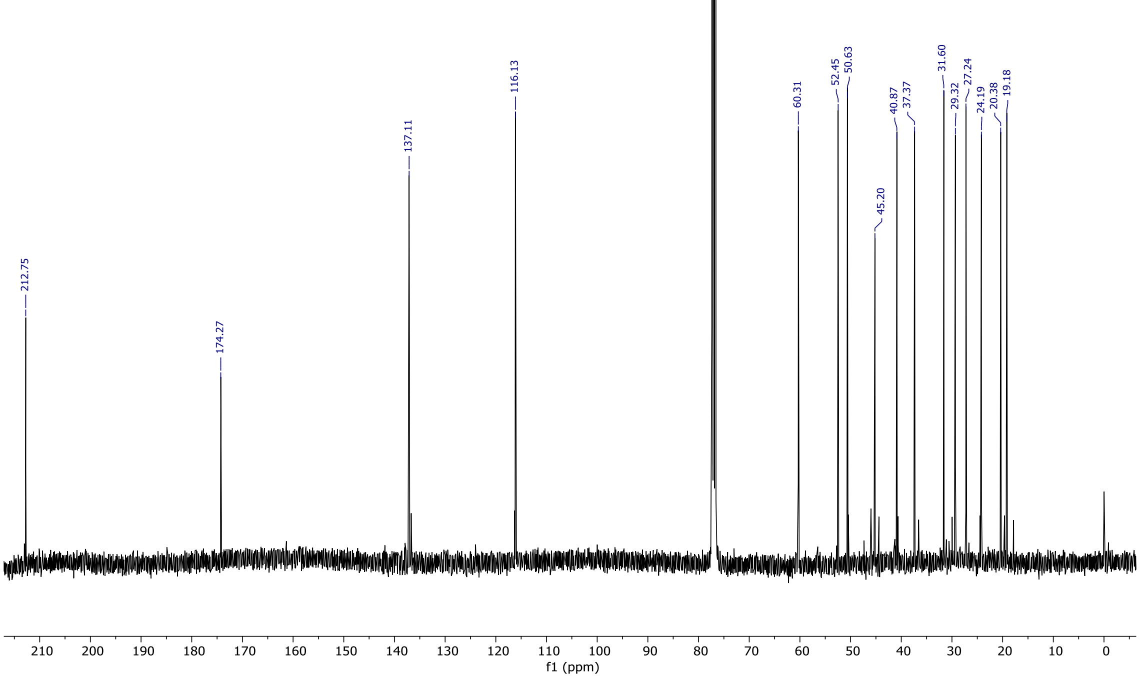

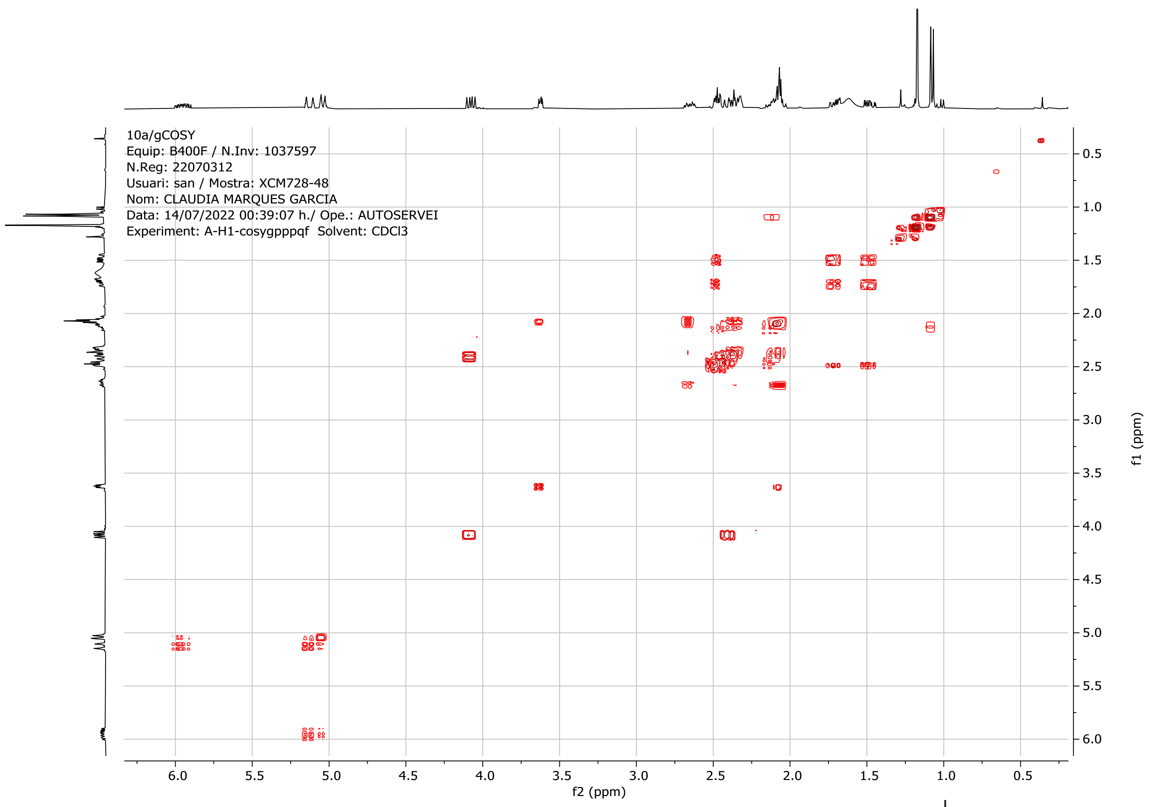
10a/gCOSY
Equip: B400F / N.Inv: 1037597
N.Reg: 22070312
Usuari: san / Mostra: XCM728-48
Nom: CLAUDIA MARQUES GARCIA
Data: 14/07/2022 00:39:07 h./ Ope.: AUTOSERVEI
Experiment: A-H1-cosygpppqf Solvent: CDCl3
f2 (ppm)
f1 (ppm)

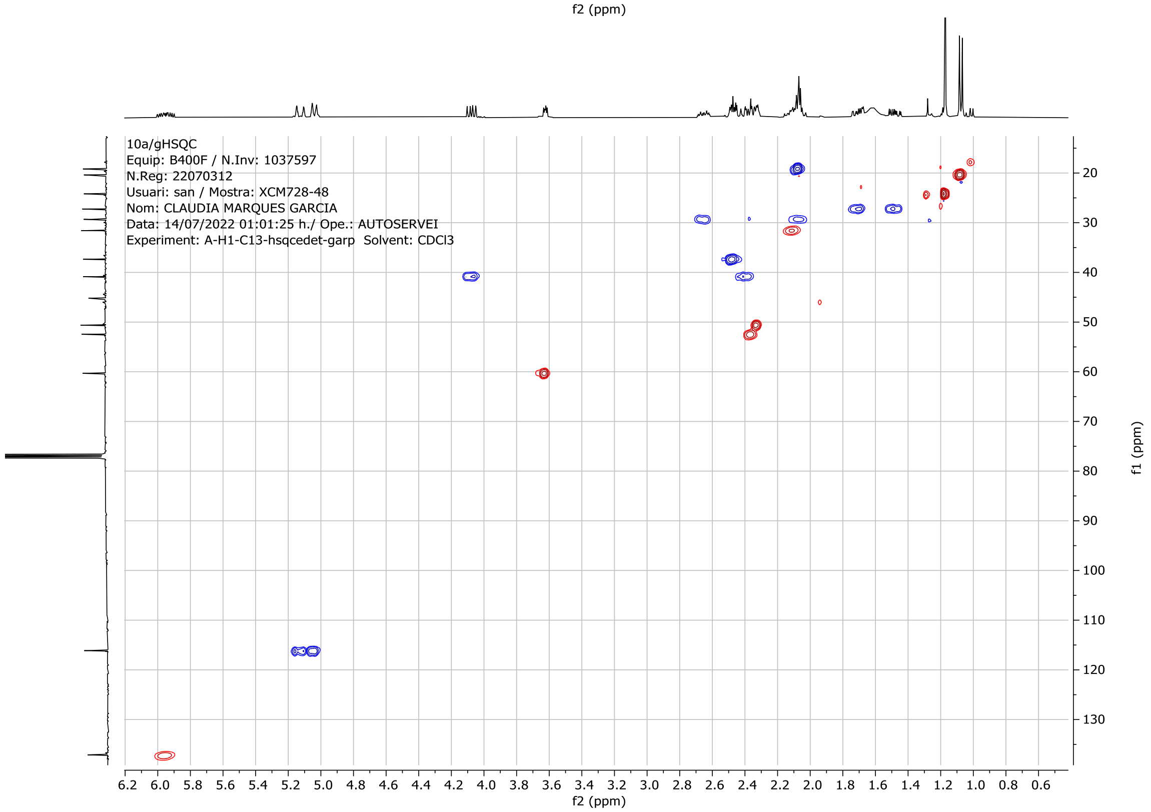
10a/gHSQC
Equip: B400F / N.Inv: 1037597
N.Reg: 22070312
Usuari: san / Mostra: XCM728-48
Nom: CLAUDIA MARQUES GARCIA
Data: 14/07/2022 01:01:25 h./ Ope.: AUTOSERVEI
Experiment: A-H1-C13-hsqcedet-garp Solvent: CDCl3
f2 (ppm)
f1 (ppm)

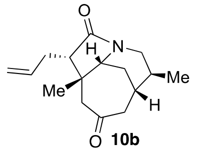

10b

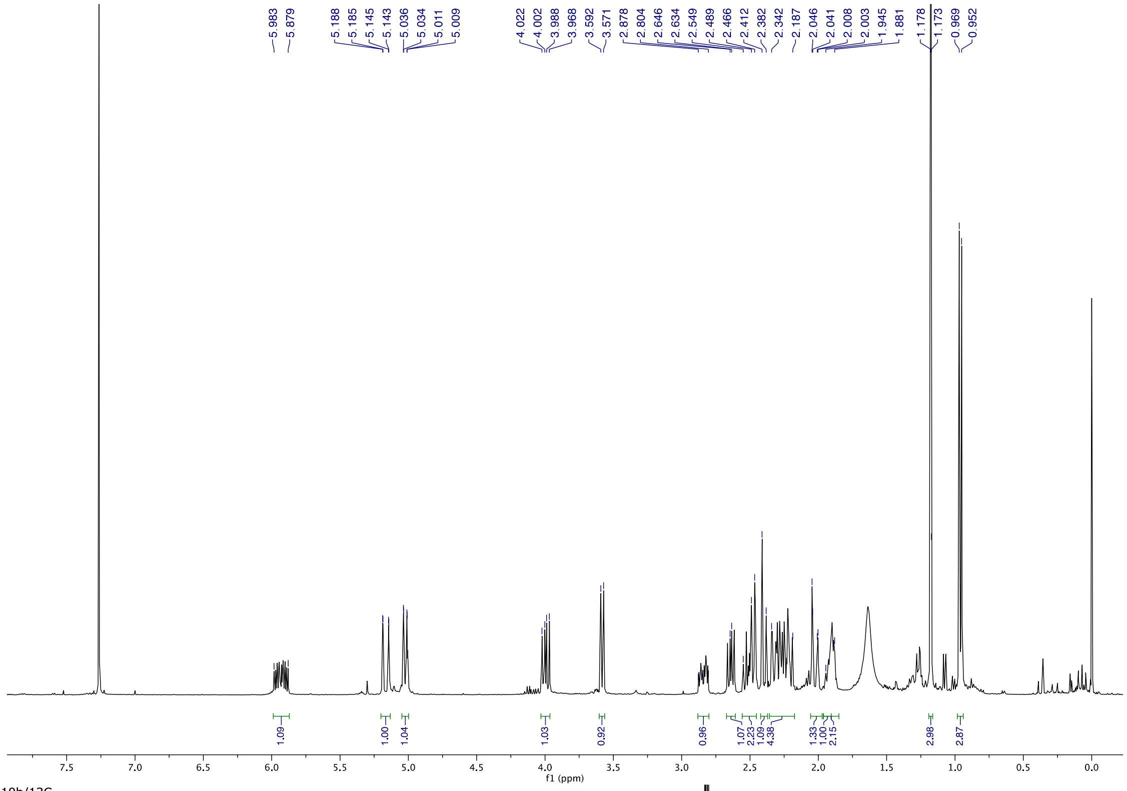

10b/13C
Equip: B400F / N.Inv: 1037597
N.Reg: 22070473
Usuari: san / Mostra: XCM728-57
Nom: CLAUDIA MARQUES GARCIA
Data: 19/07/2022 05:50:40 h./ Ope.: AUTOSERVEI
Experiment: A-C13-zgpg30 Solvent: CDCl3

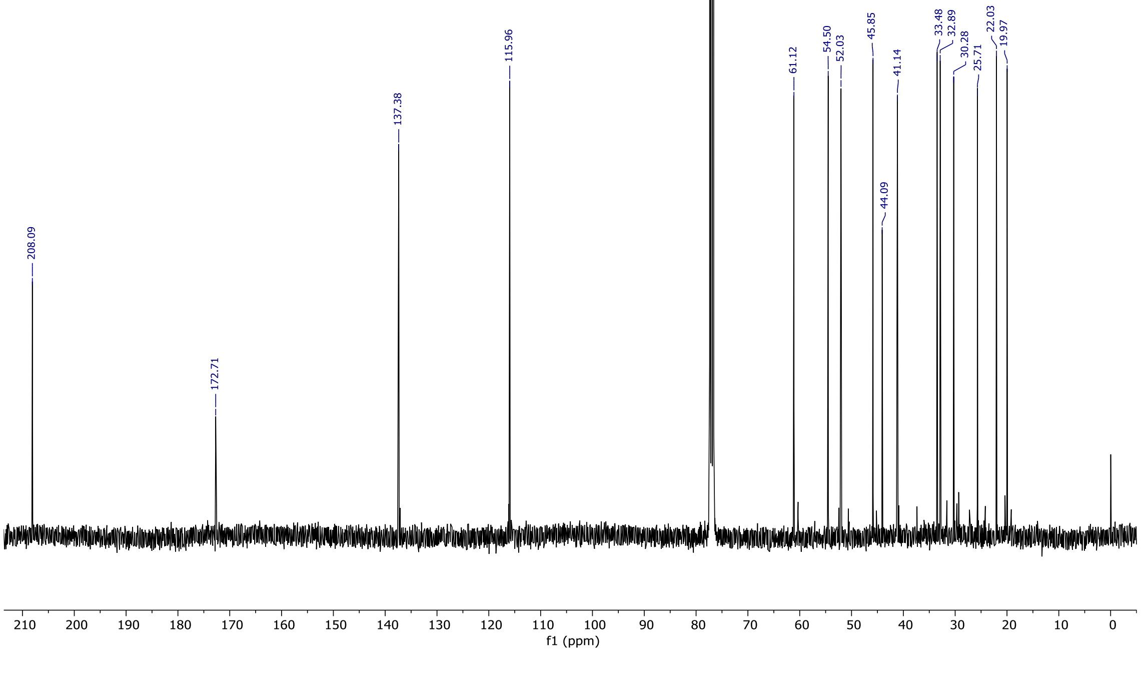

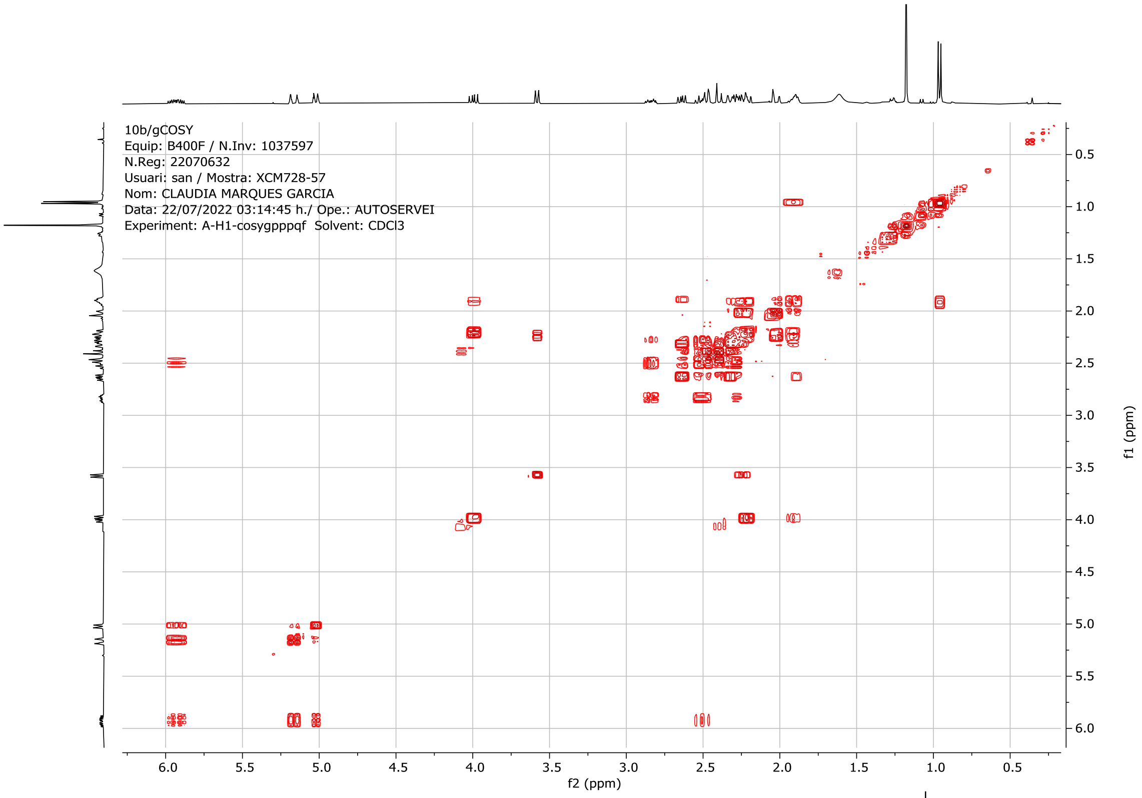
10b/gCOSY
Equip: B400F / N.Inv: 1037597
N.Reg: 22070632
Usuari: san / Mostra: XCM728-57
Nom: CLAUDIA MARQUES GARCIA
Data: 22/07/2022 03:14:45 h./ Ope.: AUTOSERVEI
Experiment: A-H1-cosygpppqf Solvent: CDCl3
f2 (ppm)
f1 (ppm)

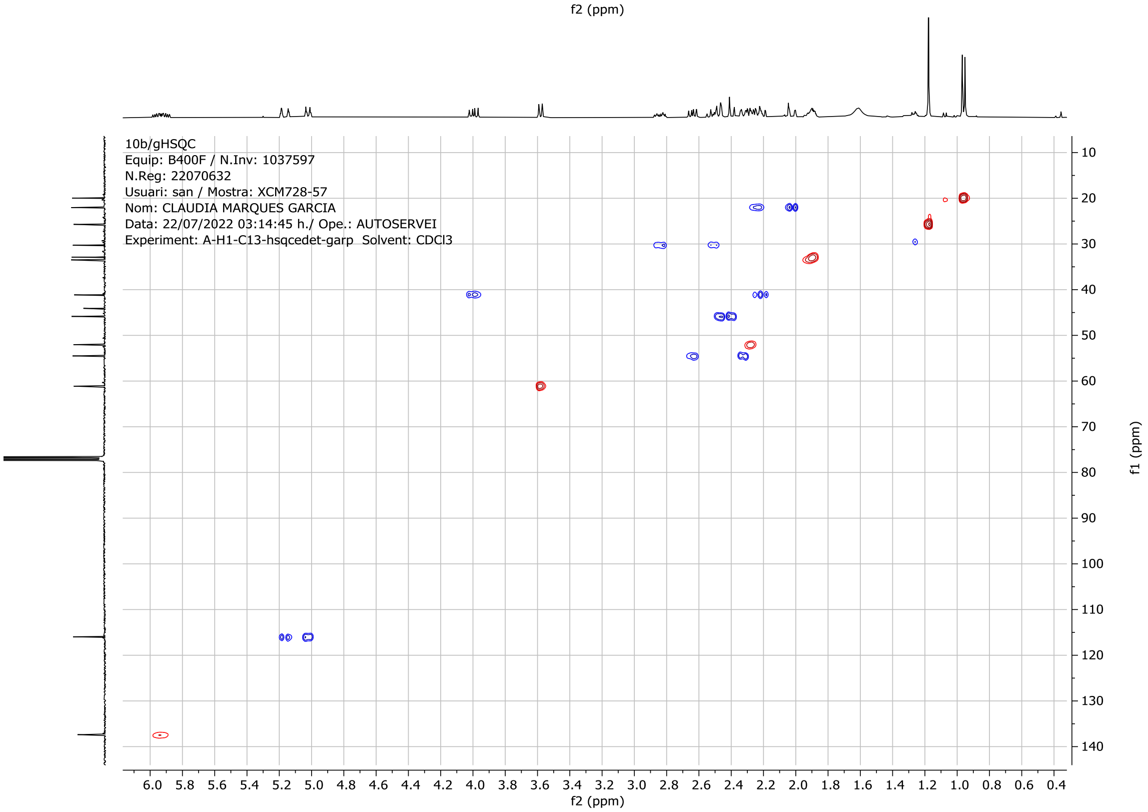
10b/gHSQC
Equip: B400F / N.Inv: 1037597
N.Reg: 22070632
Usuari: san / Mostra: XCM728-57
Nom: CLAUDIA MARQUES GARCIA
Data: 22/07/2022 03:14:45 h./ Ope.: AUTOSERVEI
Experiment: A-H1-C13-hsqcedet-garp Solvent: CDCl3
f2 (ppm)
f1 (ppm)

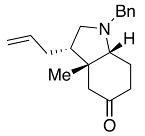

12

12/1H
Equip: B400F / N.Inv: 1037597
N.Reg: 23010638
Usuari: san / Mostra: XCM803-6
Nom: CLAUDIA MARQUES GARCIA
Data: 24/01/2023 10:48:22 h./ Ope.: AUTOSERVEI
Experiment: A-H1-zg30 Solvent: CDCl3

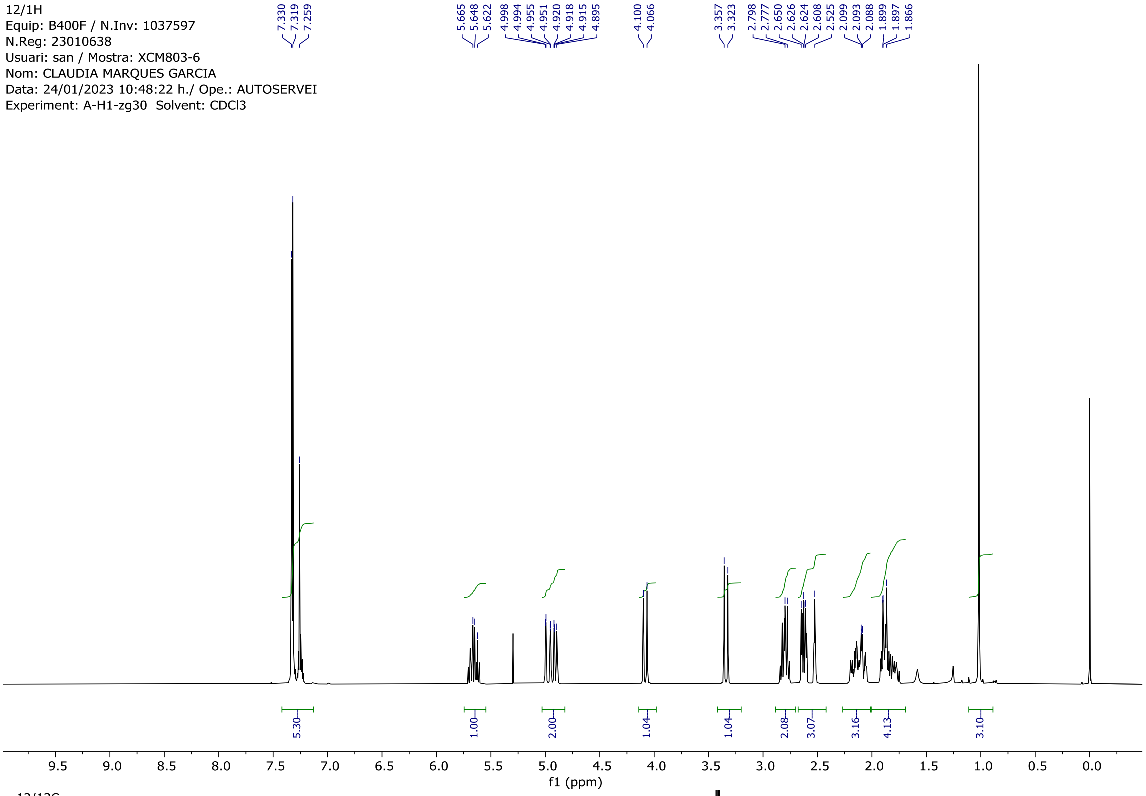

12/13C
Equip: B400F / N.Inv: 1037597
N.Reg: 23010638
Usuari: san / Mostra: XCM803-6
Nom: CLAUDIA MARQUES GARCIA
Data: 24/01/2023 23:54:53 h./ Ope.: AUTOSERVEI
Experiment: A-C13-zgpg30 Solvent: CDCl3

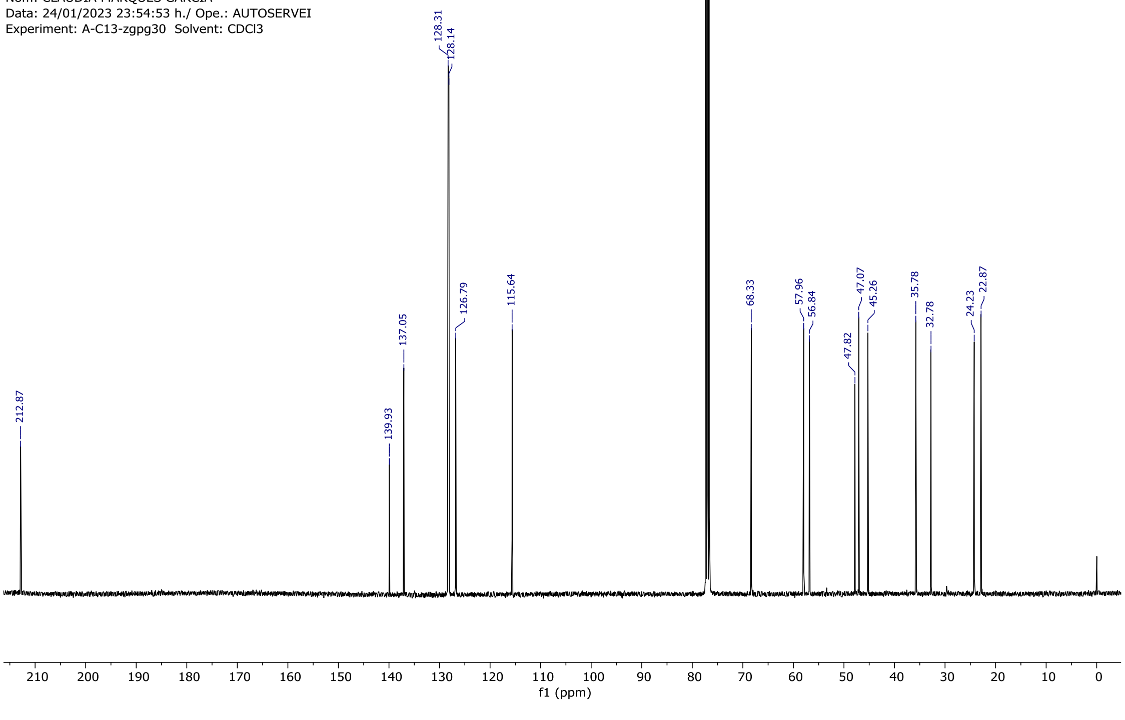

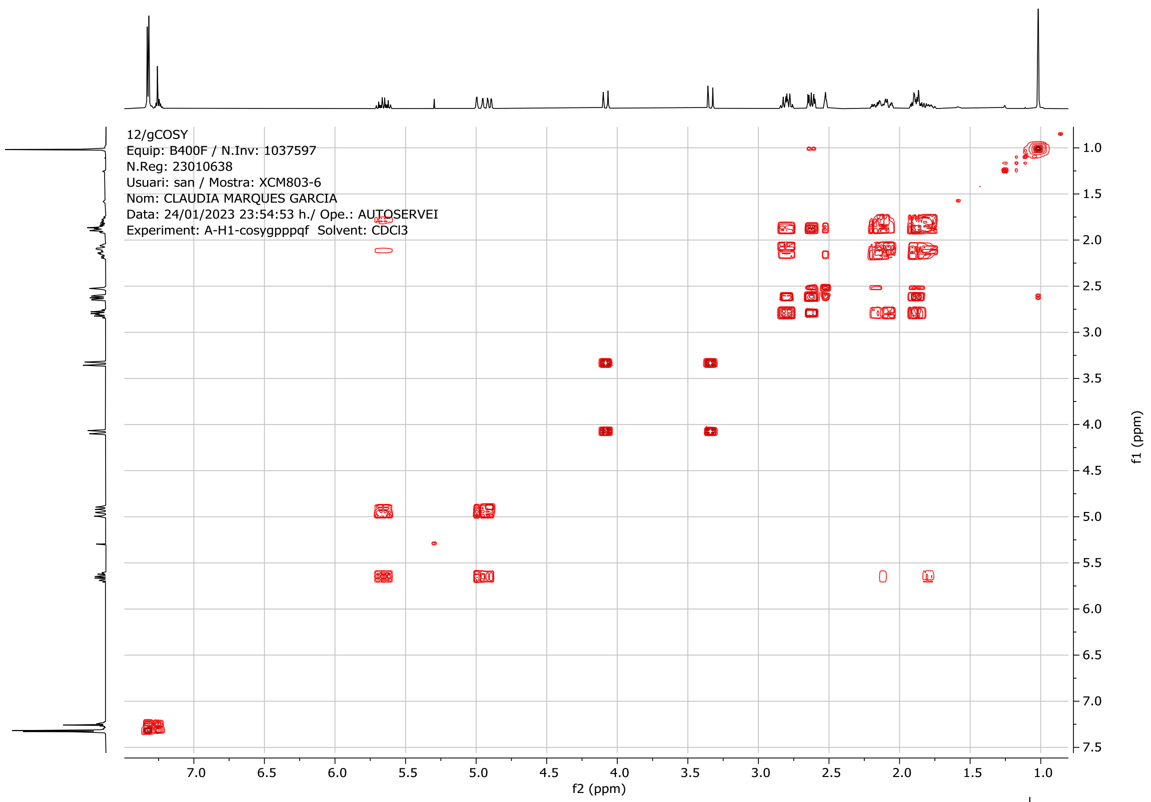
12/gCOSY
Equip: B400F / N.Inv: 1037597
N.Reg: 23010638
Usuari: san / Mostra: XCM803-6
Nom: CLAUDIA MARQUES GARCIA
Data: 24/01/2023 23:54:53 h./ Ope.: AUTOSERVEI
Experiment: A-H1-cosygpppqf Solvent: CDCl3
f2 (ppm)
f1 (ppm)

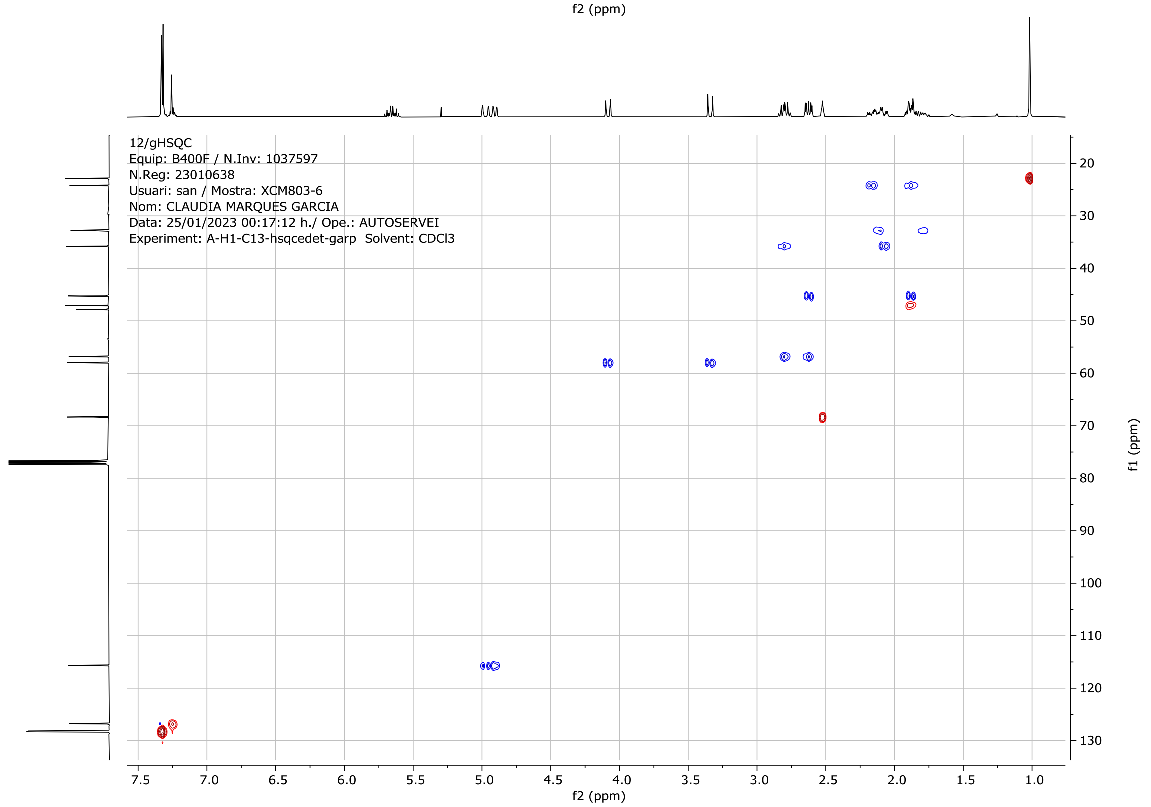
12/gHSQC
Equip: B400F / N.Inv: 1037597
N.Reg: 23010638
Usuari: san / Mostra: XCM803-6
Nom: CLAUDIA MARQUES GARCIA
Data: 25/01/2023 00:17:12 h./ Ope.: AUTOSERVEI
Experiment: A-H1-C13-hsqcedet-garp Solvent: CDCl3
f2 (ppm)
f1 (ppm)

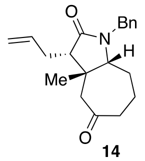
O
N
Bn
H
Me
O

14

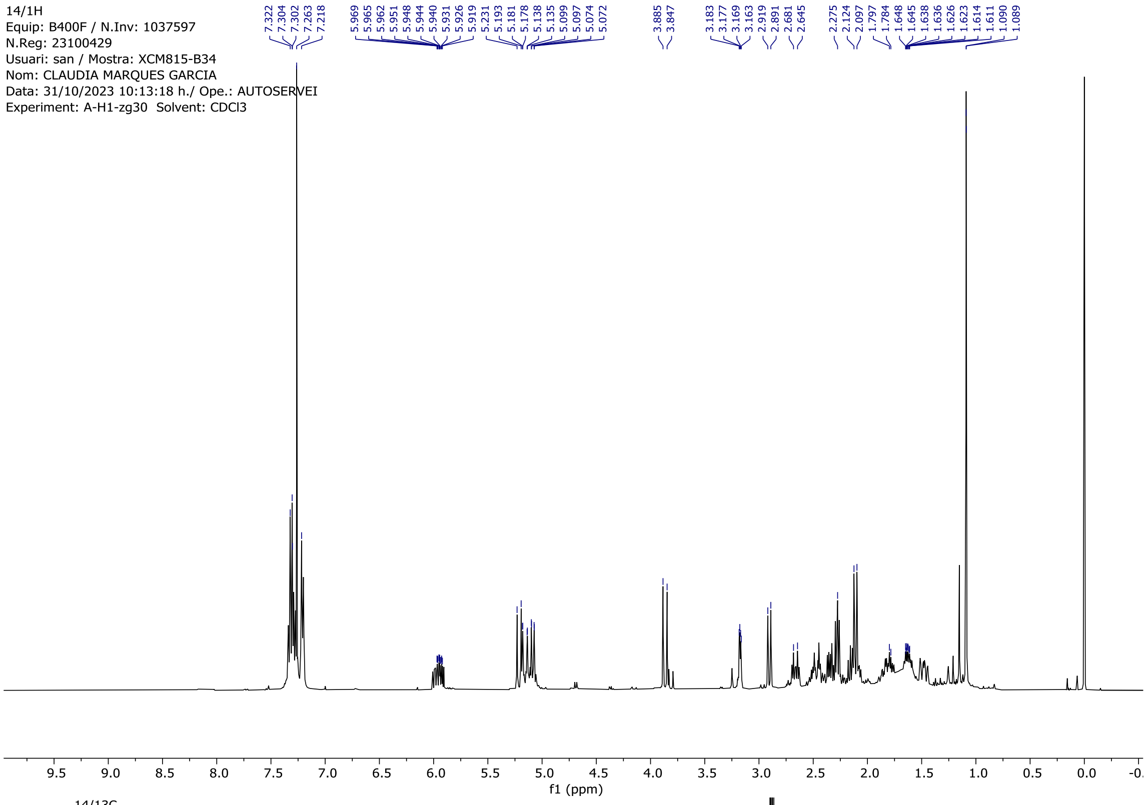
14/1H
Equip: B400F / N.Inv: 1037597
N.Reg: 23100429
Usuari: san / Mostra: XCM815-B34
Nom: CLAUDIA MARQUES GARCIA
Data: 31/10/2023 10:13:18 h./ Ope.: AUTOSERVEI
Experiment: A-H1-zg30 Solvent: CDCl3
7.322
7.304
7.302
7.263
7.218
5.969
5.965
5.962
5.951
5.948
5.944
5.940
5.931
5.926
5.919
5.231
5.193
5.181
5.178
5.138
5.135
5.099
5.097
5.074
5.072
3.885
3.847
3.183
3.177
3.169
3.163
2.919
2.891
2.681
2.645
2.275
2.124
2.097
1.797
1.784
1.648
1.645
1.638
1.636
1.626
1.623
1.614
1.611
1.090
1.089
9.5
9.0
8.5
8.0
7.5
7.0
6.5
6.0
5.5
5.0
4.5
4.0
3.5
3.0
2.5
2.0
1.5
1.0
0.5
0.0
f1 (ppm)

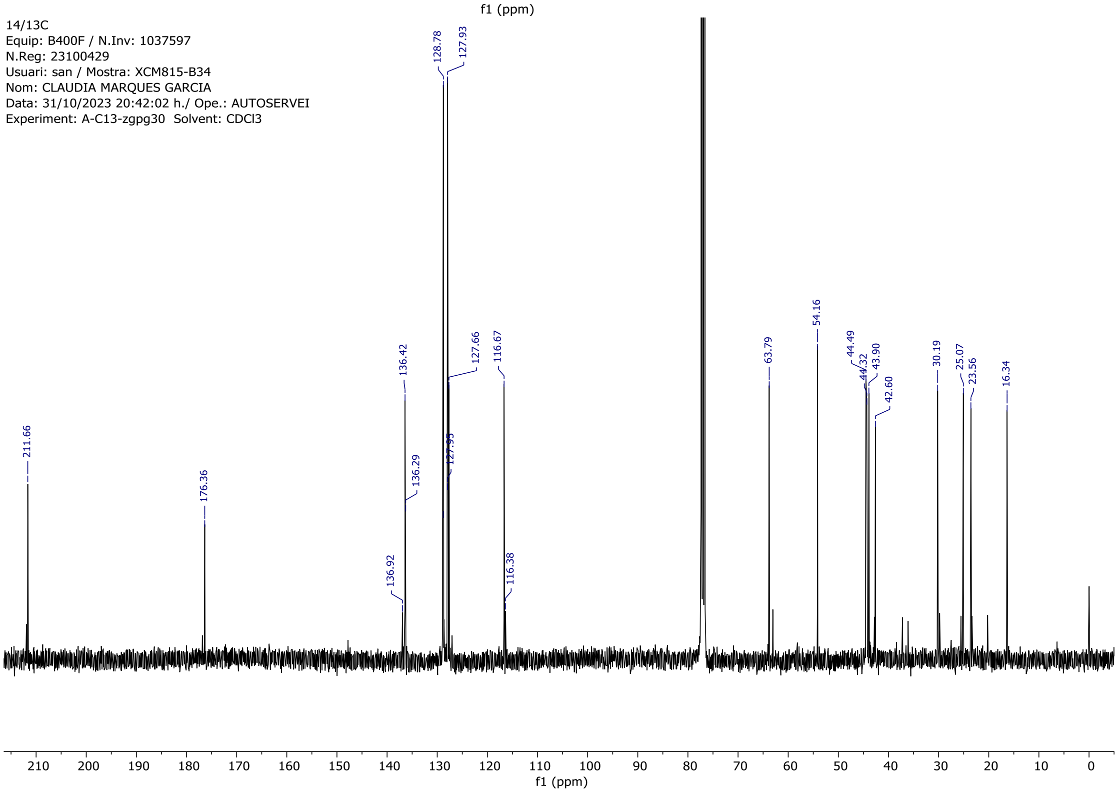
14/13C
Equip: B400F / N.Inv: 1037597
N.Reg: 23100429
Usuari: san / Mostra: XCM815-B34
Nom: CLAUDIA MARQUES GARCIA
Data: 31/10/2023 20:42:02 h./ Ope.: AUTOSERVEI
Experiment: A-C13-zgpg30 Solvent: CDCl3
128.78
127.93
211.66
176.36
136.42
136.29
136.92
127.66
127.93
116.67
116.38
63.79
54.16
44.49
44.32
43.90
42.60
30.19
25.07
23.56
16.34
210
200
190
180
170
160
150
140
130
120
110
100
90
80
70
60
50
40
30
20
10
0
f1 (ppm)

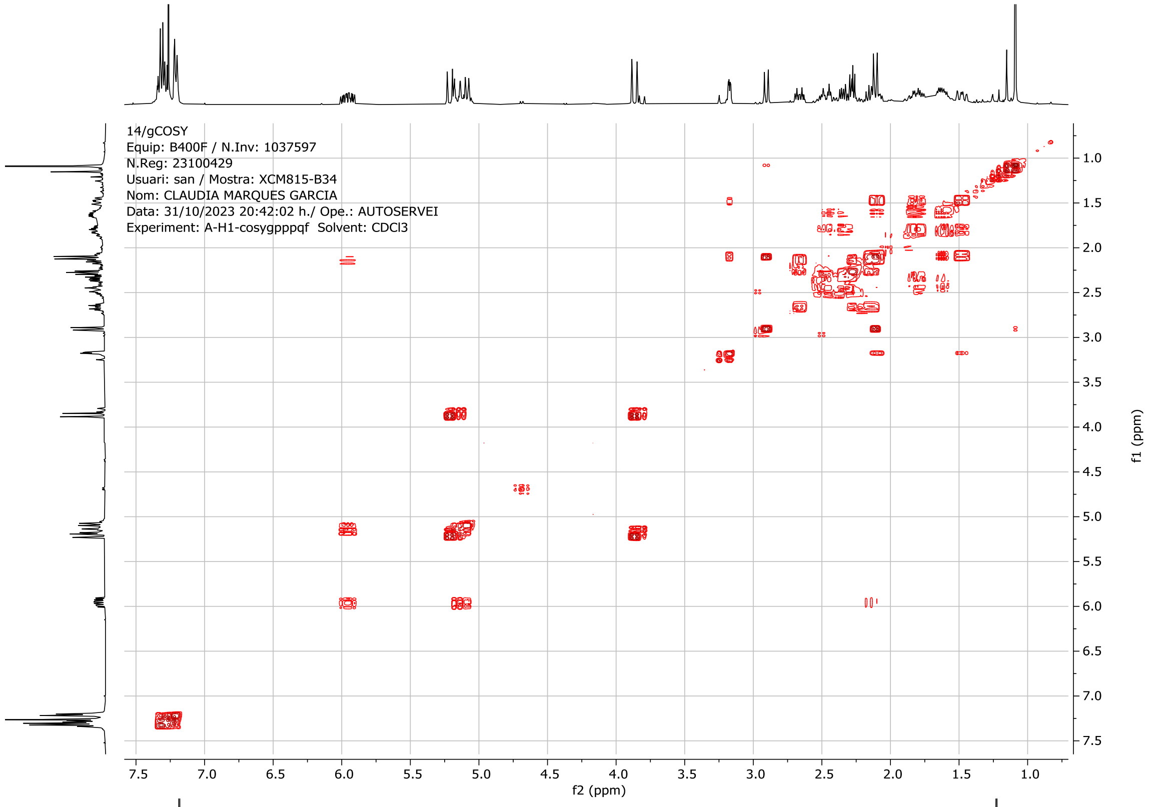
14/gCOSY
Equip: B400F / N.Inv: 1037597
N.Reg: 23100429
Usuari: san / Mostra: XCM815-B34
Nom: CLAUDIA MARQUES GARCIA
Data: 31/10/2023 20:42:02 h./ Ope.: AUTOSERVEI
Experiment: A-H1-cosygpppqf Solvent: CDCl3
f2 (ppm)
f1 (ppm)

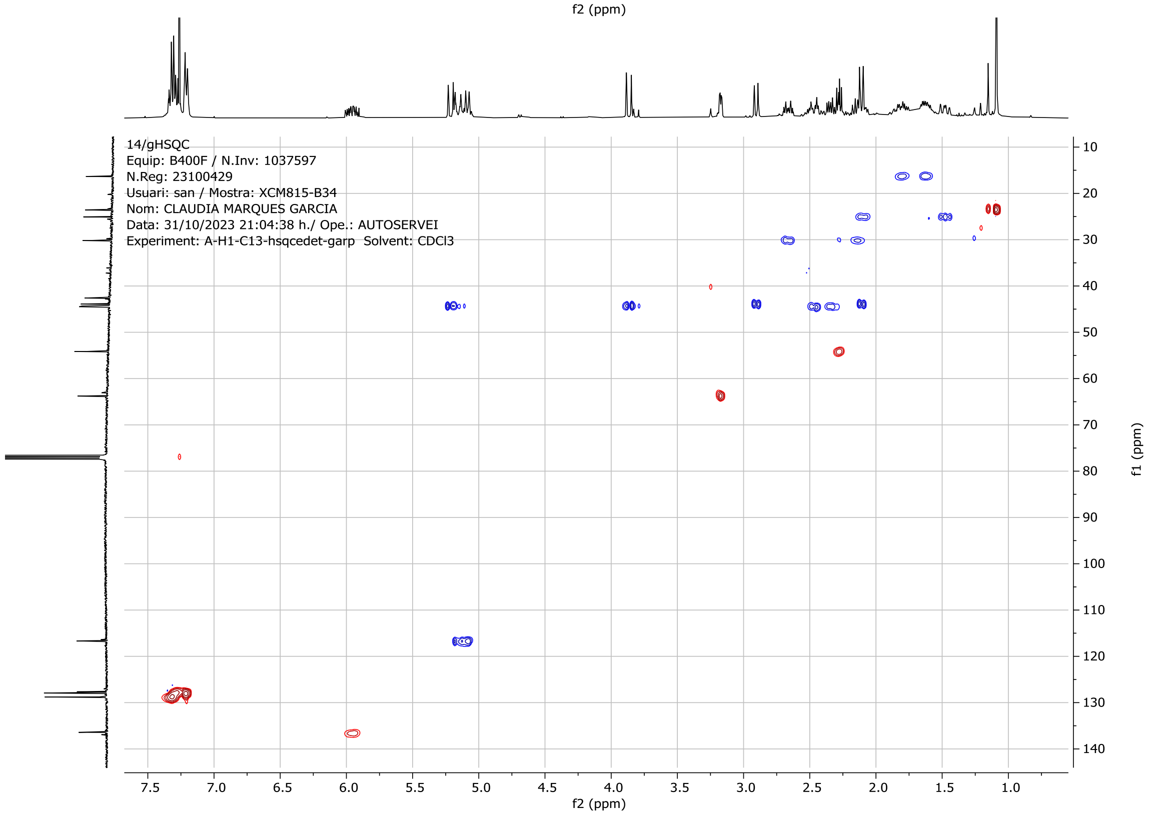
14/gHSQC
Equip: B400F / N.Inv: 1037597
N.Reg: 23100429
Usuari: san / Mostra: XCM815-B34
Nom: CLAUDIA MARQUES GARCIA
Data: 31/10/2023 21:04:38 h./ Ope.: AUTOSERVEI
Experiment: A-H1-C13-hsqcedet-garp Solvent: CDCl3
f2 (ppm)
f1 (ppm)

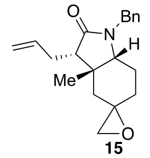

15

15/1H
Equip: B400F / N.Inv: 1037597
N.Reg: 23031028
Usuari: san / Mostra: CM831-46
Nom: CLAUDIA MARQUES GARCIA
Data: 28/03/2023 11:15:56 h./ Ope.: AUTOSERVEI
Experiment: A-H1-zg30 Solvent: CDCl3

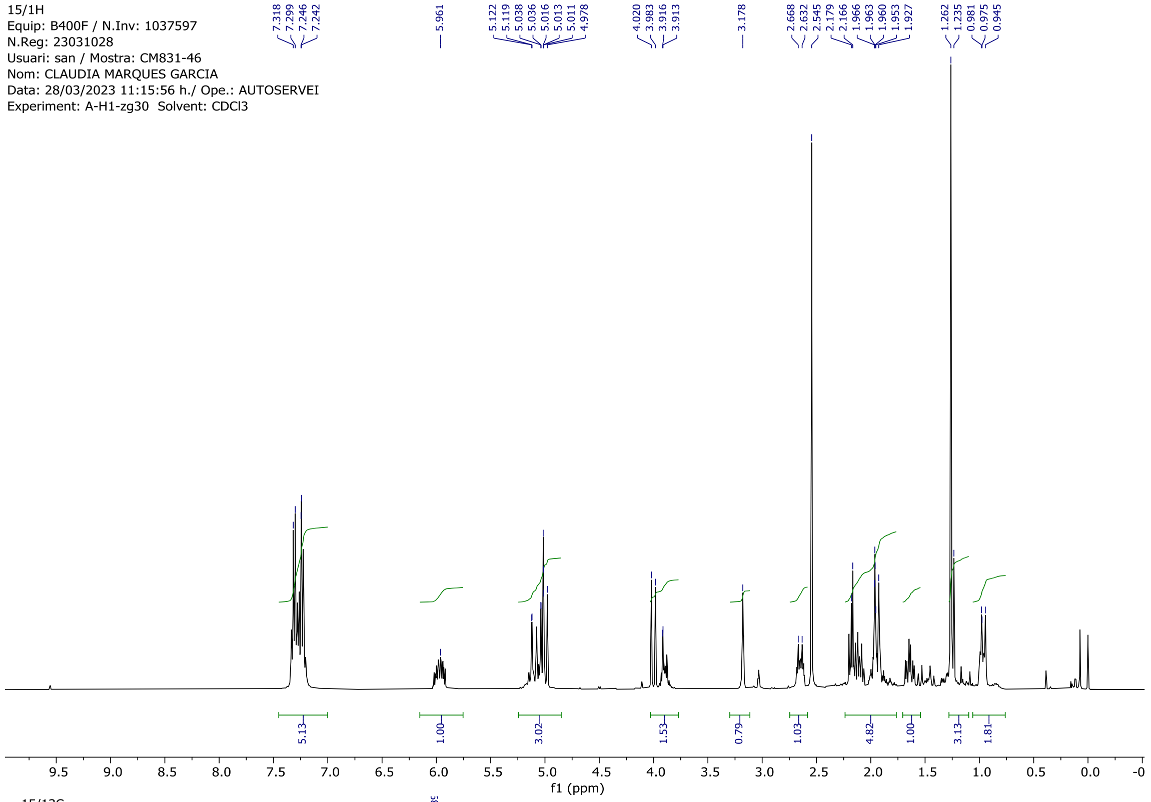

15/13C
Equip: B400F / N.Inv: 1037597
N.Reg: 23031028
Usuari: san / Mostra: CM831-46
Nom: CLAUDIA MARQUES GARCIA
Data: 29/03/2023 04:59:20 h./ Ope.: AUTOSERVEI
Experiment: A-C13-zgpg30 Solvent: CDCl3

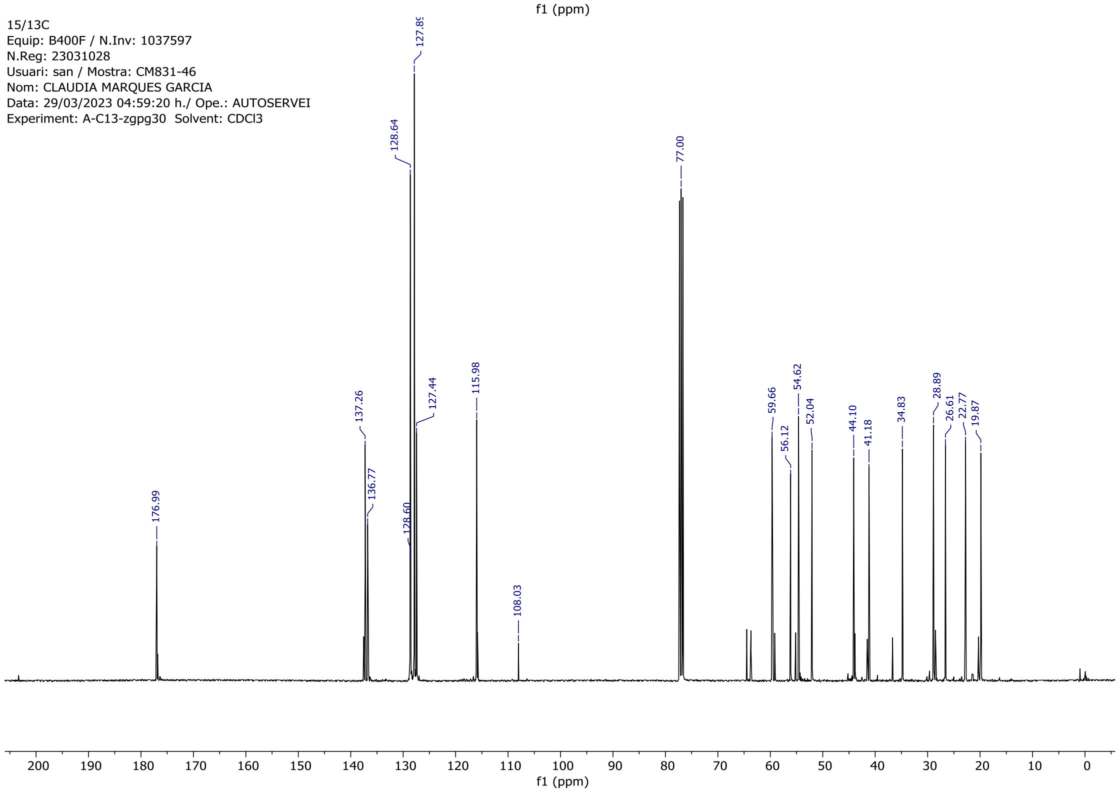

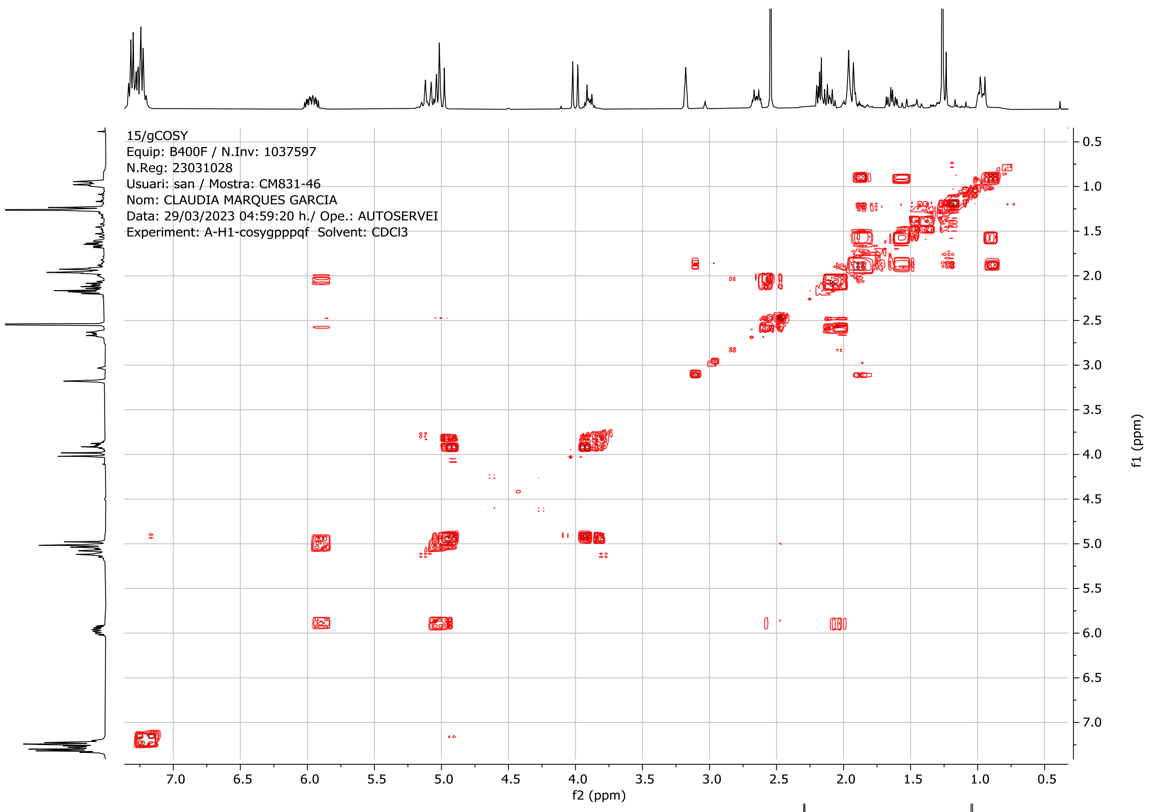
15/gCOSY
Equip: B400F / N.Inv: 1037597
N.Reg: 23031028
Usuari: san / Mostra: CM831-46
Nom: CLAUDIA MARQUES GARCIA
Data: 29/03/2023 04:59:20 h./ Ope.: AUTOSERVEI
Experiment: A-H1-cosygpppqf Solvent: CDCl3
f2 (ppm)
f1 (ppm)

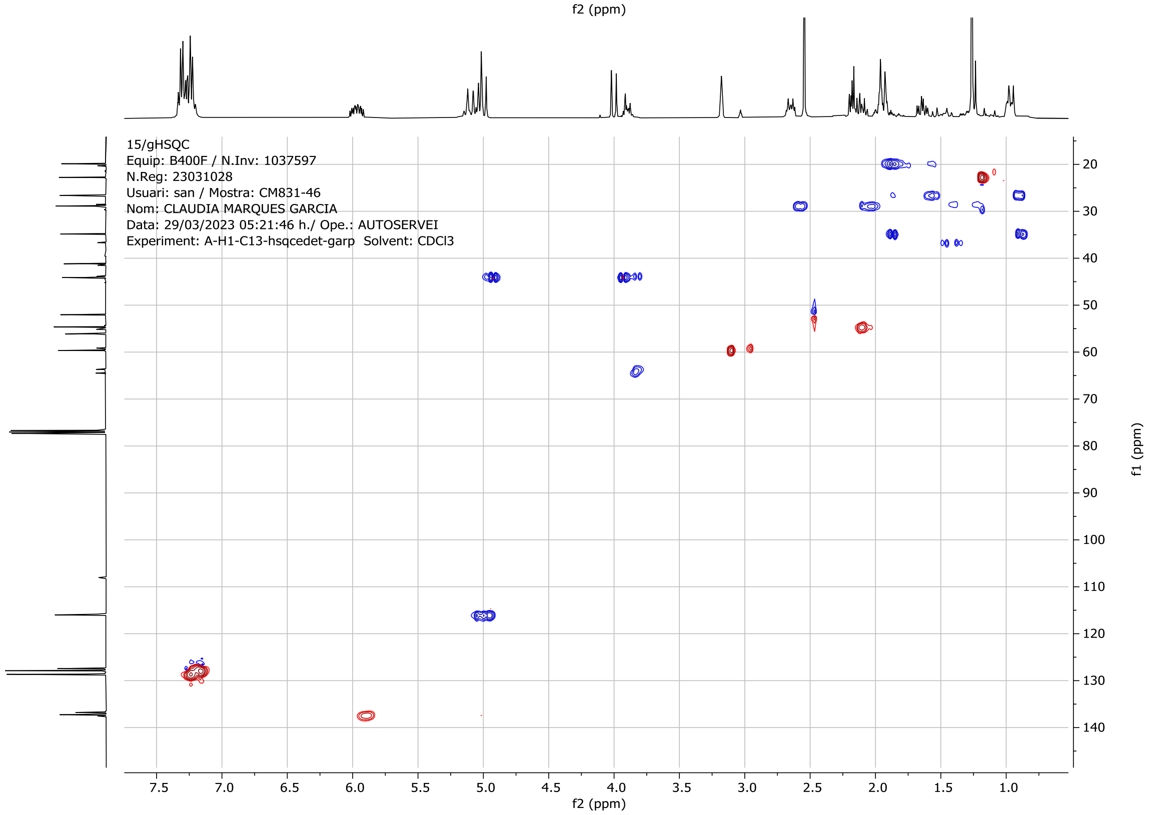
15/gHSQC
Equip: B400F / N.Inv: 1037597
N.Reg: 23031028
Usuari: san / Mostra: CM831-46
Nom: CLAUDIA MARQUES GARCIA
Data: 29/03/2023 05:21:46 h./ Ope.: AUTOSERVEI
Experiment: A-H1-C13-hsqcedet-garp Solvent: CDCl3
f2 (ppm)
f1 (ppm)

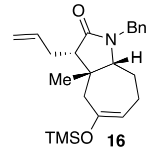

16

16/1H
Equip: B400F / N.Inv: 1037597
N.Reg: 23031029
Usuari: san / Mostra: CM848-12
Nom: CLAUDIA MARQUES GARCIA
Data: 28/03/2023 11:21:50 h./ Ope.: AUTOSERVEI
Experiment: A-H1-zg30 Solvent: CDCl3

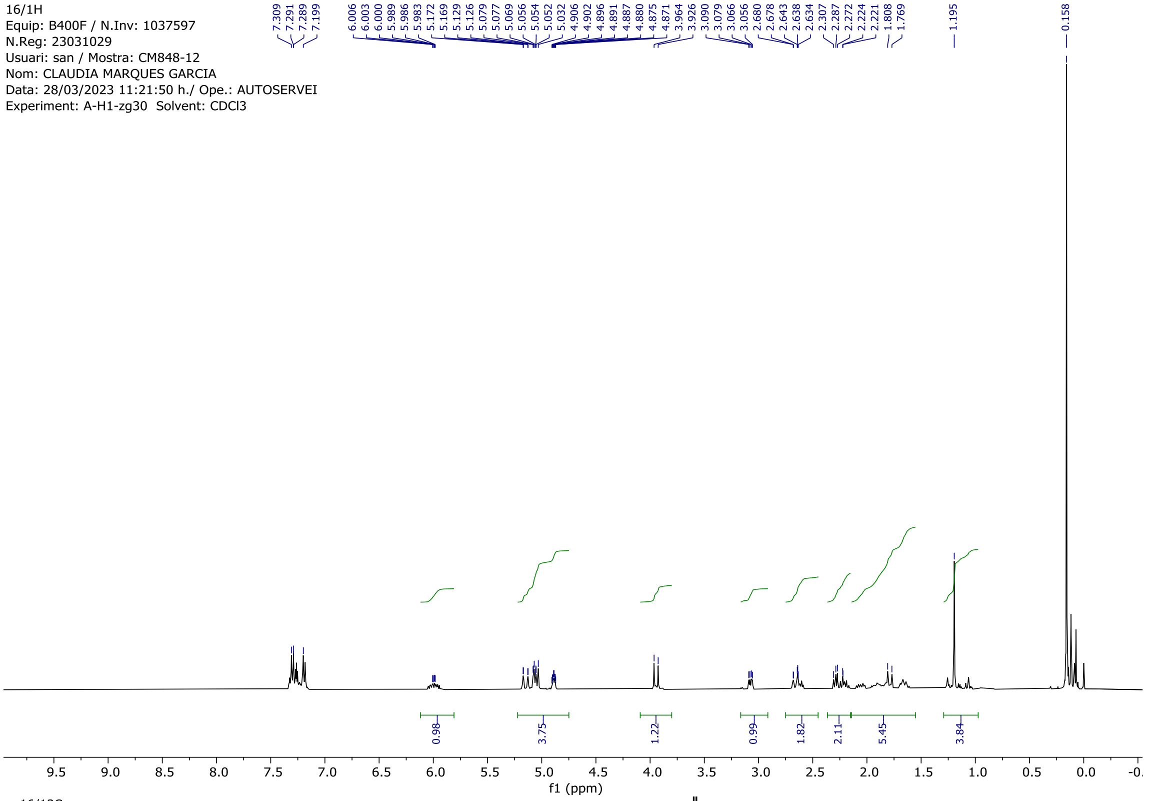

16/13C
Equip: B400F / N.Inv: 1037597
N.Reg: 23031029
Usuari: san / Mostra: CM848-12
Nom: CLAUDIA MARQUES GARCIA
Data: 29/03/2023 07:17:13 h./ Ope.: AUTOSERVEI
Experiment: A-C13-zgpg30 Solvent: CDCl3

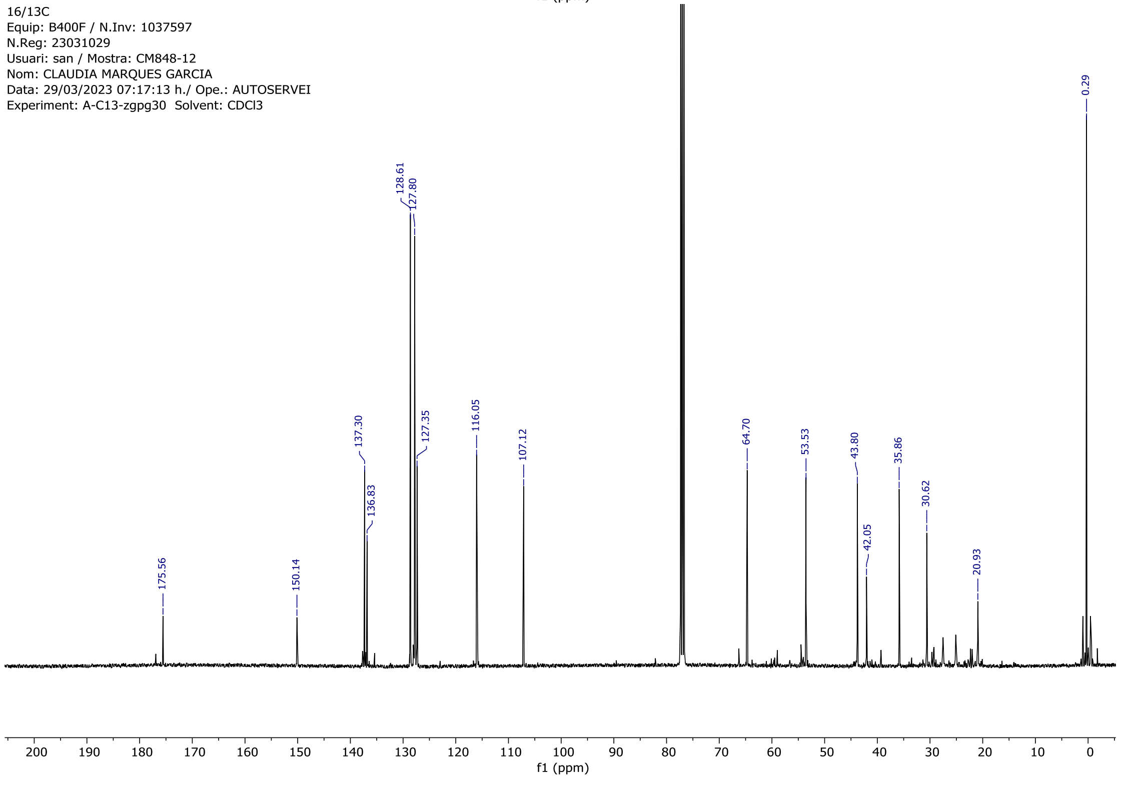

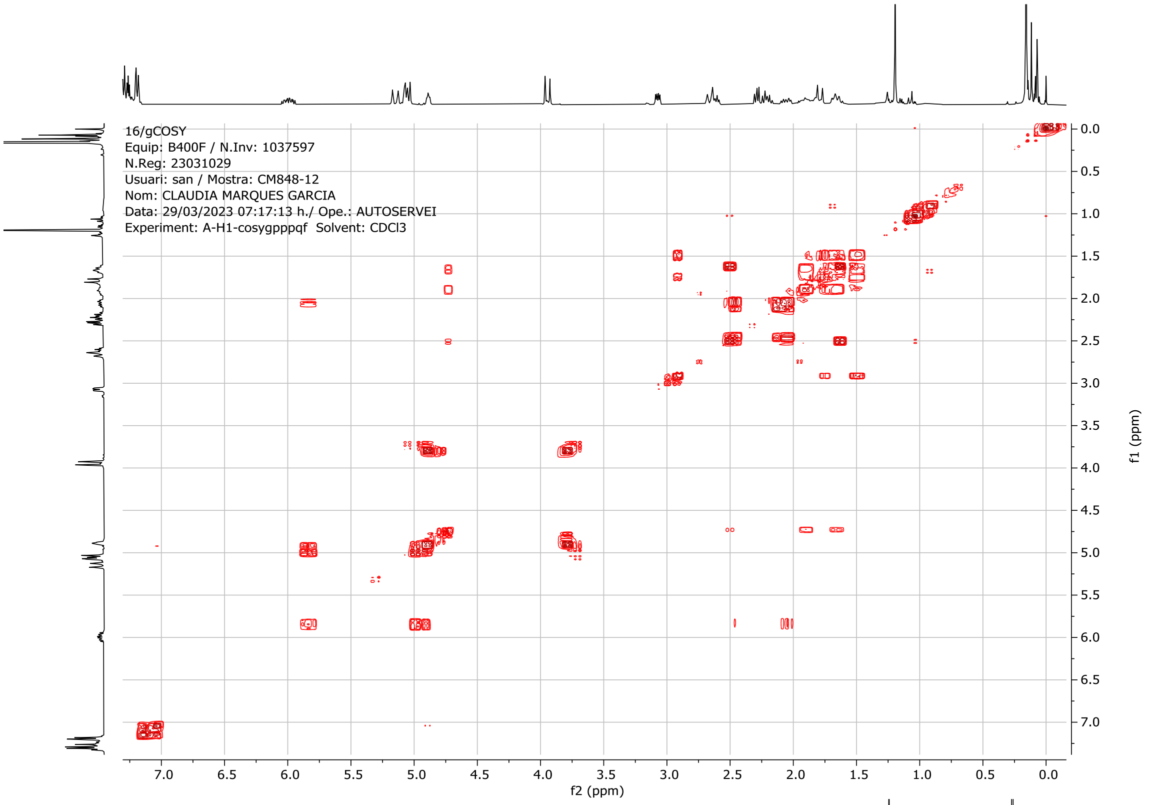
16/gCOSY
Equip: B400F / N.Inv: 1037597
N.Reg: 23031029
Usuari: san / Mostra: CM848-12
Nom: CLAUDIA MARQUES GARCIA
Data: 29/03/2023 07:17:13 h./ Ope.: AUTOSERVEI
Experiment: A-H1-cosygpppqf Solvent: CDCl3
f2 (ppm)
f1 (ppm)

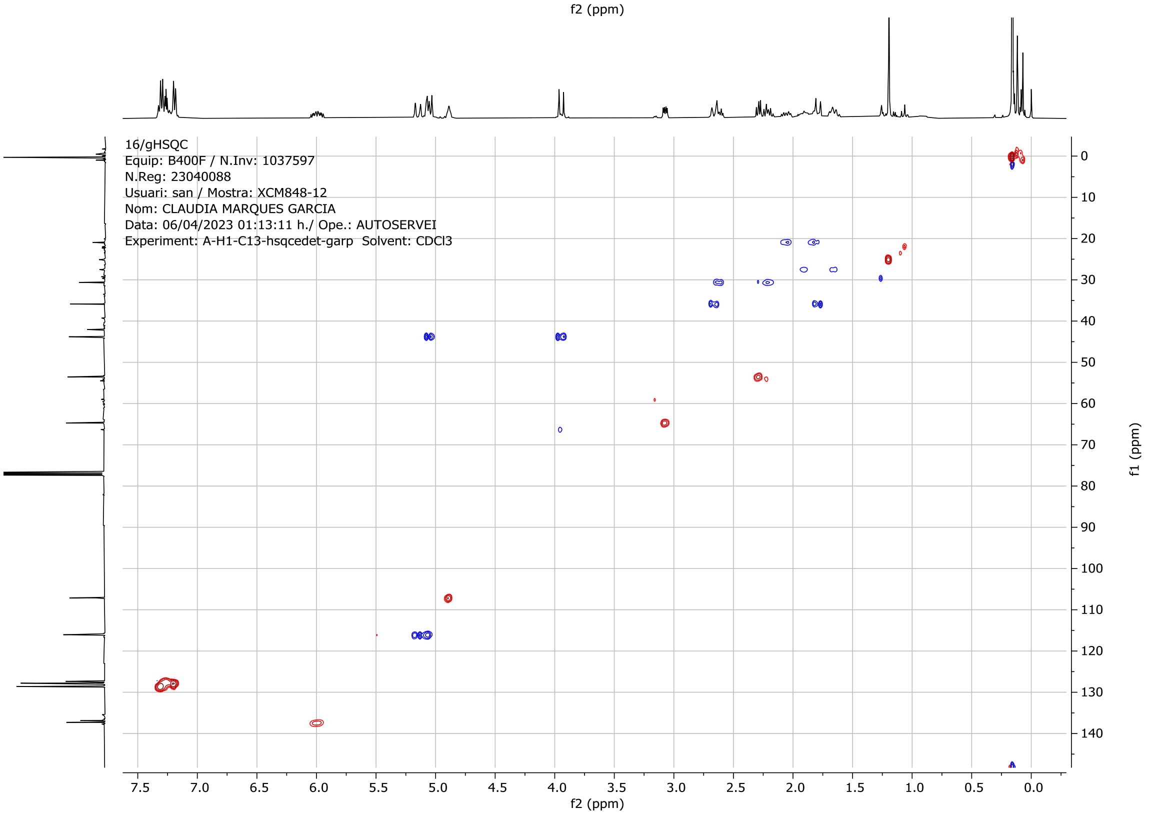
16/gHSQC
Equip: B400F / N.Inv: 1037597
N.Reg: 23040088
Usuari: san / Mostra: XCM848-12
Nom: CLAUDIA MARQUES GARCIA
Data: 06/04/2023 01:13:11 h./ Ope.: AUTOSERVEI
Experiment: A-H1-C13-hsqcedet-garp Solvent: CDCl3
f2 (ppm)
f1 (ppm)

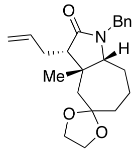

17

17/1H
Equip: B400F / N.Inv: 1037597
N.Reg: 24020415
Usuari: san / Mostra: CM937-60
Nom: CLAUDIA MARQUES GARCIA
Data: 14/02/2024 10:58:03 h./ Ope.: AUTOSERVEI
Experiment: A-H1-zg30 Solvent: CDCl3

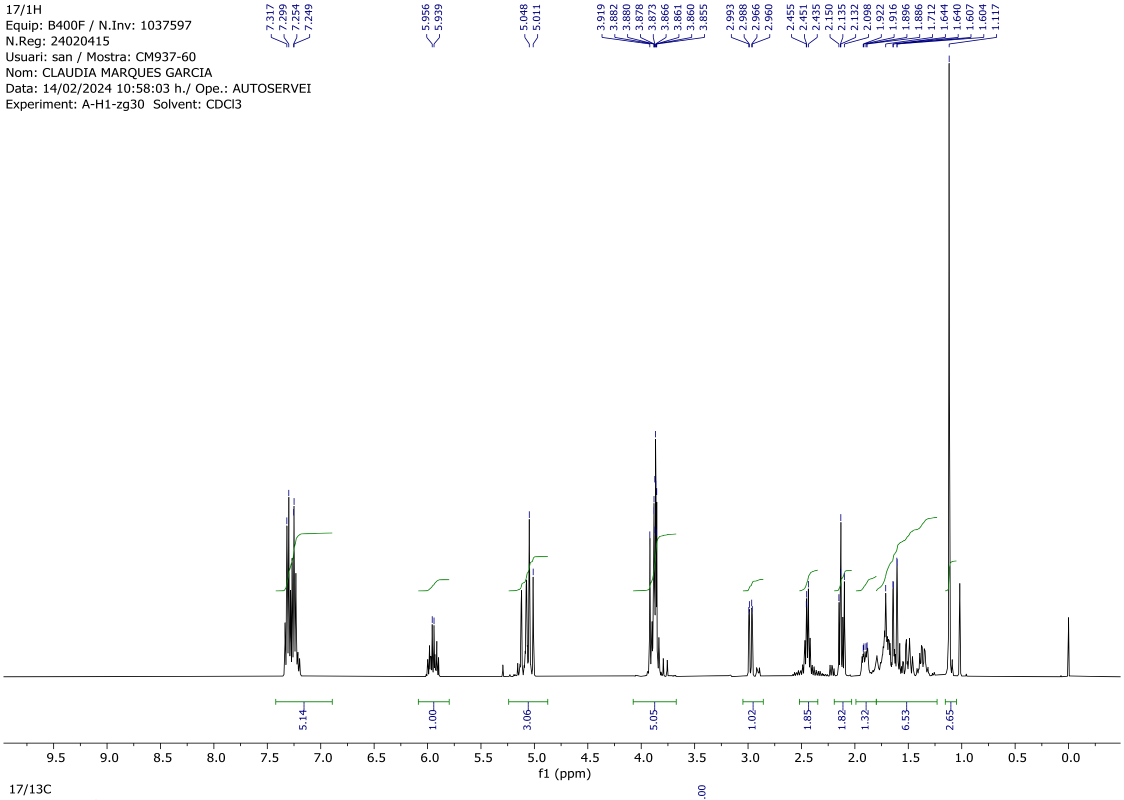

17/13C
Equip: B400F / N.Inv: 1037597
N.Reg: 24020415
Usuari: san / Mostra: CM937-60
Nom: CLAUDIA MARQUES GARCIA
Data: 14/02/2024 23:55:46 h./ Ope.: AUTOSERVEI
Experiment: A-C13-zgpg30 Solvent: CDCl3

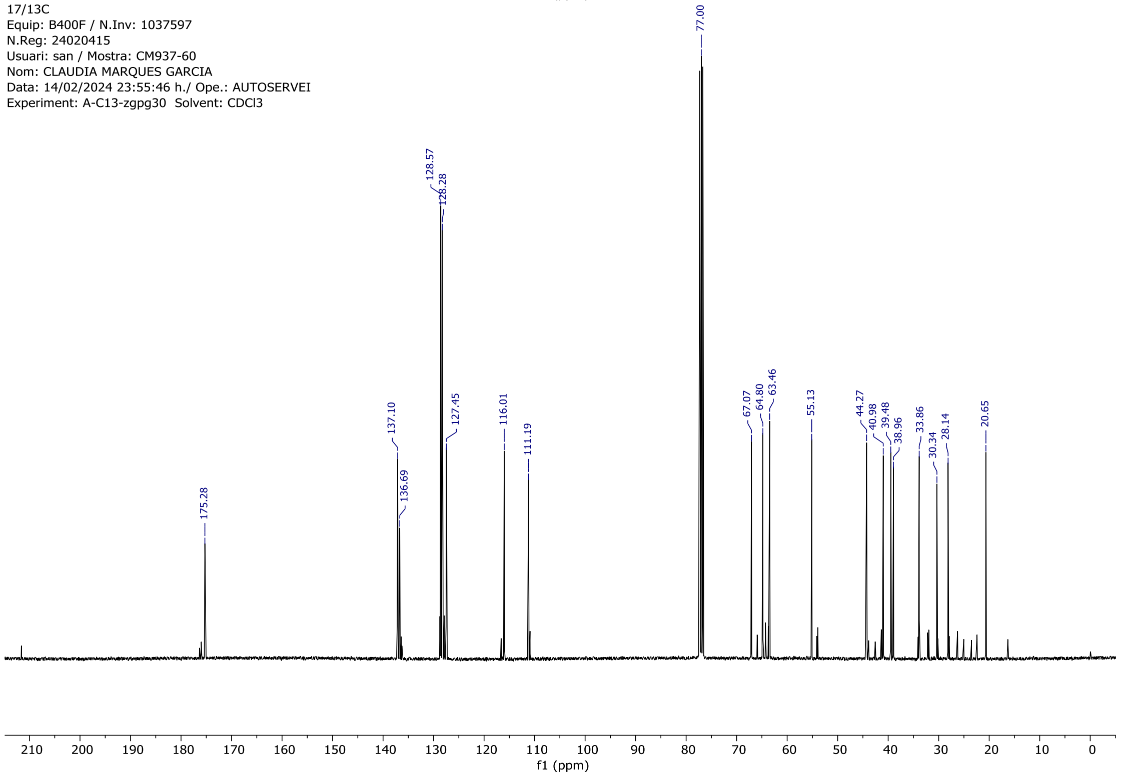

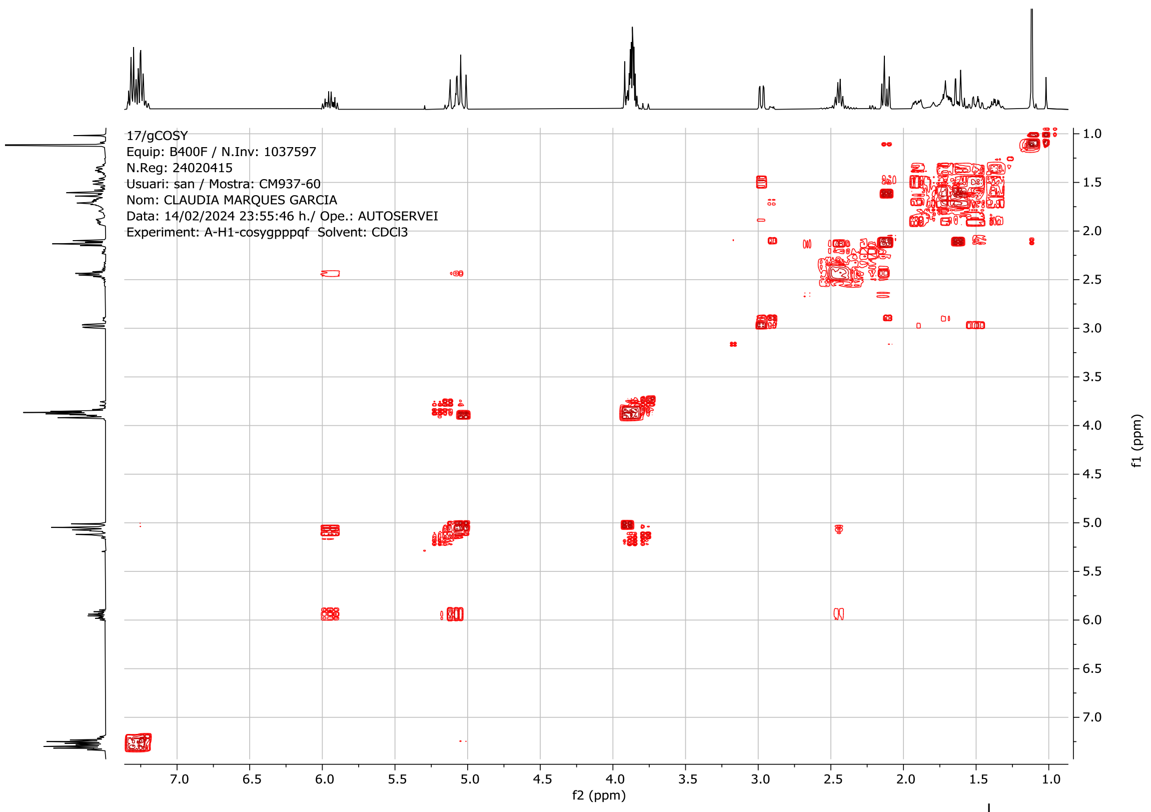
17/gCOSY
Equip: B400F / N.Inv: 1037597
N.Reg: 24020415
Usuari: san / Mostra: CM937-60
Nom: CLAUDIA MARQUES GARCIA
Data: 14/02/2024 23:55:46 h./ Ope.: AUTOSERVEI
Experiment: A-H1-cosygpppqf Solvent: CDCl3
f2 (ppm)
f1 (ppm)

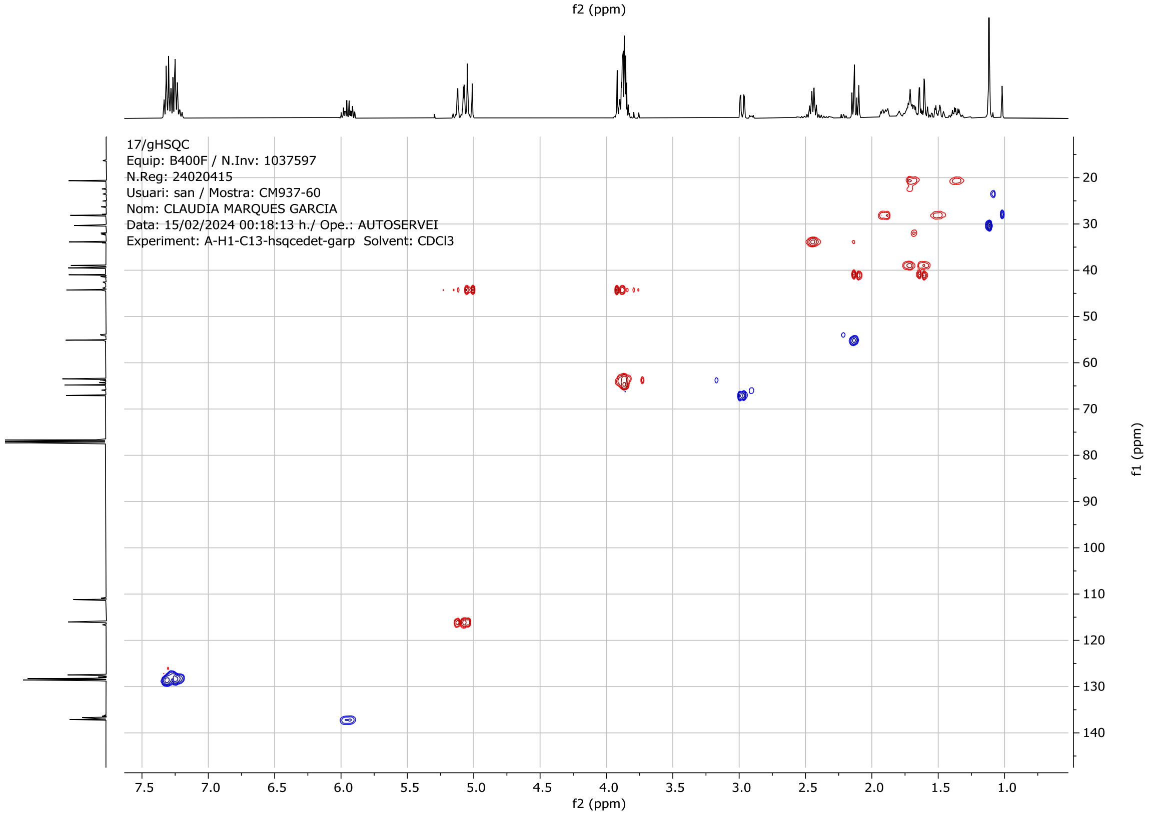
17/gHSQC
Equip: B400F / N.Inv: 1037597
N.Reg: 24020415
Usuari: san / Mostra: CM937-60
Nom: CLAUDIA MARQUES GARCIA
Data: 15/02/2024 00:18:13 h./ Ope.: AUTOSERVEI
Experiment: A-H1-C13-hsqcedet-garp Solvent: CDCl3
f2 (ppm)
f1 (ppm)

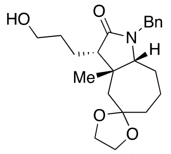

18

18/1H
Equip: B400F / N.Inv: 1037597
N.Reg: 23030266
Usuari: san / Mostra: CM834-20
Nom: CLAUDIA MARQUES GARCIA
Data: 06/03/2023 18:50:00 h./ Ope.: AUTOSERVEI
Experiment: A-H1-zg30 Solvent: CDCl3

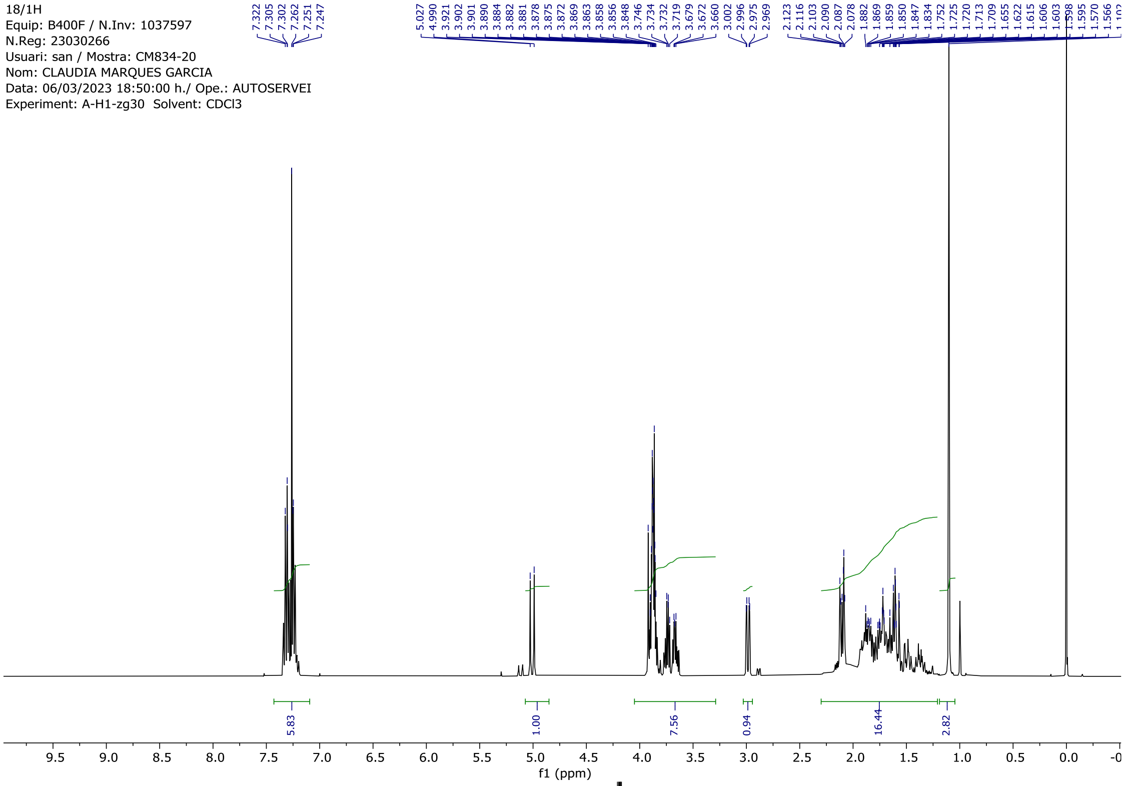

18/13C
Equip: B400F / N.Inv: 1037597
N.Reg: 23030266
Usuari: san / Mostra: CM834-20
Nom: CLAUDIA MARQUES GARCIA
Data: 07/03/2023 00:50:52 h./ Ope.: AUTOSERVEI
Experiment: A-C13-zgpg30 Solvent: CDCl3

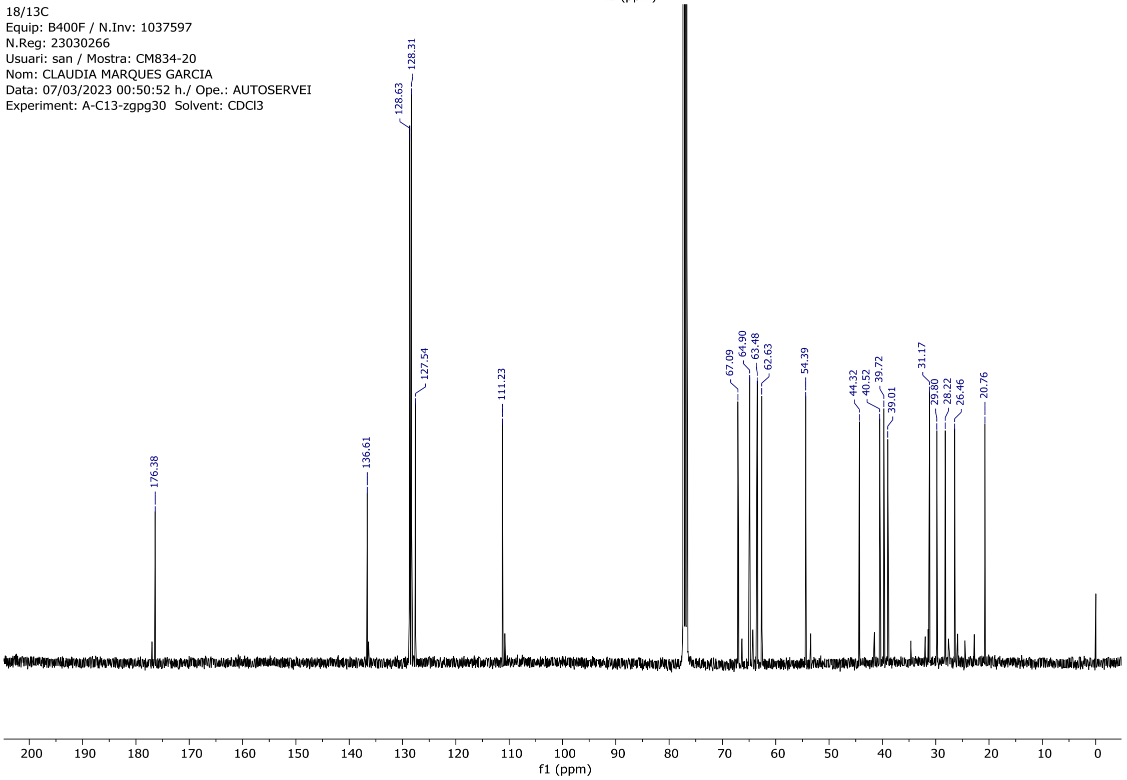

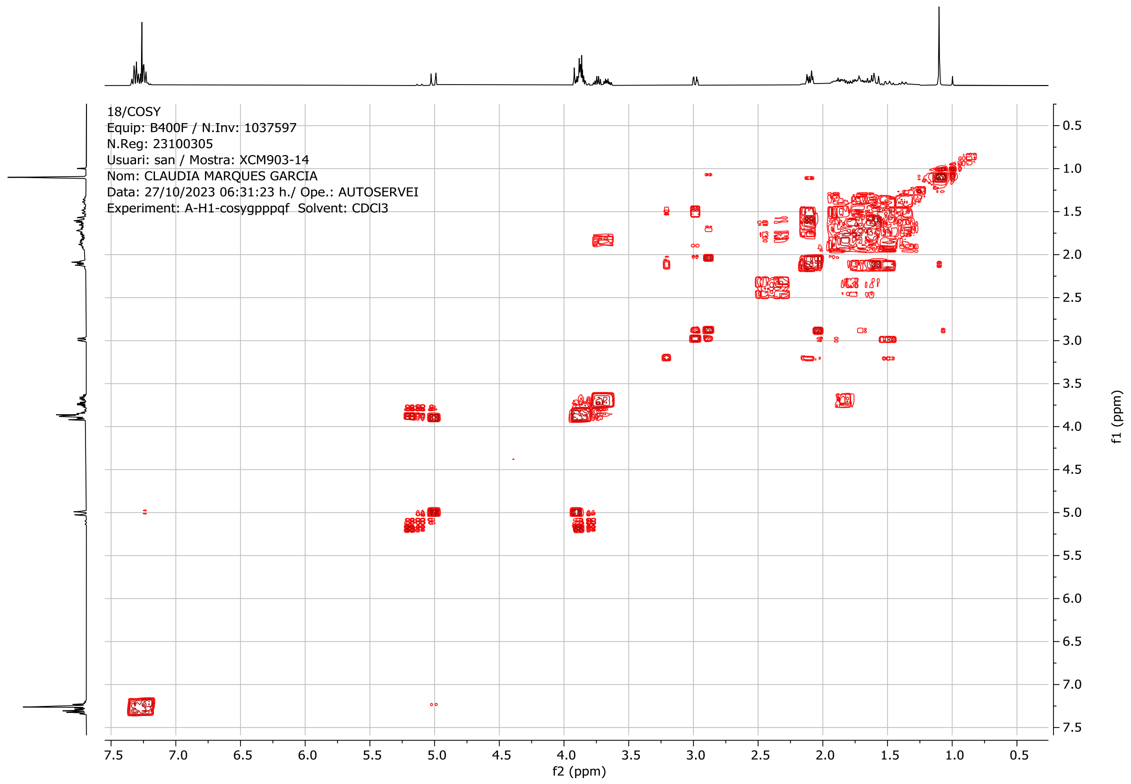
18/COSY
Equip: B400F / N.Inv: 1037597
N.Reg: 23100305
Usuari: san / Mostra: XCM903-14
Nom: CLAUDIA MARQUES GARCIA
Data: 27/10/2023 06:31:23 h./ Ope.: AUTOSERVEI
Experiment: A-H1-cosygpppqf Solvent: CDCl3
f2 (ppm)
f1 (ppm)

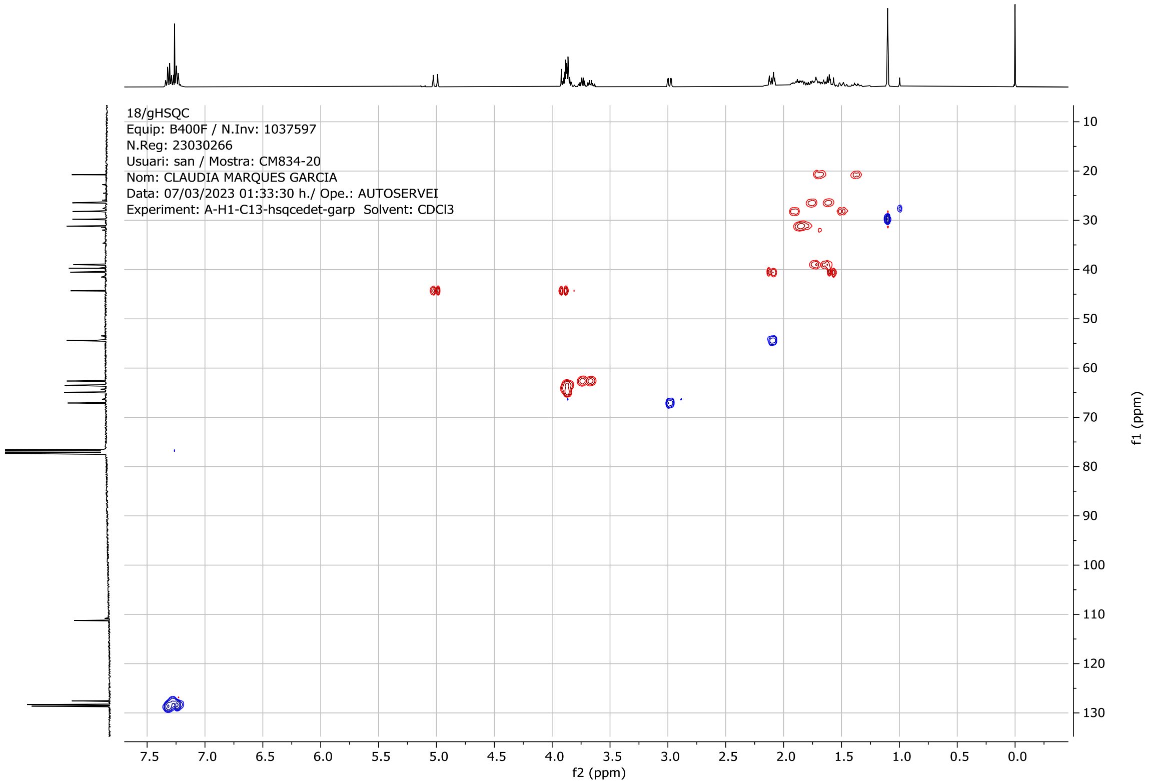
18/gHSQC
Equip: B400F / N.Inv: 1037597
N.Reg: 23030266
Usuari: san / Mostra: CM834-20
Nom: CLAUDIA MARQUES GARCIA
Data: 07/03/2023 01:33:30 h./ Ope.: AUTOSERVEI
Experiment: A-H1-C13-hsqcedet-garp Solvent: CDCl3
f2 (ppm)
f1 (ppm)

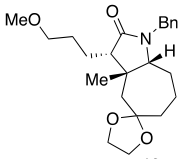

MeO
O
N
Bn
H
Me
O
O

19

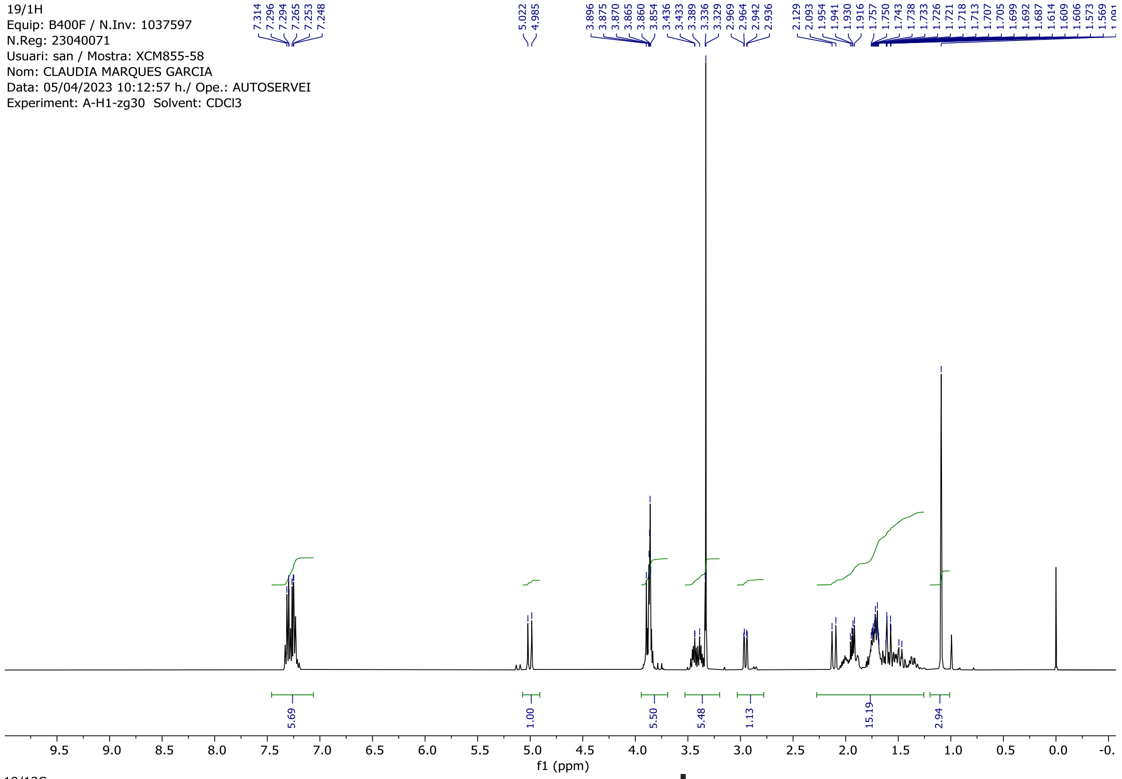

19/1H
Equip: B400F / N.Inv: 1037597
N.Reg: 23040071
Usuari: san / Mostra: XCM855-58
Nom: CLAUDIA MARQUES GARCIA
Data: 05/04/2023 10:12:57 h./ Ope.: AUTOSERVEI
Experiment: A-H1-zg30 Solvent: CDCl3
7.314
7.296
7.294
7.265
7.253
7.248
5.022
4.985
3.896
3.875
3.870
3.865
3.860
3.854
3.436
3.433
3.389
3.336
3.329
2.969
2.964
2.942
2.936
2.129
2.093
1.954
1.941
1.930
1.916
1.757
1.750
1.743
1.738
1.733
1.726
1.721
1.718
1.713
1.707
1.705
1.699
1.692
1.687
1.614
1.609
1.606
1.573
1.569
5.69
1.00
5.50
5.48
1.13
15.19
2.94
9.5
9.0
8.5
8.0
7.5
7.0
6.5
6.0
5.5
5.0
4.5
4.0
3.5
3.0
2.5
2.0
1.5
1.0
0.5
0.0
-0.
f1 (ppm)

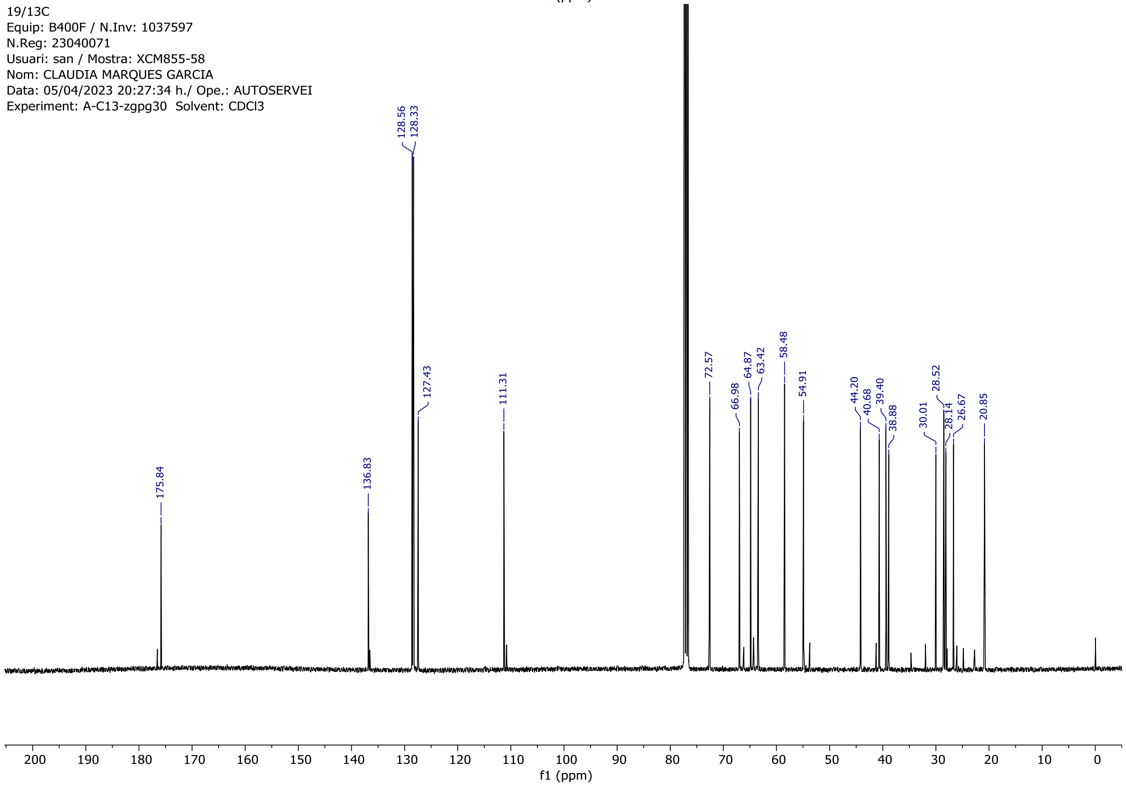

19/13C
Equip: B400F / N.Inv: 1037597
N.Reg: 23040071
Usuari: san / Mostra: XCM855-58
Nom: CLAUDIA MARQUES GARCIA
Data: 05/04/2023 20:27:34 h./ Ope.: AUTOSERVEI
Experiment: A-C13-zgpg30 Solvent: CDCl3
175.84
136.83
128.56
128.33
127.43
111.31
72.57
66.98
64.87
63.42
58.48
54.91
44.20
40.68
39.40
38.88
30.01
28.52
28.14
26.67
20.85
200
190
180
170
160
150
140
130
120
110
100
90
80
70
60
50
40
30
20
10
0
f1 (ppm)

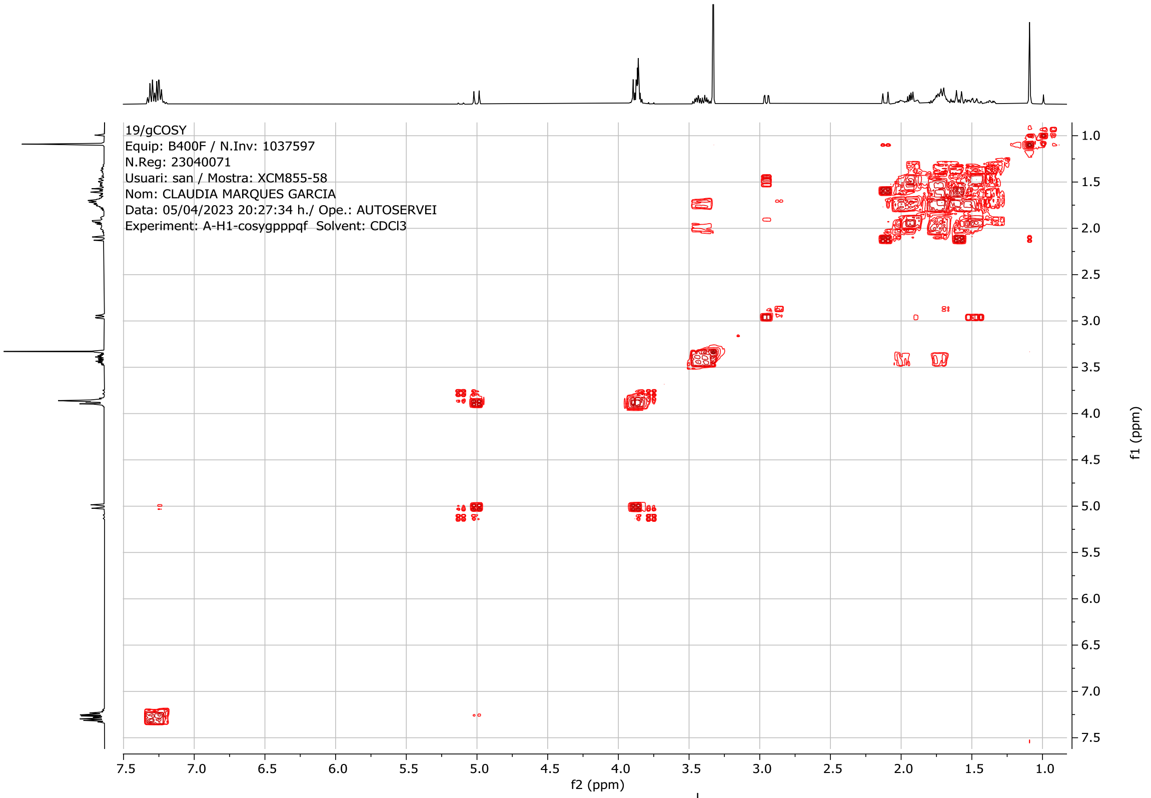
19/gCOSY
Equip: B400F / N.Inv: 1037597
N.Reg: 23040071
Usuari: san / Mostra: XCM855-58
Nom: CLAUDIA MARQUES GARCIA
Data: 05/04/2023 20:27:34 h./ Ope.: AUTOSERVEI
Experiment: A-H1-cosygpppqf Solvent: CDCl3
f1 (ppm)
f2 (ppm)

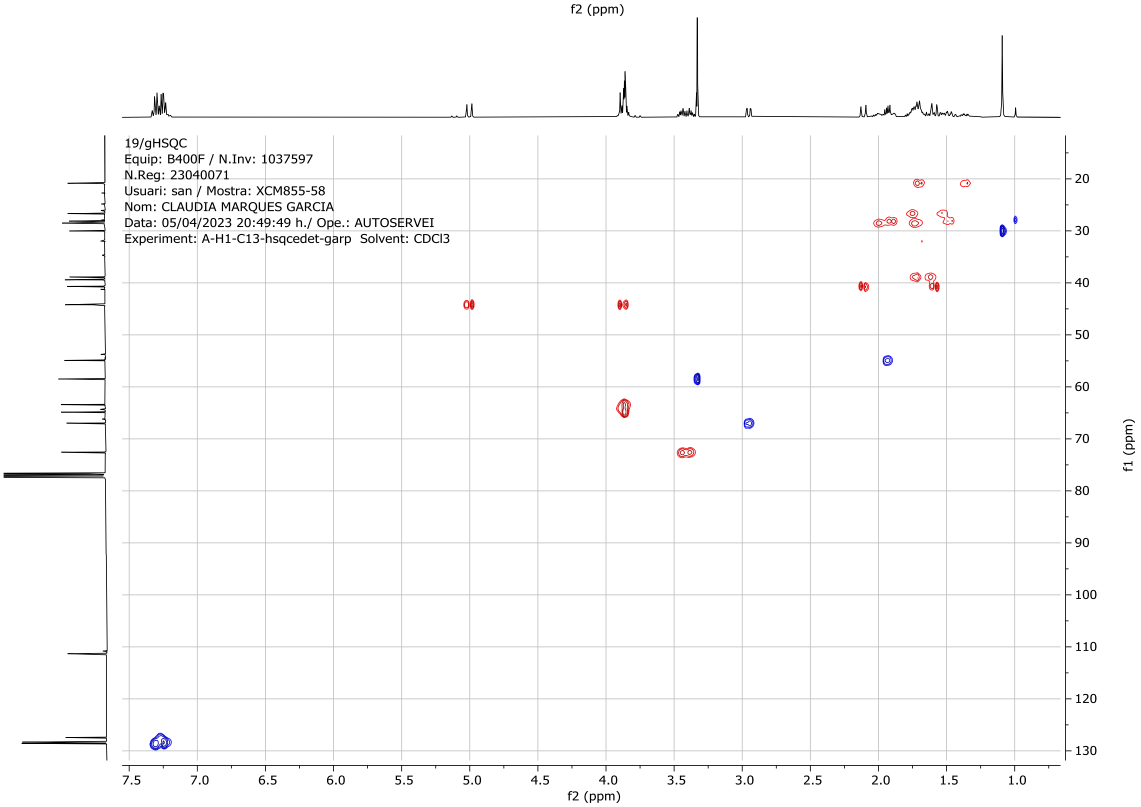
19/gHSQC
Equip: B400F / N.Inv: 1037597
N.Reg: 23040071
Usuari: san / Mostra: XCM855-58
Nom: CLAUDIA MARQUES GARCIA
Data: 05/04/2023 20:49:49 h./ Ope.: AUTOSERVEI
Experiment: A-H1-C13-hsqcedet-garp Solvent: CDCl3
f1 (ppm)
f2 (ppm)

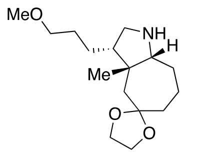
MeO
NH
H
Me
O
O

21

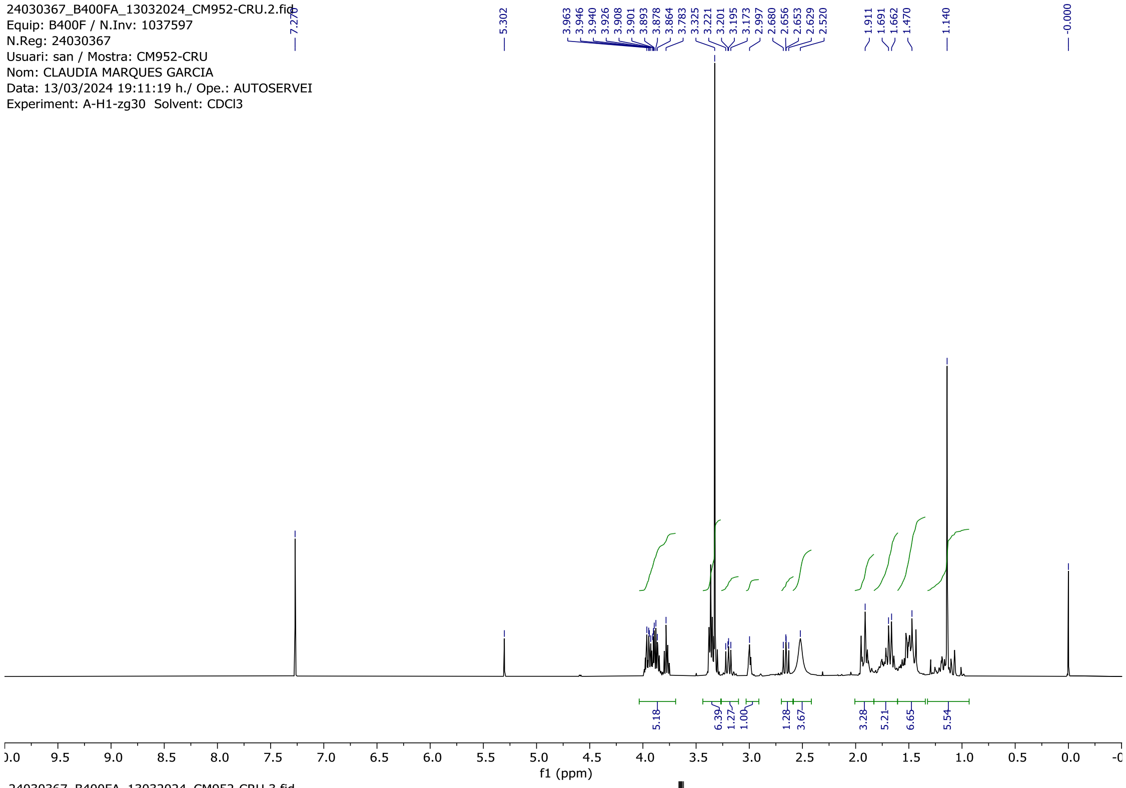
24030367_B400FA_13032024_CM952-CRU.2.fid
Equip: B400F / N.Inv: 1037597
N.Reg: 24030367
Usuari: san / Mostra: CM952-CRU
Nom: CLAUDIA MARQUES GARCIA
Data: 13/03/2024 19:11:19 h./ Ope.: AUTOSERVEI
Experiment: A-H1-zg30 Solvent: CDCl3
7.270
5.302
3.963 3.946 3.940 3.926 3.908 3.901 3.893 3.878 3.864 3.783 3.325 3.221 3.201 3.195 3.173 2.997 2.680 2.656 2.653 2.629 2.520
1.911 1.691 1.662 1.470
1.140
-0.000
5.18
6.39 1.27 1.00
1.28 3.67
3.28 5.21 6.65 5.54
f1 (ppm)

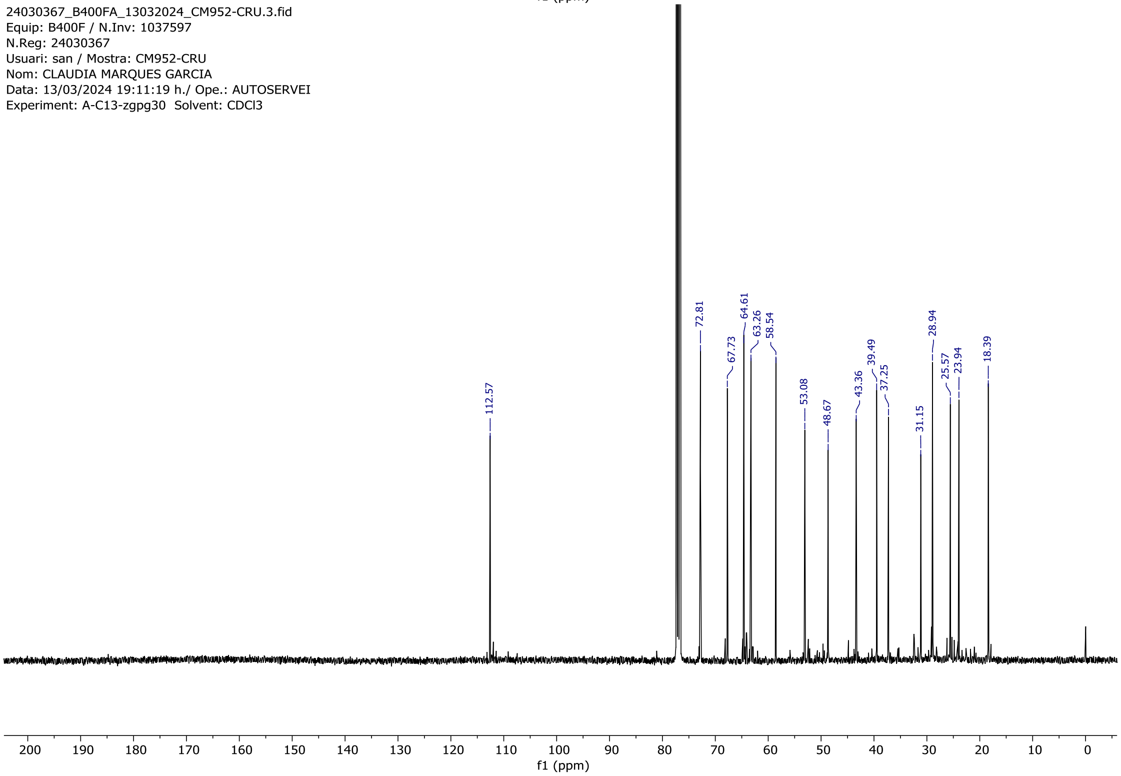
24030367_B400FA_13032024_CM952-CRU.3.fid
Equip: B400F / N.Inv: 1037597
N.Reg: 24030367
Usuari: san / Mostra: CM952-CRU
Nom: CLAUDIA MARQUES GARCIA
Data: 13/03/2024 19:11:19 h./ Ope.: AUTOSERVEI
Experiment: A-C13-zgpg30 Solvent: CDCl3
112.57
72.81
67.73
64.61
63.26
58.54
53.08
48.67
43.36
39.49
37.25
31.15
28.94
25.57
23.94
18.39
f1 (ppm)

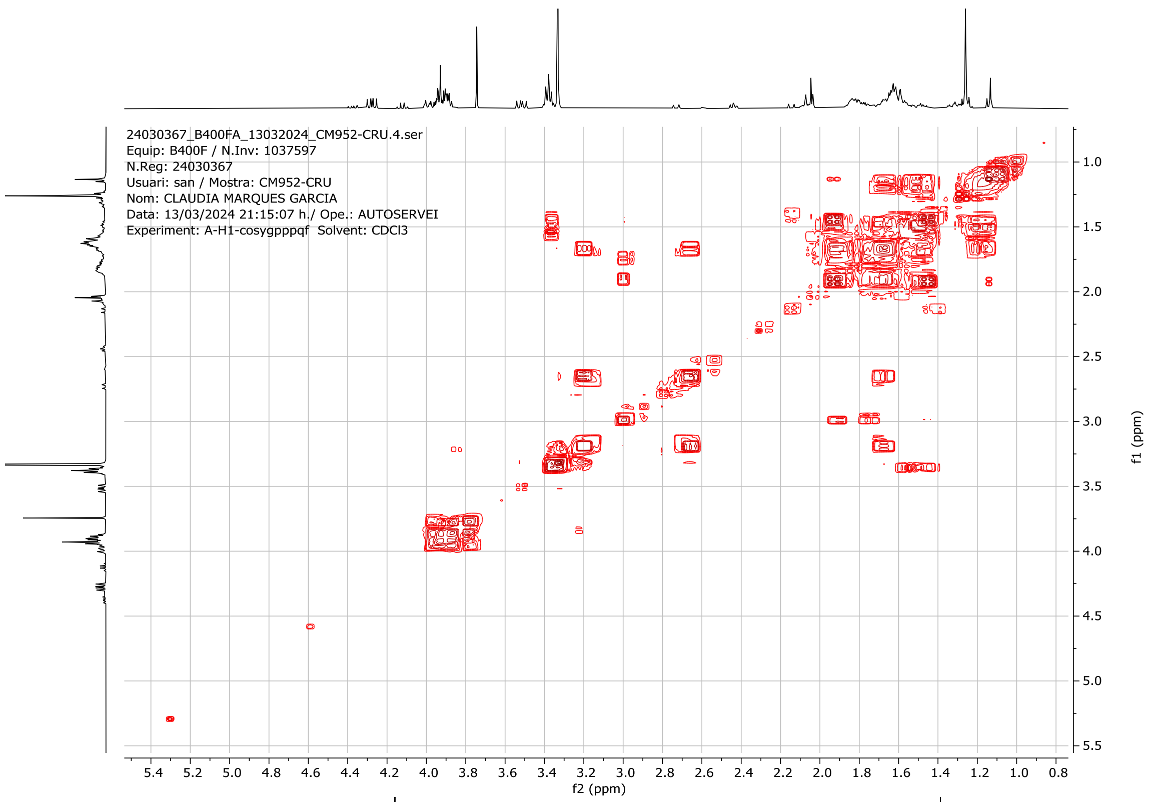

24030367_B400FA_13032024_CM952-CRU.4.ser
Equip: B400F / N.Inv: 1037597
N.Reg: 24030367
Usuari: san / Mostra: CM952-CRU
Nom: CLAUDIA MARQUES GARCIA
Data: 13/03/2024 21:15:07 h./ Ope.: AUTOSERVEI
Experiment: A-H1-cosygpppqf Solvent: CDCl3
f2 (ppm)
f1 (ppm)

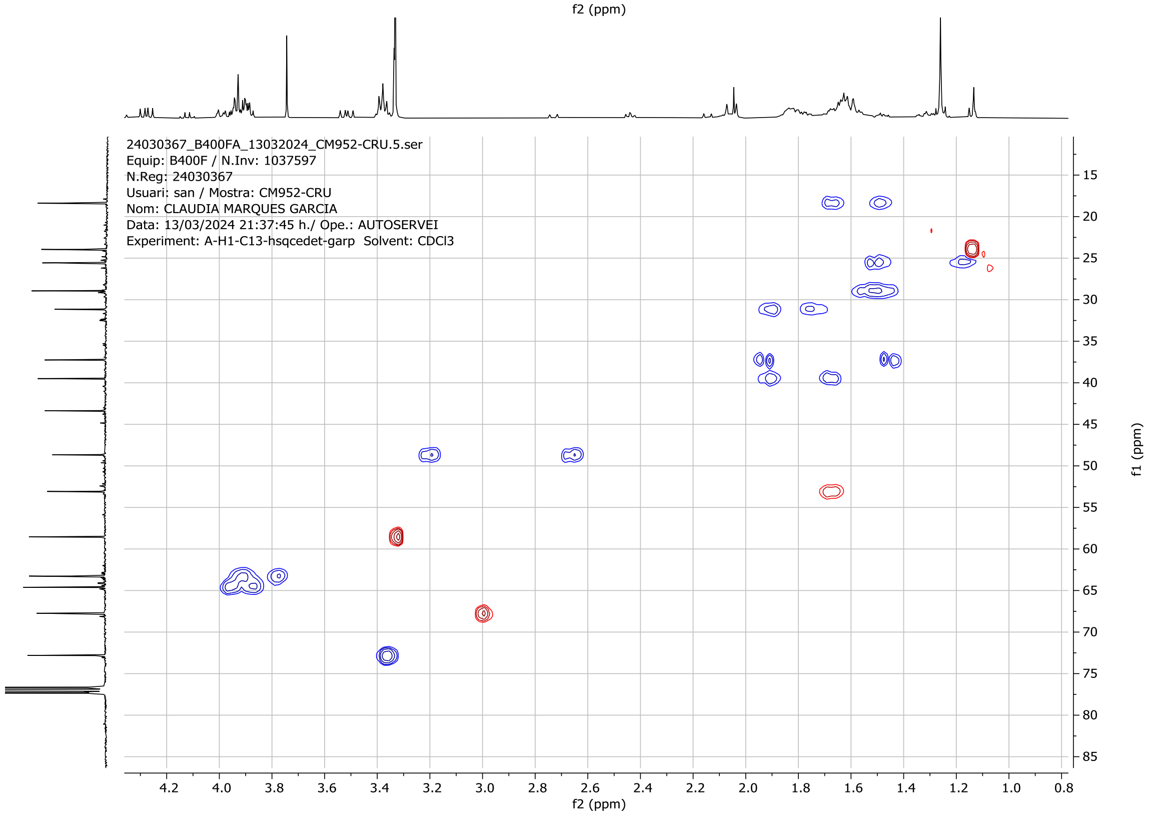

24030367_B400FA_13032024_CM952-CRU.5.ser
Equip: B400F / N.Inv: 1037597
N.Reg: 24030367
Usuari: san / Mostra: CM952-CRU
Nom: CLAUDIA MARQUES GARCIA
Data: 13/03/2024 21:37:45 h./ Ope.: AUTOSERVEI
Experiment: A-H1-C13-hsqcedet-garp Solvent: CDCl3
f2 (ppm)
f1 (ppm)

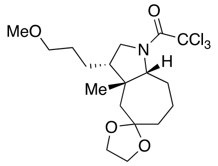

22

22/1H
Equip: B400F / N.Inv: 1037597
N.Reg: 24030299
Usuari: san / Mostra: XCM955-18
Nom: CLAUDIA MARQUES GARCIA
Data: 12/03/2024 11:25:38 h./ Ope.: AUTOSERVEI
Experiment: A-H1-zg30 Solvent: CDCl3

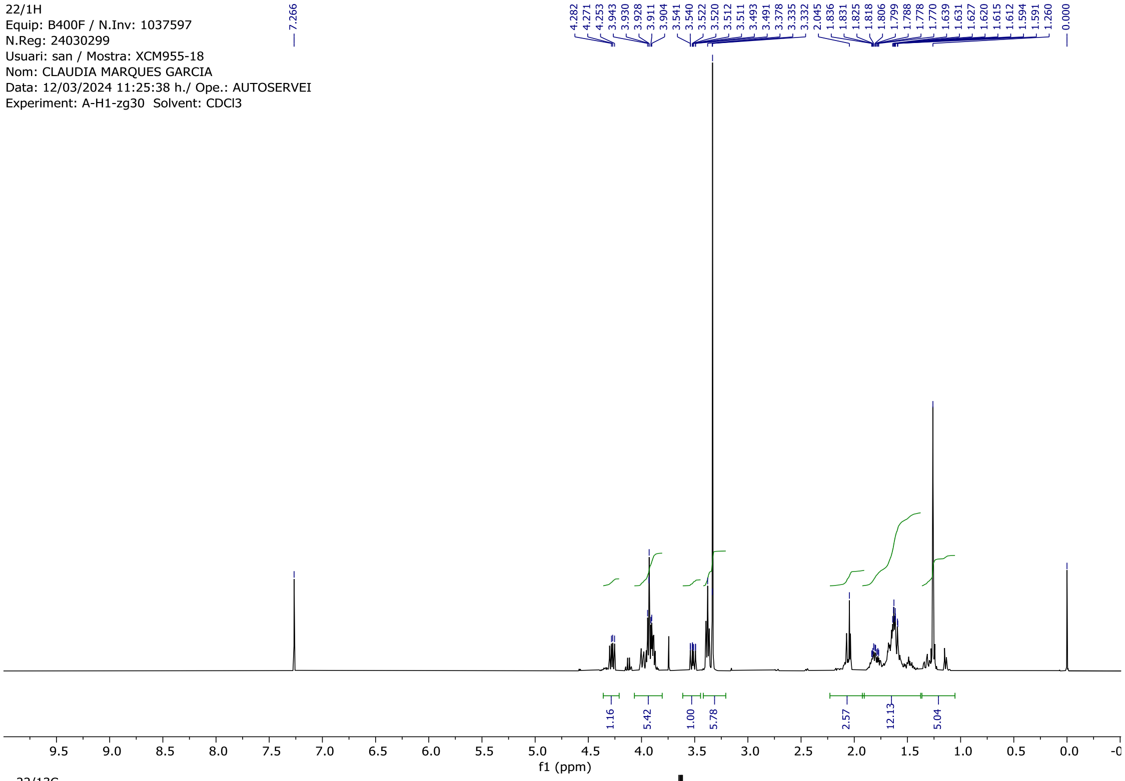

22/13C
Equip: B400F / N.Inv: 1037597
N.Reg: 24030299
Usuari: san / Mostra: XCM955-18
Nom: CLAUDIA MARQUES GARCIA
Data: 13/03/2024 00:50:43 h./ Ope.: AUTOSERVEI
Experiment: A-C13-zgpg30 Solvent: CDCl3

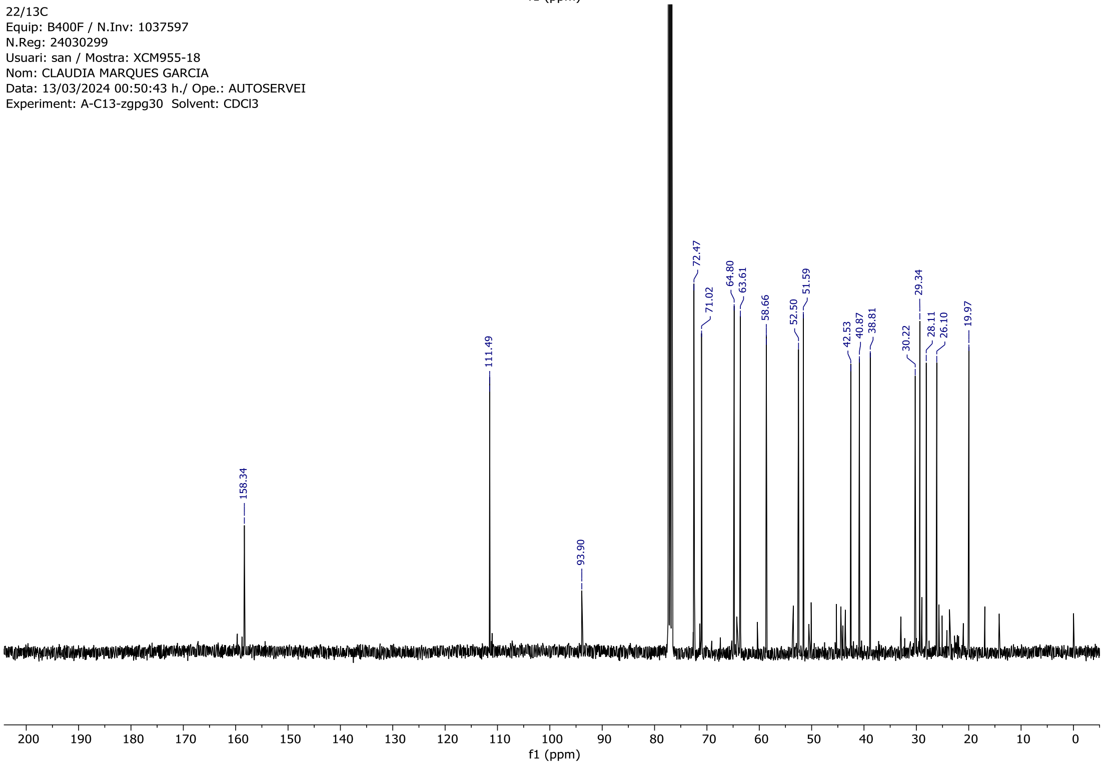

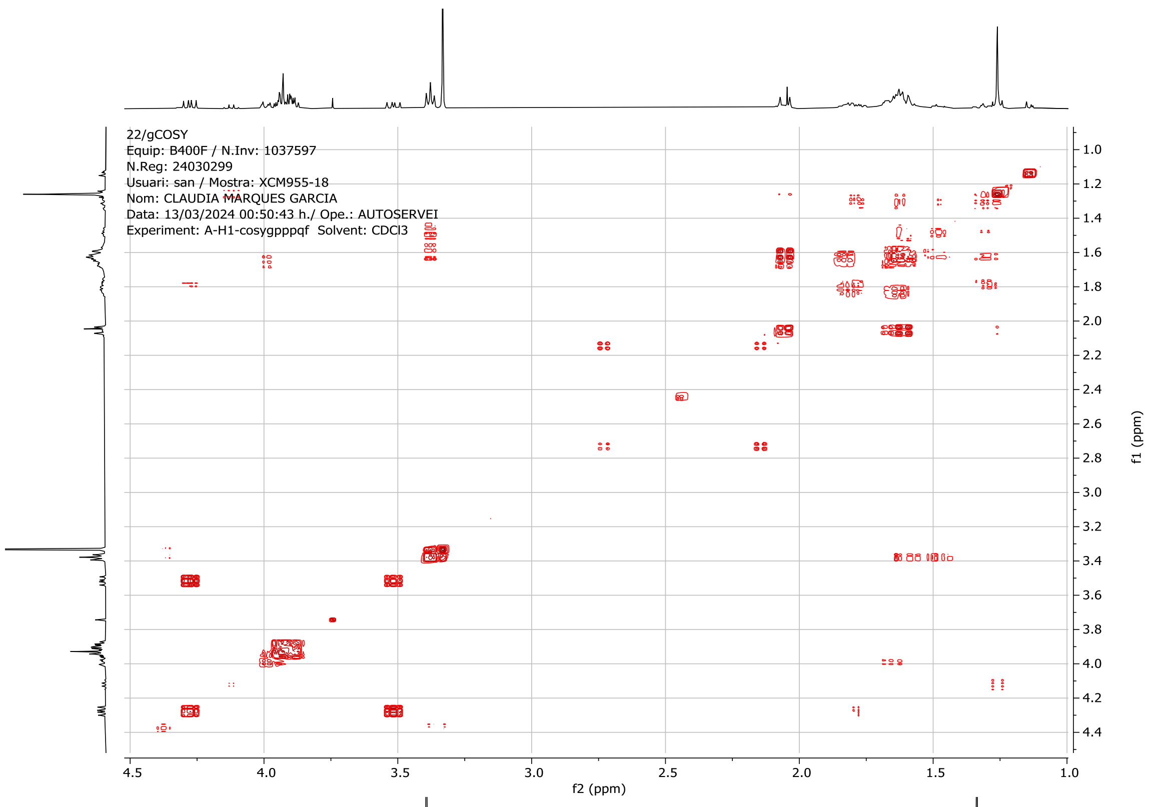
22/gCOSY
Equip: B400F / N.Inv: 1037597
N.Reg: 24030299
Usuari: san / Mostra: XCM955-18
Nom: CLAUDIA MARQUES GARCIA
Data: 13/03/2024 00:50:43 h./ Ope.: AUTOSERVEI
Experiment: A-H1-cosygpppqf Solvent: CDCl3
f1 (ppm)
f2 (ppm)

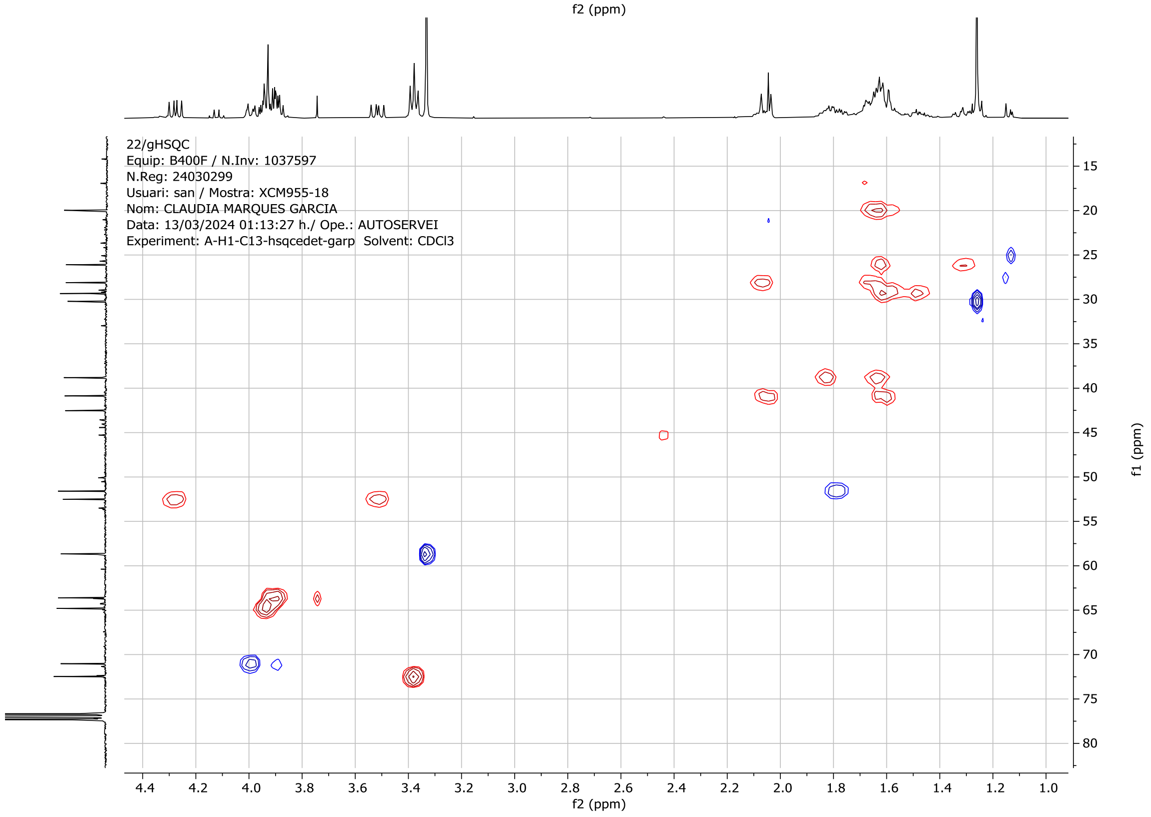
22/gHSQC
Equip: B400F / N.Inv: 1037597
N.Reg: 24030299
Usuari: san / Mostra: XCM955-18
Nom: CLAUDIA MARQUES GARCIA
Data: 13/03/2024 01:13:27 h./ Ope.: AUTOSERVEI
Experiment: A-H1-C13-hsqcedet-garp Solvent: CDCl3
f1 (ppm)
f2 (ppm)

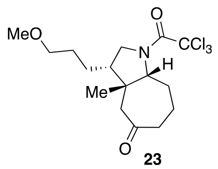

23

23/1H
Equip: B400F / N.Inv: 1037597
N.Reg: 23050387
Usuari: san / Mostra: XCM881-37
Nom: CLAUDIA MARQUES GARCIA
Data: 14/05/2023 05:25:45 h./ Ope.: AUTOSERVEI
Experiment: A-H1-zg30 Solvent: CDCl3

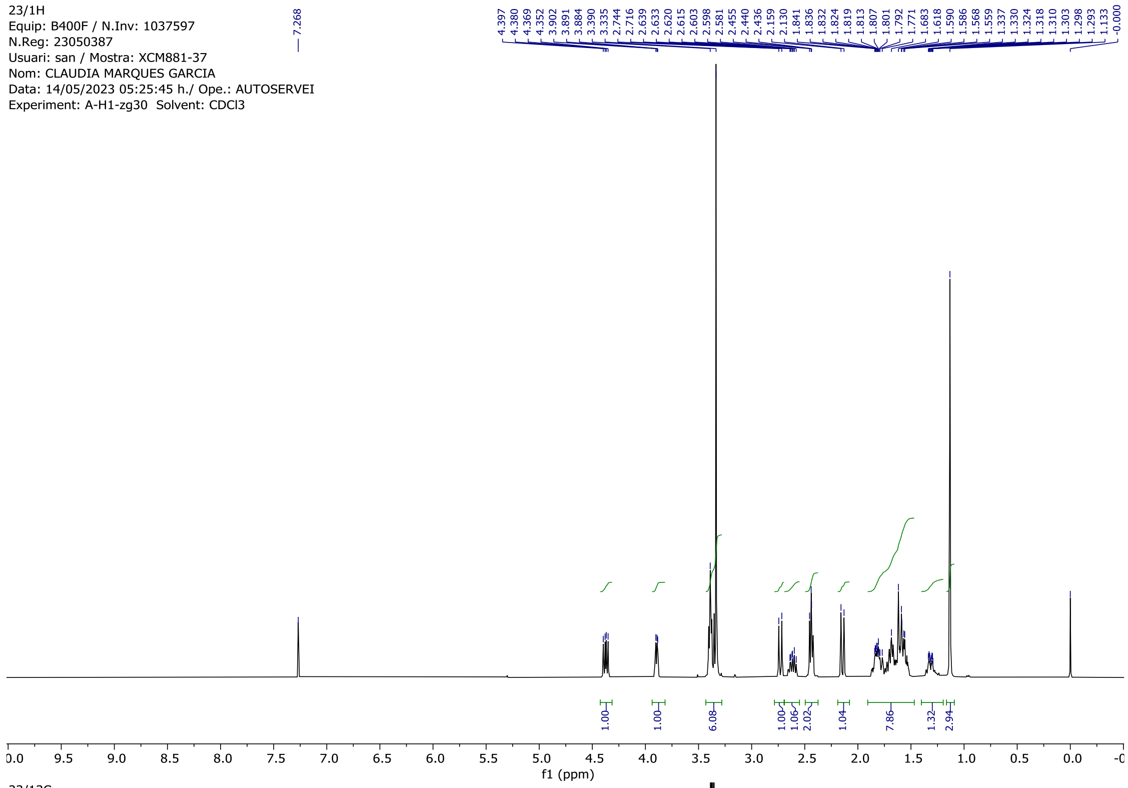

23/13C
Equip: B400F / N.Inv: 1037597
N.Reg: 23050387
Usuari: san / Mostra: XCM881-37
Nom: CLAUDIA MARQUES GARCIA
Data: 14/05/2023 05:25:45 h./ Ope.: AUTOSERVEI
Experiment: A-C13-zgpg30 Solvent: CDCl3

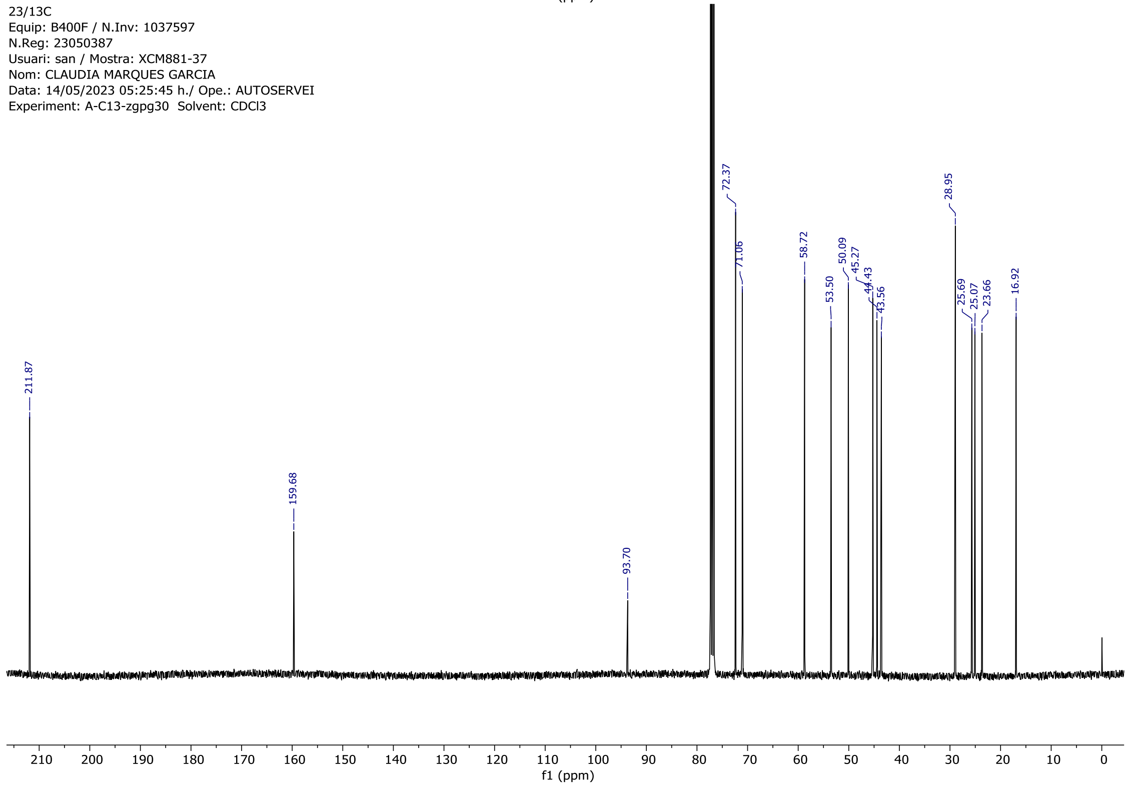

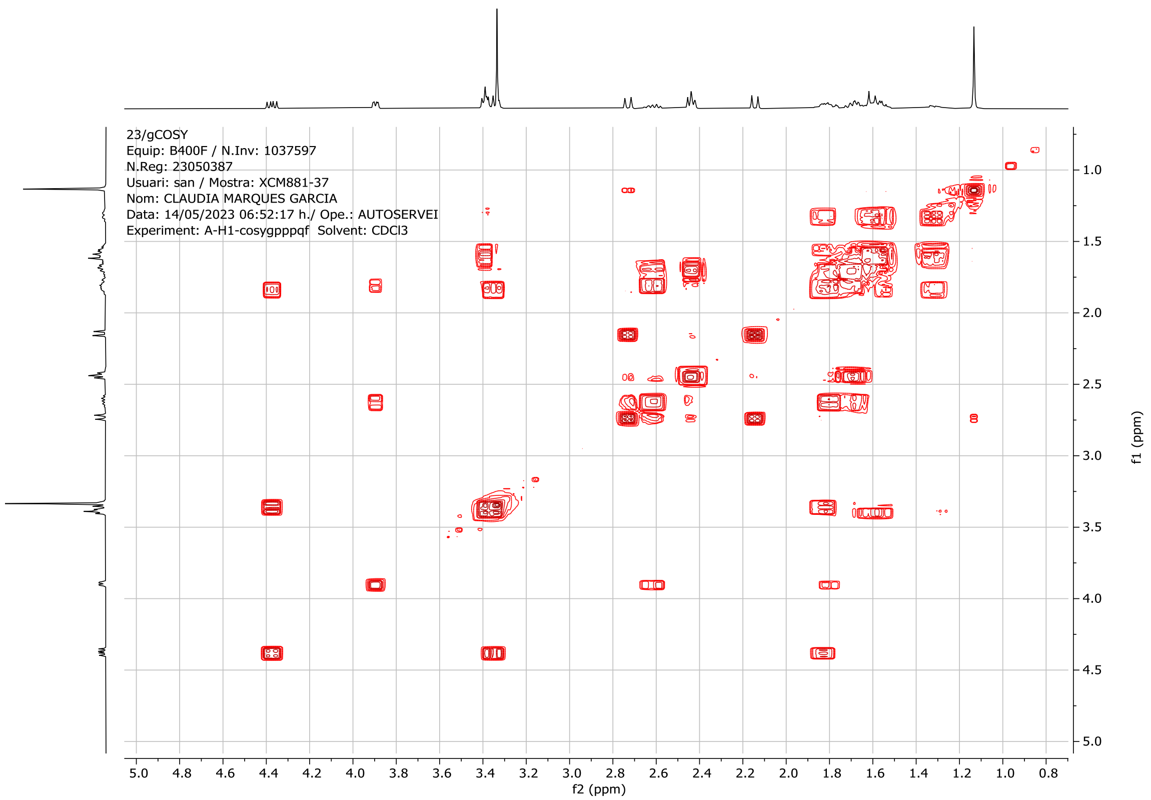
23/gCOSY
Equip: B400F / N.Inv: 1037597
N.Reg: 23050387
Usuari: san / Mostra: XCM881-37
Nom: CLAUDIA MARQUES GARCIA
Data: 14/05/2023 06:52:17 h./ Ope.: AUTOSERVEI
Experiment: A-H1-cosygpppqf Solvent: CDCl3
f2 (ppm)
f1 (ppm)

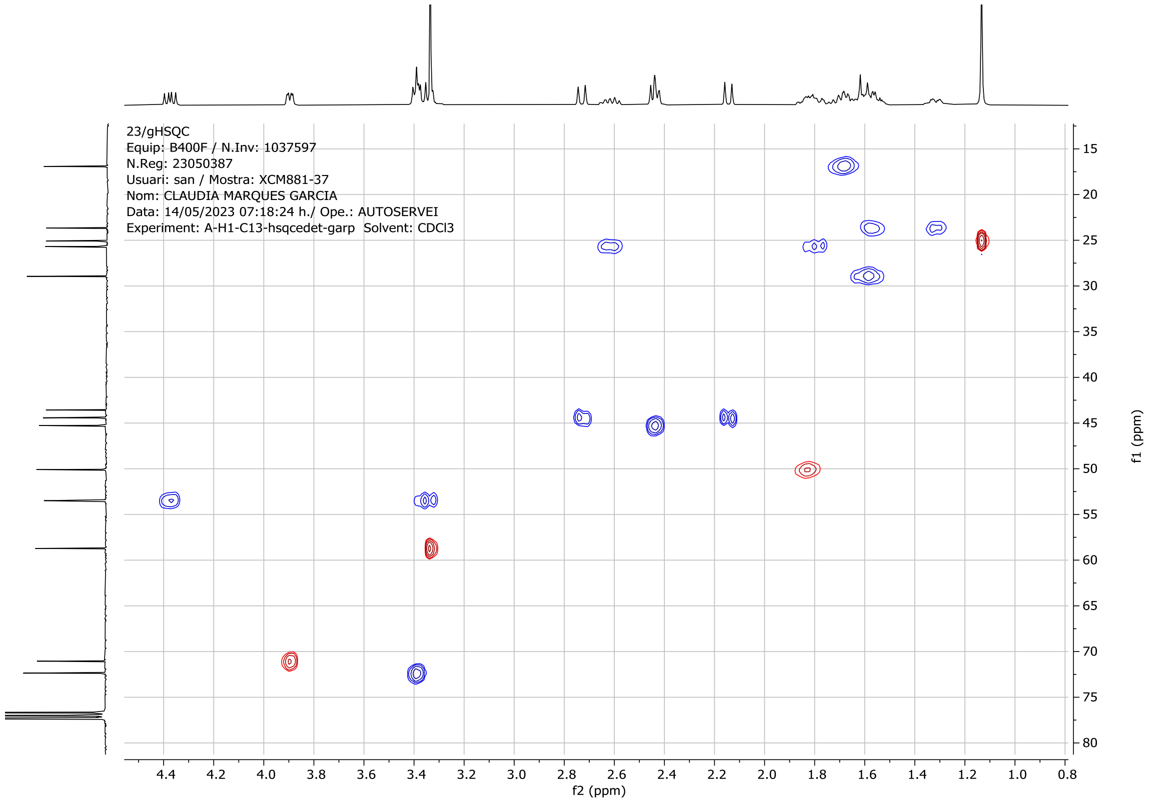
23/gHSQC
Equip: B400F / N.Inv: 1037597
N.Reg: 23050387
Usuari: san / Mostra: XCM881-37
Nom: CLAUDIA MARQUES GARCIA
Data: 14/05/2023 07:18:24 h./ Ope.: AUTOSERVEI
Experiment: A-H1-C13-hsqcedet-garp Solvent: CDCl3
f2 (ppm)
f1 (ppm)

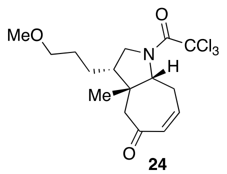

24

24/1H
Equip: B400F / N.Inv: 1037597
N.Reg: 23050554
Usuari: san / Mostra: XCM882-B36
Nom: CLAUDIA MARQUES GARCIA
Data: 17/05/2023 16:32:49 h./ Ope.: AUTOSERVEI
Experiment: A-H1-zg30 Solvent: CDCl3

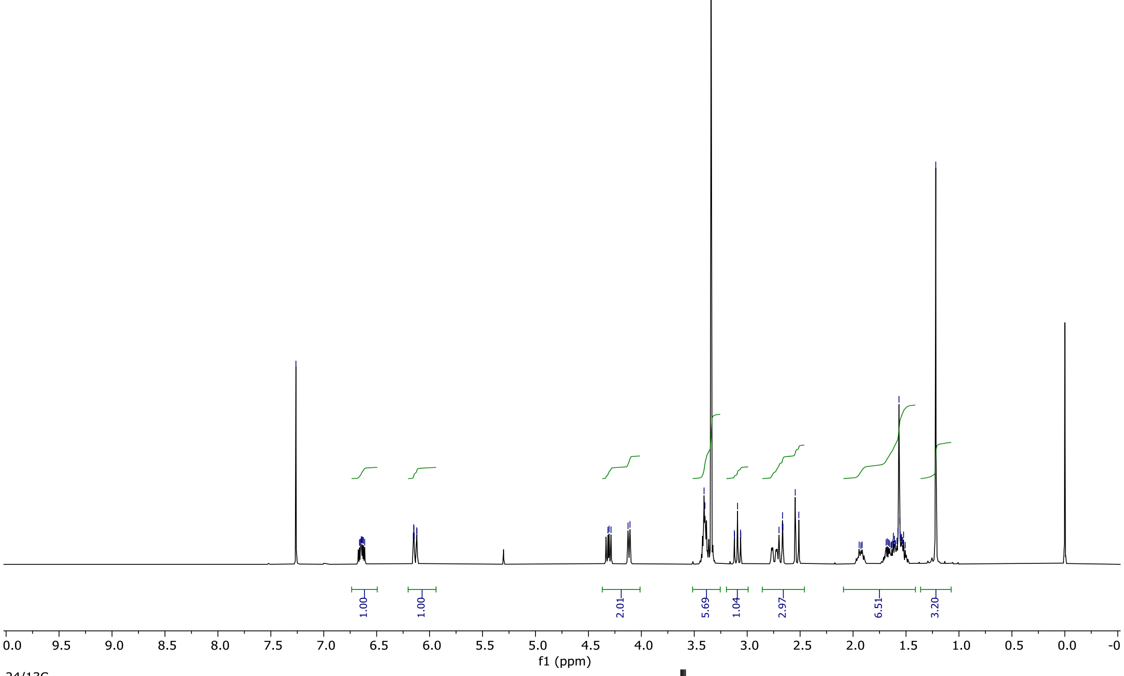

24/13C
Equip: B400F / N.Inv: 1037597
N.Reg: 23050554
Usuari: san / Mostra: XCM882-B36
Nom: CLAUDIA MARQUES GARCIA
Data: 18/05/2023 05:01:36 h./ Ope.: AUTOSERVEI
Experiment: A-C13-zgpg30 Solvent: CDCl3

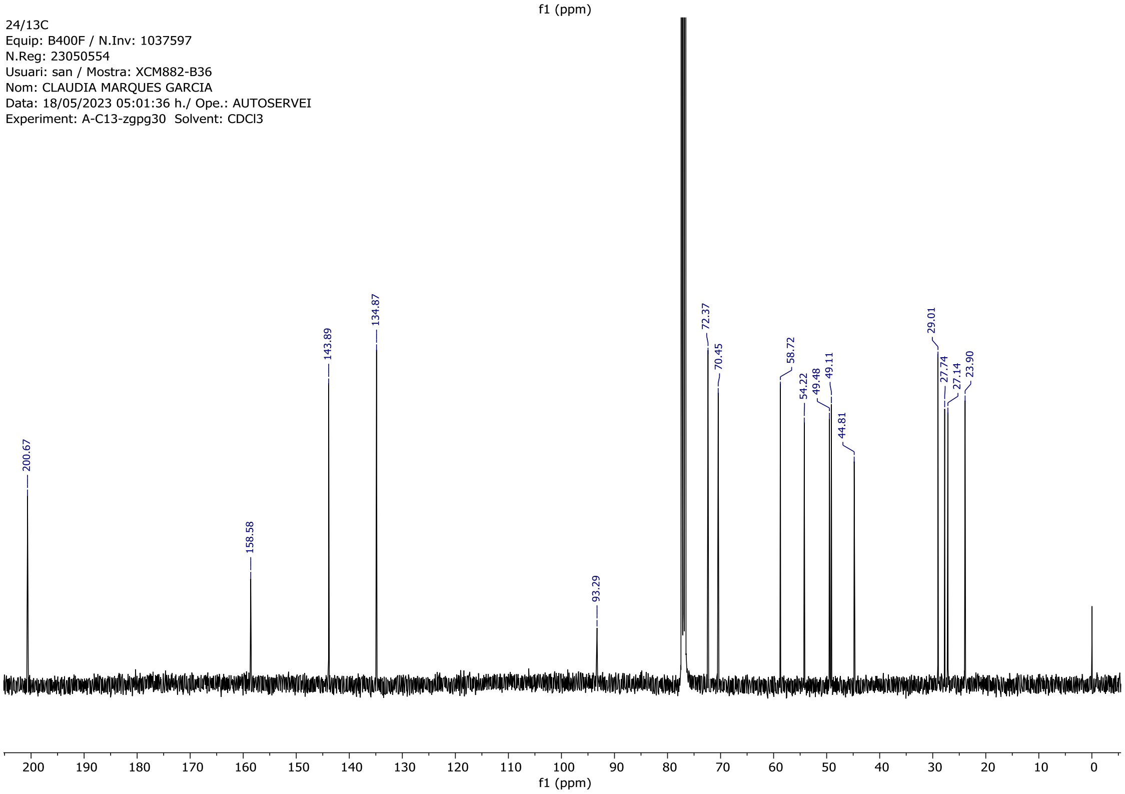

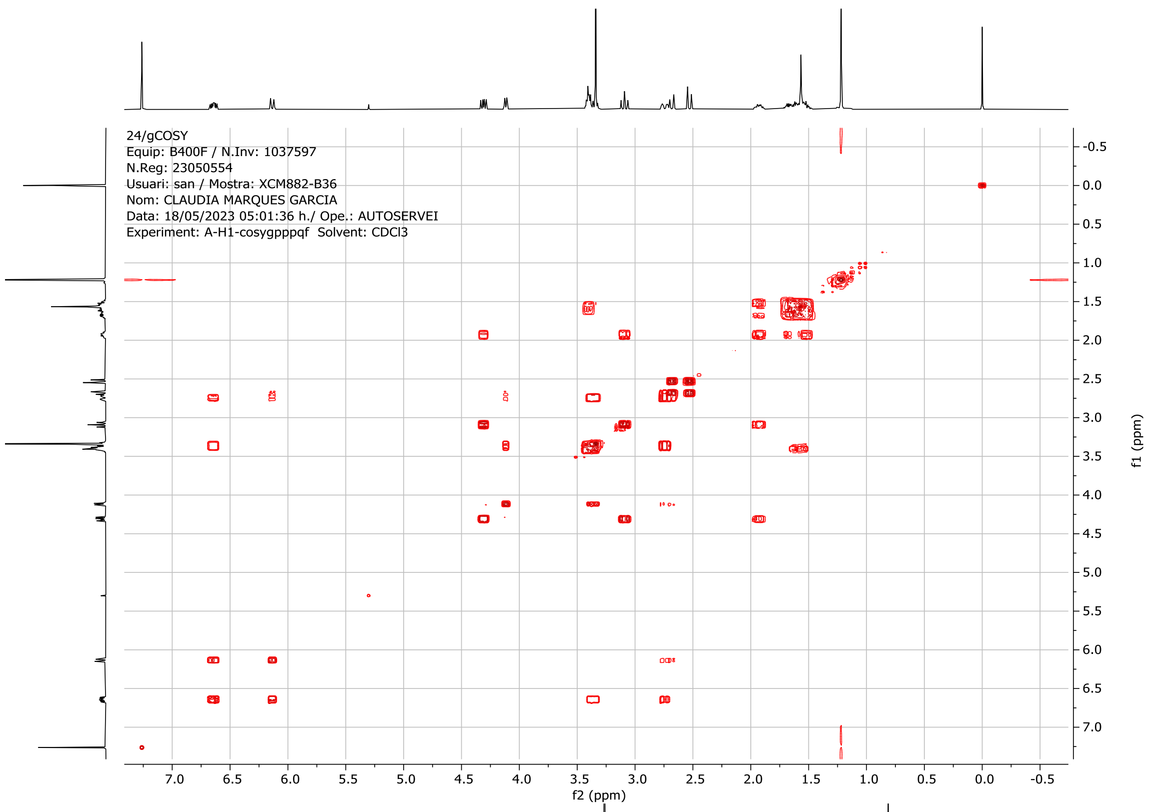
24/gCOSY
Equip: B400F / N.Inv: 1037597
N.Reg: 23050554
Usuari: san / Mostra: XCM882-B36
Nom: CLAUDIA MARQUES GARCIA
Data: 18/05/2023 05:01:36 h./ Ope.: AUTOSERVEI
Experiment: A-H1-cosygpppqf Solvent: CDCl3
f2 (ppm)
f1 (ppm)

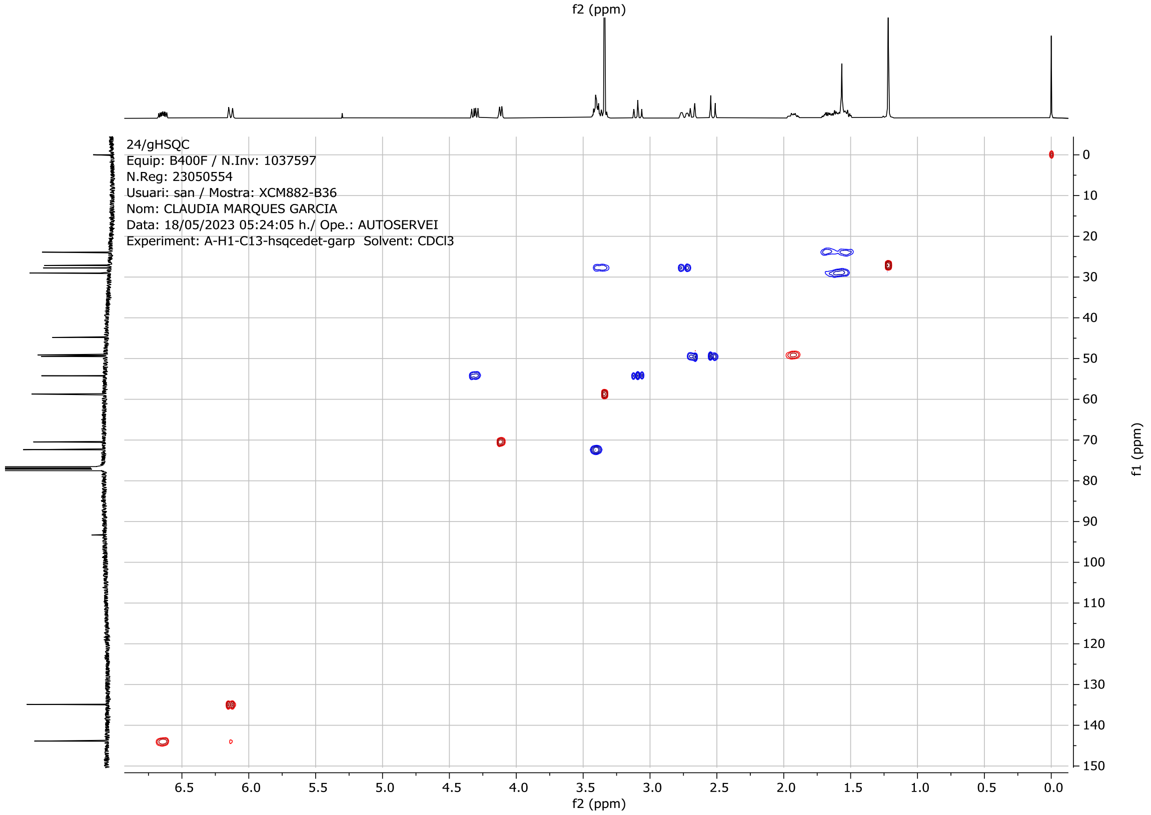
24/gHSQC
Equip: B400F / N.Inv: 1037597
N.Reg: 23050554
Usuari: san / Mostra: XCM882-B36
Nom: CLAUDIA MARQUES GARCIA
Data: 18/05/2023 05:24:05 h./ Ope.: AUTOSERVEI
Experiment: A-H1-C13-hsqcedet-garp Solvent: CDCl3
f2 (ppm)
f1 (ppm)

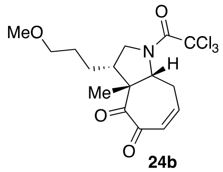

24b

24b/1H
Equip: B400F / N.Inv: 1037597
N.Reg: 23050643
Usuari: san / Mostra: XCM882-B13
Nom: CLAUDIA MARQUES GARCIA
Data: 19/05/2023 18:27:14 h./ Ope.: AUTOSERVEI
Experiment: A-H1-zg30 Solvent: CDCl3

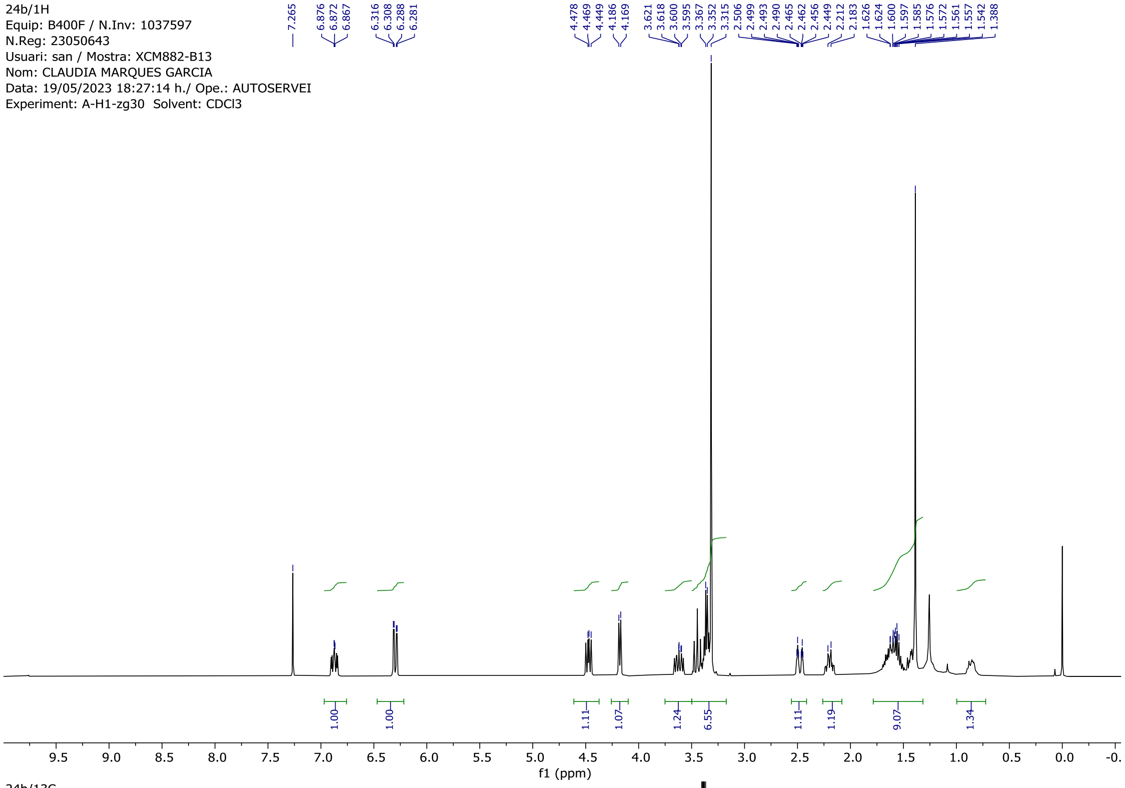

24b/13C
Equip: B400F / N.Inv: 1037597
N.Reg: 23050643
Usuari: san / Mostra: XCM882-B13
Nom: CLAUDIA MARQUES GARCIA
Data: 19/05/2023 21:06:30 h./ Ope.: AUTOSERVEI
Experiment: A-C13-zgpg30 Solvent: CDCl3

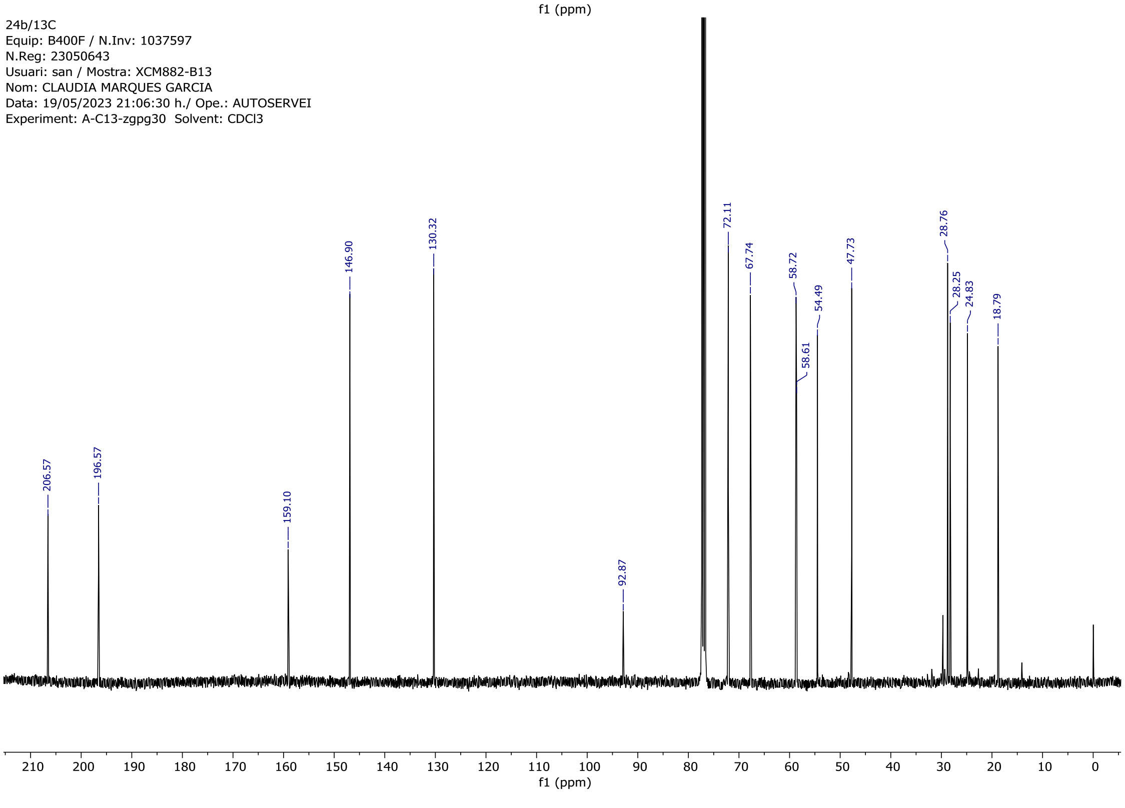

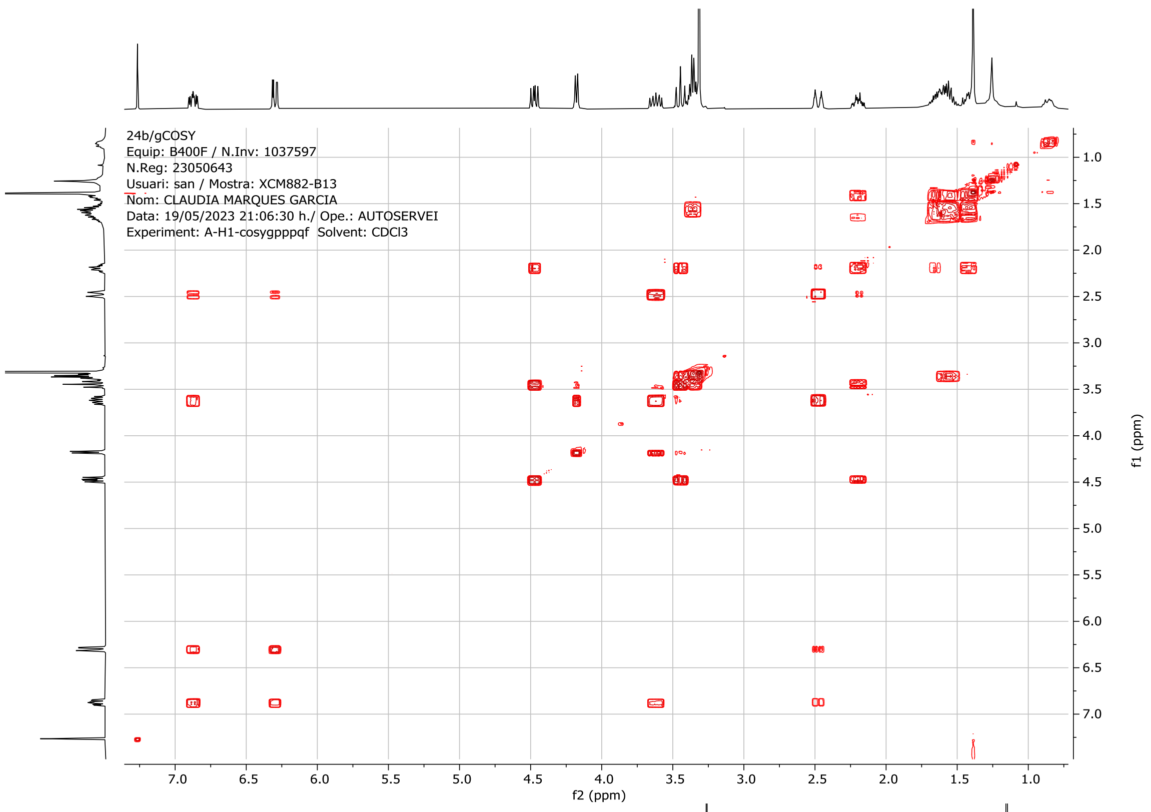
24b/gCOSY
Equip: B400F / N.Inv: 1037597
N.Reg: 23050643
Usuari: san / Mostra: XCM882-B13
Nom: CLAUDIA MARQUES GARCIA
Data: 19/05/2023 21:06:30 h./ Ope.: AUTOSERVEI
Experiment: A-H1-cosygpppqf Solvent: CDCl3
f2 (ppm)
f1 (ppm)

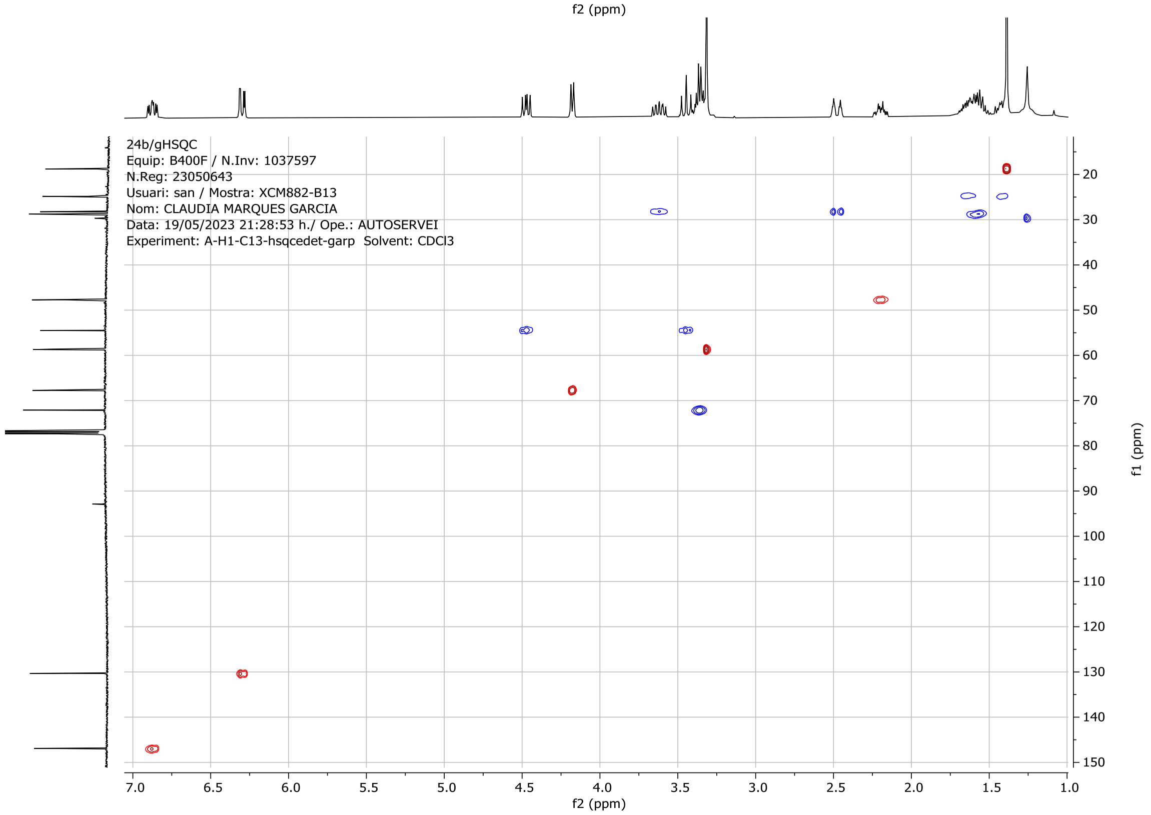
24b/gHSQC
Equip: B400F / N.Inv: 1037597
N.Reg: 23050643
Usuari: san / Mostra: XCM882-B13
Nom: CLAUDIA MARQUES GARCIA
Data: 19/05/2023 21:28:53 h./ Ope.: AUTOSERVEI
Experiment: A-H1-C13-hsqcedet-garp Solvent: CDCl3
f2 (ppm)
f1 (ppm)

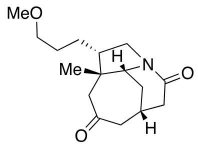

25

25/1H
Equip: B400F / N.Inv: 1037597
N.Reg: 23040613
Usuari: san / Mostra: XCM864-B16
Nom: CLAUDIA MARQUES GARCIA
Data: 26/04/2023 12:00:09 h./ Ope.: AUTOSERVEI
Experiment: A-H1-zg30 Solvent: CDCl3

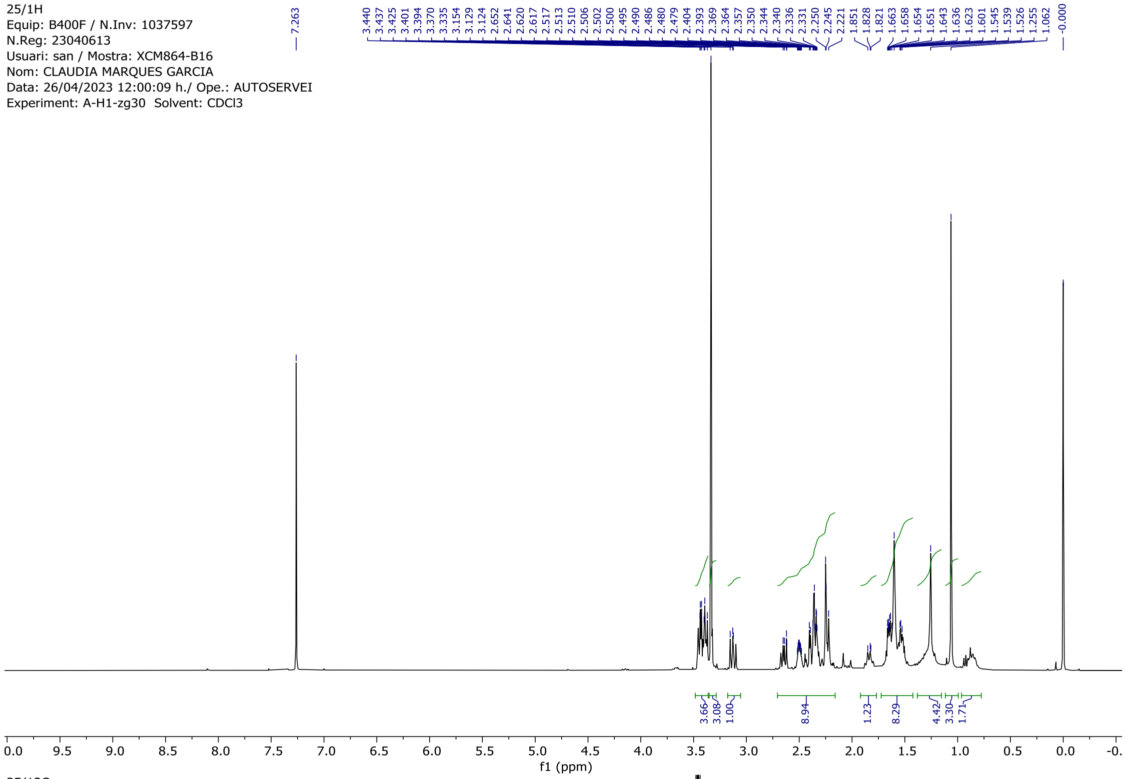

25/13C
Equip: B400F / N.Inv: 1037597
N.Reg: 23040613
Usuari: san / Mostra: XCM864-B16
Nom: CLAUDIA MARQUES GARCIA
Data: 27/04/2023 01:39:47 h./ Ope.: AUTOSERVEI
Experiment: A-C13-zgpg30 Solvent: CDCl3

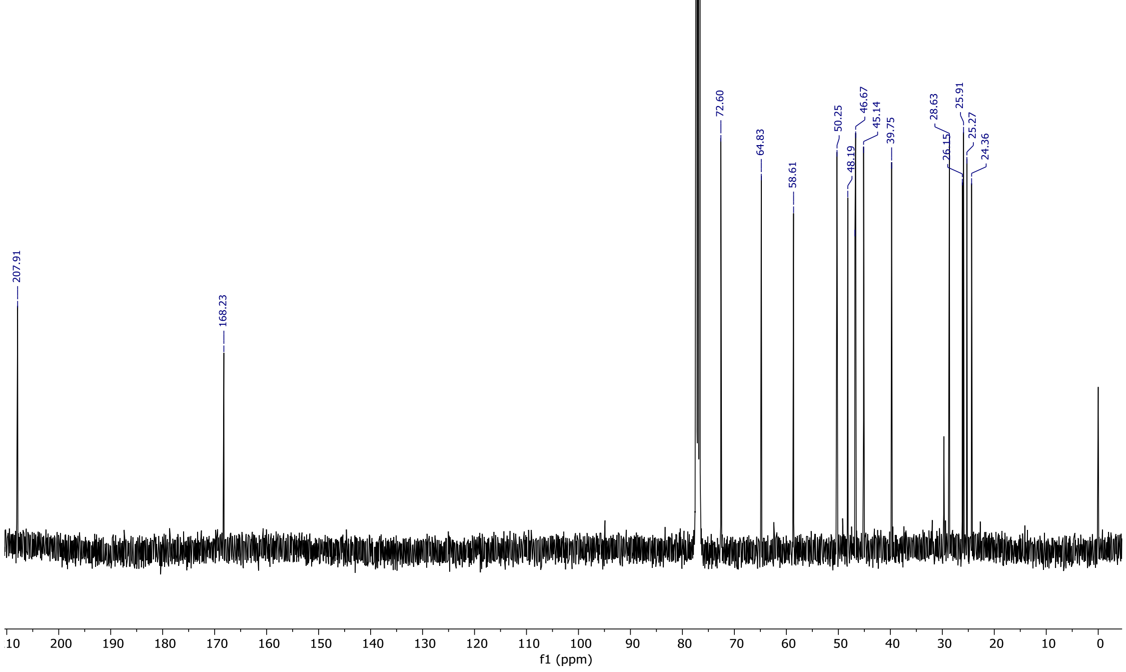

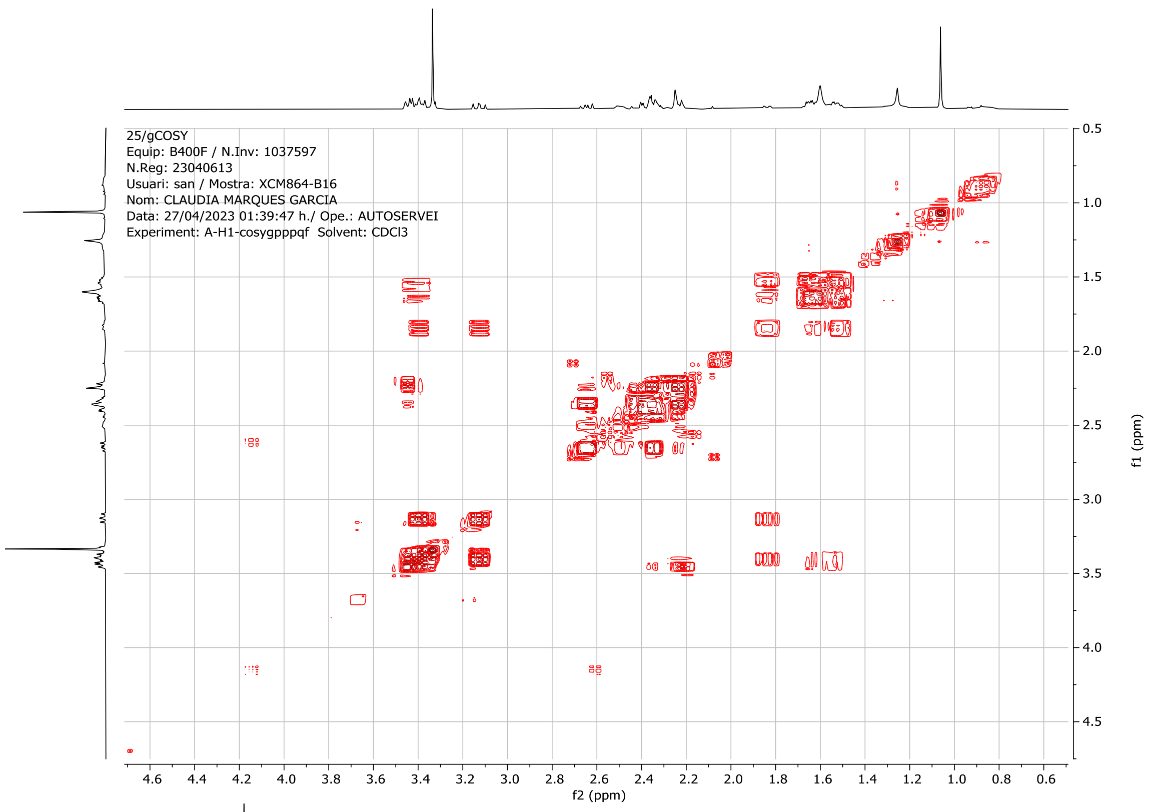
25/gCOSY
Equip: B400F / N.Inv: 1037597
N.Reg: 23040613
Usuari: san / Mostra: XCM864-B16
Nom: CLAUDIA MARQUES GARCIA
Data: 27/04/2023 01:39:47 h./ Ope.: AUTOSERVEI
Experiment: A-H1-cosygpppqf Solvent: CDCl3
f2 (ppm)
f1 (ppm)

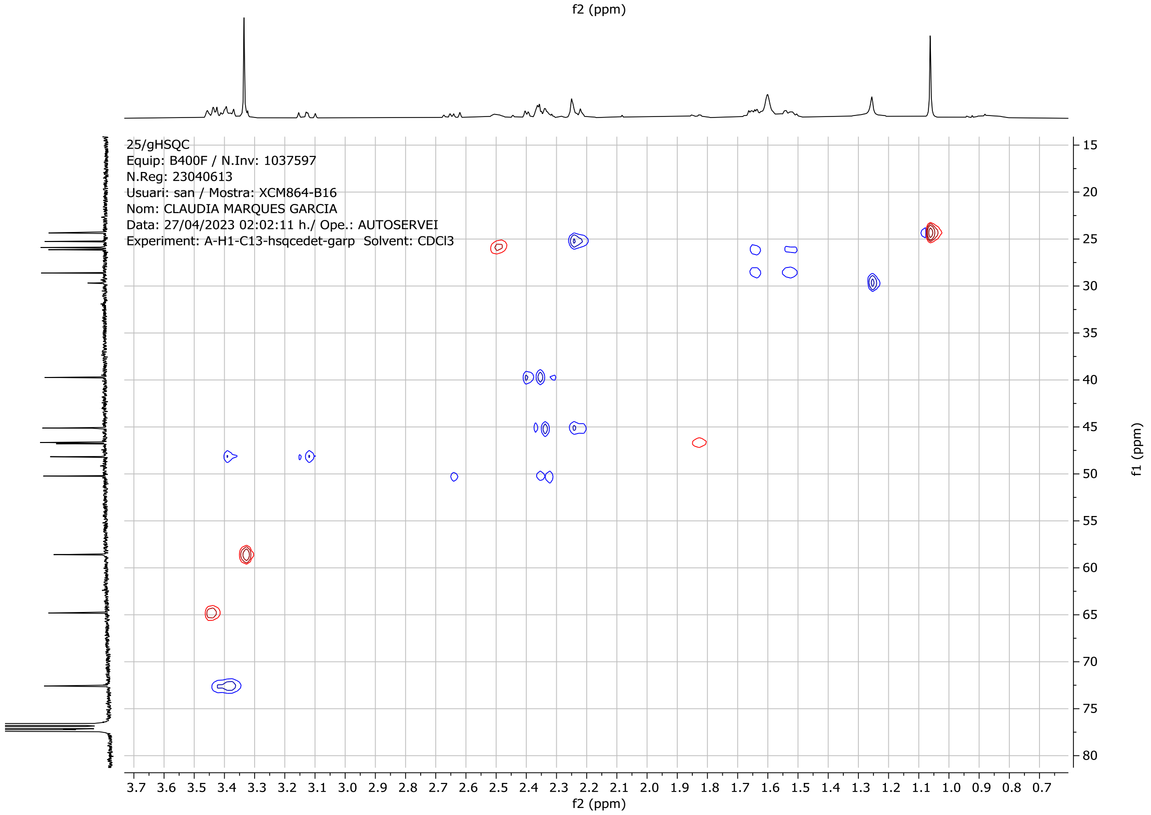
25/gHSQC
Equip: B400F / N.Inv: 1037597
N.Reg: 23040613
Usuari: san / Mostra: XCM864-B16
Nom: CLAUDIA MARQUES GARCIA
Data: 27/04/2023 02:02:11 h./ Ope.: AUTOSERVEI
Experiment: A-H1-C13-hsqcedet-garp Solvent: CDCl3
f2 (ppm)
f1 (ppm)
